# Synthesis and characterization of iron oxide nanoparticles from *Lawsonia inermis* and its effect on the biodegradation of crude oil hydrocarbon

**DOI:** 10.1038/s41598-024-61760-6

**Published:** 2024-05-17

**Authors:** Balakrishnan Muthukumar, Ramanathan Duraimurugan, Punniyakotti Parthipan, Rajaram Rajamohan, Rajakrishnan Rajagopal, Jayaraman Narenkumar, Aruliah Rajasekar, Tabarak Malik

**Affiliations:** 1 Environmental Molecular Microbiology Research Laboratory, Department of Biotechnology, Thiruvalluvar University, Serkkadu, Vellore, Tamil Nadu 632115 India; 2 Department of Biotechnology, Faculty of Science and Humanities, SRM Institute of Science and Technology, Kattankulathur, Chengalpattu, Tamil Nadu 603203 India; 3 Organic Materials Synthesis Lab, School of Chemical Engineering, Yeungnam University, Gyeongsan-si, 38541 Republic of Korea; 4 Department of Botany and Microbiology, College of Science, King Saud University, 11451 Riyadh, Saudi Arabia; 5 Department of Environmental & Water Resources Engineering, School of Civil Engineering (SCE), Vellore Institute of Technology, Vellore, Tamil Nadu 632014 India; 6 Present Address: Department of Biomedical Sciences, Institute of Health, Jimma University, 378, Jimma, Ethiopia; 7 Adjunct Faculty, Division of Research and Development, Lovely Professional University, Phagwara, Punjab 144411 India

**Keywords:** Crude oil contaminations, *Pseudomonas aeruginosa*, Biosurfactants, Iron oxide nanoparticles, Green synthesis, Surface analysis, Biotechnology, Environmental sciences, Nanoscience and technology

## Abstract

Crude oil hydrocarbons are considered major environmental pollutants and pose a significant threat to the environment and humans due to having severe carcinogenic and mutagenic effects. Bioremediation is one of the practical and promising technology that can be applied to treat the hydrocarbon-polluted environment. In this present study, rhamnolipid biosurfactant (BS) produced by *Pseudomonas aeruginosa* PP4 and green synthesized iron nanoparticles (G-FeNPs) from *Lawsonia inermis* was used to evaluate the biodegradation efficiency (BE) of crude oil. The surface analysis of G-FeNPs was carried out by using FESEM and HRTEM to confirm the size and shape. Further, the average size of the G-FeNPs was observed around 10 nm by HRTEM analysis. The XRD and Raman spectra strongly confirm the presence of iron nanoparticles with their respective peaks. The BE (%) of mixed degradation system-V (PP4+BS+G-FeNPs) was obtained about 82%. FTIR spectrum confirms the presence of major functional constituents (C=O, –CH_3_, C–O, and OH) in the residual oil content. Overall, this study illustrates that integrated nano-based bioremediation could be an efficient approach for hydrocarbon-polluted environments. This study is the first attempt to evaluate the G-FeNPs with rhamnolipid biosurfactant on the biodegradation of crude oil.

## Introduction

The spillage, discharge, and disposal of crude oil could cause serious threats to both the environment and humans^1,2^. In general, oil contamination is one of the major problems due to having a mixture of several components such as toxic heavy metals along with long-chain hydrocarbons, including alkanes, aromatics, and polyaromatic hydrocarbons, etc^3–6^. The presence of these groups of toxic compounds in the discharged crude oil may contribute to the contamination of water and soil by changing their physicochemical characteristics like temperature, pH, and affect the plant growth indirectly^7,8^. It could cause several diseases in humans due to its carcinogenic and mutagenic effects^9,10^.

Bioremediation is a one of the effective approaches to the treatment of crude oil contaminations in the environment^11–13^. There are some physical and chemical methods available for the removal of pollutants however, they require expensive equipment and it may produce secondary toxic pollutants which could affects the environment. Bioremediation is an ecologically acceptable and highly sustainable approach compared to other removal techniques and has many advantages such as more efficiency, low cost, and eco-friendliness^14,15^. The conventional bioremediation approach has a few disadvantages, such as a slow process, the unavailability of potential microorganisms, and the need to include external factors to stimulate the degradation process^16,17^. To improve the conventional bioremediation process, integrated approaches have been adapted by using nanomaterials. Several nanomaterials are used in the biodegradation process as adsorbing and desorbing agents to promote microbial growth (co-promotor sources along with other nutrients)^18,19^. Among the used nanomaterials, iron nanoparticles (Magnetic) accelerate higher interest in bioremediation research due to having the largest surface area^20,21^.

Nanoparticles have higher surface areas and are used as catalysts and adsorbing agents to improve various physiochemical reactions^22,23^. In general, nanoparticles have been used currently in different environmental sites to control or reduce the various pollutants from air, water, and soil. Nanoparticles have the ability such as high adsorption capacity thus, they can easily absorb pollutants from water and soil. Among the nanomaterials, iron nanoparticles are proven as one of the most influential and vital reducing and catalytic agents due to their physical, chemical, and biological properties, which increase their availability and impact on the environmental remediation process^24^. The role of iron nanoparticles in the remediation mechanism is to act as an electron donor during the oxidation and reduction process and donate the electron to the pollutants by contacting it in the medium resulting in the contaminant becoming more stable and converted or breakdown by less toxic/mobile form^25,26^. According to several reports, many plants have been used for the synthesis of iron nanoparticles however *Lawsonia inermis* is a potential plant and the presence of lawson (dye) is a major active constituent and has higher reducing properties which reduce the ferrous sulfate into metal nanoparticles^27^.

*Pseudomonas aeruginosa* is a ubiquitous in nature and one of the efficient oil-degrading bacteria reported by several kinds of literature^28^. *Pseudomonas* species have effectively degraded petroleum hydrocarbons by producing surface-active biomolecules called biosurfactants^29,30^. Biosurfactants have been used more than other synthetic surfactants due to having many advantages such as less toxicity, better-foaming properties, eco-friendly and easy biodegradability, and contain both hydrophobic and hydrophilic moieties with capability to reduce the surface tension of the solution^31–33^. Several treatment methods have been implemented to eliminate the contaminants including chemical oxidation, physical methods, and bioremediation. Among all the approaches, bioremediation is an eco-friendly method. However, it is not effective to remove the mixed contaminants such as PAHs, heavy metals, and some other hydrophobic compounds due to their lower solubility. Recently iron nanoparticles have been used in the bioremediation process where they could easily adhere to the contaminants whereas biosurfactants also increase the bioavailability of the hydrophobic contaminants by solubilizing them in different environments. To overcome these issues, combining both biosurfactants and nanoparticles could enhance the removal of hydrophobic pollutants in the bioremediation approach. There are very few studies only done by the combination of biosurfactant and nanoparticle with bacterial strains for the removal of contaminants. In this study, green synthesized iron nanoparticle was applied to the degradation of crude oil with the bacteria and biosurfactant (BS) and evaluated the biodegradation efficiency (BE).

## Results and discussion

### Synthesis and characterization of G-FeNPs nanoparticles

**Figure 1:**
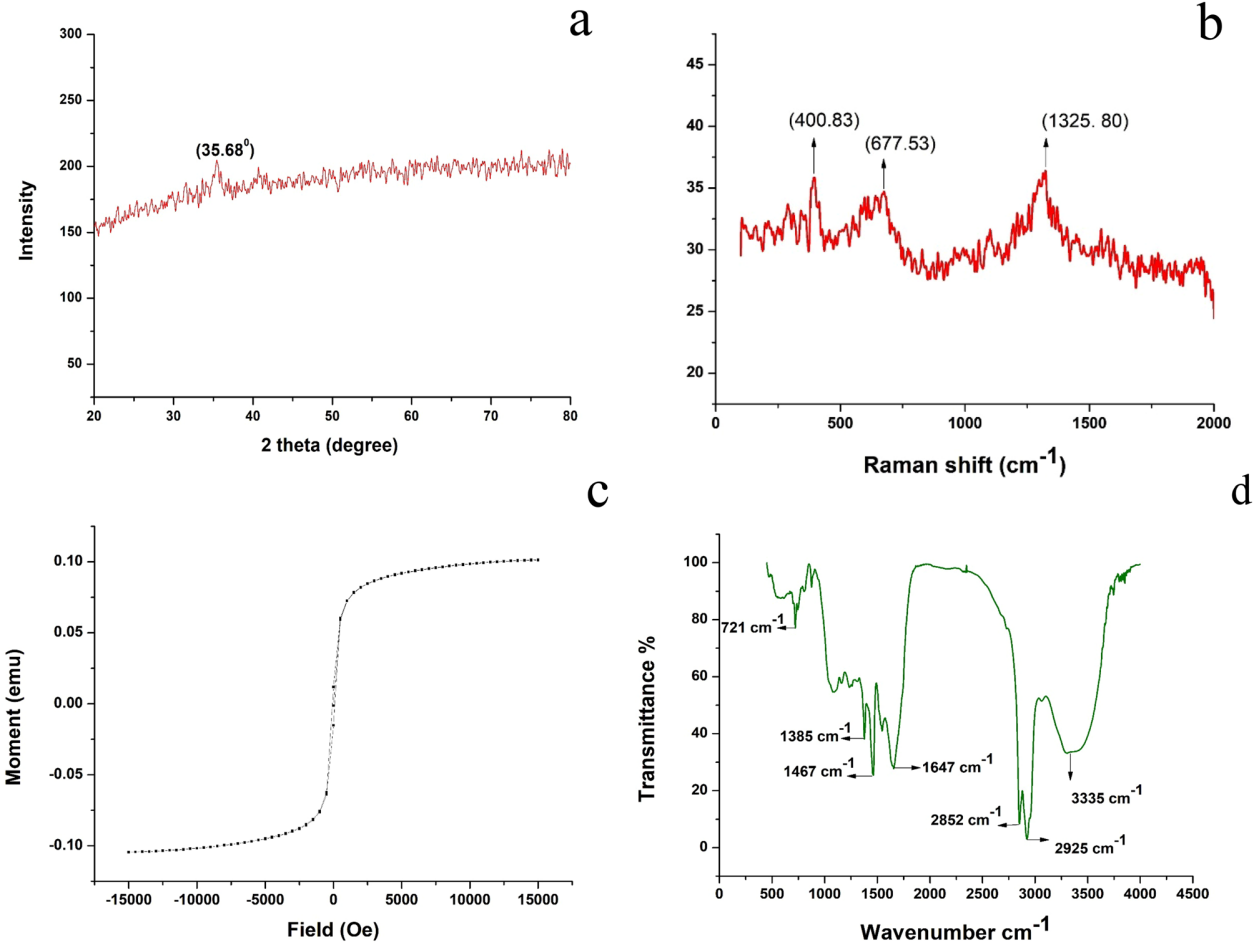
(**a**) X-ray Diffraction analysis of G-FeNPs, (**b**) Raman spectra of G-FeNPs, (**c**) VSM spectrum of G-FeNPs, (**d**) FTIR spectra of G-FeNPs.

The synthesis of iron nanoparticles was confirmed by using XRD analysis. The XRD analysis strongly confirms the formation of iron oxide nanoparticles. XRD analysis was used to determine the crystalline structure of the synthesized iron nanoparticle, and the result was shown in Fig. 1a. The characteristics peak of synthesized G-FeNPs was observed around 2θ of 35.68º. The observed diffraction peaks denote the crystalline phase structure of the synthesized iron nanoparticle (JCPDS No-01-1030). Dana et al.^34^ reported that a similar value for the green synthesized iron nanoparticles, which confirming the presence of iron nanoparticles crystalline in nature. Micro-Raman spectroscopy analysis is a non-destructive and rapid technique which applied extensively to determine and investigate the carbon materials present in the sample. The Raman spectra results are shown in Fig. 1b. From this spectrum, different peaks have appeared around 1325 cm^−1^, 677 cm^−1^, and 400 cm^−1^, respectively. This result confirmed the presence of Fe_3_O_4_ nanoparticles. A similar Raman spectra result was obtained for Fe_3_O_4_ nanoparticles by Lee et al.^35^. The magnetic properties of the synthesized G-FeNPs were confirmed by using a vibrating sample magnetometer as illustrated in Fig. 1c. From this figure, a superparamagnetic behaviour with a magnetization of 0.102 emu with a coercivity value of 91.001Oe was confirmed. This indicates that obtained iron nanoparticles had effective magnetic properties. The various functional groups present in the synthesized G-FeNPs were identified by using the FTIR spectrum. The range of the wavenumber was measured as 400–4000 cm^−1^ with a resolution of 4 cm^−1^. The absorption peak of G-FeNPs by FTIR spectra is presented in Fig. 1d. From this spectrum, FTIR results showed the presence of major peaks at 1385, 1467, 1647, 2852, and 2925 cm^−1^, respectively. The peak between 1385 and 1467 cm^−1^ confirms the presence of the alkane group (–CH_3_). The peak shown at 1647 cm^−1^ indicates the presence of amide -I in the synthesized iron nanoparticles. The peaks between 2852 and 2925 cm^−1^ suggest the presence of CH stretch. The absorption peak at 3335 cm^−1^ reveals the existence of the hydroxyl (OH) group.

**Figure 2:**
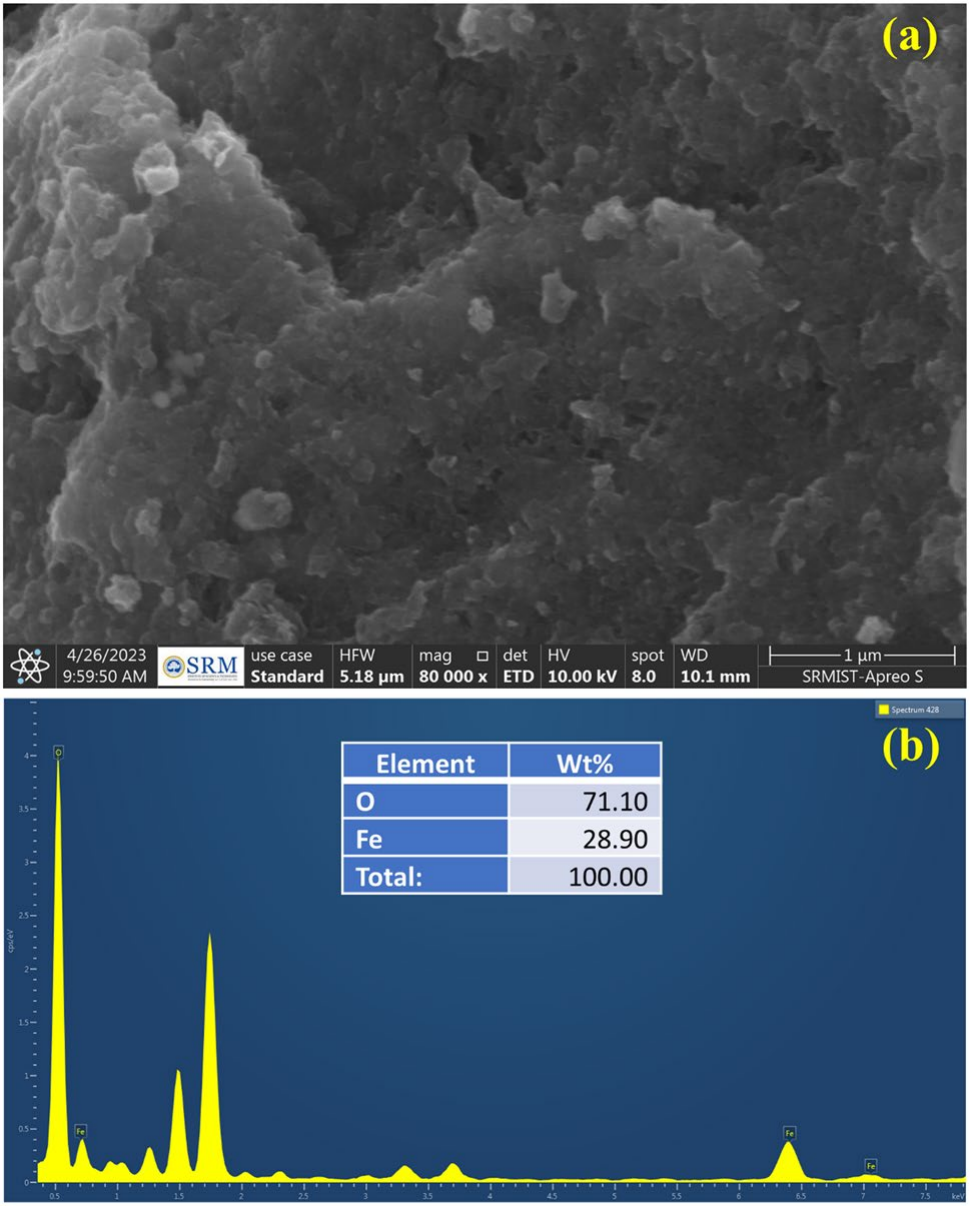
(**a**) FESEM profile of G-FeNPs, (**b**) EDAX spectrum of G-FeNPs.

Further surface analysis was carried out with FESEM, and the obtained micrographs were shown in Fig. 2a. As shown in Fig. 2a, the irregular shape of the iron nanoparticle was observed due to the agglomeration nature. A similar irregular shape was obtained for the synthesized Fe_3_O_4_ by Takai et al.^36^.This might be due to their magnetic nature particles always tended to attract each other in the solution. The EDAX image was obtained and confirmed the presence of Fe by indicating the appearance of the Fe, and O elements, as illustrated in Fig. 2b. The EDAX confirmed that obtained iron oxide nanoparticles contain significant portions of iron and its oxide without any other impurities. Carbon peaks indicate that obtained iron oxide nanoparticles have a trace amount of plant extract over their surface.

**Figure 3:**
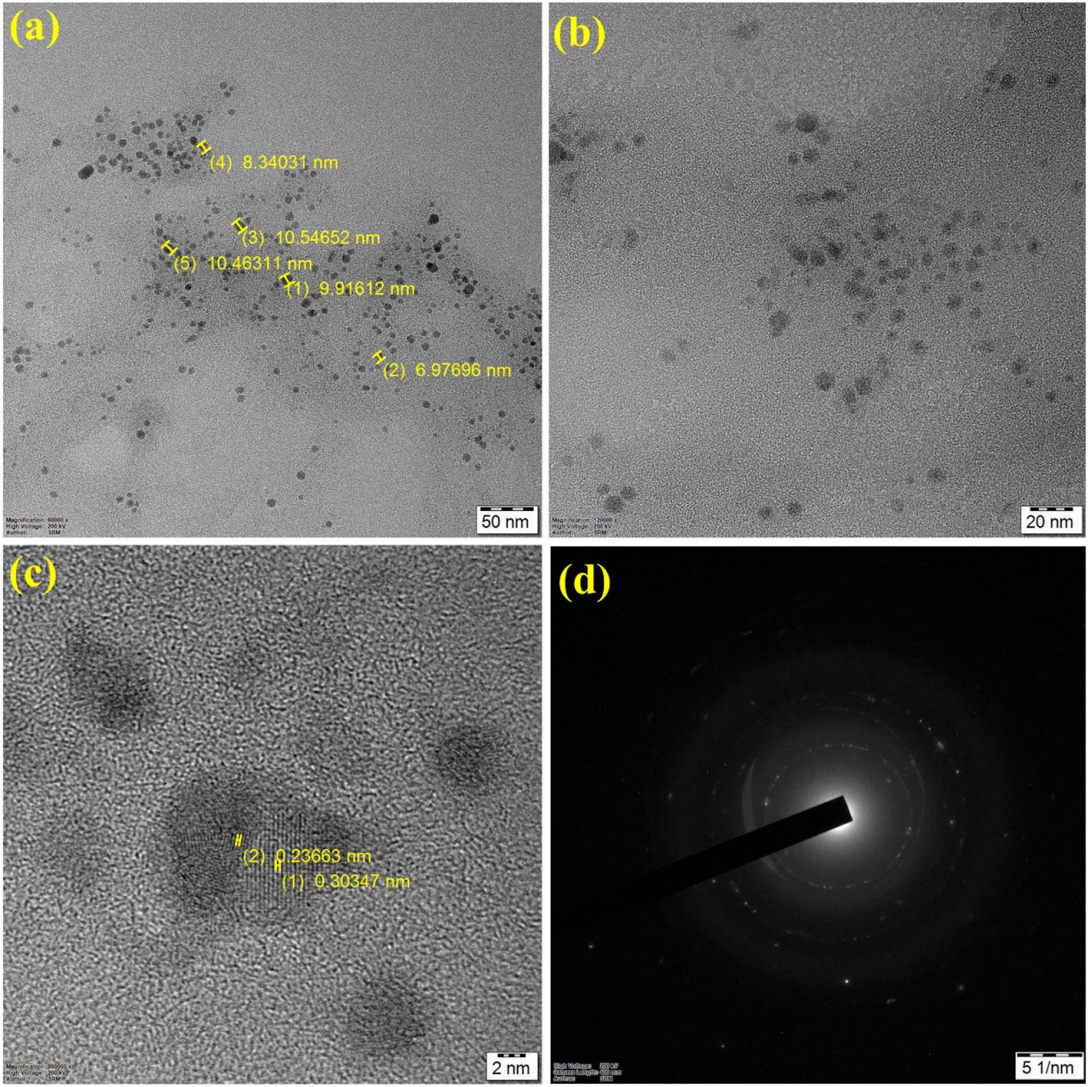
TEM analysis of synthesized G-FeNPs: (**a**) and (**b**) Different magnifications (50 nm and 20 nm) (**c**) high-resolution image of G-FeNPs and (**d**) SAED pattern of G-FeNPs.

The HRTEM analysis was used to investigate the surface morphology of the synthesized iron nanoparticles. The HRTEM images of the synthesized iron nanoparticles are shown in Fig. 3a–d. The average size of the single particle was found to be around 10 nm, as shown in Fig. 3a. and confirmed the spherical-like structure. A similar average size of iron nanoparticles was obtained^37^. Kumar et al.^38^ also reported that the size of the synthesized iron nanoparticle from *Terminalia chebula* extract was found to be around 80 nm and determined to be less than 100 nm. Figure 3c provides the HRTEM images of the iron nanoparticles with d-space values of 0.23–0.30 nm which strongly confirms the presence of (311) plane of iron nanoparticles with clear lattice. The selected area electron diffraction (SAED) pattern (Fig. 3d) strongly confirms the interplanar spacing result with a clear diffraction pattern of iron oxide nanoparticles with a clear crystal lattice.

### Gas chromatography and mass spectrometry analysis of crude oil

**Figure 4:**
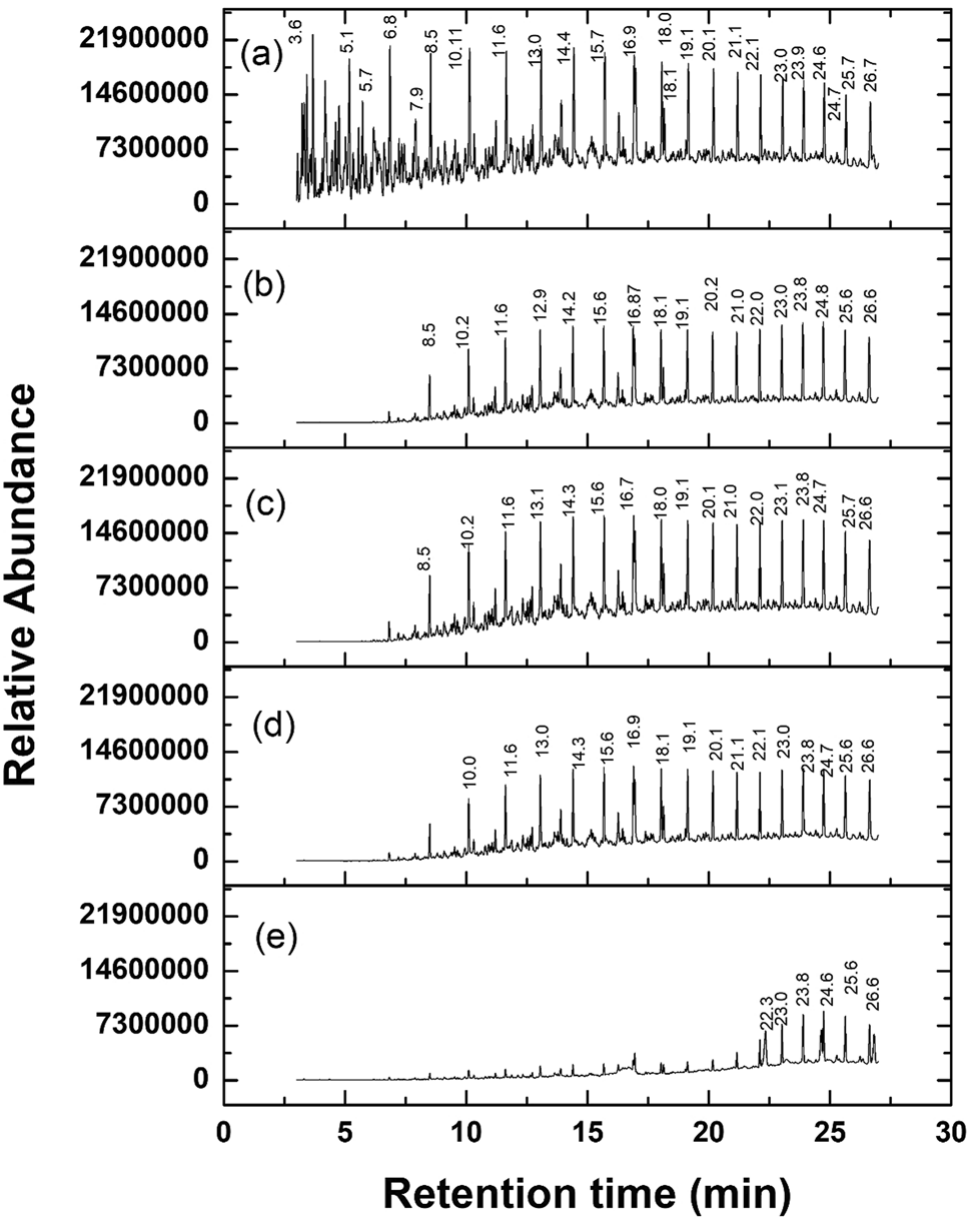
Mass chromatogram profiles of residual crude oil. (**a**) Abiotic control, (**b**) Crude oil+PP4, (**c**) Crude oil+PP4+Biosurfactant, (**d**) Crude oil+PP4+G-FeNPs, (**e**) Crude oil+PP4+Biosurfactant+G-FeNPs.

The residual crude oil from all the flasks was withdrawn after 20 days of incubation and characterized using GCMS analysis to evaluate the biodegradation efficiency. The mass spectrum images of all systems were presented in Fig. 4. From the GCMS results, the biodegradation efficiency of crude oil with PP4 (System -II) was obtained about 35%, and the crude oil+PP4+BS (system III) was found to be 43%. At the same time, the biodegradation efficiency of system IV (Crude oil+PP4+G-FeNPs) was found to be 51%. The highest biodegradation efficiency percentage was obtained for system V (Crude oil+PP4+BS+G-FeNPs) at 82%. The above-mentioned mass spectra results demonstrate the utilization of crude oil containing hydrocarbons during biodegradation. When compared to all biodegradation systems, the degradation rate was efficiently increased in degradation system IV. It could be possible due to the rapid growth of bacterial cells in the aqueous medium and utilized the hydrocarbons as carbon and energy sources. Whereas having effective adherence ability of the added biosurfactant to the hydrophobic hydrocarbons in resultant increased bioavailability of the compounds. Moreover, the addition of iron nanoparticles also increased the biodegradation rate by oxidation and reduction process, resulting in it reducing the pollutants by the hydrocarbons rapid adsorption due to having the highest surface area^39^. Interestingly, *P. aeruginosa* PP4, rhamnolipid biosurfactant, and G-FeNPs effectively contributed to and degraded the hydrocarbons in the short period.

### Fourier transform infrared spectroscopy (FTIR) analysis

**Figure 5:**
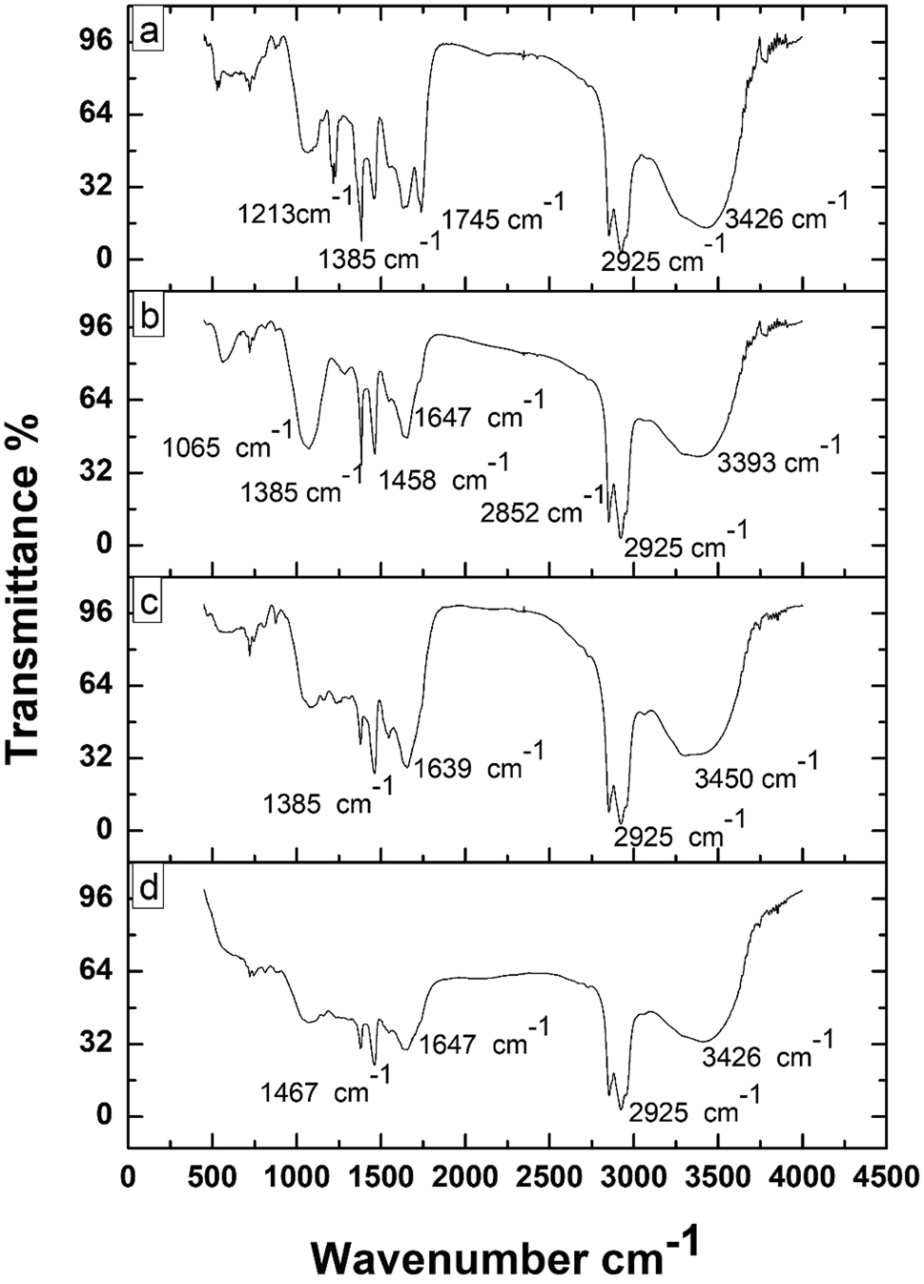
FTIR spectra of residual crude oil. (**a**) crude oil+PP4, (**b**) Crude oil+PP4+Biosurfactant, (**c**) crude oil+PP4+G-FeNPs, (**d**) Crude oil+PP4+Biosurfactant+G-FeNps.

The residual crude oil was collected from all system flasks and characterized using FTIR analysis to identify the functional groups present in the residual content. The FTIR spectra results are shown in Fig. 5a–d. From the Fig. 5, some similar absorption peaks were observed. As shown in Fig. 5a identified major peaks were found around 1213, 1385, 1745, 2925, and 3426 cm^−1^, respectively. The peaks at 1213 cm^−1^ represent a hydroxy ester bond (C–O). The peak at 1385 cm^−1^ confirms the presence of the alkane group (–CH_3_). The peak vibration at 1745 cm^−1^ indicates the presence of the carbonyl (C=O) group. The peaks between 2852 and 2925 cm^−1^ denote the presence of CH stretch. Further, the peak was around 3426 cm^−1^, the hydroxyl group (OH). As shown in Fig. 5b, the identified major peaks are 1065, 1385, 1458, and 2925 cm^−1^ respectively; the identified peaks around 1065 cm^−1^ represent hydroxy ester bond (C–O). The peak at 1385 cm^−1^ confirms the presence of the alkane group (–CH_3_). The absorption peaks at 1458 cm^−1^ confirm the presence of the alkane group (–CH_3_). The peak at 2925 cm^−1^ denotes the presence of CH stretch. As shown in Fig. 5c, the peaks at 1385 cm^−1^ indicate the presence of an alkane group (–CH_3_). The peaks around 1639 cm^−1^ suggest the presence of amide -I in the residual. The peak at 2925 cm^−1^ represents the presence of CH stretch. The peaks around 3450 cm^−1^ denote hydroxyl group (OH) presence. As shown in Fig. 5d, the observed peaks at 1467 cm^−1^ confirm the alkane group (–CH_3_) presence. The peak at 2925 cm^−1^ confirms the presence of CH stretch. The peaks around 3426 cm^−1^ indicate the hydroxyl group (OH) presence. From Fig. 5a–d, some similar peaks were observed in all degradation systems (I–IV). Compared to systems II–IV, the low absorbance peaks were observed in system V, indicating the occurrence of degradation.

### Possible mechanism of crude oil biodegradation

#### Effect of *Pseudomonas aeruginosa*

The hydrocarbons existing in the crude oil were effectively utilized by bacteria which was confirmed by the mass spectrum analysis. The mass spectrum result demonstrated that the strain PP4 could efficiently degrade the hydrocarbons by using them as a sole carbon source for their metabolism and growth in the aqueous medium. In this study, many degraded end by-products were identified by the mass spectrum. For instance, the presence of many acid contents was observed by mass spectrum. They are malonic acid, oxalic acid, and sulfurous acid in the biodegradation system II and dichloroacetic acid, diflurobenzoic acid, fumaric acid in system III, and carbonic acid, and butyric acid in system IV and methoxy acetic acid, pentafluoro propionic acid and decanoic acid in the system V. These acids are very small end by-products that bacteria can efficiently utilize^40,41^.

**Figure 6:**
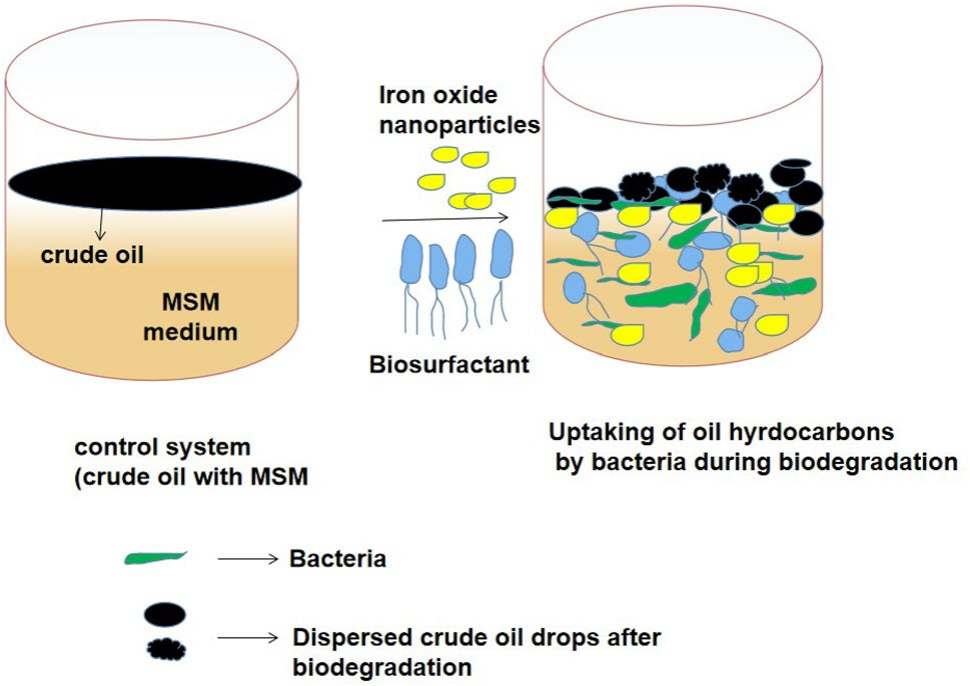
The overview of crude oil degradation.

Many compounds were degraded entirely in the biodegradation system, particularly in system V, shown in Fig. 4. As shown in Fig. 4, when comparing the control peaks with the mixed system (V), most of the compounds were completely degraded from the retention time 0 to 21. These results showed the utilization of low molecular weight hydrocarbon (C_1_–C_22_) and intermediate molecular weight compounds (C_23_–C_39_) by the presence of bacteria, rhamnolipid biosurfactant, and iron nanoparticles. The remaining few peaks may denote the higher molecular weight compounds in the crude oil. Still, some of the higher molecular weight compounds were also significantly utilized by the PP4 strain in the presence of biosurfactants and iron oxide nanoparticles, and the identified major compounds in each degradation systems were shown in Table 1. This result indicates that *P. aeruginosa* PP4 can effectively contribute to crude oil biodegradation with biosurfactant and iron nanoparticles. The overview of the biodegradation of crude oil degradation was shown in Fig. 6. Alkane hydroxylase is the responsible enzyme for the hydroxylation of alkane compounds in the *P. aeruginosa* PP4. Many literatures suggests that several genes are responsible for the alkane hydroxylase production in the multiple bacterial species among them, the strains of *Pseudomonas* are well known and utilize the aliphatic compounds (C_14_–C_32_) as the carbon source and degrade it easily in the aqueous medium by the produced enzyme alkane hydroxylase^42^. Similarly, alcohol dehydrogenase and naphthalene dioxygenase are also responsible for the initial attack of aromatic and polyaromatic hydrocarbon degradation. Alcohol dehydrogenase is further oxidizing the end substrates into fatty acid compounds^43^.

**Table 1:** Percentage of biodegradation of hydrocarbon in presence of crude oil.

Compounds	R. Time	BE %			
		S II	S III	S IV	S V
OCTANE	3.6	100	99.9	99.9	99.98
NONANE	5.1	99.7	99.9	99.9	99.8
Cyclohexane, propyl-	5.7	99.2	99.7	99.7	99.7
Undecane	6.8	87	92.7	94.7	98.2
NAPHTHALENE, DECAHYDRO-, TRANS-	7.9	81	98.9	91.4	97.3
Undecane	8.5	55	67.6	74.9	95.5
Dodecane	10.1	36.3	51.3	59.2	93.7
TRIDECANE	11.6	25.6	42.5	48.9	93.1
TETRADECANE	13.0	20.3	37.9	43.3	91.46
2,6,10-Trimethyltridecane	13.9	21.6	44.8	49.2	89.8
Pentadecane	14.4	17.4	35.9	39.8	91.1
HEXADECANE	15.7	99.8	33.6	36.3	98.4
Heptadecane	16.9	12.1	32.4	34	88.2
HEXADECANE, 2,6,10,14-TETRAMETHYL	16.9	15.5	35.8	38.2	81
Octadecane	18.0	10.5	31.7	32.8	90.6
HEXADECANE, 2,6,10,14-TETRAMETHYL	18.1	100	100	41.6	100
Heneicosane	19.1	10.5	30.7	32.2	90.4
Heneicosane	20.1	8.3	29.4	30.6	89
Heneicosane	21.1	6.7	27.2	29.9	82
Heneicosane	22.1	3.2	22.7	27.7	82.9
Heneicosane	23.0	2.2	15.8	23.7	45.1
HEXACOSANE	23.9	6.5	11.1	19.5	54.8
Docosyl octyl ether	24.6	100	100	100	84
HEXACOSANE	24.7	75.3	7	17.7	38.5
HEXACOSANE	25.6	11	2.9	91.3	36.1
HEXADECANE, 2,6,10,14-TETRAMETHYL	26.6	1.1	2.8	11.2	42.4
HEXATRIACONTANE	26.7	100	100	100	97.1
Total biodegradation efficiency (%)		35	43	51	82

*BE* Biodegradation efficiency (%); *R. time* Retention time.

SII- Crude oil+PP4; SIII- Crude oil+PP4+Biosurfactant; SIV- Crude oil+PP4+G-FeNPs; SV- Crude oil+PP4+Biosurfactant+G-FeNPs.

#### Effect of crude biosurfactant

Microorganisms usually produce potent emulsifiers called biosurfactants, and these biosurfactants help bacteria to degrade the hydrocarbons by reducing the interface between the two different phases. *Pseudomonas aeruginosa* was an efficient oil-degrading bacteria reported by many literatures. In general, *Pseudomonas* species effectively degrade the hydrocarbons by producing some enzymes and surface-active compounds called biosurfactants, and it is one of the bacteria that produce high-yield biosurfactants^44^. Rhamnolipid is an efficient biosurfactant that easily solubilizes the hydrocarbons and changes the hydrophobicity level of the bacteria, enhancing the biodegradation rate^45^. In this study, rhamnolipid biosurfactant obtained a biodegradation efficiency of about 43% in system III. This result demonstrates that the rhamnolipid biosurfactant influences the biodegradation rate by solubilizing the hydrocarbon pollutants.

#### Impact of iron nanoparticle

Based on the impact of FeNPs, which plays a significant role in the biodegradation process. Iron nanoparticles reported by earlier studies mainly influenced two possible mechanisms. As per the first one, it efficiently adsorbs the higher molecular weight hydrocarbons due to its large surface areas^46^. The second one is that the iron nanoparticles effectively attract the adhesion of bacterial cells by their surface charge and increase the contact of the bacteria and hydrophobic hydrocarbons indirectly, increasing the availability and utilization of the hydrocarbons. In the second mechanism, the bacterial growth was influenced indirectly by the involvement of other produced enzymes and surface-active biomolecules by bacteria in the aqueous medium^47^.

In general, iron plays an important role in the environmental recycling of major pollutants. Iron particles can increase their surface area due to having unique magnetic properties. This could be useful to adsorb and clean-up of the environmental contaminants^48^. Moreover, in the environment, iron plays a significant role as a contaminant mobility and reduce/breakdown the compounds due to acting as an electron donor^49^. Recently, the usage of iron nanoparticles has been interestingly increased in the bioremediation process owing to their many physio/chemical and biological properties.

Iron nanoparticles could effectively degrade organic pollutants like PAHs and other hydrophobic hydrocarbons^50,51^. It is one of the potential nanomaterials used for various environmental cleanup due to enhancing the microbial mechanisms by easy cell attachment and entrapment and helps microbes to degrade toxic contaminants^52,53^. Moreover, the Fe_3_O_4_ nanoparticle has higher adsorption capacity, super Para magnetism, and chemical inertness. It’s also responsible for cell permeability and can induce the degrading enzymes and their activities reported by Wang et al.^54^. In addition, some secondary metabolites and other organic components might be good nutrient and carbon sources for bacterial growth, and their activities in the aqueous medium increased the biodegradation process^55,56^. Recently, iron nanoparticles have been used as a growth-promoting source in many environmental applications like oil degradation.

## Methods

### Chemicals

Chemicals such as ferrous sulphate (FeSO_4_), Bushnell haas (BH) broth, and LB broth were purchased from Himedia (Mumbai, India). The solvents used for extraction, such as n-hexane and ethyl acetate, were obtained from Merck, India. All the received chemicals were used as received. The crude oil sample used in this study was obtained from the Oil and Natural Gas Corporation of India (ONGC)^57^.

### Bacterial culture

*Pseudomonas aeruginosa* PP4 was used in this study which was previously isolated from oil-contaminated soil as described earlier^58^. The stored bacterial culture was retrieved and sub-cultured using LB broth, and freshly prepared culture was used for further studies.

### Synthesis and characterization of iron nanoparticles

#### Collection of plant leaves

The fresh henna plant leaves (*Lawsonia inermis*) were collected from the Thiruvalluvar University Garden with proper approval from university authorities. The collected leaves were identified and authenticated (PARC/2019/3966) as *Lawsonia inermis* by Dr. Jayaraman, and the voucher was stored at the Herbarium in Plant Anatomy Research Centre, Tambaram, Chennai. Plant materials collection and process were carried out as per World Health Organization (WHO)—Geneva guidelines^59^. The collected leaves were washed with tap water, followed by distilled water for three times to remove the dust and waste debris. Then, the washed leaves were dried under sunlight for a week. After that, dried leaves were crushed using a mortar and pestle to get the powder. Further, the powder was sieved to obtain uniform-sized fine particles. The obtained powder (5 g) was added with 50 mL of deionized water, and the mixture was continuously stirred by a magnetic stirrer for 3 h, resulting in the solution being kept for 1 h to stabilize and filtered using Whatman no.1 filter paper. Finally obtained extract was stored at 4 °C for further use^60^.

#### Preparation of green synthesis of iron nanoparticles (G-FeNPs) using plant extract

For the preparation of G-FeNPs, FeSO_4_ (10 mL of 0.1 M) was used with filtered plant extract. The 2 mL of plant extract was added every five minutes interval with FeSO_4_ solution until it reached 50 mL resulting in the reaction mixture being heated at 70 °C with constant stirring. After that, the solution was allowed to cool down and centrifuged at 10,000 rpm for 2 minutes^61^. After this, the collected product was washed several times and dried for 3 h at 50 °C for 24 h and used for further experiments. The X-ray diffraction (XRD) analysis was used to determine the crystalline nature of the synthesized iron nanoparticles (Rigaku-Ultima IV modal; λ = 0.154 nm). High-Resolution Transmission Electron Microscope (HR-TEM) (JEOL, Japan, JEM-2100 plus) and Field Emission Scanning Electron Microscope (FESEM – Thermo scientific apreo S) were used to identify the size, shape, and surface morphology of the synthesized iron nanoparticle. The carbon materials present in the synthesized iron nanoparticle was determined by Raman Spectroscopy (Renishaw-Via™ Qontor™). The magnetic nature of the iron nanoparticle was observed by Vibrating Sample Magnetometer (VSM) (Lake Shore model-4700) analysis. FTIR analysis was used to confirm the functional compounds present in the synthesized nanoparticles.

#### Production and extraction of biosurfactant

The Bushnell hass (BH) broth was used for the biosurfactant production. The production was carried out in a 500 mL Erlenmeyer flask containing 300 mL of MSM (pH – 7.0), bacterial culture (4.2 × 10^3^ CFU/g), and crude oil (3%) as the sole carbon source. Then the flask was incubated for seven days (150 rpm) at 37 °C under aerobic conditions. The procedure of biosurfactant extraction was followed as described earlier^62,63^. In brief, cell-free supernatant was obtained by centrifugation at 10,000 rpm for 15 min and pH was adjusted to 2.0 for the precipitation. The whole content was stored in a refrigerator overnight at 4 °C. After this, the precipitated biosurfactant was centrifuged at 8000 rpm for 10 min, and the collected supernatant was mixed with an equal volume of ethyl acetate for 2–3 min in a separate funnel and kept for 2 h to allow the phase separation. After that, the collected biosurfactant from the organic phase was used for further studies. The collected crude biosurfactant was characterized and reported earlier^58^. The same rhamnolipid biosurfactant was used for this current study.

#### Biodegradation of crude oil

The biodegradation study was carried out in a 250 mL sterilized conical flask containing 100 mL of MSM broth supplemented by sterile (using 0.2 µm syringe filter) crude oil (2%) under the various systems as follows: (i) crude oil (abiotic control) (ii) crude oil with PP4 (2%); (iii) crude oil+PP4+produced biosurfactant (BS) (10 mg/L); (iv) crude oil+PP4+G-FeNPs (0.15 mg/L); (v) Crude oil+PP4+BS+G-FeNPs. Further, all flasks were incubated for 20 days (150 rpm) at 37 °C under aerobic conditions.

#### Analytical methods

The residual crude oil was withdrawn from all flasks using n-hexane as solvent after the incubation and subjected to GCMS (Agilent, Palo, Alto CA- GC model 6890 and mass selective detector model 5973) analysis to evaluate the biodegradation efficiency. The detailed GCMS procedure was followed as described earlier^40^. To confirm the functional constituents, present in the residual crude oil was characterized by using FTIR spectrum (Perkin Elmer Inc., USA) with potassium bromide. The FTIR protocol was followed as previously described^41^.

## Conclusions

In this study, crude oil containing hydrocarbons was effectively degraded by adding *P. aeruginosa* PP4, producing rhamnolipid biosurfactant and green synthesized iron nanoparticles. HRTEM analysis confirmed the spherical-like structure of the synthesized iron nanoparticle. The XRD analysis confirmed the crystalline nature of the synthesized iron nanoparticle. The Raman spectra showed the presence of Fe_3_O_4_. The functional constituents present in the G-FeNPs were identified by FTIR characterization. The highest biodegradation efficiency about 82% was obtained for the hybrid system (PP4+BS+G-FeNPs) in 20 days incubation period whereas, 35%, 43%, and 51% were obtained for systems (II-IV), respectively. The FTIR analysis confirmed the functional group's presence in the residual crude oil. The above results demonstrated that the hydrocarbons are utilized by bacterium with the help of combining rhamnolipid biosurfactants, and synthesized iron nanoparticles. Finally, it concluded that this nano-bioremediation approach is a promising technology for many environmental applications like oil degradation.

### Supplementary Information

Supplementary Information.

## Data Availability

All data generated or analysed during this study are included in this published article.
