# Supplementary material for: Synthesis and characterization of iron oxide nanoparticles from Lawsonia inermis and its effect on the biodegradation of crude oil hydrocarbon
Source: Sci Rep. 2024 May 17;14:11335. doi: 10.1038/s41598-024-61760-6 (PMC11101646; doi:10.1038/s41598-024-61760-6)
Supplement: Supplementary file 1 — Supplementary Information. [file 41598_2024_61760_MOESM1_ESM.pdf]

Project: 26.04.23
User Name: SRM IST Research Institute

Full scale counts: 413
Integral Counts: 13525

Base(3)

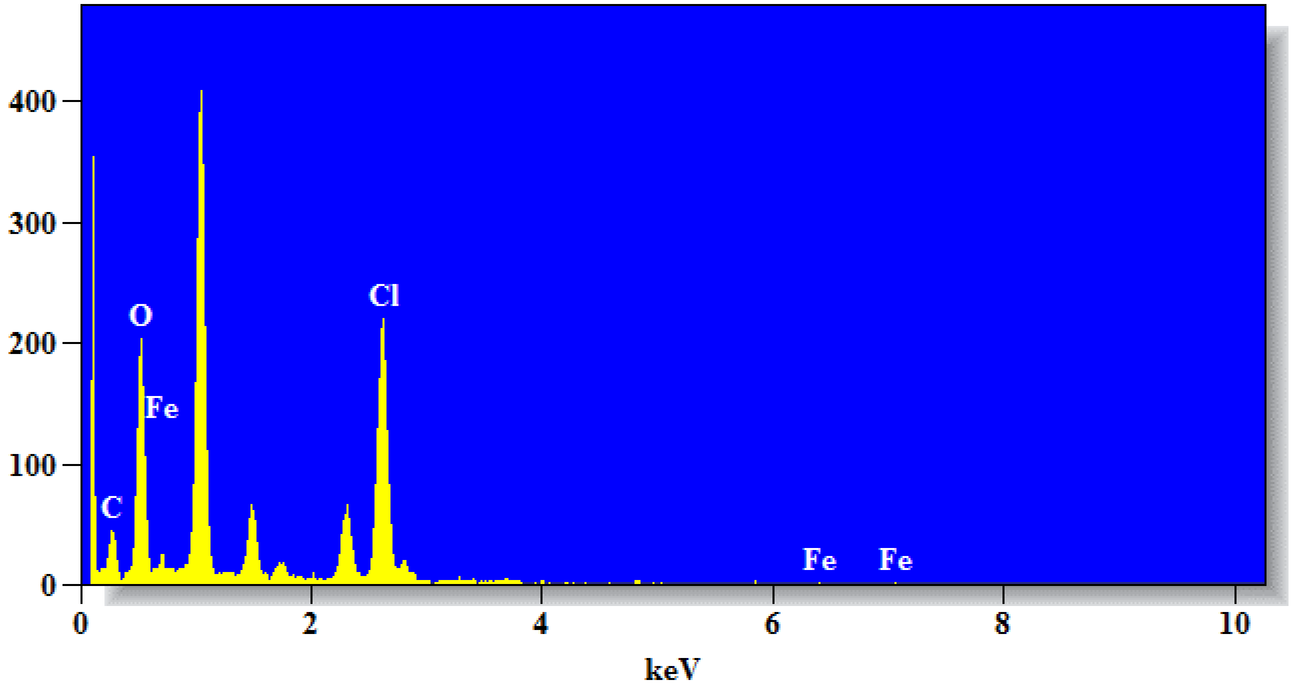

Quantitative Results for: Base(3)

Element	*Net Counts*	*Weight %*	*Atom %*	*Atom % Error*	*Formula*
C	0	0.00	0.00	± 0.00	C
O	1714	31.82	54.91	± 1.63	O
Cl	2923	40.04	31.18	± 0.83	Cl
Fe	520	28.15	13.92	± 1.50	Fe
Total		100.00	100.00		

ROI Results for: Base(3)

Operation	*Gross Counts*	*Net Counts*

Project: 26.04.23
User Name: SRM IST Research Institute

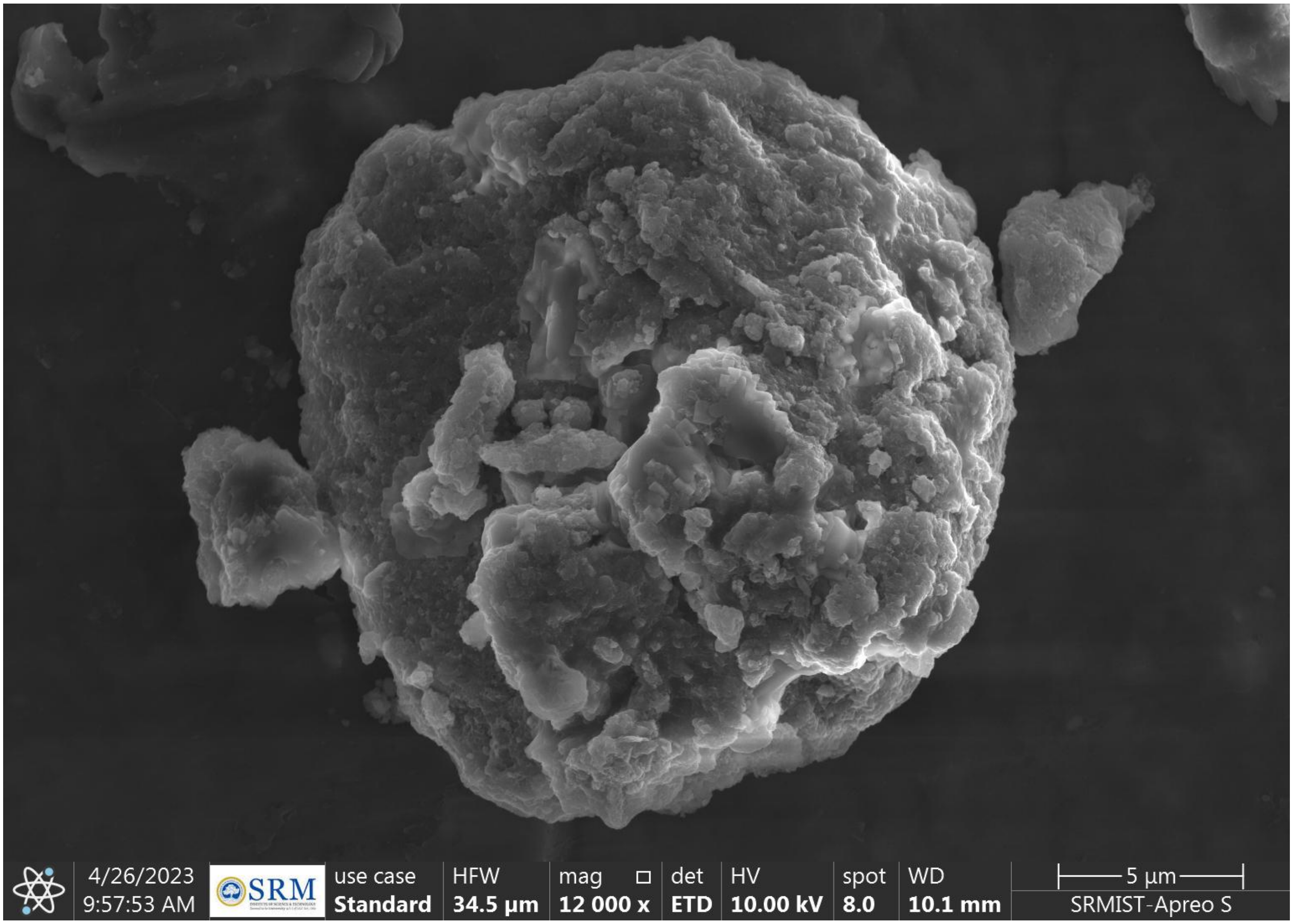

Project: 26.04.23
User Name: SRM IST Research Institute

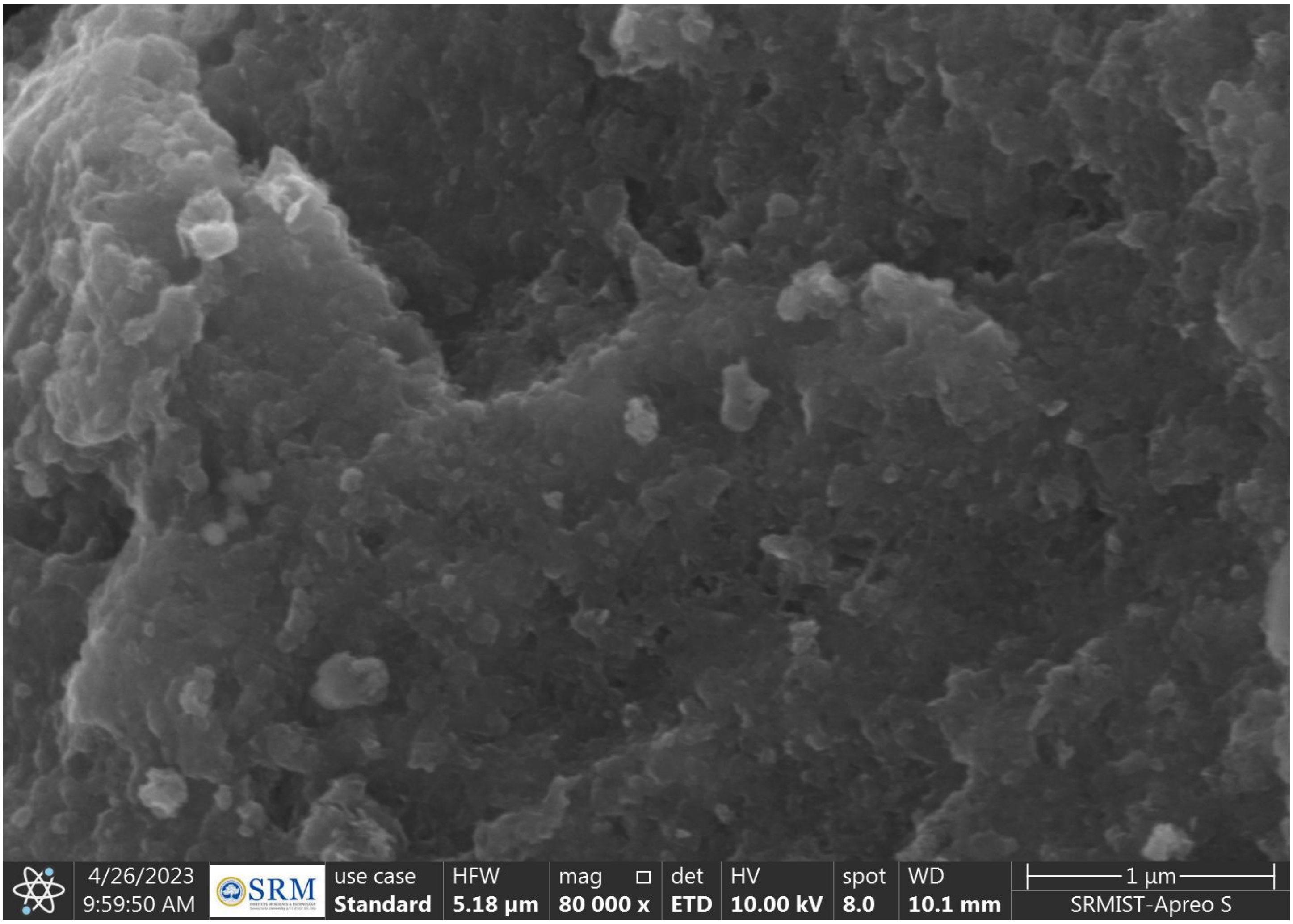

Project: 26.04.23
User Name: SRM IST Research Institute

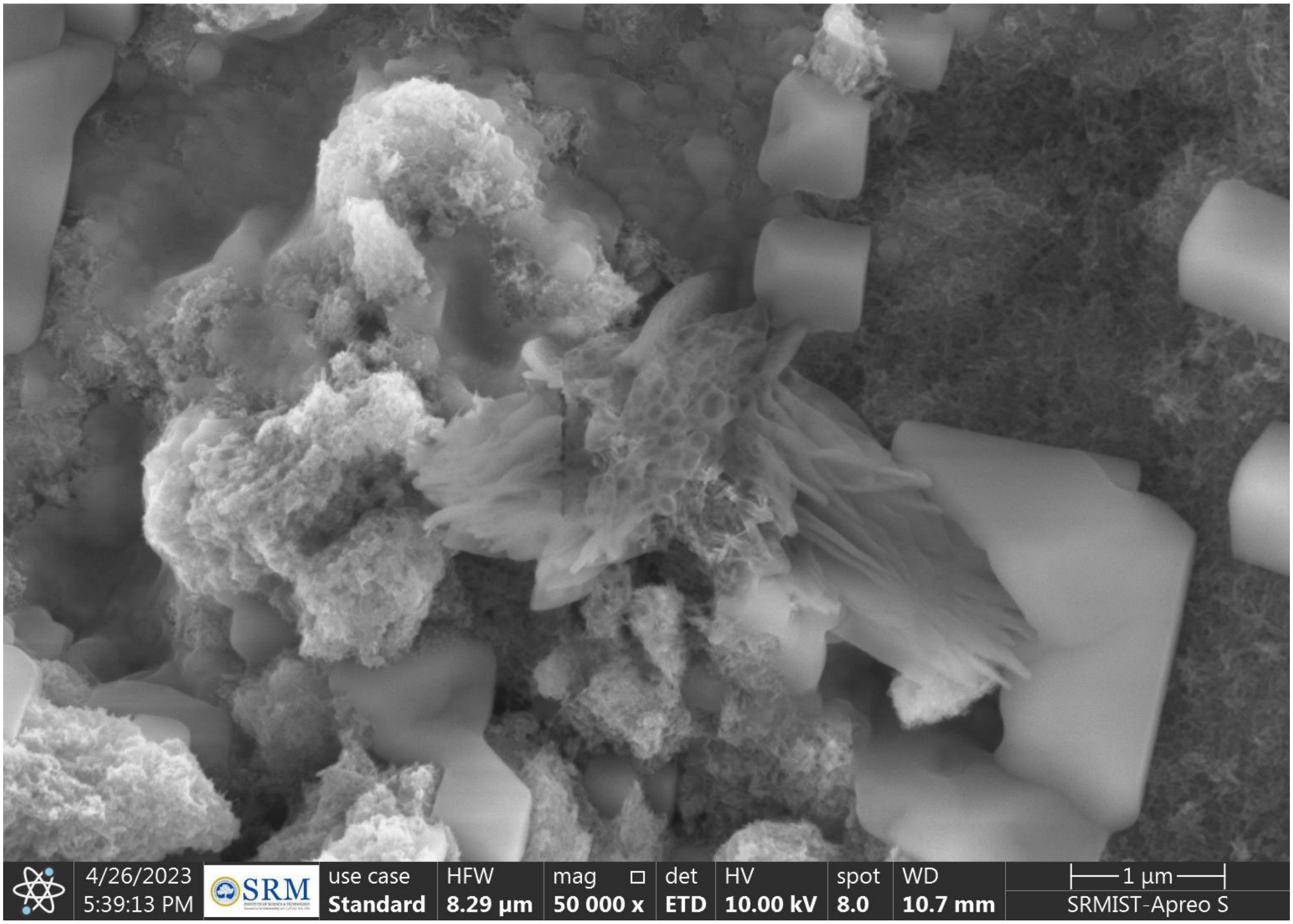

Project: 26.04.23
User Name: SRM IST Research Institute

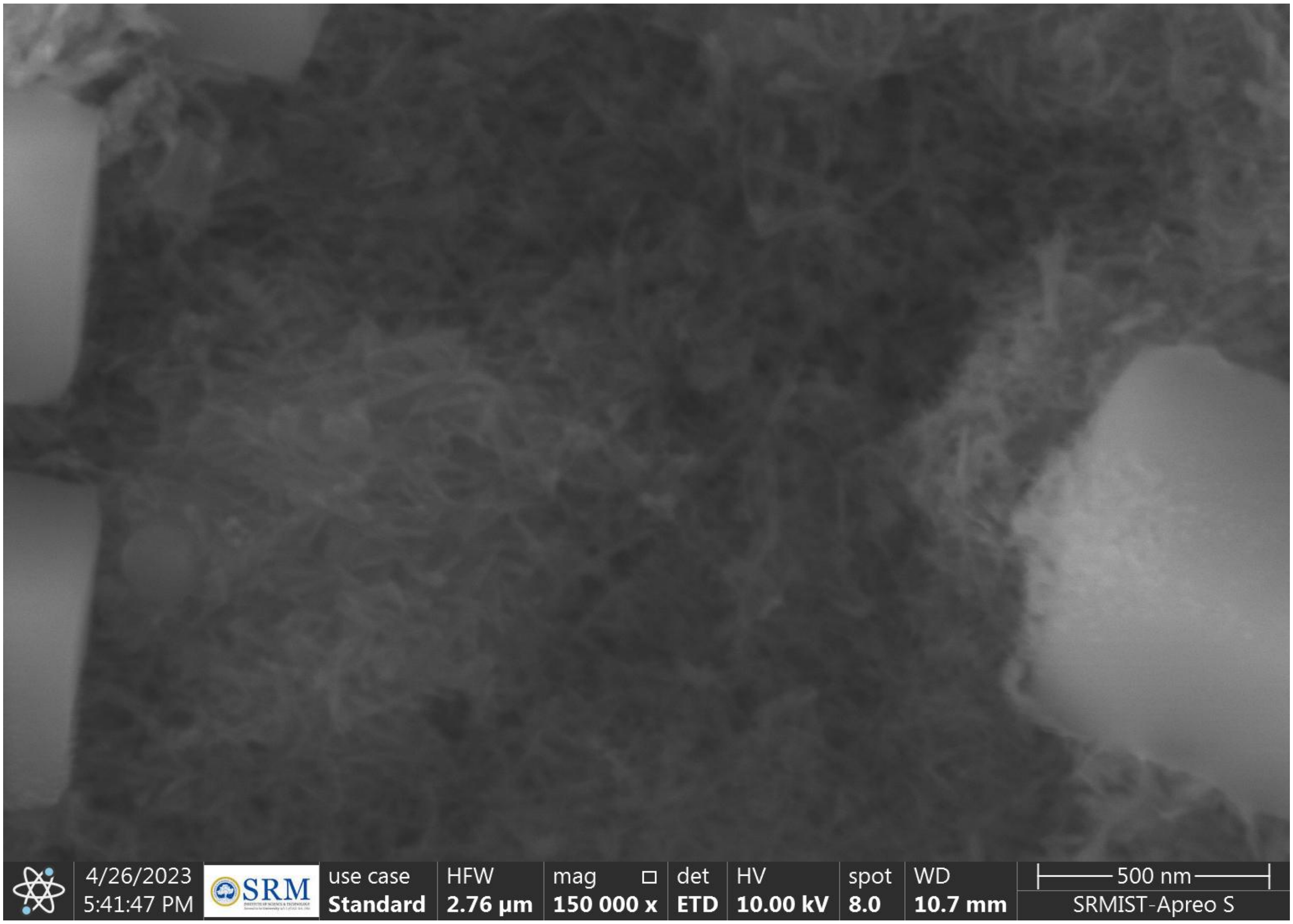

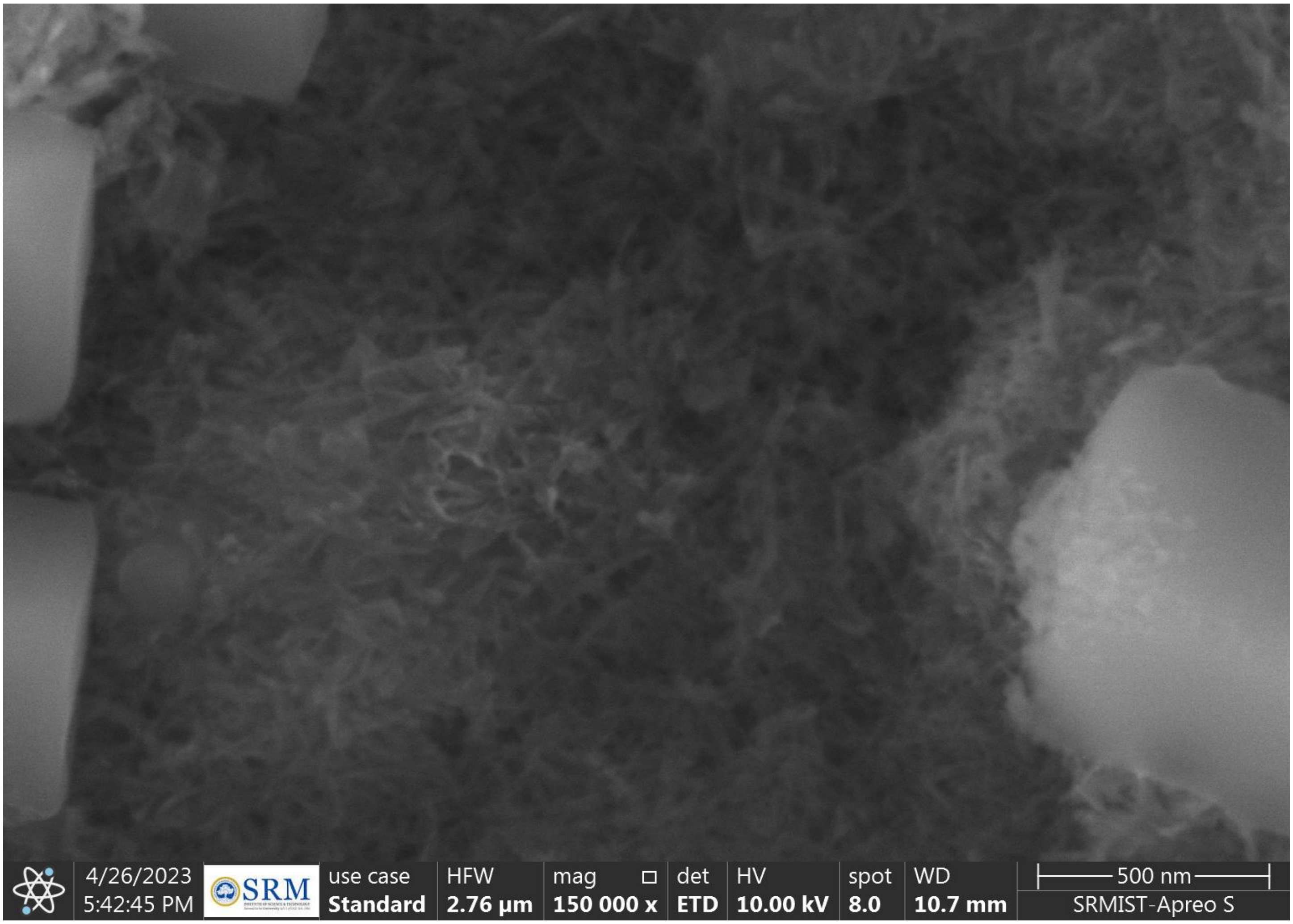
4/26/2023
5:42:45 PM
SRM
use case
Standard
HFW
2.76 µm
mag
150 000 x
det
ETD
HV
10.00 kV
spot
8.0
WD
10.7 mm
500 nm
SRMIST-Apreo S

Project: 26.04.23
User Name: SRM IST Research Institute

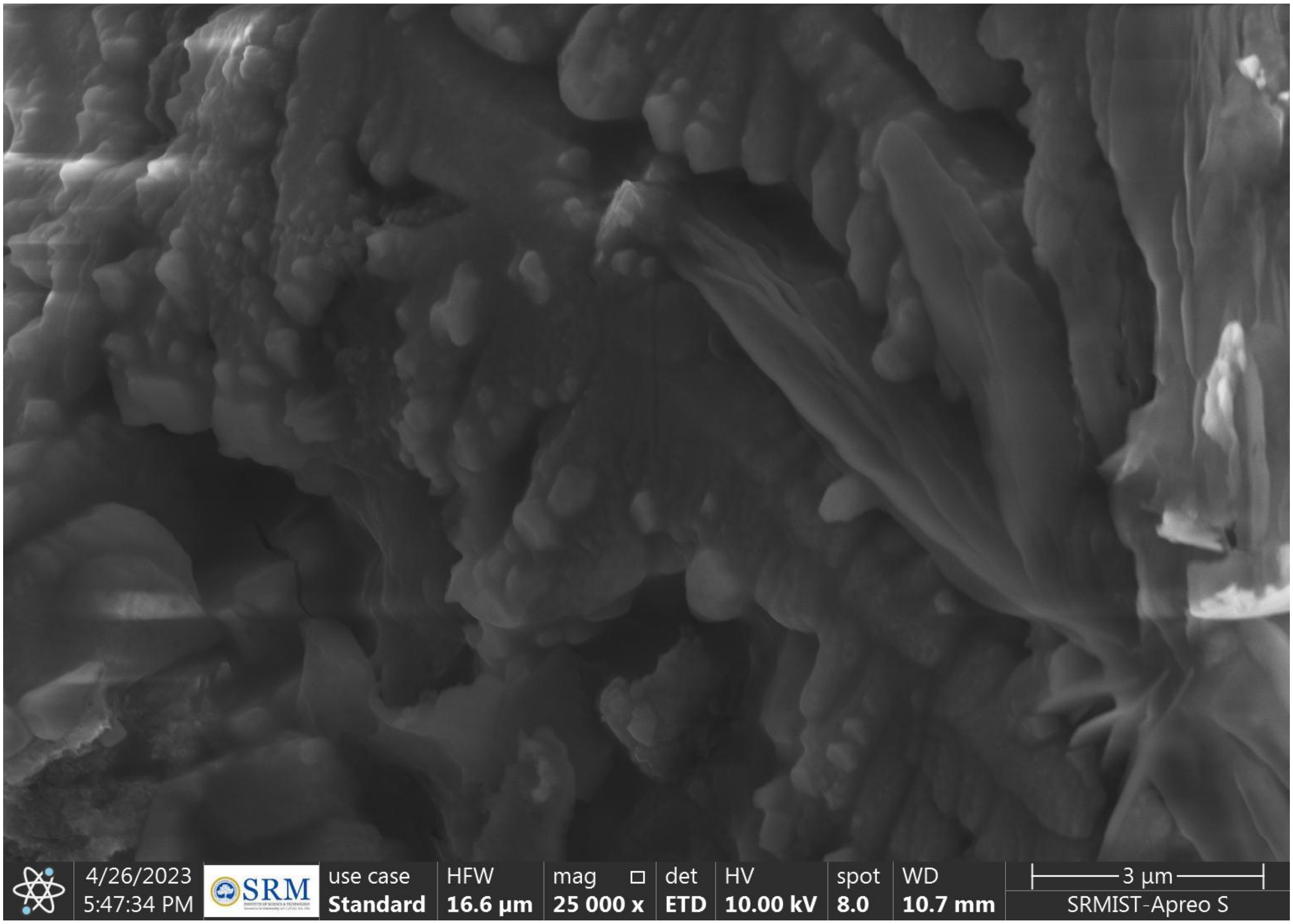

FEB01

100.25	681
100.77	676.817
101.289	674.634
101.809	720.452
102.329	636.269
102.847	684.086
103.366	669.904
103.886	667.721
104.406	688.925
104.925	652.543
105.445	668.81
105.964	668.47
106.484	659.769
107.001	670.904
107.521	654.911
108.04	666.416
108.56	682.465
109.079	680.035
109.598	684.803
110.116	671.599
110.635	671.447
111.154	646.623
111.674	670.282
112.193	665.842
112.71	666.16
113.229	667.777

113.748	665.309
114.267	672.213
114.784	675.898
115.303	680.432
115.822	672.94
116.341	693.586
116.858	675.559
117.377	690.997
117.896	684.748
118.415	680.985
118.931	683.845
119.45	673.803
119.969	689.499
120.486	708.09
121.004	694.055
121.523	692.694
122.042	685.211
122.558	677.072
123.077	681.078
123.596	677.963
124.112	683.404
124.63	669.557
125.149	686.611
125.665	685.827
126.184	677.893
126.702	677.525
127.218	693.157

127.737	672.768
128.253	688.782
128.771	692.617
129.29	689.917
129.806	671.803
130.324	693.912
130.84	676.015
131.358	711.626
131.876	655.994
132.392	682.619
132.91	683.988
133.426	675.353
133.944	669.98
134.46	661.07
134.978	661.581
135.496	680.426
136.012	682.848
136.53	677.632
137.045	675.103
137.563	672.762
138.079	689.267
138.597	673.429
139.112	669.66
139.63	683.65
140.145	669.093
140.663	669.75
141.178	675.662

141.696	678.541
142.211	664.592
142.729	689.705
143.244	677.572
143.76	664.641
144.277	686.689
144.792	684.804
145.31	684.461
145.825	675.971
146.342	673.41
146.858	679.537
147.375	687.463
147.89	675.837
148.405	692.928
148.922	679.723
149.437	693.453
149.954	678.402
150.469	679.091
150.984	674.566
151.501	695.369
152.016	692.94
152.531	668.834
153.048	686.341
153.563	689.335
154.078	691.962
154.594	711.758
155.109	716.205

155.624	695.501
156.141	726.202
156.655	695.195
157.17	702.292
157.687	719.887
158.201	690.921
158.716	694.955
159.232	695.78
159.747	701.879
160.261	698.489
160.776	705.43
161.292	706.558
161.806	709.984
162.321	704.405
162.835	708.791
163.351	703.808
163.866	711.236
164.38	704.94
164.894	718.75
165.41	723.791
165.924	715.121
166.438	712.753
166.953	713.357
167.467	718.466
167.983	709.616
168.497	735.276
169.011	717.01

169.525	719.025
170.038	714.682
170.554	730.054
171.068	731.833
171.582	729.425
172.096	719.546
172.61	712.714
173.123	725.597
173.637	730.98
174.153	730.312
174.666	704.416
175.18	744.321
175.694	731.285
176.207	715.645
176.721	713.502
177.234	697.869
177.748	719.645
178.261	723.334
178.777	727.269
179.29	720.49
179.803	721.044
180.317	711.716
180.83	728.825
181.343	703.381
181.856	714.185
182.37	725.516
182.883	713.847

183.396	728.163
183.909	709.362
184.422	719.736
184.935	720.046
185.448	694.97
185.961	712.53
186.474	700.885
186.987	724.367
187.5	723.259
188.013	719.725
188.526	726.836
189.039	734.101
189.552	688.225
190.064	701.471
190.577	709.171
191.09	719.448
191.603	719.694
192.115	714.008
192.628	697.953
193.138	701.499
193.651	721.902
194.164	723.381
194.676	727.22
195.189	711.846
195.701	722.922
196.214	702.142
196.726	704.827

197.238	740.562
197.749	724.594
198.261	707.471
198.773	713.138
199.286	730.537
199.798	701.027
200.31	726.172
200.822	715.409
201.332	711.913
201.844	717.727
202.357	737.023
202.869	728.451
203.381	705.83
203.893	735.507
204.403	737.877
204.915	747.701
205.427	713.27
205.938	729.034
206.448	730.239
206.96	719.938
207.472	725.539
207.984	744.29
208.493	733.771
209.005	739.447
209.517	726.54
210.029	732.039
210.538	734.649

211.05	742.212
211.561	754.89
212.073	718.945
212.582	740.78
213.094	770.536
213.605	737.66
214.115	753.221
214.626	737.497
215.138	754.671
215.647	733.777
216.158	745.089
216.669	734.615
217.179	750.733
217.69	745.96
218.201	734.623
218.71	750.494
219.221	744.602
219.733	749.811
220.242	766.704
220.753	767.16
221.262	759.893
221.773	768.341
222.284	756.051
222.793	758.227
223.304	759.141
223.812	768.536
224.323	768.102

224.834	754.25
225.343	738.331
225.854	771.052
226.362	751.699
226.873	743.603
227.382	762.331
227.893	762.236
228.401	730.359
228.912	764.21
229.422	751.593
229.931	772.843
230.441	760.518
230.95	764.88
231.46	751.924
231.969	762.511
232.479	755.843
232.987	755.917
233.496	736.772
234.006	757.71
234.514	754.017
235.025	751.924
235.533	746.912
236.043	760.284
236.551	762.263
237.061	734.106
237.57	758.437
238.08	770.283

238.588	750.606
239.096	755.828
239.606	749.087
240.114	737.117
240.624	755.255
241.132	754.382
241.639	738.075
242.149	757.039
242.657	774.366
243.165	763.452
243.675	766.419
244.182	756.346
244.692	755.139
245.2	758.174
245.707	771.121
246.217	765.528
246.725	745.173
247.232	751.553
247.742	763.651
248.249	767.514
248.757	761.537
249.264	745.405
249.774	748.884
250.281	753.907
250.788	758.123
251.298	768.209
251.805	756.418

252.312	762.6
252.82	765.413
253.329	761.106
253.836	746.632
254.343	740.684
254.85	751.468
255.36	764.972
255.867	745.945
256.374	745.063
256.881	748.592
257.388	760.111
257.897	755.519
258.404	756.575
258.911	753.854
259.418	768.162
259.924	755.611
260.433	776.128
260.94	764.07
261.447	769.658
261.954	798.183
262.46	776.465
262.967	767.814
263.476	780.51
263.982	757.147
264.489	733.788
264.996	752.453
265.502	790.209

266.009	757.506
266.515	750.029
267.022	751.193
267.528	753.52
268.036	778.675
268.543	797.58
269.049	778.365
269.555	764.924
270.062	779.172
270.568	809.177
271.074	789.414
271.58	769.336
272.087	794.914
272.593	776.492
273.099	762.008
273.605	789.88
274.111	781.533
274.617	770.28
275.123	790.204
275.629	764.26
276.135	787.459
276.641	786.429
277.147	782.664
277.653	797.045
278.159	807.586
278.665	780.01
279.17	791.986

279.676	806.697
280.182	813.069
280.688	797.403
281.193	776.156
281.699	802.679
282.204	809.022
282.71	798.086
283.216	768.477
283.721	794.946
284.225	758.364
284.73	762.138
285.236	790.572
285.741	815.478
286.246	822.688
286.752	802.907
287.257	795.188
287.762	785.496
288.266	778.785
288.771	799.333
289.276	798.916
289.781	786.429
290.287	790.942
290.792	789.141
291.295	789.633
291.8	817.927
292.305	816.67
292.81	797.456

293.315	809.734
293.82	776.737
294.323	769.171
294.828	811.847
295.333	799.058
295.838	805.373
296.34	765.201
296.845	784.956
297.35	802.719
297.855	797.814
298.358	805.978
298.862	793.003
299.367	778.217
299.872	800.209
300.374	810.107
300.879	808.363
301.383	782.95
301.888	789.274
302.39	806.698
302.895	795.711
303.399	801.658
303.902	792.731
304.406	803.764
304.91	796.498
305.413	788.922
305.917	801.116
306.421	803.687

306.924	793.936
307.428	793.003
307.932	801.456
308.434	817.794
308.938	796.983
309.44	780.568
309.944	788.762
310.449	782.815
310.951	778.527
311.455	785.978
311.957	802.355
312.46	793.972
312.964	796.294
313.466	792.832
313.97	780.661
314.472	798.049
314.976	800.17
315.478	785.467
315.981	781.209
316.483	790.573
316.987	790.098
317.488	798.55
317.992	790.537
318.496	790.271
318.997	810.736
319.501	825.091
320.002	816.997

320.506	802.309
321.007	791.015
321.509	803.04
322.012	805.8
322.513	782.829
323.017	786.362
323.518	793.892
324.021	812.601
324.523	787.638
325.026	790.759
325.527	796.016
326.03	787.159
326.532	801.826
327.033	806.841
327.536	812.743
328.037	806.088
328.54	800.314
329.041	803.73
329.542	812.407
330.045	814.492
330.546	802.298
331.047	799.435
331.55	811.388
332.051	821.878
332.553	824.192
333.054	835.625
333.555	828.291

334.058	820.556
334.558	827.105
335.059	822.913
335.562	805.209
336.062	824.508
336.563	820.002
337.064	826.167
337.566	815.488
338.067	820.607
338.567	814.744
339.07	835.547
339.57	841.29
340.07	836.617
340.571	829.747
341.073	814.248
341.574	815.98
342.074	830.474
342.574	852.715
343.076	846.177
343.577	846.034
344.077	818.391
344.577	848.406
345.077	817.914
345.579	855.302
346.079	835.763
346.579	831.055
347.079	869.839

347.579	833.99
348.081	842.047
348.581	867.303
349.081	835.174
349.581	836.193
350.081	839.091
350.581	877.648
351.081	866.451
351.582	856.488
352.082	850.272
352.582	832.647
353.082	852.916
353.581	874.154
354.081	852.805
354.58	856.56
355.08	888.784
355.58	874.511
356.081	832.972
356.581	878.092
357.08	838.119
357.58	866.826
358.079	877.332
358.578	827.571
359.078	858.891
359.577	822.055
360.076	838.551
360.576	842.791

361.075	863.81
361.574	885.588
362.073	873.337
362.573	854.104
363.072	843.517
363.105	842.791
363.608	836.722
364.109	868.637
364.612	810.689
365.114	852.519
365.617	866.238
366.118	860.126
366.621	828.402
367.124	863.669
367.625	858.119
368.128	875.553
368.629	851.905
369.132	888.925
369.633	841.975
370.136	834.006
370.636	845.165
371.139	860.918
371.64	854.114
372.143	872.094
372.643	827.763
373.146	816.395
373.647	883.576

374.15	846.547
374.65	873.254
375.153	897.009
375.653	863.776
376.154	859.555
376.656	891.305
377.157	861.838
377.659	879.065
378.16	855.181
378.662	857.178
379.162	874.818
379.663	877.241
380.165	879.304
380.665	865.364
381.168	845.412
381.668	846.762
382.168	863.983
382.67	868.81
383.17	840.865
383.671	857.936
384.173	846.161
384.673	890.482
385.173	836.35
385.675	862.975
386.175	854.317
386.675	860.708
387.177	872.35

387.677	833.75
388.176	855.499
388.678	875.43
389.178	875.406
389.678	880.36
390.18	872.397
390.679	858.929
391.179	873.334
391.679	854.032
392.18	881.912
392.68	868.349
393.18	862.461
393.679	864.968
394.181	890.276
394.68	882.5
395.18	863.14
395.679	866.749
396.181	860.118
396.68	868.061
397.179	881.943
397.679	870.361
398.178	868.153
398.679	857.626
399.179	893.276
399.678	876.728
400.177	876.338
400.676	895.127

401.175	901.778
401.677	871.305
402.176	887.643
402.675	873.415
403.174	892.552
403.673	871.673
404.172	862.421
404.671	886.151
405.172	892.71
405.671	892.38
406.169	890.822
406.668	867.012
407.167	877.016
407.666	901.105
408.165	887.309
408.663	852.816
409.162	901.387
409.661	906.516
410.162	896.683
410.66	901.71
411.159	914.307
411.657	907.769
412.156	893.606
412.654	899.544
413.153	877.77
413.651	892.338
414.15	908.801

414.648	899.205
415.147	885.242
415.645	928.431
416.143	917.932
416.642	896.278
417.14	898.638
417.638	875.234
418.136	893.605
418.635	889.297
419.133	873.926
419.631	887.93
420.129	923.91
420.627	912.136
421.123	897.651
421.621	890.907
422.119	904.32
422.617	893.429
423.115	900.018
423.613	896.775
424.111	905.929
424.609	886.622
425.107	886.857
425.605	917.311
426.102	888.394
426.598	915.225
427.096	897.643
427.594	891.355

428.091	871.919
428.589	915.961
429.086	909.54
429.584	904.388
430.08	899.056
430.577	911.554
431.075	897.106
431.572	916.704
432.07	897.687
432.567	876.99
433.062	918.112
433.56	902.078
434.057	913.563
434.555	885.429
435.052	888.323
435.547	902.092
436.044	916.727
436.542	888.074
437.039	902.045
437.534	928.568
438.031	875.153
438.528	893.498
439.025	920.714
439.52	894.914
440.017	884.492
440.514	891.372
441.011	873.877

441.506	885.631
442.003	911.373
442.5	874.173
442.995	917.639
443.492	880.194
443.989	877.363
444.483	897.771
444.98	888.363
445.477	885.836
445.971	886.472
446.468	892.515
446.965	877.77
447.459	880.833
447.956	895.058
448.453	909.878
448.947	900.371
449.444	890.012
449.938	894.307
450.435	889.693
450.931	863.107
451.425	865.58
451.922	875.3
452.416	893.077
452.912	881.499
453.409	889.332
453.903	893.195
454.399	890.832

454.893	887.822
455.39	893.754
455.884	912.333
456.38	906.532
456.874	906.915
457.37	896.028
457.864	885.149
458.36	887.189
458.856	875.633
459.35	879.601
459.846	876.379
460.34	862.091
460.834	876.839
461.33	906.429
461.824	883.782
462.319	890.428
462.813	881.278
463.309	889.076
463.803	897.736
464.298	868.971
464.792	900.017
465.288	888.669
465.781	897.764
466.277	905.781
466.77	901.82
467.264	919.661
467.759	873.823

468.253	895.292
468.748	904.529
469.242	881.431
469.735	890.522
470.231	894.644
470.724	921.039
471.217	897.01
471.713	904.342
472.206	906.075
472.701	897.915
473.194	911.881
473.687	896.115
474.183	915.017
474.676	901.39
475.169	908.27
475.664	917.224
476.157	910.782
476.65	911.96
477.143	897.508
477.638	922.152
478.131	899.474
478.624	927.956
479.119	910.793
479.612	924.846
480.104	924.906
480.597	922.653
481.092	916.908

481.585	925.7
482.078	923.453
482.57	936.31
483.065	926.986
483.558	928.432
484.05	940.851
484.543	931.774
485.035	919.666
485.53	928.438
486.022	932.83
486.515	933.84
487.007	940.805
487.5	935.851
487.994	932.853
488.487	938.962
488.979	932.86
489.471	933.714
489.964	926.825
490.456	952.149
490.95	959.123
491.442	943.12
491.934	926.647
492.427	949.222
492.919	946.109
493.411	948.966
493.903	952.664
494.395	949.838

494.887	954.06
495.381	954.001
495.873	959.083
496.365	950.946
496.857	963.246
497.349	954.721
497.841	940.601
498.333	947.444
498.824	957.488
499.316	957.575
499.808	978.605
500.3	955.546
500.791	950.581
501.283	957.7
501.775	960.452
502.266	944.188
502.758	970.83
503.25	950.6
503.741	953.604
504.233	964.422
504.724	952.825
505.216	965.633
505.707	955.907
506.198	975.357
506.69	961.479
507.181	958.812
507.673	956.066

508.164	953.202
508.655	959.126
509.146	949.551
509.638	950.708
510.129	936.8
510.62	955.179
511.109	968.262
511.6	981.271
512.091	953.272
512.582	962.326
513.074	966.171
513.565	975.762
514.056	971.572
514.547	963.356
515.035	976.858
515.526	961.281
516.017	958.951
516.508	960.537
516.999	977.894
517.49	958.971
517.979	968.454
518.469	984.893
518.96	972.836
519.451	957.293
519.941	970.274
520.432	973.409
520.921	980.664

521.411	965.667
521.902	966.023
522.392	939.662
522.881	959.391
523.371	954.851
523.862	964.21
524.352	943.533
524.841	956.081
525.331	958.286
525.821	950.676
526.312	960.057
526.8	947.157
527.29	951.15
527.781	958.921
528.269	954.005
528.759	962.977
529.249	959.514
529.739	953.278
530.227	957.468
530.718	934.259
531.208	959.656
531.696	944.51
532.186	955.959
532.676	936.21
533.164	942.793
533.654	962.757
534.141	940.264

534.631	966.277
535.121	936.993
535.609	956.173
536.099	939.853
536.589	959.912
537.076	951.919
537.566	926.177
538.054	935.833
538.543	923.503
539.033	921.945
539.521	952.328
540.01	927.637
540.498	939.07
540.987	935.139
541.475	910.764
541.964	946.421
542.452	950.547
542.941	951.926
543.429	952.206
543.918	923.227
544.405	930.635
544.895	940.003
545.382	927.035
545.871	910.11
546.359	955.718
546.848	952.876
547.335	940.981

547.824	945.798
548.311	945.883
548.8	929.896
549.287	935.755
549.777	947.723
550.264	944.2
550.753	932.351
551.24	966.63
551.727	917.957
552.215	946.029
552.702	912.03
553.191	933.412
553.678	937.25
554.167	929.335
554.654	951.95
555.141	927.993
555.629	932.283
556.116	952.544
556.603	941.136
557.091	960.341
557.578	962.824
558.065	962.94
558.553	947.307
559.04	945.909
559.526	966.81
560.015	951.906
560.501	953.743

560.988	956.356
561.476	963.029
561.963	955.04
562.449	947.65
562.937	965.376
563.424	957.115
563.91	961.404
564.396	961.207
564.885	972.525
565.371	956.675
565.857	948.319
566.345	971.845
566.831	956.839
567.317	931.61
567.803	949.113
568.289	962.361
568.778	972.032
569.264	979.554
569.75	980.896
570.236	988.8
570.723	984.382
571.209	972.671
571.695	974.52
572.181	972.875
572.667	976.526
573.153	978.405
573.641	979.854

574.126	971.016
574.612	968.754
575.098	961.558
575.583	976.939
576.069	991.65
576.555	990.127
577.042	994.326
577.528	1014.81
578.013	1005.88
578.499	987.033
578.984	1003.14
579.47	1006.04
579.955	1010.85
580.441	1021.53
580.926	1027.77
581.411	1020.12
581.897	1018.85
582.384	1008.65
582.869	1026.71
583.355	1023.78
583.84	1007.4
584.325	1004.07
584.81	1000.46
585.295	1013.28
585.781	1034.92
586.266	1029.46
586.751	1012.65

587.236	1016.7
587.721	1032.65
588.206	1039.13
588.691	1025.57
589.176	1047.49
589.661	1042
590.146	1040.78
590.63	1012.64
591.115	1022.59
591.598	1044.49
592.083	1025.9
592.568	1015.68
593.052	1022.65
593.537	1037.98
594.022	1037.42
594.506	1040.38
594.991	1035.44
595.476	1059.64
595.96	1033.79
596.445	1030.09
596.929	1034.77
597.412	1050.95
597.896	1033.2
598.381	1036.7
598.865	1047.43
599.35	1049.39
599.834	1047.43

600.318	1042.92
600.801	1027.45
601.285	1048.71
601.769	1041.63
602.254	1052.31
602.738	1036.39
603.22	1032.24
603.704	1011.36
604.188	1061.65
604.672	1053.52
605.156	1047.54
605.639	1032.59
606.123	1045.13
606.607	1047.02
607.091	1031.98
607.573	1028.21
608.057	1054.48
608.54	1026.54
609.024	1055.81
609.506	1010.19
609.99	1016.72
610.474	1014.19
610.958	1045.63
611.439	1031.86
611.923	1046.68
612.407	1056.59
612.888	1045.57

613.372	1054.59
613.856	1042.97
614.337	1029.81
614.821	1014.2
615.305	1043.21
615.786	1059.44
616.269	1019.08
616.753	1024.78
617.234	1050.57
617.718	1048.31
618.199	1046.42
618.201	1046.38
618.687	1028.34
619.174	1046.31
619.659	1058.22
620.145	1028.19
620.63	1036.14
621.115	1063.81
621.602	1031.99
622.088	1030.1
622.573	1038.03
623.058	1063.1
623.543	994.444
624.028	1012.35
624.513	1037.57
625	1064.77
625.485	1045.35

625.97	1064.59
626.455	1050.2
626.94	1041.73
627.425	1039.42
627.91	1046.74
628.395	1055.22
628.88	1093.27
629.365	1039.75
629.852	1053.13
630.336	1044.25
630.821	1053.71
631.306	1030.69
631.79	1044.86
632.275	1044.34
632.76	1012.94
633.244	1052.71
633.729	1006.52
634.214	1054.86
634.698	1059.52
635.183	1026.34
635.667	1036.8
636.151	1045.77
636.636	1093.59
637.12	1041.65
637.605	1100.17
638.089	1069.67
638.573	1043.23

639.058	1037.3
639.542	1041.82
640.026	1050.52
640.51	1044.21
640.995	1034.19
641.479	1068.92
641.963	1039.75
642.445	1053.49
642.929	1034.97
643.413	1080.19
643.897	1048.93
644.381	1069.27
644.865	1041.23
645.349	1045.33
645.833	1062.7
646.317	1083.46
646.801	1076.75
647.283	1067.69
647.767	1057.84
648.25	1072.95
648.734	1071.34
649.218	1058.15
649.702	1074.94
650.185	1073.94
650.667	1060.51
651.151	1038.37
651.634	1062.59

652.118	1063.13
652.602	1097.71
653.083	1058.15
653.567	1098.78
654.05	1079.6
654.534	1107.29
655.017	1061.55
655.499	1049.3
655.982	1045.5
656.465	1078.3
656.949	1075.19
657.43	1072.37
657.913	1067.5
658.397	1087.98
658.88	1096.63
659.361	1071.58
659.844	1081.52
660.327	1065.23
660.81	1046.09
661.292	1072.35
661.775	1062.38
662.258	1074
662.739	1066.27
663.222	1067.84
663.705	1107.98
664.186	1089.1
664.669	1089.49

665.152	1050.17
665.633	1104.65
666.115	1082.52
666.598	1077.05
667.079	1088.15
667.562	1096.57
668.045	1074.7
668.525	1084.86
669.008	1105.35
669.489	1122.17
669.971	1094.68
670.454	1090.23
670.935	1125.1
671.417	1095.15
671.898	1087.55
672.38	1093.99
672.861	1122.53
673.343	1105.03
673.826	1120.7
674.306	1111.4
674.788	1079.72
675.269	1115.14
675.751	1130.03
676.231	1103.17
676.714	1136.07
677.194	1107.58
677.676	1099.36

678.156	1127.57
678.639	1119.15
679.119	1097.63
679.601	1106.97
680.081	1119.06
680.563	1108.22
681.043	1126.42
681.525	1082.76
682.005	1098.67
682.487	1086.1
682.967	1115.34
683.449	1092.4
683.929	1129.58
684.409	1077.59
684.891	1099.21
685.371	1120.43
685.852	1138.72
686.332	1107.49
686.814	1102.92
687.294	1090.44
687.773	1113.75
688.255	1110.51
688.735	1127.25
689.216	1116.47
689.696	1111.65
690.176	1108.51
690.657	1108.75

691.137	1079.39
691.616	1088.57
692.098	1107.2
692.577	1085.69
693.057	1105.22
693.538	1089.63
694.017	1101.7
694.497	1110.06
694.978	1099.16
695.457	1108.74
695.937	1114.42
696.416	1084.59
696.897	1108.39
697.376	1100.41
697.855	1113.09
698.336	1073.22
698.816	1111.78
699.295	1100
699.774	1092.98
700.255	1086.59
700.734	1098.01
701.213	1101.45
701.692	1099.22
702.173	1110.72
702.652	1094.74
703.131	1101.54
703.609	1109.5

704.088	1100.78
704.569	1096.31
705.048	1070.91
705.527	1089.75
706.005	1095.4
706.484	1115.48
706.963	1089.71
707.443	1081.83
707.922	1101.6
708.401	1082.48
708.879	1098.96
709.358	1077.97
709.836	1099.56
710.315	1113.65
710.795	1087.62
711.274	1058.6
711.752	1111.82
712.231	1085.31
712.709	1060.12
713.187	1069.94
713.666	1078.1
714.144	1065.15
714.622	1100.31
715.101	1073.4
715.579	1087.21
716.059	1108.16
716.537	1100.12

717.015	1091.78
717.494	1071.84
717.972	1063.88
718.45	1073.18
718.928	1079.51
719.406	1062.92
719.884	1079.2
720.362	1107.56
720.84	1084.78
721.318	1060.48
721.796	1058.1
722.273	1080.44
722.751	1071.89
723.229	1090.18
723.707	1084.35
724.185	1064.16
724.662	1076.91
725.138	1072.16
725.616	1078.78
726.094	1085.66
726.571	1076.87
727.049	1087.34
727.526	1080.87
728.004	1074.13
728.482	1079.84
728.959	1100.18
729.437	1055.58

729.914	1082.03
730.392	1070.89
730.867	1090.08
731.344	1051.31
731.822	1077.99
732.299	1083.16
732.776	1083.68
733.254	1093.83
733.731	1095.82
734.206	1071.71
734.683	1086.19
735.161	1077.37
735.638	1099.61
736.115	1086.09
736.59	1075.91
737.067	1083.68
737.544	1084.8
738.021	1099.24
738.498	1079.65
738.973	1109.67
739.45	1099.04
739.927	1096.31
740.404	1095.27
740.879	1093.55
741.356	1099.55
741.833	1102.31
742.31	1090.94

742.784	1109.89
743.261	1109.69
743.738	1095.62
744.215	1101.57
744.689	1091.67
745.166	1098.64
745.643	1114.33
746.117	1115.32
746.594	1111.73
747.07	1115.69
747.545	1122.53
748.022	1121.97
748.498	1101.93
748.973	1115.29
749.449	1119.13
749.925	1107.33
750.4	1088.13
750.876	1119.03
751.352	1117.88
751.827	1110.64
752.303	1116.35
752.779	1119.36
753.254	1132.25
753.73	1113.69
754.204	1125.55
754.68	1141.56
755.154	1126.99

755.63	1127.81
756.107	1134.87
756.581	1147.08
757.057	1120.37
757.531	1138.4
758.007	1145.68
758.481	1140.61
758.957	1121.56
759.431	1149.4
759.906	1166.35
760.382	1135.14
760.856	1136.07
761.332	1156.18
761.806	1151.69
762.282	1154.27
762.755	1156.07
763.229	1138.71
763.705	1135.19
764.178	1148.23
764.654	1159.41
765.128	1161.35
765.603	1164.42
766.077	1163.83
766.552	1150.01
767.026	1152.22
767.501	1165.97
767.975	1169.74

768.448	1165.35
768.924	1157.78
769.397	1136.4
769.873	1160.25
770.346	1182.32
770.819	1157.95
771.295	1188.84
771.768	1177.18
772.243	1168.29
772.716	1176.06
773.19	1175.19
773.665	1157.93
774.138	1166.67
774.611	1187.02
775.086	1172.2
775.559	1156.89
776.032	1176.26
776.507	1169.24
776.98	1170.55
777.453	1181.06
777.928	1166.43
778.401	1192.74
778.874	1179.82
779.347	1186.39
779.822	1188.27
780.295	1183.27
780.767	1168.3

781.242	1187.94
781.715	1194.07
782.188	1175.38
782.66	1185.01
783.135	1194.96
783.608	1206.31
784.08	1206.17
784.553	1181.85
785.026	1196.33
785.5	1200.9
785.973	1198.86
786.445	1193.53
786.918	1190.33
787.39	1193.68
787.864	1184.36
788.337	1158.48
788.809	1193.02
789.282	1202.73
789.754	1199.11
790.226	1167.87
790.701	1172.4
791.173	1165.46
791.645	1194.64
792.117	1180.49
792.589	1191.55
793.062	1208.06
793.534	1191.42

794.006	1190.61
794.478	1207.7
794.952	1204.45
795.424	1178.89
795.896	1171.48
796.368	1205.39
796.84	1207.58
797.312	1168.94
797.784	1201.06
798.256	1169.63
798.728	1166.31
799.199	1210.76
799.671	1136.67
800.143	1148.05
800.615	1182.81
801.087	1197.73
801.558	1183.33
802.03	1173.15
802.502	1174.28
802.973	1189.66
803.445	1189.87
803.917	1178.7
804.388	1187.76
804.86	1186.08
805.331	1184.53
805.803	1158.74
806.274	1162.6

806.746	1193.34
807.217	1165.16
807.688	1157.34
808.16	1189.71
808.631	1157.86
809.102	1184.97
809.572	1170.3
810.043	1160.13
810.514	1166.14
810.986	1166.56
811.457	1149.22
811.928	1153.89
812.399	1152.54
812.87	1179.48
813.341	1152.49
813.81	1159.65
814.281	1158.19
814.752	1149.06
815.223	1162.52
815.694	1170.56
816.165	1159.02
816.634	1143.81
817.105	1159.63
817.576	1152.29
818.047	1142.53
818.518	1138.68
818.987	1144.14

819.457	1158.78
819.928	1165.68
820.399	1164.74
820.87	1178.36
821.338	1165.14
821.809	1159.59
822.28	1184.73
822.75	1158.72
823.219	1157.27
823.689	1158.93
824.16	1149.81
824.628	1155.05
825.099	1161.89
825.569	1152.67
826.04	1127.12
826.508	1150.58
826.979	1173.4
827.449	1172.81
827.917	1156.5
828.388	1148.27
828.858	1150.98
829.326	1146.42
829.797	1144.35
830.267	1164.81
830.735	1179.35
831.205	1187.43
831.675	1174.85

832.143	1165.17
832.614	1172.27
833.082	1173.84
833.552	1173.12
834.022	1177.35
834.49	1166.43
834.96	1169.08
835.428	1178.1
835.898	1176.3
836.367	1168.55
836.835	1175.28
837.305	1179.19
837.773	1172.6
838.243	1154.17
838.711	1172.08
839.18	1196.51
839.648	1198.52
840.118	1180.61
840.586	1175.74
841.055	1194.38
841.523	1180.59
841.992	1178.85
842.46	1164.74
842.93	1172.08
843.397	1201.36
843.867	1172.51
844.334	1185.83

844.804	1169.01
845.271	1171.51
845.741	1184.42
846.208	1212.75
846.677	1218.51
847.145	1190.64
847.612	1188.43
848.081	1211.88
848.549	1200.1
849.018	1206.74
849.485	1199.36
849.954	1227.46
850.421	1212.67
850.889	1196.29
851.358	1216.05
851.825	1223.48
852.292	1217.52
852.761	1250
853.228	1195.47
853.695	1217.48
854.164	1220.57
854.631	1212
855.1	1219.85
855.567	1215.09
856.034	1229.12
856.501	1236.14
856.969	1226.69

857.436	1202.56
857.903	1198.57
858.372	1225.22
858.839	1236.73
859.305	1213.27
859.774	1229.99
860.241	1194.97
860.707	1242.36
861.174	1197.12
861.621	1231.14
862.091	1223.36
862.56	1231.29
863.03	1238.92
863.501	1199.67
863.971	1223.37
864.442	1238.5
864.912	1244.79
865.38	1219.65
865.851	1207.92
866.321	1229.26
866.791	1231.23
867.262	1207.85
867.732	1246.99
868.2	1235.12
868.67	1280.83
869.14	1234.75
869.61	1264.14

870.081	1252.34
870.549	1273.82
871.019	1224.02
871.489	1234.14
871.959	1241.23
872.427	1243.51
872.897	1285.13
873.367	1250.15
873.836	1240.22
874.304	1231.42
874.774	1233.52
875.244	1263.88
875.712	1271.49
876.182	1259.51
876.651	1240.78
877.121	1255.37
877.589	1276.08
878.058	1258.66
878.528	1235.04
878.996	1243.42
879.465	1262.18
879.935	1254.03
880.403	1279.55
880.872	1253.32
881.342	1262.27
881.809	1255.47
882.279	1317.93

882.746	1249.78
883.215	1273.43
883.685	1267.58
884.152	1279.01
884.621	1255.05
885.089	1285.65
885.558	1284.08
886.027	1252.42
886.495	1272.64
886.964	1275.31
887.431	1260.63
887.9	1248.48
888.369	1271.98
888.836	1257.82
889.306	1221.47
889.773	1267.34
890.242	1233.91
890.709	1235.57
891.178	1228.67
891.645	1234.95
892.114	1278.52
892.581	1262.76
893.049	1251.19
893.516	1249.13
893.985	1261.79
894.452	1241.11
894.921	1260.87

895.388	1250.42
895.856	1239.45
896.323	1260.33
896.792	1241.22
897.259	1260.64
897.725	1251.56
898.194	1245.6
898.661	1249.99
899.129	1242.65
899.596	1241.86
900.064	1233.87
900.531	1229.92
900.997	1259.59
901.466	1281.14
901.932	1260.97
902.401	1218.09
902.867	1233.66
903.333	1223.79
903.802	1282.94
904.268	1232.66
904.734	1245.81
905.203	1264.53
905.669	1247.42
906.135	1255.82
906.603	1245.1
907.07	1219.81
907.536	1219.17

908.004	1230.4
908.47	1245.89
908.936	1239
909.404	1247.8
909.87	1240.82
910.336	1238.98
910.804	1244
911.27	1247.57
911.736	1254.56
912.202	1258.07
912.67	1242.78
913.136	1261.13
913.602	1235.31
914.067	1216.76
914.535	1249.46
915.001	1265.1
915.467	1244.84
915.932	1218.86
916.4	1252.22
916.866	1251.44
917.331	1242.33
917.797	1260.14
918.263	1255.97
918.73	1225.04
919.196	1242.64
919.661	1253.53
920.127	1224.3

920.592	1264.77
921.058	1225.84
921.525	1243.06
921.991	1254.72
922.456	1218.12
922.921	1233.3
923.387	1246.43
923.852	1245.41
924.317	1234.59
924.783	1222.21
925.25	1244.57
925.715	1216.22
926.18	1220.74
926.646	1253.79
927.111	1251.03
927.576	1215.22
928.041	1252.09
928.506	1231.52
928.971	1217.63
929.436	1233.08
929.901	1246.06
930.366	1221.65
930.831	1208.52
931.296	1223.99
931.761	1231.55
932.226	1242.09
932.691	1242.32

933.155	1229.39
933.62	1205.62
934.085	1260.37
934.55	1235.87
935.015	1234.93
935.479	1261.76
935.944	1259.39
936.409	1249.37
936.873	1255.75
937.338	1245.63
937.802	1263.11
938.267	1233.73
938.732	1260.06
939.196	1235.62
939.661	1258.62
940.125	1270.76
940.59	1280.05
941.054	1271.23
941.518	1252.21
941.981	1231.56
942.445	1261.99
942.91	1256.42
943.374	1278.96
943.838	1268.32
944.302	1282.36
944.767	1277.22
945.231	1263.78

945.693	1258.63
946.157	1268.51
946.621	1273.35
947.086	1259.58
947.55	1273.61
948.014	1262.25
948.476	1278.97
948.94	1289.39
949.404	1268.55
949.868	1271.9
950.332	1298.28
950.794	1286.97
951.258	1291.85
951.721	1262.24
952.185	1290.99
952.649	1294.47
953.111	1291.7
953.575	1303.91
954.039	1307.07
954.502	1302.1
954.964	1291.38
955.428	1282.53
955.891	1288.49
956.353	1295.86
956.817	1319.19
957.28	1307.21
957.744	1317.48

958.205	1310.37
958.669	1302.54
959.132	1313.73
959.594	1310.23
960.057	1315.62
960.521	1304.52
960.982	1343.11
961.446	1316.3
961.909	1291.41
962.37	1306.31
962.834	1334.83
963.297	1327.4
963.758	1334.86
964.221	1347.13
964.683	1335.66
965.146	1321.84
965.609	1328.26
966.07	1348.31
966.533	1335.36
966.994	1340.61
967.457	1332.47
967.921	1350.08
968.382	1338.37
968.845	1355.56
969.306	1337.66
969.768	1338.88
970.229	1334.36

970.692	1357.06
971.153	1337.08
971.616	1319.56
972.077	1341.38
972.54	1344.86
973.001	1337.39
973.463	1326.27
973.924	1339.84
974.387	1348.11
974.848	1354.92
975.31	1353.67
975.771	1326.44
976.234	1342.68
976.694	1368.05
977.157	1359.45
977.617	1370.58
978.08	1354.67
978.54	1367.71
979.003	1353.22
979.463	1358.51
979.924	1368.05
980.386	1349.13
980.847	1349.6
981.309	1374.63
981.769	1370.7
982.23	1381.27
982.692	1365.67

983.152	1359.07
983.614	1351.19
984.075	1365.29
984.535	1373.86
984.997	1381.92
985.457	1375.26
985.917	1368.15
986.38	1375.51
986.84	1391.99
987.3	1379.16
987.762	1380.14
988.222	1375.85
988.682	1377.61
989.144	1365.45
989.604	1355.72
990.064	1346.99
990.524	1360.3
990.985	1362.13
991.445	1370.07
991.905	1378.28
992.367	1395.53
992.827	1350.16
993.286	1357.99
993.746	1359.12
994.208	1382.5
994.668	1357.08
995.127	1360.96

995.587	1363.23
996.047	1353.76
996.508	1363.51
996.968	1350.84
997.427	1386.75
997.887	1353.7
998.346	1358.28
998.808	1356.31
999.267	1353.41
999.727	1337.59
1000.19	1354.84
1000.65	1383.75
1001.1	1350.17
1001.57	1339.38
1002.03	1355.41
1002.48	1341.52
1002.94	1356.61
1003.4	1356.94
1003.86	1345
1004.32	1342.96
1004.78	1357.98
1005.24	1330.46
1005.7	1347.87
1006.16	1363.86
1006.62	1345.53
1007.08	1343.08
1007.54	1344.63

1008	1316.69
1008.46	1335.04
1008.91	1341.94
1009.37	1354.5
1009.83	1345.28
1010.29	1330.24
1010.75	1333.13
1011.21	1365.06
1011.67	1343.66
1012.13	1328.34
1012.59	1362.91
1013.04	1343.63
1013.5	1333.79
1013.96	1332.75
1014.42	1316.78
1014.88	1330.4
1015.34	1344.29
1015.8	1360.17
1016.25	1330.2
1016.71	1340.08
1017.17	1325.4
1017.63	1329.24
1018.09	1341.04
1018.55	1332.78
1019.01	1319.17
1019.46	1323.86
1019.92	1334.59

1020.38	1319.79
1020.84	1327.25
1021.3	1322.56
1021.75	1320.33
1022.21	1324.9
1022.67	1350.77
1023.13	1326.64
1023.59	1308.55
1024.04	1334.9
1024.5	1324.63
1024.96	1337.78
1025.42	1323.02
1025.88	1330.2
1026.33	1326.35
1026.79	1334.84
1027.25	1327.47
1027.71	1339.03
1028.16	1331.33
1028.62	1327.75
1029.08	1306.56
1029.54	1321.51
1029.99	1318.06
1030.45	1311.62
1030.91	1336.08
1031.37	1326.21
1031.82	1315.98
1032.28	1298.42

1032.74	1286.67
1033.19	1340.14
1033.65	1310.96
1034.11	1319.56
1034.57	1316.1
1035.02	1320.93
1035.48	1325.17
1035.94	1313.59
1036.39	1349.34
1036.85	1304.15
1037.31	1328.13
1037.76	1297.91
1038.22	1342.5
1038.68	1302.14
1039.14	1332.94
1039.59	1317.71
1040.05	1323.14
1040.51	1323.44
1040.96	1312.13
1041.42	1324.23
1041.88	1320.8
1042.33	1310.01
1042.79	1329.92
1043.25	1341.88
1043.7	1326.6
1044.16	1328.69
1044.61	1331.24

1045.07	1339.44
1045.53	1312.89
1045.98	1324.36
1046.44	1299.19
1046.9	1337.13
1047.35	1351.22
1047.81	1311.64
1048.26	1360.93
1048.72	1362.66
1049.18	1318.56
1049.63	1336.78
1050.09	1350.96
1050.54	1318.89
1051	1363.98
1051.46	1324.84
1051.91	1350.09
1052.37	1328.55
1052.82	1336.65
1053.28	1360.52
1053.73	1359.2
1054.19	1379.08
1054.64	1337.44
1055.1	1366.82
1055.56	1360.55
1056.01	1372.77
1056.47	1352.52
1056.92	1351.7

1057.38	1377.48
1057.83	1388.58
1058.29	1373.3
1058.74	1383.48
1059.2	1363.74
1059.65	1371.7
1060.11	1376.31
1060.57	1342.42
1061.02	1367.5
1061.48	1382.45
1061.93	1398.37
1062.38	1386.96
1062.84	1400.34
1063.29	1388.8
1063.75	1385.38
1064.21	1386.85
1064.66	1356.15
1065.11	1356.41
1065.57	1358.38
1066.02	1377.42
1066.48	1393.8
1066.93	1400.28
1067.39	1395.09
1067.84	1376.28
1068.3	1384.56
1068.75	1397.65
1069.21	1401.37

1069.66	1425.65
1070.11	1391.18
1070.57	1381.09
1071.02	1408.97
1071.48	1409.86
1071.93	1403.43
1072.39	1401.93
1072.84	1408.11
1073.29	1414.63
1073.75	1406
1074.2	1411.35
1074.66	1404.05
1075.11	1402.61
1075.56	1436.41
1076.02	1445.46
1076.47	1428.17
1076.93	1421.04
1077.38	1424.63
1077.83	1447.86
1078.29	1453.01
1078.74	1446.37
1079.19	1426.06
1079.65	1420.68
1080.1	1440.38
1080.56	1415.65
1081.01	1427.86
1081.46	1448.61

1081.92	1445.36
1082.37	1461.33
1082.82	1451.79
1083.28	1439.1
1083.73	1445.18
1084.18	1452.61
1084.64	1447.23
1085.09	1427
1085.54	1441.61
1086	1454.8
1086.45	1433.26
1086.9	1465.22
1087.36	1467.94
1087.81	1446.13
1088.26	1439.66
1088.71	1455.14
1089.17	1449.31
1089.62	1442.98
1090.07	1428.45
1090.53	1457.27
1090.98	1454.37
1091.43	1450.65
1091.88	1460.5
1092.34	1467.99
1092.79	1476.42
1093.24	1495.24
1093.69	1475.19

1094.15	1412.16
1094.6	1447.84
1095.05	1466.72
1095.5	1473.53
1095.96	1455.68
1096.41	1461.48
1096.86	1452.44
1097.31	1429.85
1097.77	1450.68
1098.22	1436.03
1098.67	1457.66
1099.12	1478.6
1099.57	1460.74
1100.03	1468.75
1100.48	1451.77
1100.86	1465.27
1101.31	1466.98
1101.77	1491.7
1102.22	1464.75
1102.68	1455.49
1103.13	1457.38
1103.59	1405.05
1104.04	1434.29
1104.5	1474.19
1104.95	1465.82
1105.41	1486.16
1105.86	1450.94

1106.32	1474.42
1106.77	1492.18
1107.23	1432.33
1107.68	1447.92
1108.14	1428.69
1108.59	1467.67
1109.05	1466.71
1109.5	1498.9
1109.95	1474.15
1110.41	1489.43
1110.86	1445.79
1111.32	1447.92
1111.77	1434.77
1112.23	1482.86
1112.68	1453.29
1113.13	1453.26
1113.59	1459.05
1114.04	1418.69
1114.5	1477.48
1114.95	1489.6
1115.41	1515.73
1115.86	1432.63
1116.31	1460.17
1116.77	1438.89
1117.22	1443.54
1117.67	1447
1118.13	1477.3

1118.58	1456.86
1119.04	1431.29
1119.49	1459.91
1119.94	1445.29
1120.4	1440.55
1120.85	1435.51
1121.3	1437.19
1121.76	1454.83
1122.21	1420.49
1122.66	1419.76
1123.12	1418.39
1123.57	1440.37
1124.03	1445.07
1124.48	1425.28
1124.93	1450.8
1125.38	1430.34
1125.84	1430.7
1126.29	1403.39
1126.74	1438.86
1127.2	1439.3
1127.65	1458.05
1128.1	1427.24
1128.56	1459.29
1129.01	1436.92
1129.46	1433.94
1129.91	1426.57
1130.37	1426.96

1130.82	1440.27
1131.27	1427.43
1131.73	1470.02
1132.18	1435.41
1132.63	1413.86
1133.09	1419.94
1133.54	1410.79
1133.99	1454.62
1134.44	1459.7
1134.9	1420.7
1135.35	1420.66
1135.8	1449.6
1136.25	1431.8
1136.7	1400.46
1137.16	1411.93
1137.61	1421.1
1138.06	1435.02
1138.51	1450.64
1138.97	1451.42
1139.42	1451.08
1139.87	1450.69
1140.32	1432.61
1140.78	1445.48
1141.23	1416.44
1141.68	1446.18
1142.13	1429.14
1142.58	1414.31

1143.04	1429.82
1143.49	1426.47
1143.94	1400.86
1144.39	1446.27
1144.84	1423.87
1145.29	1424.11
1145.75	1414.87
1146.2	1449.21
1146.65	1465.49
1147.1	1452.91
1147.55	1456.39
1148	1441.48
1148.46	1444.57
1148.91	1435.74
1149.36	1455.05
1149.81	1440.8
1150.26	1417.72
1150.71	1453.63
1151.16	1401.03
1151.62	1448.89
1152.07	1477.1
1152.52	1455.08
1152.97	1459.34
1153.42	1432.23
1153.87	1444.19
1154.32	1457.19
1154.77	1438.19

1155.22	1464.21
1155.68	1459.65
1156.13	1425.6
1156.58	1463.44
1157.03	1466.7
1157.48	1450.65
1157.93	1463.72
1158.38	1455.04
1158.83	1449.33
1159.28	1483.06
1159.73	1426
1160.18	1450.5
1160.63	1435.22
1161.08	1457.22
1161.54	1464.59
1161.99	1484.04
1162.44	1457.61
1162.89	1434.02
1163.34	1499.39
1163.79	1472
1164.24	1474.65
1164.69	1470.57
1165.14	1454.95
1165.59	1446.72
1166.04	1470.58
1166.49	1455.97
1166.94	1448.29

1167.39	1483.07
1167.84	1506.89
1168.29	1496.13
1168.74	1494.91
1169.19	1502.22
1169.64	1516.05
1170.09	1504.65
1170.54	1503.18
1170.99	1485.88
1171.44	1515.91
1171.89	1502.27
1172.34	1504.45
1172.79	1501.75
1173.24	1509.1
1173.69	1523.1
1174.14	1519.17
1174.59	1487.99
1175.03	1520.7
1175.48	1526.02
1175.93	1508.28
1176.38	1504.28
1176.83	1507.43
1177.28	1523.96
1177.73	1525.59
1178.18	1523.69
1178.63	1548.31
1179.08	1541.02

1179.53	1533.98
1179.98	1542.97
1180.43	1509.31
1180.88	1544.22
1181.32	1510.29
1181.77	1534.96
1182.22	1545.84
1182.67	1544.12
1183.12	1551.08
1183.57	1573.44
1184.02	1540.85
1184.47	1532.27
1184.92	1555.75
1185.37	1573.07
1185.81	1547.35
1186.26	1538.31
1186.71	1531.1
1187.16	1543.8
1187.61	1552.14
1188.06	1533.44
1188.5	1574.48
1188.95	1566.69
1189.4	1550.49
1189.85	1556.11
1190.3	1555.94
1190.75	1560.93
1191.2	1568.91

1191.64	1565.65
1192.09	1567.73
1192.54	1556.25
1192.99	1595.52
1193.44	1594.72
1193.88	1577.09
1194.33	1579.57
1194.78	1578.84
1195.23	1565.23
1195.68	1583.58
1196.12	1571.75
1196.57	1579.27
1197.02	1549.58
1197.47	1582.86
1197.92	1564.72
1198.36	1565.09
1198.81	1582.51
1199.26	1576.31
1199.71	1585.06
1200.15	1575.12
1200.6	1602.06
1201.05	1596.8
1201.5	1591.6
1201.94	1622.28
1202.39	1609.01
1202.84	1567.55
1203.29	1577.02

1203.73	1587.95
1204.18	1577.92
1204.63	1605.78
1205.07	1599.43
1205.52	1598.14
1205.97	1589.35
1206.42	1582.17
1206.86	1585.73
1207.31	1609.34
1207.76	1586.23
1208.2	1582.85
1208.65	1594.83
1209.1	1589.24
1209.55	1594.48
1209.99	1593.01
1210.44	1586.78
1210.89	1593.19
1211.33	1592.23
1211.78	1569.24
1212.23	1612.23
1212.67	1563.61
1213.12	1596.05
1213.57	1569.17
1214.01	1574.05
1214.46	1572.35
1214.9	1584.23
1215.35	1592.82

1215.8	1584.57
1216.24	1591.37
1216.69	1597.03
1217.14	1581.83
1217.58	1556.9
1218.03	1576.68
1218.48	1578.42
1218.92	1564.56
1219.37	1537.53
1219.82	1580.89
1220.26	1545.67
1220.71	1546.63
1221.15	1567.45
1221.6	1564.97
1222.04	1561.08
1222.49	1576.68
1222.94	1557.48
1223.38	1581.07
1223.83	1536.93
1224.27	1569.38
1224.72	1563.76
1225.17	1574.14
1225.61	1579.26
1226.06	1563.54
1226.5	1571.6
1226.95	1548.71
1227.39	1539.64

1227.84	1577.71
1228.28	1559.39
1228.73	1544.14
1229.18	1552.74
1229.62	1534.2
1230.07	1538.53
1230.51	1555.33
1230.96	1557.17
1231.4	1544.99
1231.85	1527.45
1232.29	1526.83
1232.74	1541.42
1233.18	1521.92
1233.63	1543.84
1234.07	1557.66
1234.52	1540.38
1234.96	1540.41
1235.41	1532.15
1235.85	1540.85
1236.3	1509.92
1236.74	1525.09
1237.19	1548.55
1237.63	1531.72
1238.08	1523.62
1238.52	1536.9
1238.97	1534.69
1239.41	1517.55

1239.85	1504.53
1240.3	1533.48
1240.75	1537.35
1241.19	1547.39
1241.63	1524.96
1242.08	1507.02
1242.52	1519.67
1242.97	1501.88
1243.41	1517.21
1243.86	1511.83
1244.3	1524.64
1244.74	1526.22
1245.19	1526.9
1245.63	1530.48
1246.08	1494.17
1246.52	1514.31
1246.96	1530.85
1247.41	1514.42
1247.85	1491.27
1248.3	1507.68
1248.74	1527.22
1249.18	1508.51
1249.63	1502.16
1250.07	1500.03
1250.52	1490.77
1250.96	1511.28
1251.4	1522.88

1251.85	1533.24
1252.29	1508.36
1252.73	1513.42
1253.18	1503.78
1253.62	1513.74
1254.06	1512.22
1254.51	1492.97
1254.95	1484.51
1255.4	1533.48
1255.84	1522.37
1256.28	1494.04
1256.73	1515.84
1257.17	1503.81
1257.61	1507.96
1258.05	1484.96
1258.5	1496.37
1258.94	1494.41
1259.38	1507.27
1259.83	1499.94
1260.27	1507.3
1260.71	1509.3
1261.16	1506.94
1261.6	1513.22
1262.04	1509.76
1262.49	1505.94
1262.93	1504.71
1263.37	1488.05

1263.81	1501.57
1264.26	1526.1
1264.7	1516.47
1265.14	1555.46
1265.58	1504.96
1266.03	1485.14
1266.47	1496.01
1266.91	1485.13
1267.35	1498.97
1267.8	1505.25
1268.24	1526.87
1268.68	1525.96
1269.13	1516.48
1269.57	1533.19
1270.01	1518.92
1270.45	1525.5
1270.89	1546.96
1271.34	1545.52
1271.78	1522.84
1272.22	1508.95
1272.66	1545.02
1273.11	1516.63
1273.55	1528.99
1273.99	1561.92
1274.43	1558.39
1274.87	1539.56
1275.32	1544.3

1275.76	1532.5
1276.2	1540.75
1276.64	1554.5
1277.08	1520.8
1277.52	1554.89
1277.96	1567.78
1278.41	1557.57
1278.85	1569.45
1279.29	1556.56
1279.73	1548.79
1280.17	1571.29
1280.61	1562.23
1281.06	1575.34
1281.5	1572.25
1281.94	1541.41
1282.38	1574.73
1282.82	1597.37
1283.26	1617.47
1283.7	1559.39
1284.15	1542.25
1284.59	1587.99
1285.03	1566.68
1285.47	1568.86
1285.91	1573.33
1286.35	1591.07
1286.79	1593.92
1287.23	1576.33

1287.67	1553.83
1288.11	1583.96
1288.56	1595.41
1289	1605.09
1289.44	1603.67
1289.88	1607.95
1290.32	1593.41
1290.76	1570.16
1291.2	1617.37
1291.64	1601.34
1292.08	1623.45
1292.52	1592.44
1292.96	1597.53
1293.4	1639.02
1293.85	1623.65
1294.29	1623.55
1294.72	1613.72
1295.17	1619.07
1295.61	1593.62
1296.05	1590.77
1296.49	1596.74
1296.93	1626.23
1297.37	1628.43
1297.81	1642.84
1298.25	1638.04
1298.69	1626.37
1299.13	1649.62

1299.57	1658.05
1300.01	1645.97
1300.45	1616.82
1300.89	1626.19
1301.33	1628.09
1301.77	1637.17
1302.21	1626.05
1302.65	1634.1
1303.09	1652.07
1303.53	1646.89
1303.97	1661.03
1304.41	1645.09
1304.85	1632.06
1305.29	1670.41
1305.72	1677.03
1306.16	1666.57
1306.6	1644.13
1307.04	1651.95
1307.48	1646.86
1307.92	1661.06
1308.36	1670.99
1308.8	1674.8
1309.24	1682.58
1309.68	1671.49
1310.12	1649.8
1310.56	1655.24
1311	1671.39

1311.44	1680.59
1311.88	1662.58
1312.32	1654.39
1312.76	1673
1313.19	1663.11
1313.63	1661.78
1314.07	1681.15
1314.51	1686.74
1314.95	1675.74
1315.39	1672.43
1315.83	1699.31
1316.27	1705.88
1316.7	1688.65
1317.14	1677.26
1317.58	1664.37
1318.02	1700.95
1318.46	1705.43
1318.9	1680.51
1319.34	1695.64
1319.77	1687.87
1320.21	1673.4
1320.65	1681.66
1321.09	1679.17
1321.53	1673.01
1321.97	1671.87
1322.41	1676.61
1322.84	1680.83

1323.28	1704.93
1323.72	1747.53
1324.16	1706.52
1324.6	1677.46
1325.04	1683.89
1325.47	1691.56
1325.91	1688.1
1326.35	1659.36
1326.79	1656.39
1327.08	1677.64
1327.52	1698.14
1327.96	1658.33
1328.4	1675.66
1328.84	1623.3
1329.29	1647.31
1329.73	1704.2
1330.17	1678.59
1330.61	1674.75
1331.05	1700.16
1331.49	1679.02
1331.93	1711.28
1332.37	1711.94
1332.81	1680.37
1333.25	1670.38
1333.69	1637.84
1334.13	1723.02
1334.57	1679.25

1335.01	1685.3
1335.45	1660.56
1335.89	1671.91
1336.33	1679.78
1336.77	1678.42
1337.21	1688.38
1337.65	1684.16
1338.09	1684.82
1338.54	1656.06
1338.98	1635.53
1339.42	1686.97
1339.86	1648.09
1340.29	1673.42
1340.73	1657.68
1341.17	1676.19
1341.61	1696.3
1342.05	1679.8
1342.49	1644.75
1342.93	1678.38
1343.37	1628.36
1343.81	1666.64
1344.25	1648.5
1344.69	1644.51
1345.13	1660.06
1345.57	1649.44
1346.01	1684.4
1346.45	1644.74

1346.89	1627.81
1347.33	1675.99
1347.77	1655.19
1348.21	1611.58
1348.65	1622.37
1349.09	1651.78
1349.53	1639.07
1349.97	1652.23
1350.41	1632.02
1350.85	1621.59
1351.29	1635.82
1351.72	1629.31
1352.16	1623.87
1352.6	1621.43
1353.04	1652.81
1353.48	1665.85
1353.92	1649.74
1354.36	1661.6
1354.8	1657.52
1355.24	1663.49
1355.67	1613.24
1356.11	1651.79
1356.55	1605.51
1356.99	1634.39
1357.43	1637.15
1357.87	1645.79
1358.31	1647.58

1358.75	1620.2
1359.19	1587.39
1359.62	1620.1
1360.06	1613.9
1360.5	1660.98
1360.94	1602.57
1361.38	1626.51
1361.82	1625.28
1362.25	1613.81
1362.69	1622.38
1363.13	1633.37
1363.57	1608.41
1364.01	1619.92
1364.44	1599.77
1364.88	1598.5
1365.32	1622.81
1365.76	1602.13
1366.2	1600.27
1366.64	1587.09
1367.07	1576.45
1367.51	1588.45
1367.95	1593.15
1368.39	1626.23
1368.83	1610.05
1369.26	1612.38
1369.7	1608.64
1370.14	1613.74

1370.58	1598.79
1371.02	1584.85
1371.45	1579.29
1371.89	1600.78
1372.33	1618.96
1372.77	1600.6
1373.2	1597.7
1373.64	1596.21
1374.08	1584.01
1374.52	1609.76
1374.95	1589.48
1375.39	1601.08
1375.83	1609.3
1376.26	1570.65
1376.7	1604.87
1377.14	1606.81
1377.58	1568.31
1378.01	1600.07
1378.45	1559.17
1378.89	1588.75
1379.32	1602.67
1379.76	1590.24
1380.2	1601.98
1380.64	1605.5
1381.07	1618.93
1381.51	1588.89
1381.95	1594.55

1382.38	1590.57
1382.82	1599.85
1383.26	1589.4
1383.69	1617.85
1384.13	1614.2
1384.57	1620
1385	1567.77
1385.44	1602.67
1385.88	1632.38
1386.31	1587.71
1386.75	1607.79
1387.19	1611.21
1387.62	1579.82
1388.06	1624.03
1388.5	1612.65
1388.93	1603.76
1389.37	1611.03
1389.8	1602.96
1390.24	1596.16
1390.68	1625.46
1391.11	1606.94
1391.55	1615.97
1391.99	1629.16
1392.42	1602.57
1392.86	1622
1393.29	1621.91
1393.73	1620.64

1394.17	1628.68
1394.6	1584.74
1395.04	1603.97
1395.47	1616.85
1395.91	1624.29
1396.35	1619.68
1396.78	1623.99
1397.22	1603.3
1397.65	1606.87
1398.09	1650.97
1398.52	1651.6
1398.96	1621.97
1399.4	1630.21
1399.83	1656.24
1400.27	1639.27
1400.7	1621.88
1401.14	1657.58
1401.57	1644.23
1402.01	1626.13
1402.44	1647.74
1402.88	1637.03
1403.32	1632.22
1403.75	1659.51
1404.19	1637.43
1404.62	1685.27
1405.06	1652.49
1405.49	1654.25

1405.93	1643.27
1406.36	1659.14
1406.8	1672.56
1407.23	1668.99
1407.67	1685.29
1408.1	1666.98
1408.54	1670.5
1408.97	1675.37
1409.41	1683.29
1409.84	1689.26
1410.27	1685.12
1410.71	1693.08
1411.14	1678.84
1411.58	1699.18
1412.01	1712.63
1412.45	1707.08
1412.88	1661.62
1413.32	1680.69
1413.75	1698.14
1414.19	1679.8
1414.62	1718.31
1415.06	1671.63
1415.49	1721.31
1415.92	1696.39
1416.36	1698.12
1416.79	1724.03
1417.23	1704.54

1417.66	1729.02
1418.1	1719.92
1418.53	1729.17
1418.96	1731.67
1419.4	1688.59
1419.83	1714.34
1420.27	1726.75
1420.7	1729.83
1421.13	1727.17
1421.57	1722.66
1422	1733.3
1422.43	1750.41
1422.87	1729.6
1423.3	1738.97
1423.74	1736.02
1424.17	1736.65
1424.6	1741.09
1425.04	1745.3
1425.47	1738.83
1425.91	1754.93
1426.34	1747.19
1426.77	1771.91
1427.21	1737.96
1427.64	1769.5
1428.07	1735.14
1428.51	1738.28
1428.94	1752.64

1429.37	1774.38
1429.81	1797
1430.24	1768.26
1430.67	1740.95
1431.1	1766.31
1431.54	1777.6
1431.97	1774.71
1432.4	1758.09
1432.84	1750.24
1433.27	1737.63
1433.7	1761.6
1434.14	1735.15
1434.57	1751.58
1435	1768.19
1435.44	1781.34
1435.87	1787.17
1436.3	1761.02
1436.73	1762.43
1437.17	1727.37
1437.6	1767.56
1438.03	1781.12
1438.46	1774.8
1438.9	1763.49
1439.33	1766.21
1439.76	1776.38
1440.2	1764.29
1440.63	1790.1

1441.06	1752.93
1441.49	1750.4
1441.92	1772.32
1442.36	1775.6
1442.79	1768.22
1443.22	1761.35
1443.65	1784.05
1444.09	1785.8
1444.52	1766.47
1444.95	1777.11
1445.38	1757.2
1445.82	1765.11
1446.25	1788.17
1446.68	1799.27
1447.11	1758.5
1447.54	1754.92
1447.97	1786.78
1448.41	1759.03
1448.84	1775.3
1449.27	1765.36
1449.7	1749.65
1450.13	1774.02
1450.57	1781.22
1451	1795.36
1451.43	1754.4
1451.86	1756.36
1452.29	1744.49

1452.72	1764.96
1453.16	1781.48
1453.59	1733.86
1454.02	1748.44
1454.45	1746.93
1454.88	1750.88
1455.31	1783.39
1455.75	1784.35
1456.18	1761.93
1456.61	1764.04
1457.04	1742.54
1457.47	1747.28
1457.9	1759.09
1458.33	1782.65
1458.76	1756.92
1459.19	1763.51
1459.62	1758.82
1460.06	1747.96
1460.49	1771.75
1460.92	1753.85
1461.35	1741.2
1461.78	1750.5
1462.21	1731.11
1462.64	1721.24
1463.07	1736.15
1463.5	1729.6
1463.93	1731.22

1464.36	1751.06
1464.8	1747.6
1465.23	1726.1
1465.66	1719.24
1466.09	1717.72
1466.52	1727.73
1466.95	1729.28
1467.38	1720.77
1467.81	1750.5
1468.24	1722.63
1468.67	1737.08
1469.1	1708.37
1469.53	1707.83
1469.96	1729.19
1470.39	1719.06
1470.82	1732.15
1471.25	1742.19
1471.68	1739.23
1472.11	1718.48
1472.54	1698.46
1472.97	1695.18
1473.4	1718.31
1473.83	1709.28
1474.26	1697.36
1474.69	1728.94
1475.12	1719.36
1475.55	1704.53

1475.98	1674.91
1476.41	1715.18
1476.84	1713.08
1477.27	1688.54
1477.7	1713.42
1478.13	1704.18
1478.56	1704.13
1478.99	1708.94
1479.42	1718.58
1479.85	1681.57
1480.28	1680.34
1480.71	1691.84
1481.14	1694.5
1481.57	1674.31
1482	1684.69
1482.42	1713.41
1482.85	1701.26
1483.28	1685.61
1483.71	1674.7
1484.14	1669.08
1484.57	1670.97
1485	1671.73
1485.43	1678.93
1485.86	1677.47
1486.29	1675.51
1486.72	1670.95
1487.15	1648.13

1487.57	1659.09
1488	1665.12
1488.43	1667.12
1488.86	1660.19
1489.29	1681.8
1489.72	1682.36
1490.15	1668.27
1490.58	1680.41
1491.01	1670.76
1491.43	1683.18
1491.86	1679.99
1492.29	1646.58
1492.72	1678.54
1493.15	1682.7
1493.58	1665.97
1494	1674.71
1494.43	1679.53
1494.86	1658.38
1495.29	1669.91
1495.72	1667.76
1496.15	1676.64
1496.57	1690.62
1497	1642.25
1497.43	1645.98
1497.86	1625.12
1498.29	1665.73
1498.72	1671.85

1499.14	1656.46
1499.57	1684.29
1500	1645.07
1500.43	1647.18
1500.86	1683.53
1501.28	1665.64
1501.71	1693.09
1502.14	1659.85
1502.57	1688.17
1502.99	1684.88
1503.42	1682.05
1503.85	1666.86
1504.28	1663.23
1504.71	1660.55
1505.13	1650.76
1505.56	1642.47
1505.99	1650.29
1506.42	1674.64
1506.84	1669.02
1507.27	1669.68
1507.7	1663.65
1508.13	1655.37
1508.55	1672.75
1508.98	1657.15
1509.41	1628.69
1509.84	1678.98
1510.26	1687.92

1510.69	1658.16
1511.12	1661.93
1511.54	1654.37
1511.97	1675.82
1512.4	1696
1512.83	1638.38
1513.25	1692.66
1513.68	1701.78
1514.11	1677.34
1514.53	1624.24
1514.96	1666.59
1515.39	1683.02
1515.81	1683.47
1516.24	1700.39
1516.67	1684.93
1517.09	1678.2
1517.52	1679.47
1517.95	1677.26
1518.37	1693.08
1518.8	1711.38
1519.23	1685.83
1519.65	1691.83
1520.08	1707.54
1520.51	1700.06
1520.93	1695.85
1521.36	1671.65
1521.79	1680.89

1522.21	1690.78
1522.64	1675.57
1523.06	1729.61
1523.49	1693.06
1523.92	1688.97
1524.34	1731.82
1524.77	1735.88
1525.2	1724.54
1525.62	1737.59
1526.05	1734.79
1526.47	1743.61
1526.9	1723.36
1527.33	1725.95
1527.75	1726.23
1528.18	1742.65
1528.6	1730.83
1529.03	1724.93
1529.46	1705.28
1529.88	1739.42
1530.31	1757.05
1530.73	1720.05
1531.16	1757.72
1531.58	1782.26
1532.01	1768.34
1532.44	1739.76
1532.86	1744.79
1533.29	1761.76

1533.71	1765.59
1534.14	1773.42
1534.56	1774.49
1534.99	1764.52
1535.41	1741.4
1535.84	1764.67
1536.26	1777.03
1536.69	1770.4
1537.11	1763.32
1537.54	1748.42
1537.96	1751.15
1538.39	1776.89
1538.81	1786.76
1539.24	1764.68
1539.66	1756.36
1540.09	1774.55
1540.52	1800.82
1540.94	1771.41
1541.36	1785.25
1541.79	1788.16
1542.21	1762.44
1542.64	1760.75
1543.06	1768.23
1543.49	1774.01
1543.91	1790.46
1544.34	1799.98
1544.76	1805.08

1545.19	1808.83
1545.61	1818.47
1546.04	1828.68
1546.46	1824.05
1546.74	1825.32
1547.17	1789.31
1547.6	1787.69
1548.03	1828.83
1548.45	1825.17
1548.88	1817.31
1549.31	1829.04
1549.73	1847.33
1550.16	1845.19
1550.59	1830.58
1551.02	1827.09
1551.44	1845.49
1551.87	1797.64
1552.3	1811.57
1552.72	1844.59
1553.15	1814.14
1553.58	1860.6
1554	1869.9
1554.43	1854.22
1554.86	1826.05
1555.29	1884.91
1555.71	1835.71
1556.14	1862.87

1556.57	1851.33
1556.99	1850.96
1557.42	1804.8
1557.85	1845.85
1558.27	1837.89
1558.7	1821.55
1559.13	1865.53
1559.55	1822.09
1559.98	1838.02
1560.41	1858.87
1560.83	1853.27
1561.26	1851.51
1561.68	1886.21
1562.11	1841.71
1562.54	1819.36
1562.96	1835.57
1563.39	1866.57
1563.82	1844.03
1564.24	1877.14
1564.67	1858.58
1565.09	1883.28
1565.52	1845.66
1565.95	1835.74
1566.37	1861.69
1566.8	1894.56
1567.23	1863.92
1567.65	1853.44

1568.08	1844.71
1568.5	1850.58
1568.93	1879.16
1569.35	1871.03
1569.78	1851.89
1570.2	1839.01
1570.63	1871.03
1571.06	1836.83
1571.48	1861.61
1571.91	1879.35
1572.33	1897.93
1572.76	1833.42
1573.18	1849.58
1573.61	1847.94
1574.04	1876.51
1574.46	1915.71
1574.89	1828.36
1575.31	1899.34
1575.74	1865.88
1576.16	1856.46
1576.59	1894.15
1577.01	1849.83
1577.44	1797.17
1577.86	1858.77
1578.29	1881.14
1578.71	1880.09
1579.14	1867.98

1579.56	1904.06
1579.99	1854.77
1580.41	1836.38
1580.84	1852.29
1581.26	1815.28
1581.69	1856.01
1582.11	1877.4
1582.54	1829.66
1582.96	1870.4
1583.39	1835.55
1583.81	1864.68
1584.24	1845.56
1584.66	1830.79
1585.09	1883.8
1585.51	1848.42
1585.94	1858.97
1586.36	1848.08
1586.79	1840.39
1587.21	1878.71
1587.63	1825.81
1588.06	1819.17
1588.48	1881.43
1588.91	1852.2
1589.33	1854.21
1589.76	1832.24
1590.18	1837.52
1590.6	1857.17

1591.03	1854.27
1591.45	1831.57
1591.88	1838.34
1592.3	1822.21
1592.72	1869.26
1593.15	1823.19
1593.57	1824.8
1594	1840.16
1594.42	1857.7
1594.85	1827.3
1595.27	1837.54
1595.69	1842.96
1596.12	1829.95
1596.54	1811.96
1596.96	1782.65
1597.39	1807.09
1597.81	1833.68
1598.23	1820.61
1598.66	1805.38
1599.08	1831.4
1599.51	1803.02
1599.93	1792.28
1600.35	1841.1
1600.78	1805.14
1601.2	1781.43
1601.62	1804
1602.05	1804.86

1602.47	1806.56
1602.89	1828.57
1603.32	1817.04
1603.74	1794.95
1604.16	1814.45
1604.58	1813.98
1605.01	1791.95
1605.43	1818.87
1605.85	1825.63
1606.28	1786.04
1606.7	1774.76
1607.12	1783.19
1607.55	1784.31
1607.97	1814.71
1608.39	1787.59
1608.82	1790.53
1609.24	1780.76
1609.66	1770.09
1610.08	1790.71
1610.51	1813.24
1610.93	1790.27
1611.35	1765.61
1611.77	1808.49
1612.2	1767.45
1612.62	1775.14
1613.04	1779.6
1613.46	1780.86

1613.89	1770.64
1614.31	1789.78
1614.73	1779.33
1615.16	1766.82
1615.58	1755.25
1616	1744.54
1616.42	1751.77
1616.85	1774.67
1617.27	1760.42
1617.69	1778.94
1618.11	1736.34
1618.53	1745.59
1618.95	1754.97
1619.38	1767.29
1619.8	1751.22
1620.22	1780.25
1620.64	1744.52
1621.07	1744.12
1621.49	1744.69
1621.91	1738.4
1622.33	1753.73
1622.75	1774.6
1623.17	1758.17
1623.6	1777.82
1624.02	1773.08
1624.44	1732.47
1624.86	1764.21

1625.28	1755.05
1625.7	1780.13
1626.13	1762.94
1626.55	1720.11
1626.97	1737.77
1627.39	1741.65
1627.81	1721.91
1628.23	1760.64
1628.65	1718.94
1629.08	1763.13
1629.5	1793.6
1629.92	1758.5
1630.34	1743.02
1630.76	1719.27
1631.18	1736.41
1631.6	1722.07
1632.03	1727
1632.45	1733.94
1632.87	1731.94
1633.29	1717.93
1633.71	1735.09
1634.13	1729.81
1634.55	1765.66
1634.97	1750.74
1635.39	1748.63
1635.81	1757.21
1636.24	1755.22

1636.66	1742.14
1637.08	1752.21
1637.5	1775.73
1637.92	1757.1
1638.34	1744.38
1638.76	1764.22
1639.18	1744.68
1639.6	1721.15
1640.02	1738.62
1640.44	1740.75
1640.86	1743.27
1641.28	1716.29
1641.7	1754.46
1642.12	1733.05
1642.54	1730.19
1642.96	1755.05
1643.38	1763.5
1643.8	1764.8
1644.22	1745.8
1644.64	1747.54
1645.06	1755.62
1645.49	1754.29
1645.91	1762.91
1646.32	1738.93
1646.75	1725.8
1647.17	1734.26
1647.58	1733.26

1648	1744.99
1648.43	1748.31
1648.84	1757.75
1649.26	1770.25
1649.69	1736.27
1650.1	1767.79
1650.52	1759.06
1650.94	1752.75
1651.36	1745.38
1651.78	1769.53
1652.2	1757.36
1652.62	1756.2
1653.04	1744.21
1653.46	1768.34
1653.88	1772.64
1654.3	1744.77
1654.72	1747.06
1655.14	1781.32
1655.56	1738.34
1655.98	1746.23
1656.4	1765.79
1656.82	1760.26
1657.24	1733.09
1657.66	1755.33
1658.07	1754.29
1658.49	1771.6
1658.91	1774.85

1659.33	1766.43
1659.75	1765.61
1660.17	1741.41
1660.59	1757.21
1661.01	1774.44
1661.43	1765.14
1661.85	1776.94
1662.26	1745.3
1662.68	1780.71
1663.1	1771.48
1663.52	1762.85
1663.94	1761.81
1664.36	1797.34
1664.78	1755.34
1665.2	1761.48
1665.61	1751.3
1666.03	1756.21
1666.45	1791.49
1666.87	1773.96
1667.29	1763.54
1667.71	1783.35
1668.13	1779.03
1668.54	1755.59
1668.96	1765.69
1669.38	1787.58
1669.8	1783.35
1670.22	1765.77

1670.64	1765.66
1671.05	1773.06
1671.47	1752.34
1671.89	1797.24
1672.31	1763.34
1672.73	1770.02
1673.14	1765.18
1673.56	1781.85
1673.98	1807.61
1674.4	1785.96
1674.82	1791.28
1675.23	1771.78
1675.65	1787.59
1676.07	1784.91
1676.49	1786.15
1676.91	1774.22
1677.32	1789.25
1677.74	1789.79
1678.16	1791.02
1678.58	1788.22
1678.99	1774.77
1679.41	1774.27
1679.83	1796.66
1680.25	1795.48
1680.66	1762.82
1681.08	1791.52
1681.5	1768.9

1681.92	1778.94
1682.33	1793.07
1682.75	1810.43
1683.17	1791.44
1683.58	1732.91
1684	1782.87
1684.42	1755.98
1684.84	1743.56
1685.26	1780.48
1685.67	1810.65
1686.09	1767.27
1686.51	1783.88
1686.92	1771.28
1687.34	1798.9
1687.76	1806.28
1688.17	1771.54
1688.59	1763.05
1689.01	1780.85
1689.42	1795.39
1689.84	1808.13
1690.26	1799.61
1690.67	1797.18
1691.09	1785.49
1691.51	1772.88
1691.92	1792.59
1692.34	1760.5
1692.76	1808.18

1693.17	1790.66
1693.59	1774.08
1694.01	1777.13
1694.42	1767.16
1694.84	1773.97
1695.26	1771.49
1695.67	1778.33
1696.09	1786
1696.51	1780.94
1696.92	1780.01
1697.34	1769.1
1697.76	1775.74
1698.17	1770.96
1698.59	1784.64
1699	1771.44
1699.42	1782.62
1699.84	1775.2
1700.25	1781.68
1700.67	1764.68
1701.08	1774.05
1701.5	1791.95
1701.91	1783.14
1702.33	1758.73
1702.75	1759.83
1703.16	1769.97
1703.58	1774.54
1703.99	1769.51

1704.41	1764.25
1704.83	1756.1
1705.24	1760.66
1705.66	1763.13
1706.07	1769.51
1706.49	1785.74
1706.9	1794.84
1707.32	1790.36
1707.73	1777.24
1708.15	1770.63
1708.57	1757.09
1708.98	1764.58
1709.4	1761.53
1709.81	1771.01
1710.23	1756.52
1710.64	1725.54
1711.06	1742.84
1711.47	1746.22
1711.89	1759.72
1712.3	1746.8
1712.72	1755.05
1713.13	1754.27
1713.55	1742.79
1713.96	1723.48
1714.38	1727.58
1714.79	1738.74
1715.21	1741.02

1715.62	1748.78
1716.04	1762.35
1716.45	1762.55
1716.87	1732.6
1717.28	1734.84
1717.7	1754.12
1718.11	1753
1718.53	1758.23
1718.94	1743.29
1719.36	1733.71
1719.77	1724.91
1720.18	1734.8
1720.6	1725.88
1721.01	1719.99
1721.43	1746.76
1721.84	1743.74
1722.26	1731.04
1722.67	1741.76
1723.09	1730.14
1723.5	1739.52
1723.91	1749.38
1724.33	1722.89
1724.74	1727.37
1725.16	1745.31
1725.57	1706.56
1725.98	1720.97
1726.4	1727.68

1726.81	1726.61
1727.23	1719.61
1727.64	1738.08
1728.05	1725.73
1728.47	1735.5
1728.88	1717.69
1729.3	1723.28
1729.71	1705.35
1730.13	1720.59
1730.54	1712.02
1730.95	1724.73
1731.37	1709.23
1731.78	1716.13
1732.19	1711.29
1732.61	1698.11
1733.02	1714.27
1733.43	1701.4
1733.85	1718.35
1734.26	1716.44
1734.67	1706.36
1735.09	1715.23
1735.5	1729.6
1735.91	1708.83
1736.33	1723.64
1736.74	1714.45
1737.16	1726.49
1737.57	1716.06

1737.98	1681.12
1738.39	1702.21
1738.81	1705.09
1739.22	1714.04
1739.63	1726.22
1740.05	1700.28
1740.46	1683.75
1740.87	1708.92
1741.28	1720.75
1741.7	1681.71
1742.11	1725
1742.52	1692.61
1742.94	1741.36
1743.35	1694.26
1743.76	1699.92
1744.18	1712.27
1744.59	1723.44
1745	1700.91
1745.41	1706.48
1745.83	1664.43
1746.24	1715.76
1746.65	1723.32
1747.06	1689.12
1747.48	1700.98
1747.89	1692.41
1748.3	1664.32
1748.71	1730.45

1749.13	1737.09
1749.54	1733.51
1749.95	1730.1
1750.36	1737.62
1750.77	1723.16
1751.19	1703.45
1751.6	1660.83
1752.01	1711.44
1752.42	1740.63
1752.84	1682.69
1753.25	1687.93
1753.66	1723.31
1754.07	1702.11
1754.48	1731.12
1754.9	1691.92
1755.31	1736.43
1755.72	1681.6
1756.13	1720.19
1756.54	1736.44
1756.95	1737.99
1757.37	1732.06
1757.78	1757.42
1758.19	1751.47
1758.6	1723.16
1759.01	1706.66
1759.42	1719.87
1759.84	1719.32

1760.25	1767.33
1760.36	1784.99
1760.77	1684.9
1761.19	1732.82
1761.6	1714.77
1762.01	1716.68
1762.43	1732.62
1762.84	1724.8
1763.26	1740.45
1763.67	1756.11
1764.09	1694.7
1764.5	1716.19
1764.91	1742.52
1765.33	1752.6
1765.74	1718.82
1766.16	1726.59
1766.57	1728.08
1766.98	1755.77
1767.4	1750.43
1767.81	1727.57
1768.23	1745.04
1768.64	1765.16
1769.05	1708.56
1769.47	1754.02
1769.88	1727.87
1770.29	1754.74
1770.71	1782.83

1771.12	1764.9
1771.54	1753.75
1771.95	1713.61
1772.36	1746.27
1772.78	1742.73
1773.19	1754.75
1773.6	1783.97
1774.02	1729.26
1774.43	1793.93
1774.84	1752.61
1775.26	1776.92
1775.67	1737.78
1776.08	1748.22
1776.5	1752.73
1776.91	1769.17
1777.33	1771.7
1777.74	1799.12
1778.15	1712.84
1778.56	1787.32
1778.98	1773.83
1779.39	1788.06
1779.8	1773.97
1780.22	1770.25
1780.63	1795.15
1781.04	1780.49
1781.45	1787.79
1781.87	1796.96

1782.28	1776.55
1782.7	1761.46
1783.11	1791.26
1783.52	1728.61
1783.93	1784.94
1784.35	1764.7
1784.76	1802.11
1785.17	1789.23
1785.58	1744.57
1786	1767.02
1786.41	1770.81
1786.82	1771.74
1787.23	1782.33
1787.65	1796.7
1788.06	1776.76
1788.47	1767.05
1788.88	1813.96
1789.3	1791.06
1789.71	1803.21
1790.12	1808.24
1790.53	1781.96
1790.95	1766.47
1791.36	1800.89
1791.77	1812.68
1792.18	1769.76
1792.59	1787.11
1793.01	1795.75

1793.42	1752.85
1793.83	1800.06
1794.24	1789.24
1794.66	1784.51
1795.07	1793.89
1795.48	1827.81
1795.89	1825.67
1796.3	1820.63
1796.71	1749.57
1797.13	1790.77
1797.54	1804.9
1797.95	1832.77
1798.36	1824.15
1798.77	1832.34
1799.18	1849.74
1799.6	1814.9
1800.01	1806.91
1800.42	1798.66
1800.83	1762.79
1801.24	1798.78
1801.65	1800.58
1802.06	1817.63
1802.48	1763.17
1802.89	1818.38
1803.3	1788.75
1803.71	1780.73
1804.12	1846.08

1804.53	1784.73
1804.94	1836.73
1805.36	1825.73
1805.77	1807.85
1806.18	1808.34
1806.59	1821.37
1807	1793.41
1807.41	1844.46
1807.82	1831.92
1808.23	1821.66
1808.64	1798.16
1809.06	1832.26
1809.46	1814.11
1809.88	1808.56
1810.29	1805.93
1810.7	1808.47
1811.11	1823.52
1811.52	1820.62
1811.93	1791.96
1812.34	1819.51
1812.75	1857.15
1813.16	1819.68
1813.57	1843.9
1813.98	1813.3
1814.39	1815.13
1814.8	1828.62
1815.21	1803.36

1815.62	1819.03
1816.04	1839.16
1816.45	1808.19
1816.86	1796.87
1817.27	1842.76
1817.68	1846.96
1818.09	1815.42
1818.5	1786.61
1818.91	1821.07
1819.32	1813.4
1819.73	1807.43
1820.14	1783.47
1820.55	1811.73
1820.96	1822.21
1821.37	1797.39
1821.78	1794.84
1822.19	1815.41
1822.6	1815.67
1823.01	1830.95
1823.42	1797.1
1823.83	1834.33
1824.24	1815.42
1824.65	1802.29
1825.06	1803.94
1825.47	1798.54
1825.88	1820.25
1826.29	1796.89

1826.69	1787.47
1827.1	1796.51
1827.51	1820.25
1827.92	1800.95
1828.33	1815.19
1828.74	1811.25
1829.15	1795.65
1829.56	1805.43
1829.97	1795.85
1830.38	1816.68
1830.79	1809.48
1831.2	1824.94
1831.61	1777.64
1832.02	1816.17
1832.43	1796.94
1832.83	1814.65
1833.25	1767.94
1833.65	1796.52
1834.06	1826.8
1834.47	1820.85
1834.88	1787.09
1835.29	1802.19
1835.7	1804.23
1836.11	1805.62
1836.52	1776.79
1836.93	1775.14
1837.33	1766.17

1837.74	1759.2
1838.15	1775.9
1838.56	1781.05
1838.97	1774.02
1839.38	1802.69
1839.79	1804.82
1840.2	1774.17
1840.6	1812.02
1841.01	1772.85
1841.42	1808.96
1841.83	1802.17
1842.24	1766.46
1842.65	1754.69
1843.05	1781.11
1843.46	1767.86
1843.87	1763.4
1844.28	1754.54
1844.69	1794.79
1845.1	1786.35
1845.51	1777.3
1845.91	1766.2
1846.32	1760.3
1846.73	1743.75
1847.14	1793.75
1847.54	1758.83
1847.95	1790.02
1848.36	1780.94

1848.77	1755.79
1849.18	1766.27
1849.58	1743.48
1849.99	1789.21
1850.4	1783.07
1850.81	1770.45
1851.22	1765.72
1851.62	1771.01
1852.03	1762.62
1852.44	1758.46
1852.85	1757.13
1853.25	1765.49
1853.66	1761.81
1854.07	1788.92
1854.48	1789.48
1854.88	1785.74
1855.29	1761.03
1855.7	1723.91
1856.11	1707.98
1856.51	1761.57
1856.92	1760.69
1857.33	1732.26
1857.74	1760.13
1858.14	1746.08
1858.55	1737.42
1858.96	1722.39
1859.37	1716.45

1859.77	1740.7
1860.18	1738.16
1860.59	1728.72
1860.99	1748.74
1861.4	1757.07
1861.81	1745.15
1862.22	1743.93
1862.62	1741.03
1863.03	1738.76
1863.44	1732.57
1863.84	1774.9
1864.25	1772.33
1864.66	1755.4
1865.06	1758.14
1865.47	1743.18
1865.88	1734.51
1866.28	1734.96
1866.69	1749.59
1867.1	1740.24
1867.5	1717.89
1867.91	1729.25
1868.32	1732.65
1868.72	1739.37
1869.13	1750.14
1869.54	1739.22
1869.94	1740.9
1870.35	1767.23

1870.75	1751.31
1871.16	1747.32
1871.57	1739.24
1871.97	1738.61
1872.38	1725.41
1872.79	1726.14
1873.19	1728.54
1873.6	1765.88
1874	1750.69
1874.41	1740.81
1874.82	1731.37
1875.22	1746.49
1875.63	1727.27
1876.03	1737.51
1876.44	1741.27
1876.85	1748.39
1877.25	1736.61
1877.66	1743.28
1878.06	1724.43
1878.47	1766.01
1878.88	1741.12
1879.28	1739.75
1879.69	1740.39
1880.09	1762.72
1880.5	1773.23
1880.9	1757.99
1881.31	1751.67

1881.72	1774.03
1882.12	1765.95
1882.53	1746.4
1882.93	1737.55
1883.34	1741.51
1883.74	1745.73
1884.15	1745.72
1884.55	1728.29
1884.96	1742.18
1885.36	1760.48
1885.77	1744.67
1886.17	1751.52
1886.58	1751.39
1886.98	1767.96
1887.39	1752.44
1887.79	1767.76
1888.2	1758.37
1888.6	1758.36
1889.01	1772.71
1889.41	1749.91
1889.82	1751.51
1890.22	1762.37
1890.63	1771.41
1891.03	1767.57
1891.44	1734.27
1891.84	1731.46
1892.25	1761.92

1892.65	1764.52
1893.06	1791.78
1893.46	1764.32
1893.87	1741.32
1894.27	1769.27
1894.67	1762.34
1895.08	1805.83
1895.48	1771.21
1895.89	1768.76
1896.29	1780.32
1896.7	1796.29
1897.1	1785.49
1897.51	1790.98
1897.91	1763.04
1898.32	1784.41
1898.72	1783.08
1899.12	1773.23
1899.53	1772.34
1899.93	1772.15
1900.34	1795.27
1900.74	1800.43
1901.15	1788.56
1901.55	1817.48
1901.95	1783.86
1902.36	1800.09
1902.76	1776.77
1903.16	1791.03

1903.57	1809.51
1903.97	1780.8
1904.38	1800.65
1904.78	1783.3
1905.18	1811.27
1905.59	1794.98
1905.99	1804.43
1906.4	1826.6
1906.8	1841.15
1907.2	1790.11
1907.61	1800.68
1908.01	1818.54
1908.41	1775.09
1908.82	1804.14
1909.22	1830.56
1909.62	1806.18
1910.03	1824.31
1910.43	1858.01
1910.83	1825.94
1911.24	1848.36
1911.64	1838.43
1912.04	1813.42
1912.45	1824.58
1912.85	1813.1
1913.25	1859.88
1913.66	1822.97
1914.06	1864.31

1914.46	1834.51
1914.87	1836.96
1915.27	1827.57
1915.67	1837.87
1916.07	1833.56
1916.48	1821.12
1916.88	1843.87
1917.28	1835.8
1917.69	1872.65
1918.09	1870.48
1918.49	1840.92
1918.89	1855.18
1919.3	1848.75
1919.7	1829.36
1920.1	1834.9
1920.5	1824.65
1920.91	1836.72
1921.31	1855
1921.71	1844.71
1922.12	1867.79
1922.52	1851.96
1922.92	1872.27
1923.32	1844.96
1923.73	1870.42
1924.13	1874.47
1924.53	1849.53
1924.93	1863.84

1925.33	1844.15
1925.74	1829.85
1926.14	1862.37
1926.54	1861.28
1926.94	1878.32
1927.35	1844.22
1927.75	1823.34
1928.15	1875.76
1928.55	1886.23
1928.95	1857.27
1929.36	1849.76
1929.76	1875.45
1930.16	1848.76
1930.56	1850.19
1930.96	1865.92
1931.36	1851.99
1931.77	1838.15
1932.17	1876.48
1932.57	1861.87
1932.97	1859.08
1933.37	1856.49
1933.78	1880.05
1934.18	1872.62
1934.58	1860
1934.98	1865.92
1935.38	1879.56
1935.78	1893.52

1936.18	1870.47
1936.59	1855.44
1936.99	1865.97
1937.39	1873.06
1937.79	1898.73
1938.19	1876.62
1938.59	1871.97
1938.99	1872.37
1939.4	1862.1
1939.8	1840.16
1940.2	1846.69
1940.6	1828.41
1941	1853.1
1941.4	1861.89
1941.8	1881.25
1942.2	1881.45
1942.6	1869.24
1943.01	1894.1
1943.41	1888.08
1943.81	1867.64
1944.21	1901.16
1944.61	1863.47
1945.01	1876.25
1945.41	1880.74
1945.81	1869.82
1946.21	1880.31
1946.61	1871.94

1947.01	1865.49
1947.41	1876.64
1947.81	1864.04
1948.22	1857.58
1948.62	1854.42
1949.02	1846.8
1949.42	1866.4
1949.82	1828.76
1950.22	1857.86
1950.62	1849.34
1951.02	1863.71
1951.42	1874.03
1951.82	1870.31
1952.22	1872.56
1952.62	1855.73
1953.02	1846.12
1953.42	1838.4
1953.82	1839.6
1954.22	1840.69
1954.62	1837.76
1955.02	1826.5
1955.42	1849.43
1955.82	1887.52
1956.22	1853.22
1956.62	1835.39
1957.02	1843.31
1957.42	1872.91

1957.82	1850.54
1958.22	1841.13
1958.62	1865.41
1959.02	1840.93
1959.42	1849.6
1959.82	1859.19
1960.22	1859.21
1960.62	1806.22
1961.02	1834.72
1961.42	1835.48
1961.82	1797.48
1962.22	1841.45
1962.62	1800.07
1963.02	1835.14
1963.42	1775.63
1963.81	1806.68
1964.21	1803.92
1964.61	1847.16
1965.01	1847.82
1965.41	1828.98
1965.81	1849.48
1966.21	1837.26
1966.61	1786.56
1967.01	1790.76
1967.41	1793.52
1967.81	1827.16
1968.21	1785.63

1968.61	1790.87
1969	1781.62
1969.4	1774.66
1969.46	1773.87
1969.86	1802.67
1970.26	1735.15
1970.66	1769.04
1971.07	1812.32
1971.47	1751.19
1971.87	1744.35
1972.27	1790.3
1972.67	1821.31
1973.07	1781.15
1973.48	1797.26
1973.88	1756.95
1974.28	1791.2
1974.68	1781.9
1975.08	1771.42
1975.48	1799.57
1975.89	1754.95
1976.29	1793.24
1976.69	1738.14
1977.09	1719.01
1977.49	1736.33
1977.89	1726.59
1978.29	1711.01
1978.7	1757.04

1979.1	1768.57
1979.5	1748.93
1979.9	1722.39
1980.3	1730.77
1980.7	1745.02
1981.1	1743.09
1981.5	1751.72
1981.9	1728.36
1982.3	1711.18
1982.71	1740.36
1983.11	1738.17
1983.51	1730.48
1983.91	1728.05
1984.31	1735.39
1984.71	1781.99
1985.11	1734.15
1985.51	1733.48
1985.91	1716.15
1986.31	1703.98
1986.71	1729.27
1987.11	1733.69
1987.51	1700.44
1987.92	1702.36
1988.32	1711.73
1988.72	1741.66
1989.12	1700.32
1989.52	1741.01

1989.92	1725.9
1990.32	1732.22
1990.72	1730.91
1991.12	1678.26
1991.52	1702.68
1991.92	1721.19
1992.32	1705.69
1992.72	1703.95
1993.12	1702.16
1993.52	1711.94
1993.92	1709.83
1994.32	1679.07
1994.72	1701.22
1995.12	1719.83
1995.52	1714.8
1995.92	1716.13
1996.32	1708.92
1996.72	1733.51
1997.12	1721.01
1997.52	1696.11
1997.92	1704.98
1998.32	1691.81
1998.72	1677.4
1999.12	1697.71
1999.52	1718.45
1999.92	1698.5

100.25	29
100.77	34.9987
101.289	28.9974
101.809	38.9961
102.329	32.9948
102.847	30.9935
103.366	32.9922
103.886	32.9909
104.406	25.4623
104.925	36.512
105.445	37.707
105.964	34.7739
106.484	29.2822
107.001	31.0736
107.521	34.1076
108.04	32.8033
108.56	31.4072
109.079	32.7203
109.598	33.433
110.116	30.1912
110.635	28.7191
111.154	34.3583
111.674	27.1728
112.193	30.6096
112.71	33.0633
113.229	33.8183

113.748	31.1008
114.267	32.1716
114.784	32.5249
115.303	30.5439
115.822	32.9528
116.341	33.3699
116.858	32.3644
117.377	31.5844
117.896	29.9341
118.415	29.4248
118.931	32.8811
119.45	33.3128
119.969	32.3985
120.486	32.7405
121.004	32.7293
121.523	34.8648
122.042	31.6427
122.558	30.7523
123.077	29.8533
123.596	27.5202
124.112	32.9913
124.63	30.797
125.149	34.8826
125.665	32.5436
126.184	29.2954
126.702	29.3393
127.218	30.7699

127.737	31.1474
128.253	35.1318
128.771	30.4761
129.29	30.7513
129.806	33.6102
130.324	33.4538
130.84	30.4812
131.358	29.0672
131.876	27.7539
132.392	30.7272
132.91	34.9253
133.426	31.8585
133.944	32.2051
134.46	30.0235
134.978	30.9696
135.496	29.2486
136.012	28.861
136.53	30.5479
137.045	34.1683
137.563	34.6404
138.079	26.9845
138.597	31.7259
139.112	28.6582
139.63	27.3121
140.145	31.0082
140.663	33.5812
141.178	33.3475

141.696	31.7824
142.211	31.2633
142.729	29.5144
143.244	33.126
143.76	32.294
144.277	33.7092
144.792	27.9862
145.31	30.942
145.825	29.0277
146.342	29.4908
146.858	34.9572
147.375	31.3126
147.89	29.1479
148.405	28.8027
148.922	30.798
149.437	29.3118
149.954	31.2492
150.469	29.4315
150.984	34.2412
151.501	32.4625
152.016	34.0463
152.531	33.0373
153.048	29.34
153.563	30.9395
154.078	34.6205
154.594	30.0772
155.109	31.0561

155.624	31.6195
156.141	31.9792
156.655	33.4977
157.17	32.1653
157.687	30.1678
158.201	28.2621
158.716	34.715
159.232	32.4162
159.747	32.8747
160.261	31.1528
160.776	31.968
161.292	31.0894
161.806	28.6033
162.321	37.0414
162.835	33.8065
163.351	32.3524
163.866	32.0773
164.38	30.9192
164.894	30.5848
165.41	31.9727
165.924	29.133
166.438	31.6112
166.953	30.4075
167.467	30.2095
167.983	29.7938
168.497	32.2485
169.011	31.7963

169.525	32.5533
170.038	28.0823
170.554	26.3266
171.068	31.0352
171.582	35.6385
172.096	31.5343
172.61	29.4744
173.123	31.2623
173.637	31.0848
174.153	29.6584
174.666	30.0927
175.18	30.9926
175.694	32.2597
176.207	28.0505
176.721	30.3209
177.234	31.8554
177.748	30.9851
178.261	29.834
178.777	35.8603
179.29	31.3141
179.803	30.5429
180.317	30.1585
180.83	31.5792
181.343	32.7452
181.856	27.8551
182.37	31.3435
182.883	30.535

183.396	27.6133
183.909	31.3559
184.422	29.7937
184.935	31.0371
185.448	31.1056
185.961	32.3013
186.474	32.1172
186.987	29.6644
187.5	28.367
188.013	25.5536
188.526	27.5452
189.039	29.3563
189.552	30.7814
190.064	30.1362
190.577	33.5858
191.09	27.7172
191.603	33.1429
192.115	30.2879
192.628	24.7647
193.138	30.5535
193.651	32.5084
194.164	31.613
194.676	36.361
195.189	32.336
195.701	30.5955
196.214	28.3788
196.726	33.4357

197.238	33.4349
197.749	29.6233
198.261	35.4548
198.773	33.475
199.286	34.2839
199.798	31.4526
200.31	35.4718
200.822	28.4959
201.332	31.3759
201.844	33.5661
202.357	27.5879
202.869	31.3305
203.381	26.4765
203.893	32.7586
204.403	35.9151
204.915	31.6068
205.427	33.5974
205.938	30.5803
206.448	37.8791
206.96	27.6895
207.472	30.7175
207.984	30.3118
208.493	33.8135
209.005	30.1571
209.517	31.1249
210.029	33.4516
210.538	35.9231

211.05	33.942
211.561	30.9977
212.073	35.6203
212.582	30.5714
213.094	33.4453
213.605	28.8104
214.115	30.2237
214.626	33.7717
215.138	33.0871
215.647	28.3857
216.158	35.2539
216.669	32.6845
217.179	27.912
217.69	29.9351
218.201	34.3817
218.71	30.4132
219.221	32.1576
219.733	31.3969
220.242	32.8215
220.753	31.8656
221.262	31.1774
221.773	33.1722
222.284	32.1156
222.793	33.0895
223.304	32.3737
223.812	32.721
224.323	31.7018

224.834	27.7118
225.343	28.4292
225.854	31.3662
226.362	32.3689
226.873	31.9884
227.382	29.1902
227.893	27.6489
228.401	29.7814
228.912	34.9035
229.422	33.3478
229.931	33.9439
230.441	34.1607
230.95	30.7608
231.46	28.764
231.969	31.8505
232.479	30.8669
232.987	32.5978
233.496	33.0758
234.006	31.584
234.514	27.3866
235.025	32.4786
235.533	28.2956
236.043	26.3756
236.551	28.8846
237.061	26.4023
237.57	31.9089
238.08	30.8402

238.588	29.8543
239.096	34.2902
239.606	34.8146
240.114	32.3568
240.624	33.2634
241.132	30.7984
241.639	28.5898
242.149	29.7032
242.657	29.4995
243.165	30.7043
243.675	31.0462
244.182	29.9168
244.692	33.5597
245.2	33.1094
245.707	34.331
246.217	27.4982
246.725	31.8904
247.232	34.4904
247.742	28.4772
248.249	31.461
248.757	30.9479
249.264	33.5566
249.774	35.6541
250.281	34.4541
250.788	27.0384
251.298	33.1795
251.805	30.5622

252.312	32.6815
252.82	33.4358
253.329	32.3146
253.836	29.8805
254.343	28.6652
254.85	28.3336
255.36	31.5169
255.867	31.4453
256.374	31.4469
256.881	29.9632
257.388	29.5655
257.897	32.3455
258.404	35.1499
258.911	34.1692
259.418	31.5365
259.924	33.3631
260.433	30.6561
260.94	31.4613
261.447	32.53
261.954	32.4092
262.46	31.4789
262.967	25.3951
263.476	32.8314
263.982	31.7116
264.489	31.4653
264.996	29.4741
265.502	30.3508

266.009	27.9037
266.515	30.4348
267.022	32.6215
267.528	32.6803
268.036	30.2283
268.543	30.0504
269.049	30.9329
269.555	30.8071
270.062	28.9774
270.568	34.2926
271.074	28.5008
271.58	34.212
272.087	35.636
272.593	33.3865
273.099	33.6156
273.605	32.9041
274.111	33.5045
274.617	28.9723
275.123	33.5061
275.629	34.8141
276.135	32.3099
276.641	35.2661
277.147	27.6211
277.653	30.9197
278.159	32.7951
278.665	30.825
279.17	28.8793

279.676	34.8664
280.182	32.8775
280.688	38.2254
281.193	35.5421
281.699	29.594
282.204	33.45
282.71	34.1034
283.216	32.1586
283.721	25.05
284.225	33.2649
284.73	33.7184
285.236	35.511
285.741	33.0636
286.246	37.2742
286.752	34.5056
287.257	32.3542
287.762	32.3637
288.266	28.5023
288.771	35.9039
289.276	38.6327
289.781	33.3359
290.287	34.4185
290.792	36.7938
291.295	33.4177
291.8	31.7786
292.305	29.0184
292.81	34.9278

293.315	31.3954
293.82	31.3142
294.323	32.648
294.828	37.3881
295.333	32.825
295.838	33.1629
296.34	34.0003
296.845	32.8509
297.35	33.6988
297.855	34.4282
298.358	31.9302
298.862	31.0771
299.367	33.3405
299.872	33.5971
300.374	34.1076
300.879	31.2852
301.383	31.046
301.888	30.6917
302.39	31.116
302.895	32.8128
303.399	34.9893
303.902	27.3315
304.406	29.099
304.91	30.6571
305.413	30.5597
305.917	31.5444
306.421	32.9444

306.924	33.518
307.428	34.7691
307.932	33.2738
308.434	33.0445
308.938	32.762
309.44	30.2334
309.944	32.6585
310.449	33.205
310.951	33.3508
311.455	37.1307
311.957	32.0726
312.46	33.1058
312.964	33.924
313.466	32.3574
313.97	30.9368
314.472	32.5375
314.976	32.7337
315.478	33.2756
315.981	32.1522
316.483	32.0309
316.987	28.6709
317.488	31.0078
317.992	33.7246
318.496	32.9423
318.997	33.1587
319.501	32.5365
320.002	30.5335

320.506	31.3565
321.007	34.6011
321.509	31.5273
322.012	31.8641
322.513	33.8522
323.017	31.6604
323.518	30.2873
324.021	27.1733
324.523	24.242
325.026	30.9955
325.527	32.3836
326.03	30.8319
326.532	29.0381
327.033	32.704
327.536	31.3943
328.037	31.2874
328.54	28.7445
329.041	27.6801
329.542	30.3836
330.045	33.6834
330.546	31.556
331.047	31.7552
331.55	34.2699
332.051	29.098
332.553	28.4166
333.054	28.2662
333.555	30.4884

334.058	31.9265
334.558	30.1659
335.059	27.2559
335.562	30.3603
336.062	33.4029
336.563	32.1253
337.064	32.4
337.566	33.9867
338.067	33.9117
338.567	31.0069
339.07	34.1599
339.57	33.0431
340.07	28.569
340.571	32.5425
341.073	35.7756
341.574	37.7214
342.074	35.3732
342.574	30.7917
343.076	33.4081
343.577	33.4952
344.077	31.1877
344.577	34.7936
345.077	31.4197
345.579	31.0719
346.079	35.1532
346.579	32.1464
347.079	33.3565

347.579	32.5983
348.081	26.9809
348.581	35.3961
349.081	33.6287
349.581	35.7479
350.081	34.852
350.581	30.2125
351.081	33.7529
351.582	33.8469
352.082	33.3962
352.582	28.196
353.082	31.679
353.581	31.6504
354.081	29.8584
354.58	31.8706
355.08	35.6228
355.58	31.8849
356.081	33.5436
356.581	30.1436
357.08	37.5288
357.58	29.9874
358.079	33.6937
358.578	35.7244
359.078	35.7127
359.577	31.9212
360.076	29.8573
360.576	25.9796

361.075	31.5415
361.574	37.4895
362.073	33.9926
362.573	37.5273
363.072	34.059
363.105	33.7933
363.608	31.7924
364.109	25.7975
364.612	27.8047
365.114	29.817
365.617	31.7868
366.118	37.7117
366.621	25.8731
367.124	35.7407
367.625	29.8201
368.128	35.7295
368.629	29.8645
369.132	25.9342
369.633	31.6576
370.136	27.7582
370.636	29.8381
371.139	31.7561
371.64	28.0702
372.143	29.7754
372.643	31.5303
373.146	33.5714
373.647	21.9419

374.15	31.5629
374.65	27.8831
375.153	26.1307
375.653	31.6964
376.154	29.6932
376.656	31.5093
377.157	35.7805
377.659	31.4763
378.16	33.5773
378.662	32.2438
379.162	33.6731
379.663	37.1838
380.165	33.5386
380.665	35.4023
381.168	31.7883
381.668	35.9166
382.168	39.3779
382.67	37.5009
383.17	35.3577
383.671	33.6301
384.173	34.2251
384.673	37.3515
385.173	37.1449
385.675	30.2587
386.175	32.1354
386.675	33.8069
387.177	39.0878

387.677	31.7911
388.176	37.3958
388.678	33.278
389.178	35.5284
389.678	31.9903
390.18	36.853
390.679	38.3736
391.179	37.1827
391.679	37.1153
392.18	38.9168
392.68	30.2013
393.18	39.1121
393.679	38.5265
394.181	31.7105
394.68	32.181
395.18	40.0807
395.679	33.6017
396.181	37.0915
396.68	35.8911
397.179	32.1558
397.679	35.9387
398.178	37.5849
398.679	37.7304
399.179	39.5183
399.678	39.5144
400.177	41.23
400.676	26.6451

401.175	30.4323
401.677	32.3751
402.176	34.0869
402.675	37.2737
403.174	30.617
403.673	28.8018
404.172	35.2591
404.671	32.2328
405.172	35.1211
405.671	37.17
406.169	33.4439
406.668	29.5659
407.167	31.7992
407.666	38.3232
408.165	36.1946
408.663	29.6717
409.162	32.3513
409.661	35.6893
410.162	35.8925
410.66	32.4164
411.159	28.8876
411.657	31.9301
412.156	30.1642
412.654	32.0212
413.153	38.1867
413.651	37.6359
414.15	36.5381

414.648	33.5607
415.147	31.7908
415.645	34.7603
416.143	35.0372
416.642	35.8428
417.14	35.7435
417.638	32.6653
418.136	32.1348
418.635	30.0191
419.133	33.591
419.631	33.3363
420.129	26.8886
420.627	28.4421
421.123	34.6861
421.621	27.3554
422.119	32.0179
422.617	29.5774
423.115	30.4154
423.613	27.7697
424.111	31.3245
424.609	36.5694
425.107	32.0832
425.605	36.3161
426.102	28.5841
426.598	29.9464
427.096	28.8317
427.594	37.1113

428.091	32.5112
428.589	34.2877
429.086	29.7906
429.584	26.808
430.08	32.5608
430.577	27.9946
431.075	35.9119
431.572	28.4376
432.07	31.1342
432.567	28.4559
433.062	36.9666
433.56	32.6712
434.057	24.911
434.555	28.8852
435.052	29.7724
435.547	28.8652
436.044	25.4605
436.542	29.479
437.039	28.2392
437.534	31.1467
438.031	33.3908
438.528	31.8475
439.025	29.3806
439.52	28.8829
440.017	34.3802
440.514	27.6133
441.011	31.0159

441.506	33.0303
442.003	29.9472
442.5	37.3531
442.995	32.8743
443.492	29.024
443.989	28.6941
444.483	33.4326
444.98	29.5108
445.477	31.2602
445.971	27.4209
446.468	30.8864
446.965	27.5357
447.459	31.9149
447.956	29.5042
448.453	29.6714
448.947	34.6062
449.444	31.3505
449.938	33.1474
450.435	28.854
450.931	31.2177
451.425	31.6922
451.922	25.8383
452.416	29.9708
452.912	33.9661
453.409	34.5786
453.903	30.7009
454.399	35.0878

454.893	29.8352
455.39	31.5094
455.884	24.1129
456.38	37.575
456.874	30.6241
457.37	34.354
457.864	31.0606
458.36	36.1244
458.856	30.9679
459.35	28.3813
459.846	31.045
460.34	29.7065
460.834	28.845
461.33	29.7897
461.824	28.5498
462.319	29.7896
462.813	32.2538
463.309	28.5614
463.803	31.3281
464.298	30.7101
464.792	28.9901
465.288	32.5185
465.781	36.2073
466.277	33.3532
466.77	29.1221
467.264	30.184
467.759	27.3595

468.253	34.0207
468.748	31.9503
469.242	27.9106
469.735	29.3909
470.231	26.547
470.724	31.3
471.217	29.3707
471.713	31.5308
472.206	26.8177
472.701	31.5286
473.194	32.8419
473.687	33.7892
474.183	27.7828
474.676	28.2886
475.169	26.0453
475.664	31.3924
476.157	29.5338
476.65	34.0367
477.143	32.8531
477.638	28.2322
478.131	31.484
478.624	29.5725
479.119	32.547
479.612	33.6076
480.104	32.3122
480.597	31.7974
481.092	31.611

481.585	31.6654
482.078	28.539
482.57	30.9555
483.065	32.5594
483.558	27.6496
484.05	29.4179
484.543	29.3448
485.035	30.9628
485.53	28.8429
486.022	32.9914
486.515	30.0267
487.007	31.6225
487.5	29.7535
487.994	29.2842
488.487	29.3645
488.979	34.5135
489.471	27.3781
489.964	31.1321
490.456	29.4253
490.95	30.4869
491.442	30.4518
491.934	30.7923
492.427	31.0694
492.919	35.7807
493.411	32.7961
493.903	30.5575
494.395	34.7109

494.887	28.9114
495.381	30.7903
495.873	30.0038
496.365	29.256
496.857	31.6832
497.349	34.5522
497.841	33.4878
498.333	30.1493
498.824	29.5515
499.316	31.8964
499.808	33.2109
500.3	32.5193
500.791	30.2246
501.283	28.8576
501.775	31.4647
502.266	33.5711
502.758	31.2741
503.25	33.2431
503.741	29.0548
504.233	32.695
504.724	32.1609
505.216	28.6657
505.707	31.9155
506.198	30.9803
506.69	31.5633
507.181	33.4541
507.673	32.7829

508.164	32.0369
508.655	30.9153
509.146	31.7876
509.638	32.1709
510.129	29.7875
510.62	32.8305
511.109	31.3249
511.6	29.8514
512.091	34.2388
512.582	29.8485
513.074	29.2748
513.565	32.2241
514.056	32.1587
514.547	35.2851
515.035	28.8441
515.526	28.6565
516.017	32.5343
516.508	33.3939
516.999	31.3773
517.49	31.7575
517.979	33.3905
518.469	31.5348
518.96	31.1054
519.451	33.3242
519.941	29.7175
520.432	33.5117
520.921	30.0161

521.411	31.0151
521.902	31.4559
522.392	32.4044
522.881	33.7547
523.371	30.543
523.862	33.2556
524.352	36.4486
524.841	32.1861
525.331	30.29
525.821	31.8114
526.312	31.0533
526.8	31.2877
527.29	32.852
527.781	30.5079
528.269	32.4056
528.759	30.9315
529.249	32.1809
529.739	27.3693
530.227	34.4884
530.718	31.9917
531.208	30.1747
531.696	32.0372
532.186	29.6368
532.676	33.8981
533.164	28.9176
533.654	32.1043
534.141	30.1582

534.631	28.7287
535.121	28.7346
535.609	28.0084
536.099	30.674
536.589	32.4822
537.076	31.557
537.566	27.321
538.054	31.2242
538.543	32.8564
539.033	26.4182
539.521	28.4136
540.01	29.9903
540.498	35.7634
540.987	30.5051
541.475	35.0865
541.964	27.0386
542.452	32.3909
542.941	35.5843
543.429	29.2225
543.918	28.9963
544.405	32.3054
544.895	27.8671
545.382	29.7326
545.871	33.6678
546.359	34.9342
546.848	33.7852
547.335	32.7965

547.824	28.443
548.311	31.2276
548.8	33.7851
549.287	32.462
549.777	37.0672
550.264	30.2822
550.753	32.5625
551.24	33.8508
551.727	35.4813
552.215	32.0376
552.702	31.9799
553.191	35.9015
553.678	31.181
554.167	31.2721
554.654	33.2815
555.141	27.2557
555.629	31.7954
556.116	31.2874
556.603	32.2777
557.091	31.2951
557.578	30.564
558.065	33.1695
558.553	31.8733
559.04	28.5177
559.526	34.0773
560.015	31.7972
560.501	29.314

560.988	32.931
561.476	32.0831
561.963	32.9429
562.449	33.784
562.937	32.2764
563.424	36.0475
563.91	29.6889
564.396	31.9948
564.885	32.9289
565.371	32.6764
565.857	28.0592
566.345	25.9316
566.831	35.6473
567.317	33.2703
567.803	30.8518
568.289	31.7836
568.778	34.171
569.264	30.5731
569.75	29.5997
570.236	30.0456
570.723	31.7834
571.209	32.4255
571.695	31.3934
572.181	32.8943
572.667	33.5216
573.153	30.0782
573.641	28.4441

574.126	31.763
574.612	31.1603
575.098	30.1293
575.583	27.8984
576.069	30.2766
576.555	29.3511
577.042	28.6175
577.528	34.9996
578.013	34.8873
578.499	31.1444
578.984	34.4351
579.47	33.3596
579.955	37.0004
580.441	38.2221
580.926	31.7966
581.411	32.2855
581.897	33.7884
582.384	32.9178
582.869	34.5977
583.355	34.38
583.84	33.1244
584.325	30.8731
584.81	33.1542
585.295	32.6494
585.781	32.1079
586.266	37.751
586.751	36.7925

587.236	31.9008
587.721	28.672
588.206	32.2478
588.691	31.4616
589.176	30.162
589.661	32.6009
590.146	30.0172
590.63	36.426
591.115	36.7859
591.598	32.9538
592.083	35.6093
592.568	33.4772
593.052	37.5634
593.537	32.3892
594.022	29.0563
594.506	27.7262
594.991	36.7747
595.476	31.5975
595.96	32.325
596.445	35.2034
596.929	38.4835
597.412	39.2477
597.896	34.4405
598.381	35.1388
598.865	36.0985
599.35	35.2995
599.834	31.7534

600.318	33.4149
600.801	29.9249
601.285	34.9513
601.769	33.0931
602.254	30.3121
602.738	31.5174
603.22	30.6474
603.704	33.18
604.188	31.0651
604.672	35.6573
605.156	33.6739
605.639	33.143
606.123	27.5725
606.607	27.1316
607.091	32.8175
607.573	36.9814
608.057	34.0345
608.54	35.4628
609.024	35.6291
609.506	35.4923
609.99	33.9206
610.474	35.3883
610.958	33.8397
611.439	33.7236
611.923	34.2646
612.407	39.2716
612.888	33.9947

613.372	37.2918
613.856	32.0076
614.337	35.5186
614.821	31.534
615.305	25.9771
615.786	29.7856
616.269	27.9123
616.753	31.8722
617.234	35.7637
617.718	39.7002
618.199	27.8307
618.201	27.7788
618.687	33.7786
619.174	35.7783
619.659	33.778
620.145	37.7613
620.63	37.7499
621.115	31.7871
621.602	29.8334
622.088	37.7314
622.573	31.736
623.058	27.8853
623.543	35.7229
624.028	29.8226
624.513	31.6956
625	27.8988
625.485	31.7764

625.97	33.8792
626.455	37.6812
626.94	31.6863
627.425	29.8254
627.91	31.6727
628.395	35.6402
628.88	31.7602
629.365	35.5169
629.852	31.9291
630.336	35.6237
630.821	31.8665
631.306	35.5952
631.79	33.6744
632.275	29.7888
632.76	33.5994
633.244	33.8025
633.729	33.8672
634.214	31.6831
634.698	28.0633
635.183	39.5109
635.667	29.9576
636.151	32.2103
636.636	36.0508
637.12	35.7522
637.605	35.2388
638.089	32.0787
638.573	37.3346

639.058	30.0981
639.542	31.6508
640.026	35.7778
640.51	35.2908
640.995	39.2731
641.479	28.5001
641.963	38.7086
642.445	37.796
642.929	32.3691
643.413	32.3813
643.897	38.816
644.381	31.7931
644.865	28.0102
645.349	35.9806
645.833	33.8141
646.317	32.2256
646.801	33.3438
647.283	33.1854
647.767	36.169
648.25	32.301
648.734	44.168
649.218	28.5066
649.702	38.3882
650.185	28.4826
650.667	35.7749
651.151	39.5157
651.634	34.0926

652.118	31.8634
652.602	32.0385
653.083	27.6092
653.567	29.9919
654.05	35.6813
654.534	40.218
655.017	35.6707
655.499	37.7564
655.982	34.1514
656.465	30.4588
656.949	28.7198
657.43	30.6744
657.913	29.9834
658.397	30.5682
658.88	33.1027
659.361	30.2499
659.844	38.5616
660.327	35.9202
660.81	32.5558
661.292	38.0841
661.775	36.0844
662.258	33.8157
662.739	27.3719
663.222	33.2532
663.705	35.1825
664.186	36.7154
664.669	32.0743

665.152	37.3317
665.633	32.8277
666.115	37.2718
666.598	33.2719
667.079	30.8796
667.562	34.8615
668.045	33.1224
668.525	36.2093
669.008	39.9467
669.489	38.0863
669.971	32.5039
670.454	30.9124
670.935	33.9597
671.417	33.8108
671.898	31.9692
672.38	34.142
672.861	31.5777
673.343	37.739
673.826	37.7165
674.306	38.3827
674.788	33.093
675.269	31.3575
675.751	38.3208
676.231	30.6689
676.714	36.4131
677.194	34.6075
677.676	36.18

678.156	39.9472
678.639	30.2078
679.119	35.1211
679.601	30.0818
680.081	34.355
680.563	30.8589
681.043	36.308
681.525	31.2998
682.005	35.265
682.487	35.755
682.967	35.0315
683.449	39.9812
683.929	36.0666
684.409	34.6244
684.891	33.1029
685.371	32.1584
685.852	31.7659
686.332	36.238
686.814	31.7518
687.294	29.9618
687.773	30.8556
688.255	31.8157
688.735	32.2846
689.216	30.0914
689.696	34.6208
690.176	32.4023
690.657	33.7213

691.137	34.5116
691.616	34.1211
692.098	32.4027
692.577	33.9126
693.057	35.0304
693.538	32.3932
694.017	35.831
694.497	33.2368
694.978	33.0593
695.457	34.0922
695.937	31.9107
696.416	32.8394
696.897	34.0976
697.376	30.351
697.855	29.8979
698.336	28.9075
698.816	29.2633
699.295	31.1029
699.774	29.0092
700.255	31.7957
700.734	31.9803
701.213	32.1489
701.692	31.16
702.173	30.8387
702.652	30.1388
703.131	32.6587
703.609	32.7317

704.088	35.2023
704.569	34.2808
705.048	28.6609
705.527	34.4122
706.005	34.4043
706.484	33.2392
706.963	31.2469
707.443	30.8791
707.922	30.74
708.401	28.7232
708.879	32.548
709.358	31.0678
709.836	31.287
710.315	35.2727
710.795	32.21
711.274	30.9107
711.752	29.3265
712.231	34.8907
712.709	27.0252
713.187	28.6724
713.666	35.8468
714.144	35.4967
714.622	29.3727
715.101	30.7093
715.579	33.2845
716.059	31.9157
716.537	29.6922

717.015	31.7778
717.494	31.0826
717.972	33.8472
718.45	35.6997
718.928	30.6257
719.406	30.6612
719.884	29.9113
720.362	33.8136
720.84	29.4745
721.318	28.7992
721.796	31.8377
722.273	29.1616
722.751	27.8798
723.229	32.2497
723.707	33.5213
724.185	32.7117
724.662	27.1275
725.138	28.5734
725.616	31.7586
726.094	31.8162
726.571	30.375
727.049	30.7723
727.526	32.1289
728.004	28.3945
728.482	31.8157
728.959	28.8079
729.437	28.4103

729.914	29.3209
730.392	32.7228
730.867	34.1172
731.344	32.8998
731.822	31.5168
732.299	33.0334
732.776	31.8148
733.254	33.6601
733.731	25.3859
734.206	31.3023
734.683	34.4051
735.161	32.1756
735.638	31.3389
736.115	33.0403
736.59	27.058
737.067	31.1812
737.544	31.8135
738.021	31.3449
738.498	26.6041
738.973	35.7238
739.45	32.0929
739.927	30.5463
740.404	30.8028
740.879	31.2526
741.356	31.9235
741.833	29.2507
742.31	29.6583

742.784	30.207
743.261	29.3819
743.738	24.124
744.215	28.1538
744.689	29.76
745.166	27.3946
745.643	28.7816
746.117	29.1262
746.594	32.4586
747.07	27.646
747.545	27.151
748.022	30.7567
748.498	32.086
748.973	31.2711
749.449	31.5299
749.925	31.8531
750.4	32.4447
750.876	27.8087
751.352	29.7322
751.827	31.629
752.303	30.1524
752.779	29.8342
753.254	29.7585
753.73	30.1547
754.204	27.4064
754.68	27.2
755.154	27.0017

755.63	29.8065
756.107	28.6258
756.581	29.6
757.057	29.8058
757.531	29.2326
758.007	28.3932
758.481	26.9486
758.957	28.0775
759.431	28.0571
759.906	29.857
760.382	28.3485
760.856	28.9633
761.332	28.436
761.806	30.515
762.282	31.2831
762.755	28.466
763.229	28.1195
763.705	30.256
764.178	32.004
764.654	29.482
765.128	28.1001
765.603	29.3237
766.077	29.3955
766.552	27.8007
767.026	26.2217
767.501	26.351
767.975	31.1267

768.448	33.372
768.924	32.1404
769.397	31.2606
769.873	28.59
770.346	29.1019
770.819	29.3961
771.295	29.3057
771.768	30.8535
772.243	33.8912
772.716	29.0547
773.19	30.1683
773.665	32.5305
774.138	32.168
774.611	26.9189
775.086	25.4507
775.559	28.106
776.032	28.9943
776.507	32.1207
776.98	28.1065
777.453	31.506
777.928	28.7898
778.401	28.3525
778.874	30.4639
779.347	28.1459
779.822	28.3275
780.295	31.7916
780.767	30.8632

781.242	31.1334
781.715	28.5019
782.188	33.3453
782.66	29.8115
783.135	30.9211
783.608	28.693
784.08	30.9447
784.553	27.3001
785.026	25.1306
785.5	30.4499
785.973	25.4069
786.445	31.8786
786.918	27.1773
787.39	28.4062
787.864	27.6537
788.337	25.7854
788.809	27.5001
789.282	25.993
789.754	29.7972
790.226	28.9767
790.701	28.4522
791.173	28.0875
791.645	31.7239
792.117	30.9436
792.589	30.2874
793.062	25.811
793.534	27.2655

794.006	32.0677
794.478	28.3412
794.952	27.5454
795.424	28.673
795.896	29.2297
796.368	29.2332
796.84	30.9745
797.312	25.6641
797.784	32.1748
798.256	29.4678
798.728	28.1995
799.199	32.5363
799.671	29.8096
800.143	29.0369
800.615	29.1349
801.087	28.0716
801.558	30.4792
802.03	25.5881
802.502	28.7373
802.973	28.4286
803.445	30.6102
803.917	28.6567
804.388	27.7709
804.86	32.1569
805.331	28.4583
805.803	29.0612
806.274	29.769

806.746	26.7517
807.217	26.6775
807.688	26.2184
808.16	24.1369
808.631	26.0451
809.102	27.8929
809.572	29.7982
810.043	29.2429
810.514	32.5716
810.986	31.2714
811.457	27.7182
811.928	27.7632
812.399	26.1993
812.87	24.9499
813.341	26.1967
813.81	24.8962
814.281	30.0773
814.752	31.7601
815.223	30.984
815.694	27.207
816.165	25.6525
816.634	27.2526
817.105	28.1296
817.576	29.2288
818.047	29.3923
818.518	26.8491
818.987	28.2669

819.457	29.0501
819.928	26.5677
820.399	28.6424
820.87	29.0721
821.338	29.3906
821.809	31.1694
822.28	29.6944
822.75	25.0717
823.219	26.0344
823.689	27.0627
824.16	28.1584
824.628	30.8216
825.099	29.203
825.569	30.0213
826.04	30.6567
826.508	32.2693
826.979	31.2202
827.449	29.5252
827.917	28.8001
828.388	31.0122
828.858	30.2365
829.326	30.722
829.797	32.3222
830.267	28.9564
830.735	29.2255
831.205	29.4867
831.675	30.0786

832.143	29.016
832.614	27.9476
833.082	30.6999
833.552	27.68
834.022	25.7371
834.49	27.5147
834.96	29.1912
835.428	28.7034
835.898	28.7761
836.367	27.3127
836.835	29.5963
837.305	31.3272
837.773	32.6289
838.243	31.071
838.711	24.3389
839.18	22.4009
839.648	29.5224
840.118	32.0017
840.586	27.2113
841.055	22.2872
841.523	27.3924
841.992	32.3309
842.46	33.8379
842.93	31.3254
843.397	24.8772
843.867	30.3016
844.334	28.4159

844.804	28.9003
845.271	27.8576
845.741	27.7211
846.208	27.7204
846.677	28.2122
847.145	29.4789
847.612	28.676
848.081	26.9742
848.549	30.8426
849.018	28.6218
849.485	24.5368
849.954	24.8635
850.421	22.1639
850.889	29.2428
851.358	27.7129
851.825	28.2705
852.292	31.2134
852.761	28.9635
853.228	26.1698
853.695	27.533
854.164	26.1907
854.631	28.5009
855.1	33.0494
855.567	29.1864
856.034	26.0408
856.501	28.1208
856.969	31.1132

857.436	27.8759
857.903	29.4764
858.372	27.2982
858.839	24.091
859.305	27.5438
859.774	25.9041
860.241	27.6993
860.707	27.5804
861.174	25.8198
861.621	27.7227
862.091	27.734
862.56	29.7017
863.03	27.6998
863.501	27.7107
863.971	25.7339
864.442	31.5894
864.912	29.651
865.38	31.6051
865.851	31.5062
866.321	25.6945
866.791	29.6138
867.262	27.8697
867.732	22.0156
868.2	31.5939
868.67	27.6844
869.14	29.6539
869.61	31.5058

870.081	25.7563
870.549	31.4573
871.019	25.6769
871.489	33.342
871.959	27.7698
872.427	31.5103
872.897	25.6849
873.367	25.4216
873.836	33.1795
874.304	29.7397
874.774	31.5424
875.244	29.3581
875.712	31.5172
876.182	23.938
876.651	27.8715
877.121	25.7781
877.589	24.164
878.058	27.8937
878.528	25.7799
878.996	27.8274
879.465	29.5893
879.935	29.7108
880.403	24.1667
880.872	27.6774
881.342	29.4381
881.809	35.1213
882.279	22.1359

882.746	33.3478
883.215	22.4251
883.685	26.0001
884.152	29.8815
884.621	23.4741
885.089	27.9455
885.558	28.0951
886.027	27.9824
886.495	24.6703
886.964	31.1183
887.431	27.9103
887.9	27.7155
888.369	26.0181
888.836	30.1342
889.306	33.199
889.773	29.2367
890.242	34.5587
890.709	29.9089
891.178	29.2707
891.645	29.9728
892.114	31.6855
892.581	27.7322
893.049	31.4515
893.516	33.1324
893.985	28.6209
894.452	25.1786
894.921	27.4793

895.388	29.121
895.856	25.7162
896.323	25.4337
896.792	27.2078
897.259	29.1303
897.725	30.8052
898.194	27.7158
898.661	24.3469
899.129	31.3955
899.596	32.0183
900.064	29.127
900.531	24.0503
900.997	34.0621
901.466	27.0717
901.932	27.0433
902.401	28.9519
902.867	27.1928
903.333	29.6316
903.802	30.7233
904.268	27.365
904.734	26.0491
905.203	26.418
905.669	31.6741
906.135	26.287
906.603	34.5906
907.07	30.6812
907.536	29.2852

908.004	26.4483
908.47	32.9389
908.936	26.5206
909.404	26.1923
909.87	33.5197
910.336	31.1278
910.804	24.8895
911.27	31.3105
911.736	24.3115
912.202	26.0654
912.67	25.815
913.136	26.0683
913.602	31.1965
914.067	26.4716
914.535	27.6637
915.001	32.8342
915.467	23.533
915.932	28.3643
916.4	20.5305
916.866	31.5873
917.331	26.227
917.797	28.2839
918.263	31.0492
918.73	25.8655
919.196	27.9321
919.661	30.0709
920.127	24.6667

920.592	30.9305
921.058	29.3926
921.525	28.0713
921.991	32.3824
922.456	28.8837
922.921	26.881
923.387	27.7082
923.852	28.2521
924.317	28.9258
924.783	28.6472
925.25	32.7961
925.715	29.3884
926.18	34.2899
926.646	26.0642
927.111	29.186
927.576	27.0749
928.041	24.718
928.506	29.7335
928.971	25.4208
929.436	25.7567
929.901	27.1211
930.366	29.7341
930.831	26.5709
931.296	26.9509
931.761	30.0042
932.226	31.1455
932.691	24.4198

933.155	26.5931
933.62	30.864
934.085	29.4142
934.55	30.5236
935.015	29.7355
935.479	28.6547
935.944	32.5139
936.409	26.9576
936.873	28.8793
937.338	27.505
937.802	26.2715
938.267	32.807
938.732	28.3796
939.196	28.3835
939.661	25.9735
940.125	30.3915
940.59	32.2393
941.054	27.075
941.518	26.5664
941.981	32.5261
942.445	30.3901
942.91	29.3255
943.374	28.9472
943.838	29.0088
944.302	29.3073
944.767	29.2242
945.231	27.0415

945.693	30.6879
946.157	26.5761
946.621	32.1972
947.086	28.0741
947.55	29.1136
948.014	32.8478
948.476	30.0042
948.94	27.9663
949.404	30.1096
949.868	28.7252
950.332	26.2596
950.794	26.9966
951.258	26.9928
951.721	27.8258
952.185	26.7025
952.649	33.1357
953.111	30.2158
953.575	29.7399
954.039	27.412
954.502	27.3404
954.964	31.6436
955.428	27.924
955.891	29.2202
956.353	28.3796
956.817	37.8261
957.28	26.9267
957.744	33.9831

958.205	33.0264
958.669	24.9932
959.132	29.6543
959.594	33.8768
960.057	26.1405
960.521	32.0018
960.982	31.6024
961.446	25.8623
961.909	27.9768
962.37	30.7967
962.834	34.3733
963.297	27.2295
963.758	32.3048
964.221	27.7415
964.683	28.6262
965.146	27.6803
965.609	31.3828
966.07	31.192
966.533	28.1261
966.994	28.619
967.457	29.6868
967.921	29.9722
968.382	25.6081
968.845	32.7676
969.306	25.5347
969.768	26.7023
970.229	32.8381

970.692	32.8153
971.153	30.8305
971.616	27.558
972.077	28.9841
972.54	30.6539
973.001	30.6694
973.463	29.9115
973.924	32.607
974.387	29.0361
974.848	27.5566
975.31	30.6493
975.771	27.9848
976.234	28.3055
976.694	26.7355
977.157	27.8066
977.617	29.6701
978.08	29.7425
978.54	28.8546
979.003	31.5635
979.463	29.8407
979.924	27.7119
980.386	27.8538
980.847	32.6457
981.309	29.346
981.769	30.0093
982.23	24.3115
982.692	29.4334

983.152	31.4379
983.614	30.8838
984.075	29.8699
984.535	28.7196
984.997	30.4101
985.457	28.7072
985.917	30.1392
986.38	26.8578
986.84	34.1569
987.3	29.4425
987.762	30.2358
988.222	29.8833
988.682	29.6274
989.144	27.7426
989.604	30.9874
990.064	29.8493
990.524	30.7976
990.985	32.6947
991.445	27.9758
991.905	28.5287
992.367	30.7781
992.827	27.7424
993.286	30.199
993.746	31.4817
994.208	34.2
994.668	29.4443
995.127	30.6865

995.587	30.8574
996.047	30.1121
996.508	29.6136
996.968	26.2868
997.427	28.7596
997.887	29.6833
998.346	29.2888
998.808	26.5005
999.267	33.7419
999.727	29.7635
1000.19	28.7555
1000.65	30.1999
1001.1	30.2926
1001.57	27.3438
1002.03	32.0906
1002.48	28.8682
1002.94	29.1242
1003.4	26.6599
1003.86	27.5816
1004.32	32.2711
1004.78	33.0425
1005.24	32.1816
1005.7	29.0964
1006.16	30.2991
1006.62	29.56
1007.08	29.8622
1007.54	32.9305

1008	30.5119
1008.46	26.5233
1008.91	28.9059
1009.37	29.9333
1009.83	29.6203
1010.29	28.9786
1010.75	26.4939
1011.21	28.6539
1011.67	29.0555
1012.13	32.539
1012.59	28.7973
1013.04	26.1094
1013.5	32.6805
1013.96	31.9338
1014.42	30.4673
1014.88	29.6329
1015.34	29.899
1015.8	29.3608
1016.25	27.0274
1016.71	28.2313
1017.17	30.1765
1017.63	26.9183
1018.09	31.4948
1018.55	32.6431
1019.01	29.8078
1019.46	25.3991
1019.92	25.3581

1020.38	31.045
1020.84	29.3113
1021.3	27.7385
1021.75	29.4834
1022.21	29.3313
1022.67	28.5353
1023.13	31.3456
1023.59	28.7033
1024.04	26.4441
1024.5	28.3807
1024.96	27.4571
1025.42	29.4547
1025.88	30.1865
1026.33	29.9215
1026.79	29.3804
1027.25	27.82
1027.71	33.3963
1028.16	26.0438
1028.62	28.7235
1029.08	27.0307
1029.54	30.617
1029.99	29.7891
1030.45	25.1681
1030.91	31.1731
1031.37	30.9078
1031.82	28.81
1032.28	30.9268

1032.74	30.6625
1033.19	28.2123
1033.65	35.384
1034.11	26.4899
1034.57	29.9691
1035.02	29.925
1035.48	31.7439
1035.94	30.9866
1036.39	32.8207
1036.85	30.3744
1037.31	28.4038
1037.76	30.2656
1038.22	28.3455
1038.68	24.6766
1039.14	22.6236
1039.59	29.0556
1040.05	25.8825
1040.51	30.3664
1040.96	31.2632
1041.42	20.4873
1041.88	27.764
1042.33	28.7962
1042.79	31.2471
1043.25	29.751
1043.7	35.2501
1044.16	26.7512
1044.61	32.6816

1045.07	29.3379
1045.53	32.2421
1045.98	27.8854
1046.44	22.7412
1046.9	31.4133
1047.35	29.4479
1047.81	26.3716
1048.26	27.6194
1048.72	23.2768
1049.18	27.6419
1049.63	27.2977
1050.09	27.7294
1050.54	28.2984
1051	31.7172
1051.46	30.5031
1051.91	28.4907
1052.37	29.3197
1052.82	26.5604
1053.28	29.8451
1053.73	34.4505
1054.19	28.3291
1054.64	34.2868
1055.1	32.641
1055.56	28.2947
1056.01	28.5695
1056.47	34.3248
1056.92	31.3515

1057.38	28.7876
1057.83	33.6305
1058.29	30.9906
1058.74	31.3706
1059.2	29.3955
1059.65	30.2958
1060.11	32.2924
1060.57	27.3643
1061.02	27.4041
1061.48	28.2053
1061.93	30.3807
1062.38	27.2842
1062.84	27.5805
1063.29	28.6737
1063.75	32.4953
1064.21	31.8007
1064.66	28.4966
1065.11	28.9579
1065.57	26.2391
1066.02	23.7222
1066.48	29.1424
1066.93	30.0324
1067.39	29.9785
1067.84	30.6047
1068.3	28.8909
1068.75	27.4502
1069.21	29.6294

1069.66	29.8558
1070.11	27.0345
1070.57	27.9384
1071.02	26.3076
1071.48	27.0398
1071.93	31.0492
1072.39	30.1681
1072.84	28.1908
1073.29	28.7243
1073.75	28.9533
1074.2	28.167
1074.66	25.2334
1075.11	25.2675
1075.56	31.2274
1076.02	32.982
1076.47	29.6491
1076.93	24.3234
1077.38	26.3846
1077.83	31.5296
1078.29	32.1184
1078.74	29.0175
1079.19	28.772
1079.65	30.576
1080.1	32.0459
1080.56	33.031
1081.01	31.7081
1081.46	27.9059

1081.92	27.6562
1082.37	29.4023
1082.82	30.8357
1083.28	31.7128
1083.73	30.3372
1084.18	31.1516
1084.64	29.6163
1085.09	27.1806
1085.54	29.1969
1086	28.8692
1086.45	29.005
1086.9	31.3591
1087.36	30.3466
1087.81	30.6182
1088.26	34.521
1088.71	32.2032
1089.17	29.8068
1089.62	30.5214
1090.07	31.2573
1090.53	31.8808
1090.98	31.6252
1091.43	31.6288
1091.88	31.7072
1092.34	31.5648
1092.79	31.0953
1093.24	29.5162
1093.69	29.9816

1094.15	30.6969
1094.6	29.3342
1095.05	31.632
1095.5	26.2441
1095.96	29.9907
1096.41	36.0707
1096.86	31.6021
1097.31	31.2963
1097.77	29.6378
1098.22	30.1169
1098.67	31.7303
1099.12	30.8592
1099.57	27.7936
1100.03	28.7776
1100.48	33.5402
1100.86	33.5356
1101.31	35.4558
1101.77	33.5456
1102.22	37.348
1102.68	31.563
1103.13	25.7755
1103.59	27.6835
1104.04	33.5689
1104.5	27.8824
1104.95	35.5446
1105.41	31.669
1105.86	29.7701

1106.32	29.6608
1106.77	29.57
1107.23	29.6152
1107.68	33.6193
1108.14	31.8338
1108.59	29.781
1109.05	35.2399
1109.5	31.3491
1109.95	27.4708
1110.41	33.1199
1110.86	25.9922
1111.32	27.8108
1111.77	29.4387
1112.23	27.6476
1112.68	29.5081
1113.13	31.3993
1113.59	33.5487
1114.04	37.2623
1114.5	31.4923
1114.95	25.9738
1115.41	27.9794
1115.86	31.5864
1116.31	31.4407
1116.77	24.2932
1117.22	35.0889
1117.67	31.4207
1118.13	27.7848

1118.58	31.2788
1119.04	24.3537
1119.49	27.9466
1119.94	33.2359
1120.4	29.5953
1120.85	31.816
1121.3	28.4891
1121.76	29.4361
1122.21	27.3582
1122.66	28.8685
1123.12	23.7412
1123.57	34.3507
1124.03	32.8237
1124.48	27.8163
1124.93	27.7817
1125.38	32.5174
1125.84	31.9649
1126.29	29.8161
1126.74	31.6046
1127.2	29.4708
1127.65	27.6774
1128.1	29.1814
1128.56	26.0158
1129.01	30.2619
1129.46	29.8475
1129.91	27.5778
1130.37	28.0511

1130.82	31.3811
1131.27	29.5024
1131.73	31.4371
1132.18	33.2354
1132.63	33.5781
1133.09	32.6631
1133.54	28.9309
1133.99	29.6447
1134.44	30.8474
1134.9	26.4344
1135.35	36.4747
1135.8	28.7987
1136.25	32.1069
1136.7	28.8227
1137.16	27.0257
1137.61	30.7885
1138.06	30.1623
1138.51	28.8334
1138.97	31.6884
1139.42	27.6915
1139.87	31.9969
1140.32	27.1888
1140.78	33.8332
1141.23	34.3062
1141.68	29.6573
1142.13	26.5237
1142.58	28.9862

1143.04	29.7301
1143.49	33.694
1143.94	28.9096
1144.39	24.962
1144.84	30.5965
1145.29	32.4762
1145.75	29.3304
1146.2	26.894
1146.65	30.4372
1147.1	27.7567
1147.55	32.6232
1148	30.2469
1148.46	28.2381
1148.91	33.9481
1149.36	25.5731
1149.81	29.5567
1150.26	25.0621
1150.71	29.7668
1151.16	30.4027
1151.62	27.1004
1152.07	30.4358
1152.52	28.342
1152.97	24.2129
1153.42	27.8842
1153.87	29.9873
1154.32	28.7808
1154.77	30.874

1155.22	32.0779
1155.68	29.322
1156.13	29.3338
1156.58	31.3994
1157.03	29.7709
1157.48	28.3039
1157.93	28.7547
1158.38	33.2313
1158.83	28.9828
1159.28	31.0177
1159.73	29.3268
1160.18	29.2169
1160.63	25.4428
1161.08	30.7452
1161.54	29.5988
1161.99	27.0715
1162.44	26.3385
1162.89	30.9792
1163.34	31.6021
1163.79	29.188
1164.24	32.883
1164.69	27.5577
1165.14	27.6576
1165.59	32.5839
1166.04	34.5512
1166.49	30.8142
1166.94	28.9811

1167.39	29.1589
1167.84	32.3444
1168.29	30.7227
1168.74	29.1479
1169.19	29.5705
1169.64	33.4414
1170.09	32.7029
1170.54	27.287
1170.99	31.1296
1171.44	26.6616
1171.89	27.3599
1172.34	32.0661
1172.79	30.8746
1173.24	24.0353
1173.69	30.734
1174.14	29.1287
1174.59	32.1851
1175.03	26.7897
1175.48	27.4366
1175.93	29.2669
1176.38	28.2703
1176.83	28.3834
1177.28	27.8648
1177.73	32.2434
1178.18	29.0626
1178.63	33.3113
1179.08	27.4687

1179.53	30.4873
1179.98	33.5428
1180.43	31.7591
1180.88	31.0478
1181.32	31.7868
1181.77	29.7869
1182.22	28.6598
1182.67	31.3362
1183.12	30.3265
1183.57	31.9121
1184.02	28.3159
1184.47	27.6598
1184.92	31.4345
1185.37	28.8977
1185.81	30.3528
1186.26	34.3279
1186.71	29.8895
1187.16	29.876
1187.61	34.0214
1188.06	33.6387
1188.5	33.618
1188.95	30.0335
1189.4	27.9941
1189.85	28.1496
1190.3	32.1166
1190.75	36.8169
1191.2	30.5273

1191.64	28.9554
1192.09	35.4903
1192.54	28.6842
1192.99	32.8444
1193.44	34.0666
1193.88	30.9016
1194.33	33.2402
1194.78	31.1972
1195.23	30.6375
1195.68	31.4183
1196.12	35.8833
1196.57	35.1798
1197.02	31.5375
1197.47	32.0317
1197.92	33.1451
1198.36	26.197
1198.81	31.8007
1199.26	32.0125
1199.71	29.1052
1200.15	30.2408
1200.6	26.1851
1201.05	30.0725
1201.5	31.4197
1201.94	29.393
1202.39	26.3477
1202.84	28.1745
1203.29	31.006

1203.73	32.2372
1204.18	32.0832
1204.63	29.6538
1205.07	32.6862
1205.52	32.6427
1205.97	33.6402
1206.42	30.4365
1206.86	33.2839
1207.31	30.949
1207.76	30.6221
1208.2	33.9107
1208.65	29.1917
1209.1	35.7297
1209.55	34.6178
1209.99	33.9265
1210.44	36.3055
1210.89	30.947
1211.33	31.9173
1211.78	35.708
1212.23	34.7178
1212.67	32.7297
1213.12	34.7497
1213.57	30.707
1214.01	27.8124
1214.46	29.7482
1214.9	31.7425
1215.35	32.8066

1215.8	29.8997
1216.24	31.7774
1216.69	31.7772
1217.14	29.8655
1217.58	32.7288
1218.03	30.7469
1218.48	30.882
1218.92	30.7043
1219.37	28.6302
1219.82	34.6819
1220.26	28.2351
1220.71	30.8505
1221.15	30.5977
1221.6	33.6419
1222.04	31.0353
1222.49	30.7692
1222.94	32.5929
1223.38	32.6518
1223.83	32.8759
1224.27	33.5139
1224.72	34.2723
1225.17	33.5908
1225.61	32.6762
1226.06	32.0837
1226.5	35.4431
1226.95	30.1976
1227.39	32.3765

1227.84	22.8846
1228.28	34.4807
1228.73	30.579
1229.18	36.0736
1229.62	37.8568
1230.07	34.3525
1230.51	37.1384
1230.96	34.5032
1231.4	30.1009
1231.85	29.2499
1232.29	31.4975
1232.74	33.652
1233.18	32.4728
1233.63	27.7682
1234.07	32.0429
1234.52	29.665
1234.96	32.7911
1235.41	34.2723
1235.85	33.1598
1236.3	28.3728
1236.74	26.9794
1237.19	28.482
1237.63	33.1137
1238.08	30.9182
1238.52	32.1983
1238.97	32.997
1239.41	32.237

1239.85	30.6699
1240.3	33.3839
1240.75	35.8742
1241.19	30.6827
1241.63	31.1393
1242.08	31.3429
1242.52	32.2445
1242.97	31.7611
1243.41	33.2586
1243.86	32.3514
1244.3	31.2656
1244.74	29.2282
1245.19	30.4401
1245.63	28.4961
1246.08	30.4642
1246.52	30.4599
1246.96	31.1777
1247.41	33.1946
1247.85	35.7762
1248.3	33.6202
1248.74	31.6415
1249.18	34.4804
1249.63	32.4009
1250.07	29.7412
1250.52	29.7533
1250.96	31.0808
1251.4	27.1213

1251.85	29.7015
1252.29	34.4044
1252.73	31.8554
1253.18	31.4792
1253.62	33.7512
1254.06	31.2517
1254.51	29.774
1254.95	33.8451
1255.4	34.8208
1255.84	35.1277
1256.28	35.0831
1256.73	37.7479
1257.17	34.9179
1257.61	33.7669
1258.05	34.7984
1258.5	33.4135
1258.94	33.7455
1259.38	33.3622
1259.83	31.0542
1260.27	32.2255
1260.71	29.9044
1261.16	31.5749
1261.6	32.7699
1262.04	28.4868
1262.49	30.8261
1262.93	32.6217
1263.37	34.7452

1263.81	32.218
1264.26	32.1166
1264.7	32.5956
1265.14	32.5056
1265.58	29.077
1266.03	30.7989
1266.47	25.4097
1266.91	30.1141
1267.35	34.3988
1267.8	34.7377
1268.24	28.5438
1268.68	31.5633
1269.13	36.1873
1269.57	33.58
1270.01	32.04
1270.45	32.0352
1270.89	33.9296
1271.34	32.7917
1271.78	35.932
1272.22	32.2346
1272.66	31.7414
1273.11	39.8135
1273.55	35.8469
1273.99	33.7254
1274.43	30.4059
1274.87	33.7968
1275.32	30.9835

1275.76	35.484
1276.2	37.2795
1276.64	36.7716
1277.08	33.5455
1277.52	40.5198
1277.96	35.2924
1278.41	35.7206
1278.85	34.6378
1279.29	32.1366
1279.73	32.2456
1280.17	27.2074
1280.61	31.6433
1281.06	30.0524
1281.5	38.5218
1281.94	37.9758
1282.38	31.3649
1282.82	34.1277
1283.26	35.6724
1283.7	30.1392
1284.15	41.4142
1284.59	39.3812
1285.03	32.6913
1285.47	32.6195
1285.91	34.4284
1286.35	34.579
1286.79	35.7078
1287.23	33.4427

1287.67	35.609
1288.11	30.3808
1288.56	34.4759
1289	31.3012
1289.44	33.7503
1289.88	37.3931
1290.32	35.2227
1290.76	40.5364
1291.2	31.162
1291.64	37.2106
1292.08	33.8205
1292.52	38.7301
1292.96	33.8338
1293.4	35.5017
1293.85	30.7623
1294.29	34.8928
1294.72	35.4119
1295.17	37.3961
1295.61	38.2814
1296.05	31.5683
1296.49	34.1522
1296.93	36.8194
1297.37	37.6899
1297.81	36.3129
1298.25	34.5815
1298.69	40.0909
1299.13	34.2409

1299.57	35.08
1300.01	35.4486
1300.45	31.492
1300.89	39.0807
1301.33	34.5587
1301.77	34.9274
1302.21	37.3966
1302.65	33.8585
1303.09	34.2071
1303.53	32.9746
1303.97	36.6548
1304.41	35.1824
1304.85	33.8704
1305.29	34.3207
1305.72	36.3162
1306.16	35.0985
1306.6	38.7746
1307.04	35.2561
1307.48	34.0015
1307.92	34.3478
1308.36	34.8162
1308.8	35.6684
1309.24	34.2324
1309.68	34.9337
1310.12	30.9313
1310.56	33.7
1311	36.1601

1311.44	38.7582
1311.88	39.9396
1312.32	38.3536
1312.76	31.3946
1313.19	34.2677
1313.63	41.1894
1314.07	35.0248
1314.51	33.7546
1314.95	38.6428
1315.39	39.1454
1315.83	36.7651
1316.27	35.7554
1316.7	35.5939
1317.14	33.5647
1317.58	35.5775
1318.02	33.939
1318.46	32.5782
1318.9	35.6475
1319.34	38.1296
1319.77	37.833
1320.21	36.1991
1320.65	37.7307
1321.09	34.0343
1321.53	32.558
1321.97	35.1975
1322.41	37.559
1322.84	36.8963

1323.28	36.4379
1323.72	37.6027
1324.16	36.1431
1324.6	35.8476
1325.04	39.5682
1325.47	37.4206
1325.91	37.0143
1326.35	39.392
1326.79	33.0075
1327.08	35.4937
1327.52	33.6491
1327.96	37.5496
1328.4	31.7171
1328.84	37.4439
1329.29	31.6476
1329.73	29.736
1330.17	31.6945
1330.61	33.6517
1331.05	35.5297
1331.49	33.7228
1331.93	37.3971
1332.37	31.4279
1332.81	39.2408
1333.25	31.6374
1333.69	33.4019
1334.13	35.309
1334.57	33.5083

1335.01	29.7064
1335.45	35.3188
1335.89	35.5069
1336.33	31.7022
1336.77	28.1336
1337.21	31.8996
1337.65	29.5827
1338.09	31.5453
1338.54	33.74
1338.98	33.6607
1339.42	31.744
1339.86	31.7433
1340.29	31.5912
1340.73	35.4255
1341.17	33.7547
1341.61	40.9996
1342.05	39.1567
1342.49	29.942
1342.93	35.2317
1343.37	35.2083
1343.81	35.3007
1344.25	35.2938
1344.69	30.0422
1345.13	33.9621
1345.57	34.0837
1346.01	30.4185
1346.45	33.8031

1346.89	35.2916
1347.33	35.114
1347.77	30.0787
1348.21	24.8634
1348.65	29.7377
1349.09	28.0362
1349.53	32.16
1349.97	30.1482
1350.41	33.3714
1350.85	32.1115
1351.29	33.8454
1351.72	37.7924
1352.16	35.2589
1352.6	34.6247
1353.04	29.9594
1353.48	36.6213
1353.92	33.0117
1354.36	33.8889
1354.8	28.6289
1355.24	38.9551
1355.67	33.322
1356.11	31.8903
1356.55	32.9518
1356.99	33.8429
1357.43	35.0846
1357.87	31.7776
1358.31	35.0798

1358.75	27.532
1359.19	39.9905
1359.62	33.6651
1360.06	31.001
1360.5	30.3
1360.94	31.7043
1361.38	33.4308
1361.82	30.3301
1362.25	34.8138
1362.69	26.0263
1363.13	32.2331
1363.57	36.3253
1364.01	31.5898
1364.44	32.6039
1364.88	31.0778
1365.32	26.787
1365.76	28.1948
1366.2	33.7129
1366.64	30.3951
1367.07	28.0601
1367.51	35.9524
1367.95	39.3161
1368.39	34.4013
1368.83	33.3098
1369.26	33.3792
1369.7	32.5486
1370.14	34.0114

1370.58	30.9596
1371.02	33.7206
1371.45	36.8603
1371.89	38.4189
1372.33	30.5992
1372.77	32.1321
1373.2	29.7503
1373.64	38.364
1374.08	30.1563
1374.52	28.8284
1374.95	33.2635
1375.39	32.797
1375.83	32.1895
1376.26	34.4579
1376.7	33.2127
1377.14	33.1299
1377.58	28.17
1378.01	30.8369
1378.45	32.725
1378.89	27.7601
1379.32	31.7652
1379.76	35.1812
1380.2	26.1584
1380.64	28.329
1381.07	32.6368
1381.51	35.8038
1381.95	33.164

1382.38	33.2114
1382.82	31.2113
1383.26	30.9219
1383.69	32.0753
1384.13	29.8869
1384.57	31.6679
1385	33.1922
1385.44	31.7078
1385.88	34.3883
1386.31	33.8655
1386.75	32.0183
1387.19	32.3151
1387.62	32.8068
1388.06	26.9014
1388.5	29.7602
1388.93	32.2245
1389.37	34.4496
1389.8	35.5059
1390.24	25.5349
1390.68	29.2241
1391.11	31.2894
1391.55	33.9215
1391.99	28.8117
1392.42	31.593
1392.86	27.3163
1393.29	29.2969
1393.73	29.584

1394.17	26.5623
1394.6	32.7586
1395.04	31.7573
1395.47	29.0475
1395.91	31.1635
1396.35	34.9071
1396.78	28.208
1397.22	29.3208
1397.65	32.0093
1398.09	34.8718
1398.52	29.772
1398.96	31.5554
1399.4	27.1188
1399.83	29.329
1400.27	32.5629
1400.7	32.5386
1401.14	31.7661
1401.57	34.3728
1402.01	30.8921
1402.44	33.3134
1402.88	29.2304
1403.32	28.2074
1403.75	31.1406
1404.19	30.1849
1404.62	26.3002
1405.06	26.6775
1405.49	34.7656

1405.93	32.3731
1406.36	33.034
1406.8	29.6571
1407.23	35.8864
1407.67	30.4509
1408.1	29.1391
1408.54	34.6448
1408.97	31.008
1409.41	30.2384
1409.84	36.5822
1410.27	32.257
1410.71	28.5944
1411.14	33.2741
1411.58	33.1731
1412.01	29.2815
1412.45	28.1621
1412.88	35.7437
1413.32	31.7368
1413.75	32
1414.19	32.8933
1414.62	28.4188
1415.06	27.8696
1415.49	29.4549
1415.92	33.47
1416.36	30.2716
1416.79	33.6777
1417.23	28.4325

1417.66	29.7974
1418.1	26.099
1418.53	30.4012
1418.96	32.993
1419.4	27.4491
1419.83	28.9434
1420.27	29.5181
1420.7	29.2423
1421.13	26.6845
1421.57	34.0449
1422	29.3386
1422.43	24.5592
1422.87	27.7628
1423.3	25.6723
1423.74	30.6502
1424.17	30.7374
1424.6	34.6567
1425.04	25.6149
1425.47	27.5888
1425.91	28.3116
1426.34	32.1866
1426.77	31.3673
1427.21	30.3731
1427.64	30.7303
1428.07	32.0021
1428.51	28.4843
1428.94	34.4824

1429.37	28.9344
1429.81	31.6285
1430.24	28.9294
1430.67	31.6362
1431.1	29.8061
1431.54	32.0345
1431.97	30.1704
1432.4	26.4393
1432.84	34.7443
1433.27	30.0731
1433.7	29.7398
1434.14	32.3135
1434.57	29.5414
1435	34.0333
1435.44	28.4256
1435.87	32.8178
1436.3	32.2952
1436.73	29.0204
1437.17	29.8945
1437.6	29.5054
1438.03	28.9892
1438.46	29.8091
1438.9	30.7346
1439.33	28.8792
1439.76	33.7433
1440.2	31.7473
1440.63	32.5737

1441.06	28.7266
1441.49	31.8099
1441.92	30.7589
1442.36	30.6403
1442.79	24.5377
1443.22	29.7533
1443.65	33.7761
1444.09	30.8098
1444.52	30.7432
1444.95	29.5822
1445.38	29.7133
1445.82	31.3991
1446.25	33.5473
1446.68	32.0218
1447.11	33.5835
1447.54	32.8784
1447.97	32.0231
1448.41	30.8164
1448.84	29.8274
1449.27	31.825
1449.7	26.8475
1450.13	30.8792
1450.57	30.8191
1451	31.8298
1451.43	32.8124
1451.86	28.338
1452.29	31.5906

1452.72	32.9843
1453.16	31.0662
1453.59	28.0335
1454.02	31.4108
1454.45	30.8875
1454.88	32.5511
1455.31	28.3225
1455.75	26.5269
1456.18	29.581
1456.61	29.1324
1457.04	31.8366
1457.47	35.1647
1457.9	32.9282
1458.33	31.7269
1458.76	32.6494
1459.19	34.4722
1459.62	29.6088
1460.06	27.058
1460.49	30.2729
1460.92	26.6494
1461.35	31.5964
1461.78	30.8244
1462.21	28.4106
1462.64	30.9941
1463.07	29.0883
1463.5	31.7805
1463.93	31.8757

1464.36	28.6699
1464.8	29.0205
1465.23	30.949
1465.66	31.8458
1466.09	26.6076
1466.52	29.5235
1466.95	28.5852
1467.38	27.2006
1467.81	32.7621
1468.24	29.6632
1468.67	31.4496
1469.1	31.3999
1469.53	26.3997
1469.96	28.6739
1470.39	27.7517
1470.82	30.9389
1471.25	32.3215
1471.68	29.4088
1472.11	28.9764
1472.54	30.6539
1472.97	29.1573
1473.4	29.1916
1473.83	30.4858
1474.26	27.8758
1474.69	31.1562
1475.12	29.2342
1475.55	31.2688

1475.98	30.5585
1476.41	31.688
1476.84	32.5866
1477.27	28.6028
1477.7	31.7234
1478.13	30.0075
1478.56	31.0905
1478.99	29.2619
1479.42	29.0343
1479.85	30.6763
1480.28	30.4334
1480.71	31.3663
1481.14	27.5251
1481.57	28.4519
1482	30.9907
1482.42	29.683
1482.85	28.1169
1483.28	27.9229
1483.71	29.0308
1484.14	32.3018
1484.57	29.9348
1485	30.3538
1485.43	34.9136
1485.86	29.8316
1486.29	28.8725
1486.72	30.0498
1487.15	25.867

1487.57	28.3248
1488	29.5645
1488.43	26.1615
1488.86	29.8686
1489.29	29.7432
1489.72	31.4856
1490.15	29.8697
1490.58	28.9909
1491.01	29.8198
1491.43	32.0228
1491.86	31.3696
1492.29	29.187
1492.72	30.0402
1493.15	28.6703
1493.58	29.2676
1494	26.35
1494.43	27.8489
1494.86	29.4806
1495.29	32.9438
1495.72	31.0121
1496.15	26.3567
1496.57	30.5584
1497	30.5295
1497.43	28.5385
1497.86	30.114
1498.29	28.6413
1498.72	32.2993

1499.14	27.7251
1499.57	28.1714
1500	29.3085
1500.43	29.4168
1500.86	30.7814
1501.28	29.3554
1501.71	31.2565
1502.14	28.7881
1502.57	30.2801
1502.99	30.0523
1503.42	25.0042
1503.85	33.3842
1504.28	29.5388
1504.71	31.8479
1505.13	29.4513
1505.56	26.6557
1505.99	33.8833
1506.42	24.4792
1506.84	26.9117
1507.27	31.0656
1507.7	33.1362
1508.13	37.0742
1508.55	31.0581
1508.98	33.6668
1509.41	27.5766
1509.84	27.8863
1510.26	26.6601

1510.69	32.726
1511.12	27.4232
1511.54	31.4388
1511.97	29.7304
1512.4	23.5079
1512.83	30.653
1513.25	30.0903
1513.68	28.0983
1514.11	29.9611
1514.53	29.89
1514.96	28.137
1515.39	27.8906
1515.81	25.0047
1516.24	27.3215
1516.67	27.8916
1517.09	30.1204
1517.52	32.4516
1517.95	30.5558
1518.37	30.1496
1518.8	28.2543
1519.23	27.8934
1519.65	33.3664
1520.08	29.5037
1520.51	29.0087
1520.93	30.3619
1521.36	29.895
1521.79	30.9925

1522.21	29.6967
1522.64	29.8959
1523.06	27.7452
1523.49	30.8955
1523.92	31.8968
1524.34	31.289
1524.77	30.6381
1525.2	26.8194
1525.62	32.8326
1526.05	34.1075
1526.47	31.4662
1526.9	29.7193
1527.33	27.5028
1527.75	26.0712
1528.18	30.6196
1528.6	28.9043
1529.03	27.8873
1529.46	30.8926
1529.88	28.343
1530.31	29.4152
1530.73	33.8287
1531.16	32.9914
1531.58	30.9497
1532.01	31.2444
1532.44	30.7031
1532.86	29.9028
1533.29	30.2679

1533.71	30.9366
1534.14	28.5829
1534.56	28.2681
1534.99	28.4764
1535.41	28.7912
1535.84	29.9047
1536.26	29.6212
1536.69	30.8599
1537.11	29.9299
1537.54	26.9716
1537.96	26.913
1538.39	27.0681
1538.81	29.5736
1539.24	32.8198
1539.66	32.7804
1540.09	31.1135
1540.52	29.7889
1540.94	31.0138
1541.36	29.7633
1541.79	30.635
1542.21	32.6734
1542.64	33.0818
1543.06	29.5506
1543.49	28.3303
1543.91	31.9096
1544.34	30.4386
1544.76	28.6757

1545.19	32.771
1545.61	33.6094
1546.04	27.9943
1546.46	32.5299
1546.74	33.8134
1547.17	31.8727
1547.6	35.8566
1548.03	27.9553
1548.45	33.8065
1548.88	33.7941
1549.31	29.8695
1549.73	29.8908
1550.16	33.7019
1550.59	35.6268
1551.02	26.1558
1551.44	29.8609
1551.87	28.0854
1552.3	26.0891
1552.72	27.9117
1553.15	29.9802
1553.58	30.0436
1554	27.9925
1554.43	31.9704
1554.86	31.7916
1555.29	25.9275
1555.71	30.2649
1556.14	27.888

1556.57	24.0067
1556.99	33.669
1557.42	27.999
1557.85	31.5393
1558.27	37.5193
1558.7	24.2682
1559.13	30.0173
1559.55	33.6183
1559.98	26.2981
1560.41	26.0315
1560.83	35.4424
1561.26	30.0285
1561.68	30.036
1562.11	33.7239
1562.54	27.8985
1562.96	26.5121
1563.39	29.902
1563.82	31.3838
1564.24	26.2033
1564.67	31.8615
1565.09	27.575
1565.52	29.6499
1565.95	32.0061
1566.37	28.2882
1566.8	35.1171
1567.23	33.4393
1567.65	28.443

1568.08	35.108
1568.5	31.8094
1568.93	29.9012
1569.35	25.9359
1569.78	28.9774
1570.2	29.8693
1570.63	28.4496
1571.06	33.6688
1571.48	31.6658
1571.91	29.8631
1572.33	29.8622
1572.76	32.9456
1573.18	30.1597
1573.61	31.9174
1574.04	31.652
1574.46	38.7136
1574.89	29.2982
1575.31	26.4187
1575.74	28.2091
1576.16	29.8547
1576.59	33.4882
1577.01	29.9324
1577.44	33.6864
1577.86	33.4188
1578.29	29.8505
1578.71	31.3228
1579.14	32.9091

1579.56	26.604
1579.99	26.5907
1580.41	29.6269
1580.84	30.4027
1581.26	28.2881
1581.69	30.1717
1582.11	26.3198
1582.54	31.2554
1582.96	26.3142
1583.39	24.7674
1583.81	32.4603
1584.24	32.9295
1584.66	29.7532
1585.09	24.3487
1585.51	28.87
1585.94	27.7523
1586.36	23.8597
1586.79	27.6392
1587.21	34.1656
1587.63	25.6632
1588.06	27.4356
1588.48	35.8795
1588.91	28.6733
1589.33	31.3561
1589.76	35.364
1590.18	29.438
1590.6	25.7864

1591.03	28.5721
1591.45	30.5452
1591.88	30.7485
1592.3	28.4669
1592.72	31.4015
1593.15	27.3782
1593.57	26.0304
1594	33.5317
1594.42	33.6673
1594.85	31.1121
1595.27	28.4845
1595.69	28.5374
1596.12	33.0815
1596.54	33.1354
1596.96	32.8583
1597.39	28.492
1597.81	31.4115
1598.23	35.7628
1598.66	34.4314
1599.08	29.3109
1599.51	25.5349
1599.93	24.8675
1600.35	27.4857
1600.78	30.7799
1601.2	29.6581
1601.62	34.3942
1602.05	31.503

1602.47	32.1222
1602.89	27.623
1603.32	26.8676
1603.74	30.5636
1604.16	25.727
1604.58	28.9345
1605.01	26.3444
1605.43	29.4005
1605.85	31.3937
1606.28	25.7255
1606.7	26.8219
1607.12	34.9146
1607.55	28.6635
1607.97	29.818
1608.39	31.5411
1608.82	31.1996
1609.24	32.4678
1609.66	26.5118
1610.08	30.9218
1610.51	23.2434
1610.93	31.3029
1611.35	30.7521
1611.77	31.6324
1612.2	26.3941
1612.62	30.8744
1613.04	28.0521
1613.46	29.5667

1613.89	28.8143
1614.31	34.5001
1614.73	30.1267
1615.16	25.956
1615.58	26.9777
1616	27.5199
1616.42	26.2034
1616.85	29.0855
1617.27	30.2181
1617.69	25.9681
1618.11	27.5808
1618.53	28.7114
1618.95	30.2041
1619.38	28.4468
1619.8	33.1109
1620.22	27.7485
1620.64	26.452
1621.07	26.5259
1621.49	29.1304
1621.91	27.1907
1622.33	27.8416
1622.75	22.6083
1623.17	27.7635
1623.6	29.7503
1624.02	28.4749
1624.44	25.7544
1624.86	29.7347

1625.28	27.5716
1625.7	24.4676
1626.13	28.2538
1626.55	28.9654
1626.97	29.184
1627.39	26.5747
1627.81	30.4168
1628.23	26.081
1628.65	27.7932
1629.08	29.6779
1629.5	32.8937
1629.92	27.8735
1630.34	30.1655
1630.76	32.2801
1631.18	26.794
1631.6	26.5262
1632.03	25.8277
1632.45	26.2269
1632.87	28.7009
1633.29	33.8699
1633.71	25.3935
1634.13	27.6676
1634.55	31.2364
1634.97	29.3155
1635.39	29.5765
1635.81	29.4281
1636.24	26.9097

1636.66	27.738
1637.08	28.5629
1637.5	28.8551
1637.92	29.8157
1638.34	26.9566
1638.76	31.7876
1639.18	31.3193
1639.6	30.4452
1640.02	31.9964
1640.44	25.6414
1640.86	29.296
1641.28	28.1753
1641.7	33.3504
1642.12	32.2105
1642.54	30.4331
1642.96	31.6287
1643.38	27.0428
1643.8	33.5701
1644.22	33.0484
1644.64	32.9478
1645.06	29.0796
1645.49	29.8464
1645.91	27.378
1646.32	29.2798
1646.75	23.0236
1647.17	26.0656
1647.58	29.1124

1648	29.4778
1648.43	28.9077
1648.84	31.7986
1649.26	28.1783
1649.69	27.6834
1650.1	30.4239
1650.52	27.7758
1650.94	29.5437
1651.36	29.4135
1651.78	31.4779
1652.2	30.9988
1652.62	30.6852
1653.04	28.2018
1653.46	30.1384
1653.88	29.6172
1654.3	26.665
1654.72	31.665
1655.14	31.6413
1655.56	31.1237
1655.98	27.6918
1656.4	30.193
1656.82	30.6348
1657.24	30.1531
1657.66	31.658
1658.07	27.1777
1658.49	30.1306
1658.91	25.4834

1659.33	25.8958
1659.75	27.975
1660.17	28.8998
1660.59	26.0144
1661.01	27.1359
1661.43	29.6511
1661.85	31.4311
1662.26	26.4437
1662.68	26.9789
1663.1	29.0612
1663.52	26.5538
1663.94	30.5665
1664.36	27.135
1664.78	27.7269
1665.2	31.0946
1665.61	30.3862
1666.03	29.8771
1666.45	25.5124
1666.87	26.1852
1667.29	32.2358
1667.71	31.9998
1668.13	24.8233
1668.54	26.1841
1668.96	30.0935
1669.38	28.4825
1669.8	26.5433
1670.22	33.0155

1670.64	28.4881
1671.05	27.0717
1671.47	29.0692
1671.89	30.2431
1672.31	27.8884
1672.73	28.8374
1673.14	28.7336
1673.56	29.7399
1673.98	28.8038
1674.4	30.1888
1674.82	29.8917
1675.23	30.358
1675.65	29.613
1676.07	30.8341
1676.49	27.6501
1676.91	23.8528
1677.32	24.7383
1677.74	28.5513
1678.16	27.7341
1678.58	26.5265
1678.99	28.0809
1679.41	27.0514
1679.83	28.7139
1680.25	26.9656
1680.66	26.9983
1681.08	25.0867
1681.5	33.5985

1681.92	31.5914
1682.33	32.9766
1682.75	28.9296
1683.17	28.361
1683.58	24.5715
1684	26.3903
1684.42	28.1558
1684.84	27.4522
1685.26	28.1248
1685.67	26.9737
1686.09	24.2772
1686.51	23.3991
1686.92	28.8689
1687.34	26.3176
1687.76	24.973
1688.17	27.4011
1688.59	27.7739
1689.01	26.103
1689.42	26.9766
1689.84	29.9316
1690.26	26.5207
1690.67	28.2203
1691.09	28.8026
1691.51	28.3307
1691.92	30.7165
1692.34	30.1995
1692.76	24.0108

1693.17	28.5612
1693.59	27.9616
1694.01	27.7357
1694.42	30.507
1694.84	31.7459
1695.26	29.0989
1695.67	29.1591
1696.09	31.8746
1696.51	31.1833
1696.92	30.3505
1697.34	29.8071
1697.76	26.6335
1698.17	28.6726
1698.59	31.2057
1699	30.6854
1699.42	27.6316
1699.84	28.3207
1700.25	27.3379
1700.67	29.943
1701.08	29.8567
1701.5	26.1157
1701.91	23.5633
1702.33	24.7782
1702.75	25.6662
1703.16	28.1655
1703.58	29.4904
1703.99	30.4911

1704.41	29.521
1704.83	31.5268
1705.24	28.3695
1705.66	21.9796
1706.07	24.563
1706.49	27.5204
1706.9	31.9385
1707.32	34.984
1707.73	30.7261
1708.15	28.4584
1708.57	30.9414
1708.98	32.9257
1709.4	28.3388
1709.81	31.0919
1710.23	29.7491
1710.64	28.7837
1711.06	27.3592
1711.47	30.0456
1711.89	27.6677
1712.3	26.3976
1712.72	25.0443
1713.13	27.199
1713.55	29.5254
1713.96	28.7271
1714.38	29.7528
1714.79	28.9833
1715.21	30.6853

1715.62	32.2226
1716.04	28.2768
1716.45	29.0207
1716.87	29.5272
1717.28	26.3124
1717.7	28.8466
1718.11	29.7681
1718.53	25.8101
1718.94	29.7064
1719.36	29.9803
1719.77	28.3781
1720.18	27.7857
1720.6	27.7091
1721.01	27.2596
1721.43	26.7289
1721.84	28.1905
1722.26	27.1724
1722.67	26.7333
1723.09	30.7897
1723.5	28.9051
1723.91	26.9291
1724.33	31.535
1724.74	27.3538
1725.16	28.1397
1725.57	31.9053
1725.98	28.4305
1726.4	25.1752

1726.81	28.6171
1727.23	29.6551
1727.64	28.8746
1728.05	29.0517
1728.47	30.8369
1728.88	29.7066
1729.3	27.1249
1729.71	29.0142
1730.13	24.7444
1730.54	25.8422
1730.95	24.8564
1731.37	29.9218
1731.78	25.9745
1732.19	29.951
1732.61	32.4192
1733.02	29.9066
1733.43	31.0928
1733.85	26.5924
1734.26	26.2593
1734.67	26.8787
1735.09	26.8377
1735.5	30.6741
1735.91	29.1584
1736.33	27.622
1736.74	25.561
1737.16	30.9679
1737.57	28.9532

1737.98	26.7374
1738.39	29.7799
1738.81	23.8428
1739.22	26.1753
1739.63	32.8778
1740.05	30.5397
1740.46	27.6296
1740.87	26.6543
1741.28	22.0509
1741.7	25.7277
1742.11	27.9181
1742.52	32.8511
1742.94	34.4593
1743.35	30.8955
1743.76	28.8124
1744.18	29.2107
1744.59	25.593
1745	25.4592
1745.41	29.1438
1745.83	29.0295
1746.24	33.1121
1746.65	27.8249
1747.06	29.3887
1747.48	25.7111
1747.89	21.7405
1748.3	30.6942
1748.71	29.5584

1749.13	31.1027
1749.54	34.964
1749.95	29.7086
1750.36	28.8743
1750.77	29.2636
1751.19	31.1638
1751.6	29.4006
1752.01	27.5961
1752.42	30.8186
1752.84	26.2104
1753.25	35.4539
1753.66	30.6323
1754.07	21.0004
1754.48	27.8768
1754.9	32.5891
1755.31	30.0184
1755.72	27.717
1756.13	27.4489
1756.54	24.3285
1756.95	26.6424
1757.37	27.4787
1757.78	22.7606
1758.19	24.5107
1758.6	30.06
1759.01	28.4377
1759.42	30.454
1759.84	26.9896

1760.25	28.4109
1760.36	29.4513
1760.77	29.4524
1761.19	25.4535
1761.6	27.4529
1762.01	31.437
1762.43	29.4667
1762.84	33.4364
1763.26	25.4524
1763.67	33.3874
1764.09	33.4096
1764.5	27.5286
1764.91	25.5605
1765.33	29.462
1765.74	29.4219
1766.16	31.372
1766.57	27.5139
1766.98	23.5716
1767.4	31.3479
1767.81	33.3827
1768.23	27.5805
1768.64	27.4714
1769.05	29.3476
1769.47	21.6666
1769.88	25.5968
1770.29	25.603
1770.71	29.2576

1771.12	25.5462
1771.54	33.3346
1771.95	25.7373
1772.36	25.5749
1772.78	27.7014
1773.19	33.3348
1773.6	29.6283
1774.02	29.2271
1774.43	27.5939
1774.84	29.2873
1775.26	33.1732
1775.67	29.59
1776.08	31.1733
1776.5	29.5564
1776.91	30.8384
1777.33	31.1413
1777.74	29.6015
1778.15	30.9604
1778.56	25.8784
1778.98	27.868
1779.39	29.6534
1779.8	31.0356
1780.22	27.5079
1780.63	27.2722
1781.04	28.2268
1781.45	29.3565
1781.87	29.3673

1782.28	27.7935
1782.7	24.8755
1783.11	27.1737
1783.52	25.9622
1783.93	25.7685
1784.35	29.3403
1784.76	31.1285
1785.17	31.5602
1785.58	31.553
1786	25.5632
1786.41	30.4446
1786.82	31.0888
1787.23	28.5277
1787.65	22.3386
1788.06	26.3115
1788.47	29.3358
1788.88	30.8053
1789.3	32.27
1789.71	31.77
1790.12	28.6465
1790.53	30.8468
1790.95	31.0018
1791.36	28.5175
1791.77	23.1594
1792.18	27.6698
1792.59	32.1716
1793.01	31.4557

1793.42	27.6582
1793.83	29.5534
1794.24	27.0886
1794.66	30.3347
1795.07	22.1847
1795.48	29.2248
1795.89	22.5749
1796.3	29.8214
1796.71	29.6543
1797.13	26.2758
1797.54	30.701
1797.95	31.4083
1798.36	21.4198
1798.77	26.5947
1799.18	26.1094
1799.6	27.6071
1800.01	28.746
1800.42	28.2283
1800.83	29.8967
1801.24	24.5484
1801.65	28.1865
1802.06	31.04
1802.48	30.5498
1802.89	30.7256
1803.3	29.2206
1803.71	29.7945
1804.12	31.0445

1804.53	24.1482
1804.94	28.0771
1805.36	26.0029
1805.77	22.463
1806.18	24.9137
1806.59	27.323
1807	27.6243
1807.41	29.3398
1807.82	26.6434
1808.23	27.9421
1808.64	28.8964
1809.06	28.0377
1809.46	28.948
1809.88	24.2028
1810.29	27.6334
1810.7	25.8182
1811.11	28.6937
1811.52	31.0543
1811.93	30.1153
1812.34	28.3232
1812.75	28.4041
1813.16	31.0825
1813.57	30.6517
1813.98	26.6898
1814.39	24.8291
1814.8	28.0838
1815.21	29.9076

1815.62	30.3453
1816.04	25.9153
1816.45	30.0652
1816.86	25.6547
1817.27	30.8515
1817.68	33.5212
1818.09	29.0948
1818.5	30.5872
1818.91	26.1639
1819.32	29.136
1819.73	24.7112
1820.14	28.3838
1820.55	30.7296
1820.96	29.0878
1821.37	29.0844
1821.78	25.2849
1822.19	31.1685
1822.6	27.415
1823.01	34.6282
1823.42	28.2434
1823.83	30.5958
1824.24	30.3308
1824.65	25.0017
1825.06	26.3355
1825.47	30.9911
1825.88	30.2667
1826.29	27.1066

1826.69	25.4957
1827.1	25.8718
1827.51	29.782
1827.92	26.5948
1828.33	29.2558
1828.74	25.4629
1829.15	27.9308
1829.56	24.6937
1829.97	30.6356
1830.38	28.5407
1830.79	29.0049
1831.2	29.1816
1831.61	25.6617
1832.02	30.1246
1832.43	25.6457
1832.83	29.1512
1833.25	32.464
1833.65	27.0916
1834.06	33.1456
1834.47	29.8185
1834.88	26.1674
1835.29	25.7695
1835.7	22.6395
1836.11	24.2484
1836.52	25.9208
1836.93	27.5386
1837.33	28.1027

1837.74	26.5785
1838.15	29.8965
1838.56	29.9736
1838.97	31.3571
1839.38	27.028
1839.79	28.6289
1840.2	26.86
1840.6	28.1637
1841.01	29.651
1841.42	29.1097
1841.83	29.5963
1842.24	27.8056
1842.65	31.4708
1843.05	28.2358
1843.46	29.5617
1843.87	30.535
1844.28	25.7113
1844.69	27.6677
1845.1	28.9645
1845.51	31.1735
1845.91	30.062
1846.32	26.4912
1846.73	30.8568
1847.14	32.0383
1847.54	30.8487
1847.95	29.667
1848.36	26.5036

1848.77	25.6843
1849.18	29.6168
1849.58	27.3421
1849.99	26.1915
1850.4	26.0938
1850.81	29.5247
1851.22	25.427
1851.62	29.9505
1852.03	26.1831
1852.44	27.3953
1852.85	28.5337
1853.25	30.343
1853.66	28.361
1854.07	28.0969
1854.48	28.2264
1854.88	26.3324
1855.29	30.1005
1855.7	25.6242
1856.11	27.4015
1856.51	26.8927
1856.92	27.5183
1857.33	26.5731
1857.74	31.3487
1858.14	33.499
1858.55	26.6573
1858.96	29.136
1859.37	27.411

1859.77	26.9538
1860.18	31.0395
1860.59	29.133
1860.99	25.6597
1861.4	26.51
1861.81	28.6111
1862.22	25.3973
1862.62	28.5246
1863.03	26.6877
1863.44	21.5367
1863.84	28.4122
1864.25	25.9276
1864.66	26.0369
1865.06	26.9246
1865.47	33.0722
1865.88	27.5739
1866.28	26.6517
1866.69	27.0108
1867.1	28.6544
1867.5	25.0166
1867.91	31.302
1868.32	27.1019
1868.72	26.6878
1869.13	27.4662
1869.54	29.6411
1869.94	27.8334
1870.35	28.2856

1870.75	29.0088
1871.16	27.6415
1871.57	30.4744
1871.97	31.4563
1872.38	29.025
1872.79	27.9659
1873.19	26.5731
1873.6	24.7051
1874	22.257
1874.41	27.588
1874.82	26.7143
1875.22	27.9703
1875.63	25.9705
1876.03	26.3562
1876.44	25.4393
1876.85	25.4306
1877.25	27.7642
1877.66	30.2867
1878.06	28.7383
1878.47	31.5784
1878.88	28.5158
1879.28	28.5011
1879.69	26.2348
1880.09	28.737
1880.5	24.1602
1880.9	27.6201
1881.31	28.9648

1881.72	30.1031
1882.12	29.3368
1882.53	28.6357
1882.93	29.3855
1883.34	31.6153
1883.74	28.5901
1884.15	28.6234
1884.55	30.7675
1884.96	27.3794
1885.36	26.5613
1885.77	28.933
1886.17	29.8945
1886.58	30.5298
1886.98	28.3867
1887.39	25.5499
1887.79	27.2905
1888.2	27.6504
1888.6	29.6387
1889.01	27.5817
1889.41	25.5922
1889.82	26.5999
1890.22	27.9394
1890.63	31.2767
1891.03	29.679
1891.44	31.3826
1891.84	29.5695
1892.25	24.4351

1892.65	26.0895
1893.06	30.1678
1893.46	26.2592
1893.87	26.2596
1894.27	30.1793
1894.67	31.4797
1895.08	28.1663
1895.48	29.5109
1895.89	25.3267
1896.29	29.1708
1896.7	30.5471
1897.1	27.5938
1897.51	27.9451
1897.91	27.3135
1898.32	29.7529
1898.72	30.4678
1899.12	28.4104
1899.53	28.4089
1899.93	28.7861
1900.34	30.7854
1900.74	29.3321
1901.15	28.9835
1901.55	30.8527
1901.95	29.3857
1902.36	32.2455
1902.76	25.8173
1903.16	26.1388

1903.57	26.8737
1903.97	31.459
1904.38	25.6165
1904.78	27.5189
1905.18	25.2944
1905.59	32.7628
1905.99	28.3595
1906.4	27.1714
1906.8	31.1378
1907.2	29.9929
1907.61	28.8048
1908.01	30.0133
1908.41	34.921
1908.82	29.6984
1909.22	27.7573
1909.62	28.3657
1910.03	29.4872
1910.43	23.8474
1910.83	32.7388
1911.24	26.6849
1911.64	29.8162
1912.04	27.0758
1912.45	26.8273
1912.85	23.423
1913.25	29.1111
1913.66	30.8886
1914.06	27.8128

1914.46	28.1636
1914.87	26.5269
1915.27	28.9175
1915.67	31.629
1916.07	27.638
1916.48	27.5555
1916.88	27.5546
1917.28	29.5244
1917.69	25.8967
1918.09	30.237
1918.49	27.3219
1918.89	27.3624
1919.3	28.9683
1919.7	26.4925
1920.1	30.1405
1920.5	28.6276
1920.91	27.2784
1921.31	28.1169
1921.71	28.932
1922.12	30.032
1922.52	30.034
1922.92	29.0304
1923.32	29.159
1923.73	29.4207
1924.13	28.6135
1924.53	31.1975
1924.93	26.7797

1925.33	30.242
1925.74	28.6542
1926.14	29.6112
1926.54	25.2484
1926.94	30.2645
1927.35	27.0372
1927.75	26.0216
1928.15	25.6268
1928.55	30.25
1928.95	32.5189
1929.36	31.0989
1929.76	30.2849
1930.16	30.6065
1930.56	29.529
1930.96	29.3708
1931.36	28.6632
1931.77	29.3913
1932.17	29.6165
1932.57	29.3915
1932.97	28.7685
1933.37	27.5212
1933.78	26.6807
1934.18	28.7786
1934.58	32.2138
1934.98	26.1506
1935.38	30.1474
1935.78	28.186

1936.18	28.8652
1936.59	26.2841
1936.99	24.0107
1937.39	27.4058
1937.79	30.7442
1938.19	31.0118
1938.59	28.8227
1938.99	29.9266
1939.4	31.483
1939.8	30.7036
1940.2	31.1103
1940.6	28.8245
1941	29.6101
1941.4	26.324
1941.8	26.1155
1942.2	27.7308
1942.6	28.5085
1943.01	27.3583
1943.41	28.1573
1943.81	24.9843
1944.21	28.6003
1944.61	25.6638
1945.01	27.9844
1945.41	27.3285
1945.81	31.0444
1946.21	29.4486
1946.61	27.6494

1947.01	29.4438
1947.41	31.5079
1947.81	32.9101
1948.22	25.5732
1948.62	30.3588
1949.02	30.2867
1949.42	32.0802
1949.82	32.5341
1950.22	28.8835
1950.62	26.9063
1951.02	26.323
1951.42	28.5191
1951.82	29.2624
1952.22	26.347
1952.62	28.1828
1953.02	27.0797
1953.42	27.1154
1953.82	28.7217
1954.22	24.0287
1954.62	29.5882
1955.02	30.4107
1955.42	30.7179
1955.82	29.7305
1956.22	31.0195
1956.62	27.8262
1957.02	28.2334
1957.42	31.7799

1957.82	26.6317
1958.22	27.6755
1958.62	31.7818
1959.02	33.4967
1959.42	22.3605
1959.82	27.1503
1960.22	32.0225
1960.62	27.4472
1961.02	32.5285
1961.42	28.615
1961.82	29.9764
1962.22	28.6487
1962.62	23.6692
1963.02	26.312
1963.42	29.2341
1963.81	23.1754
1964.21	28.261
1964.61	31.8652
1965.01	24.4323
1965.41	23.8832
1965.81	26.771
1966.21	22.3464
1966.61	23.7163
1967.01	22.2382
1967.41	25.0741
1967.81	28.584
1968.21	29.3395

1968.61	25.7101
1969	25.1284
1969.4	33.3935
1969.46	34.5211
1969.86	26.9425
1970.26	25.2637
1970.66	28.8027
1971.07	29.1047
1971.47	27.2026
1971.87	26.8032
1972.27	28.5703
1972.67	26.7328
1973.07	33.3261
1973.48	27.1438
1973.88	26.9175
1974.28	30.3522
1974.68	24.5997
1975.08	30.0875
1975.48	27.0204
1975.89	23.6434
1976.29	28.2585
1976.69	28.9852
1977.09	27.4818
1977.49	26.9108
1977.89	25.2284
1978.29	27.6181
1978.7	25.2587

1979.1	25.5661
1979.5	27.6062
1979.9	30.1211
1980.3	32.9317
1980.7	29.6563
1981.1	29.2318
1981.5	21.9833
1981.9	27.5577
1982.3	24.3429
1982.71	26.2355
1983.11	28.2909
1983.51	29.3021
1983.91	28.2382
1984.31	28.2125
1984.71	28.7645
1985.11	28.5772
1985.51	24.7276
1985.91	28.9215
1986.31	27.3018
1986.71	27.4521
1987.11	23.6537
1987.51	25.0135
1987.92	26.0992
1988.32	27.874
1988.72	25.9389
1989.12	24.2644
1989.52	25.8891

1989.92	28.2018

FEB02

1990.32	26.1755
1990.72	24.494
1991.12	24.1571
1991.52	20.0126
1991.92	28.3467
1992.32	28.4159
1992.72	25.9807
1993.12	27.52
1993.52	26.917
1993.92	26.3463
1994.32	27.1206
1994.72	28.0786
1995.12	29.2888
1995.52	27.7852
1995.92	27.7305
1996.32	26.4663
1996.72	24.2149
1997.12	26.6345
1997.52	25.3917
1997.92	26.5719
1998.32	22.3419
1998.72	28.317
1999.12	26.1041
1999.52	27.4468
1999.92	24.2424

FEC 01

100.25	31
100.77	31.0072
101.289	33.0144
101.809	27.0216
102.329	25.0288
102.847	29.036
103.366	33.0432
103.886	33.0504
104.406	34.7239
104.925	30.8033
105.445	32.752
105.964	31.3294
106.484	32.564
107.001	34.5295
107.521	35.703
108.04	37.0559
108.56	35.0459
109.079	29.1066
109.598	31.9953
110.116	34.3725
110.635	32.1115
111.154	30.2109
111.674	32.8191
112.193	31.4329
112.71	29.9839
113.229	31.9009

113.748	35.4823
114.267	33.1302
114.784	32.7259
115.303	31.5013
115.822	30.9007
116.341	36.5803
116.858	36.6456
117.377	31.4612
117.896	35.7783
118.415	34.9036
118.931	33.0078
119.45	30.5844
119.969	28.3883
120.486	32.4967
121.004	29.1879
121.523	35.7102
122.042	33.0978
122.558	32.2154
123.077	35.0519
123.596	34.4279
124.112	31.3426
124.63	31.9511
125.149	30.6108
125.665	32.1252
126.184	34.1226
126.702	30.0109
127.218	35.582

127.737	30.5127
128.253	29.4171
128.771	33.1939
129.29	28.2465
129.806	31.1698
130.324	30.6572
130.84	29.3178
131.358	36.2267
131.876	32.5464
132.392	35.1458
132.91	35.3193
133.426	29.1486
133.944	28.894
134.46	30.0872
134.978	31.0055
135.496	31.2687
136.012	30.4683
136.53	28.4109
137.045	28.5766
137.563	32.7533
138.079	32.6777
138.597	33.5026
139.112	34.7799
139.63	32.225
140.145	36.9708
140.663	28.5135
141.178	33.5175

141.696	33.6804
142.211	32.1123
142.729	29.9122
143.244	30.3968
143.76	34.1587
144.277	32.2672
144.792	34.1909
145.31	31.9064
145.825	32.6528
146.342	32.9462
146.858	32.2962
147.375	30.7052
147.89	32.3562
148.405	32.6234
148.922	35.3346
149.437	31.8861
149.954	33.3659
150.469	33.7218
150.984	30.6918
151.501	34.4145
152.016	33.376
152.531	29.763
153.048	35.9729
153.563	34.7473
154.078	32.7619
154.594	35.4715
155.109	33.8628

155.624	33.0812
156.141	30.8708
156.655	29.8556
157.17	30.8158
157.687	29.861
158.201	31.2441
158.716	32.2507
159.232	33.7643
159.747	35.8433
160.261	34.9473
160.776	33.1809
161.292	35.8162
161.806	34.799
162.321	37.7015
162.835	33.333
163.351	34.788
163.866	34.1977
164.38	33.6227
164.894	37.5913
165.41	34.8147
165.924	35.024
166.438	40.3733
166.953	41.4294
167.467	37.1444
167.983	38.5269
168.497	35.6842
169.011	35.4246

169.525	37.4898
170.038	34.0998
170.554	34.8948
171.068	32.3824
171.582	32.2278
172.096	32.5951
172.61	33.2155
173.123	32.2026
173.637	38.1574
174.153	33.3872
174.666	29.2561
175.18	31.4135
175.694	36.558
176.207	36.5611
176.721	34.8461
177.234	36.9556
177.748	31.98
178.261	34.3082
178.777	32.102
179.29	32.2171
179.803	37.4112
180.317	37.6656
180.83	34.9632
181.343	36.7195
181.856	37.1749
182.37	32.9973
182.883	38.5672

183.396	35.0186
183.909	36.0875
184.422	35.1988
184.935	35.4508
185.448	32.7006
185.961	33.9298
186.474	37.2624
186.987	36.3482
187.5	34.523
188.013	30.7933
188.526	35.5175
189.039	32.5011
189.552	33.8428
190.064	36.567
190.577	33.7027
191.09	33.6519
191.603	37.5698
192.115	32.4
192.628	31.7379
193.138	33.8206
193.651	35.6019
194.164	35.5414
194.676	32.6314
195.189	36.7035
195.701	35.7645
196.214	33.6717
196.726	33.6194

197.238	33.6704
197.749	38.6162
198.261	37.811
198.773	35.6604
199.286	35.7542
199.798	33.8357
200.31	35.5821
200.822	33.5663
201.332	36.7701
201.844	36.6236
202.357	34.6421
202.869	35.9929
203.381	36.1344
203.893	36.019
204.403	36.4808
204.915	31.8251
205.427	36.6076
205.938	38.7471
206.448	31.7636
206.96	31.967
207.472	38.9358
207.984	30.4492
208.493	34.4524
209.005	34.0672
209.517	35.2782
210.029	33.8646
210.538	37.4057

211.05	40.7796
211.561	32.6461
212.073	38.4819
212.582	33.0749
213.094	31.8112
213.605	34.9713
214.115	33.9451
214.626	37.8718
215.138	38.1695
215.647	32.4064
216.158	32.2612
216.669	38.551
217.179	32.7507
217.69	36.0618
218.201	40.124
218.71	35.5219
219.221	34.3296
219.733	33.6481
220.242	38.3605
220.753	37.2013
221.262	38.0232
221.773	32.1209
222.284	35.935
222.793	39.88
223.304	38.4193
223.812	35.4559
224.323	31.7803

224.834	39.5621
225.343	36.8376
225.854	35.8244
226.362	39.1427
226.873	34.8713
227.382	38.5294
227.893	36.7795
228.401	38.4721
228.912	41.5722
229.422	44.0343
229.931	38.0618
230.441	38.0894
230.95	44.6933
231.46	43.2819
231.969	47.3884
232.479	47.2328
232.987	44.7121
233.496	47.369
234.006	42.79
234.514	46.6751
235.025	49.3544
235.533	46.1838
236.043	40.5184
236.551	46.9489
237.061	43.8023
237.57	40.0472
238.08	44.8998

238.588	45.3913
239.096	43.4518
239.606	40.0413
240.114	37.0853
240.624	42.7396
241.132	41.5976
241.639	38.4218
242.149	38.6833
242.657	42.333
243.165	38.7318
243.675	42.0171
244.182	39.4297
244.692	39.1136
245.2	41.5759
245.707	42.3439
246.217	40.2504
246.725	39.8399
247.232	42.9607
247.742	42.2874
248.249	45.7014
248.757	39.6254
249.264	38.1926
249.774	38.7052
250.281	43.2433
250.788	40.988
251.298	46.4636
251.805	42.101

252.312	39.1156
252.82	38.2367
253.329	40.1765
253.836	38.8497
254.343	43.3956
254.85	41.0241
255.36	37.5863
255.867	34.0774
256.374	32.1841
256.881	36.1814
257.388	37.3585
257.897	38.8523
258.404	37.8159
258.911	38.5975
259.418	37.7201
259.924	33.3755
260.433	32.8888
260.94	38.8137
261.447	39.8532
261.954	33.3628
262.46	33.5756
262.967	35.5295
263.476	36.973
263.982	39.5668
264.489	35.2986
264.996	38.2834
265.502	40.2

266.009	36.1872
266.515	38.207
267.022	35.6349
267.528	34.5757
268.036	32.5248
268.543	31.1365
269.049	29.7768
269.555	34.3015
270.062	37.0524
270.568	36.0144
271.074	37.0767
271.58	35.0923
272.087	35.8071
272.593	32.9677
273.099	34.6039
273.605	35.4954
274.111	36.8277
274.617	31.9562
275.123	34.4884
275.629	34.2317
276.135	34.7067
276.641	36.6973
277.147	28.4994
277.653	31.2178
278.159	34.4173
278.665	33.1614
279.17	38.4219

279.676	34.4227
280.182	35.7601
280.688	36.4977
281.193	35.1622
281.699	39.0516
282.204	35.8328
282.71	32.4829
283.216	33.1283
283.721	35.8091
284.225	35.1391
284.73	33.1886
285.236	37.7785
285.741	37.3322
286.246	40.2184
286.752	35.9687
287.257	39.6221
287.762	38.1376
288.266	37.8035
288.771	34.2013
289.276	35.5408
289.781	36.4669
290.287	39.4223
290.792	38.376
291.295	36.3572
291.8	34.1319
292.305	35.7827
292.81	34.0837

293.315 39.1692

293.82 36.2573

294.323 39.9467

294.828 38.063

295.333 38.7224

295.838 41.1362

296.34 35.4241

296.845 37.6662

297.35 38.4984

297.855 36.499

298.358 37.296

298.862 40.7952

299.367 37.2534

299.872 40.6221

300.374 40.0209

300.879 45.038

301.383 45.6812

301.888 41.2931

302.39 40.7044

302.895 41.1366

303.399 40.1973

303.902 38.5363

304.406 41.2499

304.91 39.6513

305.413 34.3254

305.917 40.7839

306.421 37.8689

306.924	38.1602
307.428	35.6425
307.932	35.7343
308.434	39.8448
308.938	39.4326
309.44	35.1848
309.944	32.9512
310.449	30.3525
310.951	34.3483
311.455	33.267
311.957	38.8218
312.46	35.4806
312.964	33.0035
313.466	34.7358
313.97	34.2072
314.472	32.6034
314.976	37.2855
315.478	38.3019
315.981	33.2647
316.483	33.5135
316.987	34.5746
317.488	35.6919
317.992	39.2602
318.496	36.3194
318.997	34.7893
319.501	39.1224
320.002	35.3993

320.506	31.3534
321.007	36.0405
321.509	37.9985
322.012	36.4614
322.513	36.6614
323.017	34.4745
323.518	36.9183
324.021	34.7634
324.523	34.1078
325.026	37.7174
325.527	34.6819
326.03	31.2627
326.532	31.1607
327.033	37.8953
327.536	38.8931
328.037	34.9286
328.54	32.7032
329.041	33.4624
329.542	35.7785
330.045	34.6119
330.546	37.4509
331.047	39.1866
331.55	38.517
332.051	35.4693
332.553	34.4911
333.054	36.8376
333.555	36.28

334.058	36.4086
334.558	35.8673
335.059	35.0092
335.562	36.9789
336.062	34.9774
336.563	35.2751
337.064	42.1084
337.566	43.7174
338.067	38.3896
338.567	35.1098
339.07	31.8787
339.57	33.9177
340.07	37.5334
340.571	39.6832
341.073	35.8249
341.574	32.2135
342.074	36.3242
342.574	33.9676
343.076	33.0094
343.577	39.206
344.077	35.0272
344.577	36.4581
345.077	41.8219
345.579	36.7908
346.079	31.3085
346.579	39.9207
347.079	36.0562

347.579	39.997
348.081	35.3737
348.581	36.8868
349.081	35.1182
349.581	33.214
350.081	34.3736
350.581	44.2303
351.081	39.8186
351.582	33.4283
352.082	35.6971
352.582	40.2733
353.082	34.3913
353.581	48.0373
354.081	38.8261
354.58	38.8122
355.08	37.0301
355.58	36.9573
356.081	38.901
356.581	40.7565
357.08	38.8543
357.58	38.662
358.079	37.0285
358.578	34.8842
359.078	31.0883
359.577	40.6153
360.076	33.1794
360.576	33.0156

361.075	32.9257
361.574	32.9222
362.073	36.6959
362.573	35.0559
363.072	38.6493
363.105	38.9096
363.608	42.9094
364.109	36.9073
364.612	40.9015
365.114	36.911
365.617	36.8733
366.118	42.8503
366.621	42.8136
367.124	38.919
367.625	32.9194
368.128	36.9451
368.629	36.9729
369.132	34.9489
369.633	36.9231
370.136	40.9464
370.636	38.8982
371.139	36.8577
371.64	42.7339
372.143	44.7171
372.643	40.841
373.146	33.1234
373.647	36.9889

374.15	40.9137
374.65	46.7381
375.153	42.8261
375.653	46.7234
376.154	39.0099
376.656	46.8103
377.157	39.2144
377.659	42.9046
378.16	48.3577
378.662	44.5648
379.162	40.555
379.663	37.1497
380.165	33.5365
380.665	46.3948
381.168	48.3723
381.668	46.8971
382.168	48.3597
382.67	53.0163
383.17	48.3601
383.671	47.029
384.173	51.3428
384.673	57.848
385.173	54.3924
385.675	55.9237
386.175	49.0383
386.675	48.7641
387.177	47.2355

387.677	50.9083
388.176	50.7406
388.678	53.8206
389.178	52.9689
389.678	54.7332
390.18	47.5171
390.679	52.5483
391.179	52.486
391.679	41.2173
392.18	57.7174
392.68	42.5482
393.18	48.8664
393.679	41.0235
394.181	37.5687
394.68	43.1018
395.18	42.9514
395.679	48.7645
396.181	49.1068
396.68	50.2993
397.179	44.4991
397.679	42.8492
398.178	46.1696
398.679	41.7386
399.179	42.4643
399.678	44.4313
400.177	44.413
400.676	43.4723

401.175	45.0535
401.677	48.5366
402.176	44.5489
402.675	41.5565
403.174	45.8103
403.673	38.0002
404.172	45.8761
404.671	45.0595
405.172	40.9988
405.671	42.1323
406.169	37.1271
406.668	39.3523
407.167	41.0108
407.666	48.0001
408.165	39.2481
408.663	43.1815
409.162	35.6891
409.661	39.6087
410.162	37.974
410.66	42.4407
411.159	41.2249
411.657	42.8088
412.156	39.2727
412.654	44.6054
413.153	39.3504
413.651	43.0499
414.15	43.215

414.648	44.045
415.147	36.1508
415.645	42.6675
416.143	42.7223
416.642	41.0747
417.14	36.3619
417.638	40.3945
418.136	37.8486
418.635	43.2858
419.133	42.4395
419.631	36.2117
420.129	37.5175
420.627	38.9083
421.123	35.5498
421.621	43.1078
422.119	38.4458
422.617	36.4379
423.115	36.6553
423.613	40.8498
424.111	40.0692
424.609	37.8618
425.107	43.6873
425.605	39.1496
426.102	38.0227
426.598	37.5306
427.096	41.9301
427.594	35.7809

428.091	44.1139
428.589	41.0941
429.086	37.7265
429.584	41.2787
430.08	41.452
430.577	36.9815
431.075	36.9132
431.572	37.1895
432.07	39.5003
432.567	41.6868
433.062	38.3461
433.56	42.8432
434.057	34.3209
434.555	35.4296
435.052	38.8998
435.547	36.5467
436.044	42.124
436.542	41.8369
437.039	35.6494
437.534	38.449
438.031	35.7511
438.528	42.8783
439.025	36.5166
439.52	41.0394
440.017	42.4536
440.514	39.7364
441.011	37.9604

441.506	43.0014
442.003	32.3726
442.5	45.7984
442.995	37.3353
443.492	36.873
443.989	39.485
444.483	38.9976
444.98	37.8934
445.477	40.3762
445.971	38.1429
446.468	41.8342
446.965	40.8121
447.459	42.7436
447.956	36.7026
448.453	35.8225
448.947	34.3211
449.444	32.4168
449.938	39.2938
450.435	41.0248
450.931	36.967
451.425	42.5335
451.922	38.3229
452.416	38.1757
452.912	39.9211
453.409	45.2126
453.903	36.4179
454.399	42.7038

454.893	44.1078
455.39	42.1486
455.884	40.8578
456.38	44.7664
456.874	41.3523
457.37	35.1245
457.864	43.4884
458.36	43.5241
458.856	47.9046
459.35	40.7039
459.846	39.5297
460.34	41.9407
460.834	40.3992
461.33	39.3507
461.824	41.6223
462.319	46.0755
462.813	41.6098
463.309	38.8475
463.803	42.0648
464.298	45.7281
464.792	43.728
465.288	44.0185
465.781	46.7917
466.277	42.4593
466.77	50.7437
467.264	46.1578
467.759	50.0445

468.253	50.5421
468.748	52.5215
469.242	46.7476
469.735	49.8127
470.231	48.0793
470.724	47.1753
471.217	48.839
471.713	44.9252
472.206	48.8706
472.701	48.7356
473.194	48.0848
473.687	45.6844
474.183	44.8892
474.676	41.4319
475.169	44.1126
475.664	43.8302
476.157	44.9643
476.65	45.8986
477.143	41.9222
477.638	41.8651
478.131	45.0483
478.624	39.5461
479.119	39.7116
479.612	42.6202
480.104	38.6203
480.597	39.2079
481.092	40.2434

481.585	38.9015
482.078	44.8352
482.57	37.697
483.065	42.098
483.558	40.6789
484.05	40.8134
484.543	39.3798
485.035	41.0675
485.53	42.5974
486.022	41.0109
486.515	39.8919
487.007	36.6081
487.5	40.9618
487.994	37.1696
488.487	38.8732
488.979	39.1125
489.471	37.0521
489.964	44.1153
490.456	39.7671
490.95	36.754
491.442	40.7275
491.934	40.7753
492.427	40.595
492.919	34.4443
493.411	40.5116
493.903	38.2911
494.395	39.84

494.887	36.2895
495.381	37.8612
495.873	39.2048
496.365	35.6331
496.857	33.8234
497.349	36.1093
497.841	40.5556
498.333	34.9257
498.824	38.4226
499.316	40.4189
499.808	35.3993
500.3	39.8025
500.791	40.7804
501.283	37.6213
501.775	43.7048
502.266	40.3778
502.758	39.6771
503.25	38.0601
503.741	40.4091
504.233	38.773
504.724	39.4593
505.216	39.971
505.707	39.0811
506.198	42.2479
506.69	41.2528
507.181	40.3022
507.673	36.263

508.164	38.6484
508.655	35.4916
509.146	36.6579
509.638	36.8964
510.129	41.899
510.62	41.9018
511.109	39.7359
511.6	39.186
512.091	42.7232
512.582	44.1688
513.074	41.7984
513.565	42.315
514.056	39.4905
514.547	37.9235
515.035	39.9895
515.526	40.6562
516.017	43.1173
516.508	43.9485
516.999	39.525
517.49	39.5074
517.979	40.4837
518.469	39.4137
518.96	41.5796
519.451	42.3121
519.941	40.7364
520.432	38.473
520.921	37.8486

521.411	40.3781
521.902	37.3662
522.392	38.9094
522.881	39.0737
523.371	41.5339
523.862	40.3878
524.352	40.8469
524.841	39.2947
525.331	42.4419
525.821	42.0095
526.312	42.9872
526.8	37.1
527.29	39.9488
527.781	39.3565
528.269	45.1816
528.759	44.9448
529.249	39.7408
529.739	42.3259
530.227	46.6809
530.718	41.1132
531.208	39.7364
531.696	36.4597
532.186	40.5555
532.676	41.924
533.164	45.0447
533.654	38.4742
534.141	46.9289

534.631	39.0863
535.121	40.554
535.609	43.0321
536.099	42.429
536.589	38.8601
537.076	40.2335
537.566	43.4019
538.054	41.9705
538.543	39.8313
539.033	36.0188
539.521	39.5392
540.01	45.3549
540.498	38.1584
540.987	38.1659
541.475	42.7881
541.964	39.287
542.452	39.2817
542.941	42.8529
543.429	36.743
543.918	46.1574
544.405	40.0253
544.895	34.1649
545.382	37.9801
545.871	41.3922
546.359	40.7356
546.848	38.3174
547.335	42.5176

547.824	35.5685
548.311	36.7663
548.8	37.434
549.287	41.5228
549.777	36.9572
550.264	41.2134
550.753	43.0328
551.24	37.3038
551.727	36.9524
552.215	41.9175
552.702	41.5975
553.191	41.3664
553.678	38.6213
554.167	39.4147
554.654	44.6454
555.141	34.8369
555.629	39.6457
556.116	37.4048
556.603	41.839
557.091	35.7858
557.578	41.8949
558.065	40.5147
558.553	39.8354
559.04	36.5846
559.526	37.436
560.015	35.4328
560.501	42.175

560.988	41.7283
561.476	41.3324
561.963	38.0457
562.449	39.0619
562.937	36.1347
563.424	37.7536
563.91	42.7015
564.396	39.2764
564.885	37.9876
565.371	39.0746
565.857	37.66
566.345	38.3174
566.831	34.1836
567.317	37.8761
567.803	38.7421
568.289	33.0096
568.778	38.9378
569.264	40.1011
569.75	38.0745
570.236	36.3385
570.723	43.7046
571.209	44.2844
571.695	45.8543
572.181	38.3554
572.667	39.7386
573.153	41.2361
573.641	38.2575

574.126	36.576
574.612	39.1082
575.098	38.0806
575.583	35.1709
576.069	37.9232
576.555	35.8983
577.042	37.8889
577.528	38.3894
578.013	43.588
578.499	39.863
578.984	33.3378
579.47	30.1144
579.955	33.5484
580.441	40.2782
580.926	41.4233
581.411	38.4969
581.897	40.7902
582.384	42.5469
582.869	39.5683
583.355	34.3014
583.84	34.4417
584.325	39.5563
584.81	45.8207
585.295	44.4078
585.781	38.9967
586.266	38.79
586.751	41.4996

587.236	44.0301
587.721	44.5081
588.206	41.5542
588.691	36.6208
589.176	41.8995
589.661	42.9193
590.146	39.1604
590.63	37.6056
591.115	40.7765
591.598	37.6733
592.083	35.3454
592.568	38.354
593.052	42.705
593.537	43.5117
594.022	41.1381
594.506	38.0095
594.991	34.6875
595.476	35.425
595.96	39.2198
596.445	40.4943
596.929	40.4855
597.412	40.519
597.896	46.158
598.381	42.5457
598.865	39.5899
599.35	42.4921
599.834	43.1042

600.318	40.7112
600.801	47.6235
601.285	45.6513
601.769	47.0274
602.254	47.9436
602.738	42.0133
603.22	45.6007
603.704	42.7122
604.188	46.9397
604.672	49.2233
605.156	44.2779
605.639	38.5451
606.123	45.8536
606.607	44.4539
607.091	43.8941
607.573	42.4576
608.057	43.435
608.54	38.8517
609.024	40.9967
609.506	43.7953
609.99	41.8209
610.474	49.5827
610.958	44.4361
611.439	43.9259
611.923	37.7333
612.407	31.6961
612.888	43.8234

613.372	34.995
613.856	40.5343
614.337	40.5513
614.821	44.1456
615.305	38.7146
615.786	44.1647
616.269	36.6627
616.753	44.2469
617.234	34.5408
617.718	44.4218
618.199	46.3836
618.201	46.4045
618.687	46.4045
619.174	42.4045
619.659	44.4023
620.145	42.4017
620.63	42.4045
621.115	46.3893
621.602	40.4304
622.088	36.5098
622.573	42.3693
623.058	44.4453
623.543	42.4604
624.028	48.2516
624.513	40.3647
625	50.2299
625.485	42.3521

625.97	36.4347
626.455	46.2427
626.94	38.6349
627.425	44.2731
627.91	42.2598
628.395	46.3232
628.88	38.7503
629.365	32.6616
629.852	47.9941
630.336	34.9262
630.821	40.4367
631.306	44.2179
631.79	44.0513
632.275	46.0365
632.76	38.5767
633.244	48.1926
633.729	38.5086
634.214	36.2439
634.698	49.715
635.183	47.9604
635.667	42.5926
636.151	48.2811
636.636	49.7529
637.12	40.4831
637.605	48.2699
638.089	44.0094
638.573	40.4566

639.058	38.6173
639.542	40.4657
640.026	40.622
640.51	40.4664
640.995	38.8275
641.479	39.5238
641.963	38.6436
642.445	41.9052
642.929	40.8461
643.413	39.4381
643.897	44.2808
644.381	42.4709
644.865	50.2475
645.349	44.2835
645.833	46.0452
646.317	46.0385
646.801	41.3765
647.283	37.7837
647.767	44.7245
648.25	44.061
648.734	45.5462
649.218	44.4226
649.702	45.5633
650.185	40.9165
650.667	41.8284
651.151	40.8918
651.634	42.9824

652.118	49.2526
652.602	45.4448
653.083	47.5981
653.567	44.4546
654.05	49.4081
654.534	37.3509
655.017	38.8293
655.499	41.9574
655.982	49.7027
656.465	46.4989
656.949	39.7205
657.43	48.8766
657.913	38.7556
658.397	49.9358
658.88	49.9275
659.361	45.9926
659.844	47.7782
660.327	48.9897
660.81	44.0956
661.292	47.2274
661.775	44.0834
662.258	44.1631
662.739	42.5125
663.222	42.7775
663.705	43.9846
664.186	38.6149
664.669	54.5346

665.152	46.3546
665.633	38.557
666.115	45.2794
666.598	50.6787
667.079	46.6962
667.562	49.7164
668.045	46.6674
668.525	51.187
669.008	47.4482
669.489	46.7891
669.971	44.9507
670.454	49.0111
670.935	44.11
671.417	45.9549
671.898	51.8575
672.38	42.1461
672.861	48.8675
673.343	45.9489
673.826	50.1708
674.306	39.2524
674.788	43.7812
675.269	43.932
675.751	49.9644
676.231	48.2051
676.714	33.5402
677.194	46.6581
677.676	43.3814

678.156	44.5907
678.639	43.7718
679.119	50.789
679.601	46.5529
680.081	46.5949
680.563	44.8276
681.043	41.2704
681.525	42.0519
682.005	43.7855
682.487	45.0098
682.967	46.7336
683.449	44.2845
683.929	47.5026
684.409	42.8347
684.891	41.338
685.371	44.8028
685.852	44.1319
686.332	49.1032
686.814	49.8303
687.294	46.4865
687.773	45.6746
688.255	42.3324
688.735	47.6666
689.216	50.1303
689.696	48.5495
690.176	47.9782
690.657	50.4621

691.137	43.5107
691.616	43.5981
692.098	43.9027
692.577	47.5949
693.057	43.3383
693.538	48.797
694.017	46.9679
694.497	46.1549
694.978	40.2221
695.457	43.8132
695.937	46.0024
696.416	46.5311
696.897	40.0358
697.376	44.9649
697.855	45.8563
698.336	43.9421
698.816	43.1323
699.295	47.4117
699.774	46.3675
700.255	44.3817
700.734	46.4085
701.213	43.7559
701.692	46.43
702.173	44.2797
702.652	46.5474
703.131	46.3686
703.609	44.9843

704.088	45.574
704.569	45.0722
705.048	44.7087
705.527	41.4614
706.005	42.1842
706.484	42.5398
706.963	46.2732
707.443	50.4328
707.922	46.624
708.401	40.8883
708.879	45.211
709.358	44.2658
709.836	43.745
710.315	40.8904
710.795	43.8902
711.274	46.439
711.752	45.4923
712.231	43.1461
712.709	47.3113
713.187	49.3642
713.666	48.949
714.144	41.3735
714.622	47.3168
715.101	44.0537
715.579	41.4389
716.059	43.3424
716.537	48.728

717.015	44.0792
717.494	40.6704
717.972	39.8848
718.45	51.0104
718.928	45.7971
719.406	44.5655
719.884	41.0159
720.362	49.7825
720.84	45.0941
721.318	41.4617
721.796	45.0876
722.273	43.821
722.751	48.2149
723.229	39.5992
723.707	37.9887
724.185	46.5338
724.662	44.3326
725.138	43.045
725.616	45.1408
726.094	46.0984
726.571	39.4494
727.049	39.084
727.526	46.5248
728.004	43.5035
728.482	41.9087
728.959	43.6244
729.437	43.7176

729.914	43.4125
730.392	45.4094
730.867	47.4053
731.344	43.4943
731.822	43.6756
732.299	43.3523
732.776	42.5092
733.254	42.2076
733.731	47.0695
734.206	45.6829
734.683	44.3321
735.161	45.2826
735.638	41.9515
736.115	44.3231
736.59	46.2558
737.067	42.1447
737.544	40.308
738.021	43.6803
738.498	43.0847
738.973	42.6261
739.45	43.9242
739.927	39.0527
740.404	41.3193
740.879	41.5982
741.356	42.6202
741.833	40.3169
742.31	38.0529

742.784	39.3416
743.261	42.0456
743.738	40.2727
744.215	41.6087
744.689	42.0265
745.166	41.9967
745.643	40.6984
746.117	40.0222
746.594	39.9494
747.07	42.3264
747.545	40.739
748.022	40.1829
748.498	43.4142
748.973	41.1377
749.449	42.5176
749.925	43.8447
750.4	41.2802
750.876	44.7614
751.352	44.3977
751.827	43.5411
752.303	43.831
752.779	43.7024
753.254	43.1143
753.73	41.7373
754.204	42.3509
754.68	45.2069
755.154	37.6672

755.63	38.7075
756.107	42.0975
756.581	41.0155
757.057	40.0226
757.531	42.3439
758.007	40.0421
758.481	42.0626
758.957	44.0561
759.431	41.2781
759.906	40.8383
760.382	46.6405
760.856	41.6765
761.332	39.7241
761.806	42.0507
762.282	44.2478
762.755	44.4696
763.229	43.8368
763.705	42.7362
764.178	40.9091
764.654	37.239
765.128	40.0854
765.603	43.6878
766.077	41.1122
766.552	41.0566
767.026	44.347
767.501	43.5018
767.975	46.0904

768.448	41.7628
768.924	38.5526
769.397	37.2993
769.873	40.9443
770.346	44.2989
770.819	41.8868
771.295	44.5508
771.768	40.089
772.243	40.0432
772.716	39.6569
773.19	38.2737
773.665	40.3199
774.138	44.6634
774.611	38.6458
775.086	38.9571
775.559	43.9275
776.032	41.2648
776.507	37.0937
776.98	43.8965
777.453	45.2026
777.928	42.8547
778.401	44.9897
778.874	41.3908
779.347	40.2932
779.822	43.1113
780.295	40.943
780.767	42.4197

781.242	39.3183
781.715	39.3604
782.188	42.4296
782.66	42.8641
783.135	41.8123
783.608	42.6473
784.08	44.3938
784.553	42.207
785.026	38.9811
785.5	46.2135
785.973	40.6138
786.445	44.8928
786.918	39.3995
787.39	42.3755
787.864	38.4276
788.337	44.8969
788.809	38.9266
789.282	35.4603
789.754	38.8463
790.226	40.0511
790.701	41.1638
791.173	38.8583
791.645	43.4194
792.117	42.0196
792.589	39.9995
793.062	36.7658
793.534	41.2161

794.006	42.6473
794.478	42.0862
794.952	44.6412
795.424	41.5589
795.896	48.9028
796.368	43.6651
796.84	41.8265
797.312	46.8153
797.784	48.1134
798.256	44.2643
798.728	42.3948
799.199	41.8421
799.671	38.6197
800.143	39.5568
800.615	38.6463
801.087	42.027
801.558	43.8458
802.03	40.3123
802.502	44.9713
802.973	41.8472
803.445	41.8913
803.917	40.8985
804.388	43.4168
804.86	42.2588
805.331	43.1341
805.803	45.1375
806.274	39.4315

806.746	45.9577
807.217	40.5408
807.688	40.5296
808.16	38.0418
808.631	39.8976
809.102	41.5219
809.572	39.8451
810.043	43.5595
810.514	42.0668
810.986	43.4898
811.457	41.8045
811.928	44.26
812.399	43.4085
812.87	45.4228
813.341	43.4455
813.81	39.9743
814.281	39.3542
814.752	38.7057
815.223	40.9488
815.694	44.8096
816.165	42.2052
816.634	40.6315
817.105	44.4934
817.576	44.4323
818.047	42.8144
818.518	41.1865
818.987	37.0505

819.457	42.4747
819.928	46.6561
820.399	44.0729
820.87	43.0812
821.338	39.1507
821.809	45.136
822.28	41.8759
822.75	40.6673
823.219	39.9946
823.689	39.9498
824.16	39.9627
824.628	40.581
825.099	41.3635
825.569	40.9921
826.04	44.5369
826.508	46.9873
826.979	43.5304
827.449	45.6848
827.917	47.7898
828.388	44.2558
828.858	45.9417
829.326	46.3765
829.797	42.9614
830.267	40.6326
830.735	37.4421
831.205	38.2616
831.675	42.9701

832.143	40.5898
832.614	38.749
833.082	43.8312
833.552	43.9545
834.022	40.6684
834.49	40.8511
834.96	41.053
835.428	44.1281
835.898	42.5797
836.367	38.8385
836.835	37.696
837.305	41.1254
837.773	42.5481
838.243	38.281
838.711	43.7566
839.18	43.5961
839.648	45.2342
840.118	42.1298
840.586	40.5971
841.055	42.11
841.523	44.0125
841.992	43.3878
842.46	41.4629
842.93	44.5588
843.397	41.3431
843.867	42.8893
844.334	43.9279

844.804	42.2175
845.271	42.4694
845.741	46.8693
846.208	45.483
846.677	41.2046
847.145	46.9418
847.612	45.4261
848.081	49.1435
848.549	44.7426
849.018	38.5116
849.485	39.3362
849.954	40.5185
850.421	40.4946
850.889	38.7025
851.358	38.8674
851.825	39.6925
852.292	42.8895
852.761	35.9878
853.228	40.7954
853.695	43.2886
854.164	45.5915
854.631	39.8718
855.1	37.5429
855.567	41.8776
856.034	37.9657
856.501	46.5249
856.969	46.274

857.436	43.6163
857.903	37
858.372	40.9837
858.839	43.8907
859.305	37.4243
859.774	46.2643
860.241	42.3376
860.707	38.974
861.174	44.8822
861.621	50.5341
862.091	40.6225
862.56	40.652
863.03	40.5932
863.501	42.5373
863.971	38.6274
864.442	44.468
864.912	44.4243
865.38	42.5019
865.851	44.4493
866.321	46.3562
866.791	44.4838
867.262	36.6136
867.732	40.4361
868.2	44.4804
868.67	40.5302
869.14	44.2058
869.61	48.1576

870.081	40.4738
870.549	36.6605
871.019	36.8362
871.489	38.7564
871.959	44.5408
872.427	42.739
872.897	44.4799
873.367	38.5711
873.836	46.229
874.304	44.5362
874.774	42.5013
875.244	36.7206
875.712	36.9469
876.182	42.815
876.651	42.464
877.121	42.6301
877.589	42.9086
878.058	48.4946
878.528	42.3315
878.996	36.9074
879.465	44.0352
879.935	42.4501
880.403	40.4625
880.872	37.5836
881.342	40.72
881.809	44.3398
882.279	46.0443

882.746	38.8772
883.215	47.8314
883.685	46.1623
884.152	33.4161
884.621	43.8422
885.089	42.5381
885.558	44.3611
886.027	39.0746
886.495	40.3207
886.964	38.6403
887.431	46.5565
887.9	44.9282
888.369	46.2009
888.836	40.1313
889.306	38.9971
889.773	38.9824
890.242	42.2579
890.709	43.8519
891.178	43.003
891.645	44.4834
892.114	47.4955
892.581	44.1946
893.049	41.4986
893.516	40.7818
893.985	45.9401
894.452	43.6892
894.921	42.4994

895.388	33.6345
895.856	41.3655
896.323	44.7928
896.792	37.3925
897.259	39.9975
897.725	40.8171
898.194	35.1234
898.661	45.3852
899.129	39.9227
899.596	42.8993
900.064	44.1102
900.531	41.8629
900.997	38.485
901.466	39.4283
901.932	44.4816
902.401	38.8719
902.867	40.1581
903.333	40.7664
903.802	45.1192
904.268	39.7908
904.734	41.3829
905.203	43.6571
905.669	42.752
906.135	40.2313
906.603	42.3668
907.07	46.9853
907.536	41.7433

908.004	41.0681
908.47	45.0641
908.936	46.134
909.404	42.5946
909.87	49.2363
910.336	37.7489
910.804	45.6222
911.27	40.1854
911.736	45.1008
912.202	39.766
912.67	41.4966
913.136	42.5958
913.602	38.5189
914.067	41.9565
914.535	38.7741
915.001	40.7719
915.467	35.2108
915.932	45.1756
916.4	37.1155
916.866	35.3478
917.331	40.4617
917.797	40.7503
918.263	43.3656
918.73	47.1409
919.196	38.5635
919.661	38.8519
920.127	44.068

920.592	40.5601
921.058	43.072
921.525	41.4006
921.991	42.5467
922.456	38.907
922.921	43.2076
923.387	42.3706
923.852	39.6166
924.317	43.1964
924.783	39.8601
925.25	37.2528
925.715	44.7772
926.18	45.0961
926.646	37.8092
927.111	34.1607
927.576	40.0391
928.041	44.6072
928.506	41.9073
928.971	41.852
929.436	40.7776
929.901	43.3523
930.366	40.6144
930.831	42.4197
931.296	39.7849
931.761	42.5068
932.226	41.2532
932.691	37.8176

933.155	42.0626
933.62	45.9233
934.085	38.6527
934.55	43.7385
935.015	41.5641
935.479	42.6505
935.944	43.8911
936.409	45.4606
936.873	46.5934
937.338	49.444
937.802	36.6244
938.267	39.6842
938.732	45.6847
939.196	39.8995
939.661	40.7636
940.125	38.8788
940.59	43.0936
941.054	37.25
941.518	46.1954
941.981	47.7768
942.445	45.7708
942.91	42.1201
943.374	42.6703
943.838	39.871
944.302	40.0338
944.767	41.1609
945.231	34.311

945.693	42.2707
946.157	40.9163
946.621	44.2978
947.086	39.9419
947.55	40.4227
948.014	45.5526
948.476	43.5824
948.94	37.8127
949.404	39.7887
949.868	41.2331
950.332	46.0428
950.794	43.5143
951.258	40.5799
951.721	42.6197
952.185	45.6994
952.649	44.6023
953.111	42.2442
953.575	45.0929
954.039	42.1696
954.502	46.9554
954.964	43.3598
955.428	42.5986
955.891	40.3284
956.353	38.5298
956.817	48.2596
957.28	43.9019
957.744	47.2774

958.205	38.1649
958.669	42.1922
959.132	41.6876
959.594	40.1104
960.057	41.0592
960.521	40.6851
960.982	41.6198
961.446	44.0749
961.909	42.0756
962.37	41.4602
962.834	44.6079
963.297	40.3153
963.758	40.014
964.221	41.9803
964.683	41.2653
965.146	42.8092
965.609	39.5921
966.07	49.2854
966.533	46.4188
966.994	47.5896
967.457	40.6198
967.921	49.0139
968.382	39.3986
968.845	45.3404
969.306	49.2855
969.768	52.315
970.229	45.9505

970.692	48.1395
971.153	44.8718
971.616	42.6886
972.077	43.2114
972.54	42.2852
973.001	45.0172
973.463	48.1064
973.924	46.2962
974.387	43.6555
974.848	48.0515
975.31	43.2427
975.771	43.7462
976.234	45.6502
976.694	47.3475
977.157	46.0339
977.617	46.2365
978.08	45.2608
978.54	46.6677
979.003	47.5593
979.463	46.6329
979.924	46.5679
980.386	48.1458
980.847	45.3672
981.309	47.9767
981.769	44.3341
982.23	53.5115
982.692	50.649

983.152	43.656
983.614	47.7112
984.075	48.867
984.535	50.7222
984.997	51.0724
985.457	44.7982
985.917	45.3241
986.38	51.2777
986.84	49.1595
987.3	48.3397
987.762	47.6693
988.222	49.7882
988.682	49.8167
989.144	49.7711
989.604	44.8317
990.064	48.3977
990.524	48.6614
990.985	44.5601
991.445	47.5511
991.905	43.9075
992.367	48.4184
992.827	46.6482
993.286	44.5632
993.746	49.0839
994.208	45.7493
994.668	51.0795
995.127	49.8448

995.587	48.075
996.047	49.8706
996.508	47.964
996.968	47.3505
997.427	46.2964
997.887	49.5505
998.346	49.2249
998.808	47.8125
999.267	47.6513
999.727	47.9451
1000.19	49.5577
1000.65	47.8598
1001.1	50.3024
1001.57	48.5382
1002.03	44.694
1002.48	43.4751
1002.94	45.4459
1003.4	47.6114
1003.86	43.8467
1004.32	41.881
1004.78	45.4091
1005.24	45.4584
1005.7	47.7396
1006.16	45.4265
1006.62	45.2543
1007.08	47.1765
1007.54	46.425

1008	45.169
1008.46	44.6411
1008.91	45.8908
1009.37	49.3241
1009.83	50.6858
1010.29	49.3836
1010.75	51.1026
1011.21	51.2019
1011.67	51.5355
1012.13	54.748
1012.59	57.3627
1013.04	54.2075
1013.5	50.9093
1013.96	48.5816
1014.42	46.0495
1014.88	49.5133
1015.34	50.2832
1015.8	45.1831
1016.25	51.027
1016.71	45.5329
1017.17	42.5091
1017.63	45.338
1018.09	46.628
1018.55	46.0215
1019.01	40.1142
1019.46	46.454
1019.92	48.6097

1020.38	45.2778
1020.84	46.6736
1021.3	46.0195
1021.75	44.273
1022.21	40.6807
1022.67	41.8483
1023.13	41.1011
1023.59	43.938
1024.04	47.6063
1024.5	43.9485
1024.96	45.01
1025.42	47.101
1025.88	48.3639
1026.33	45.218
1026.79	44.9026
1027.25	45.4032
1027.71	43.1007
1028.16	43.1013
1028.62	44.102
1029.08	40.8339
1029.54	41.2647
1029.99	40.9866
1030.45	47.7757
1030.91	42.305
1031.37	42.3708
1031.82	39.4499
1032.28	46.8685

1032.74	45.3203
1033.19	46.5293
1033.65	47.2591
1034.11	43.6614
1034.57	44.7744
1035.02	42.5439
1035.48	44.1564
1035.94	46.9829
1036.39	44.6232
1036.85	48.4178
1037.31	46.295
1037.76	48.7371
1038.22	47.6248
1038.68	44.3718
1039.14	43.7787
1039.59	41.7585
1040.05	42.4943
1040.51	49.2577
1040.96	41.9923
1041.42	38.1469
1041.88	41.1775
1042.33	43.6397
1042.79	46.5098
1043.25	40.4993
1043.7	40.0569
1044.16	48.4758
1044.61	46.1445

1045.07	46.0712
1045.53	49.9349
1045.98	41.7599
1046.44	42.0085
1046.9	44.3842
1047.35	41.063
1047.81	44.3515
1048.26	43.9711
1048.72	39.646
1049.18	39.9599
1049.63	41.3822
1050.09	39.0891
1050.54	42.6124
1051	41.2519
1051.46	38.277
1051.91	43.3531
1052.37	44.8855
1052.82	43.5367
1053.28	41.8865
1053.73	46.3594
1054.19	43.0096
1054.64	45.7259
1055.1	39.7058
1055.56	39.561
1056.01	43.6316
1056.47	43.6514
1056.92	43.3934

1057.38	43.0791
1057.83	41.0082
1058.29	42.6004
1058.74	41.022
1059.2	42.6616
1059.65	43.7709
1060.11	45.3606
1060.57	45.7542
1061.02	43.2671
1061.48	48.3596
1061.93	46.2824
1062.38	46.3696
1062.84	42.6401
1063.29	47.0926
1063.75	43.2863
1064.21	43.8713
1064.66	43.5862
1065.11	39.3095
1065.57	44.98
1066.02	45.5928
1066.48	40.2249
1066.93	43.8622
1067.39	40.5753
1067.84	42.6429
1068.3	43.6834
1068.75	44.1616
1069.21	43.9525

1069.66	41.849
1070.11	42.0958
1070.57	38.8822
1071.02	38.7211
1071.48	42.0472
1071.93	46.6873
1072.39	48.0481
1072.84	42.7567
1073.29	41.0652
1073.75	41.0659
1074.2	44.6282
1074.66	45.1497
1075.11	47.8151
1075.56	49.6246
1076.02	45.2572
1076.47	41.843
1076.93	42.9655
1077.38	46.3228
1077.83	45.951
1078.29	48.6405
1078.74	48.2039
1079.19	44.8589
1079.65	44.7146
1080.1	42.5332
1080.56	41.2091
1081.01	43.3772
1081.46	44.3771

1081.92	42.7011
1082.37	42.3033
1082.82	47.2806
1083.28	46.6977
1083.73	38.0692
1084.18	41.0145
1084.64	45.9169
1085.09	43.4963
1085.54	44.9707
1086	45.1097
1086.45	49.2466
1086.9	42.5216
1087.36	36.8841
1087.81	46.2577
1088.26	48.8269
1088.71	44.8154
1089.17	44.5768
1089.62	41.5188
1090.07	39.4025
1090.53	45.5127
1090.98	48.3081
1091.43	44.4055
1091.88	45.3868
1092.34	42.8221
1092.79	47.9621
1093.24	50.2902
1093.69	47.8594

1094.15	44.3888
1094.6	45.4071
1095.05	49.3359
1095.5	41.9543
1095.96	50.3392
1096.41	48.8747
1096.86	48.1332
1097.31	45.4805
1097.77	44.7971
1098.22	39.2326
1098.67	46.1893
1099.12	43.7389
1099.57	47.2197
1100.03	47.2382
1100.48	40.0912
1100.86	44.9716
1101.31	42.9088
1101.77	40.9692
1102.22	46.8126
1102.68	54.5399
1103.13	42.885
1103.59	44.7796
1104.04	50.6962
1104.5	50.6941
1104.95	47.0135
1105.41	56.5638
1105.86	45.039

1106.32	45.0758
1106.77	46.82
1107.23	48.6048
1107.68	50.4788
1108.14	52.4027
1108.59	50.3781
1109.05	50.2958
1109.5	41.2685
1109.95	46.9629
1110.41	55.9011
1110.86	40.8115
1111.32	50.3075
1111.77	52.1639
1112.23	54.1037
1112.68	42.9779
1113.13	46.4425
1113.59	46.764
1114.04	43.0357
1114.5	48.2719
1114.95	39.1539
1115.41	44.9079
1115.86	48.5788
1116.31	50.2779
1116.77	48.3752
1117.22	42.8166
1117.67	48.2293
1118.13	48.6587

1118.58	41.6957
1119.04	42.6677
1119.49	37.4973
1119.94	42.9275
1120.4	44.9396
1120.85	41.584
1121.3	44.325
1121.76	46.915
1122.21	45.4814
1122.66	44.4508
1123.12	46.0356
1123.57	49.6053
1124.03	38.9491
1124.48	40.1344
1124.93	45.7881
1125.38	42.5463
1125.84	45.4772
1126.29	48.3154
1126.74	37.6046
1127.2	37.8862
1127.65	50.223
1128.1	43.5556
1128.56	43.1598
1129.01	42.0271
1129.46	39.1528
1129.91	45.5612
1130.37	39.2441

1130.82	44.9793
1131.27	45.4383
1131.73	45.5393
1132.18	42.7276
1132.63	47.8482
1133.09	45.4164
1133.54	43.9975
1133.99	40.705
1134.44	46.0255
1134.9	47.8121
1135.35	40.489
1135.8	46.5873
1136.25	44.584
1136.7	47.4247
1137.16	46.5899
1137.61	54.0319
1138.06	39.057
1138.51	43.0169
1138.97	43.389
1139.42	46.8134
1139.87	41.8917
1140.32	44.1765
1140.78	45.3384
1141.23	44.2679
1141.68	33.8668
1142.13	43.3189
1142.58	40.4786

1143.04	41.8081
1143.49	41.3214
1143.94	47.2733
1144.39	48.1271
1144.84	46.1104
1145.29	40.122
1145.75	44.1106
1146.2	46.9703
1146.65	44.5463
1147.1	45.2135
1147.55	40.9661
1148	40.5385
1148.46	40.0809
1148.91	44.4523
1149.36	40.118
1149.81	44.6385
1150.26	40.2706
1150.71	44.5337
1151.16	48.4239
1151.62	45.0132
1152.07	44.5309
1152.52	41.813
1152.97	47.1944
1153.42	44.4768
1153.87	47.9144
1154.32	46.1774
1154.77	46.8129

1155.22	47.3662
1155.68	49.9428
1156.13	48.6787
1156.58	42.8885
1157.03	46.9182
1157.48	44.5737
1157.93	43.8151
1158.38	42.4593
1158.83	44.4771
1159.28	45.5383
1159.73	41.422
1160.18	47.7534
1160.63	43.5363
1161.08	46.4135
1161.54	45.421
1161.99	43.6776
1162.44	44.9776
1162.89	41.6923
1163.34	50.1581
1163.79	45.8053
1164.24	46.4788
1164.69	48.1958
1165.14	52.6589
1165.59	52.1991
1166.04	44.2877
1166.49	48.9802
1166.94	42.6611

1167.39	44.943
1167.84	45.0333
1168.29	46.0355
1168.74	51.8934
1169.19	39.2022
1169.64	47.7151
1170.09	42.0577
1170.54	40.7815
1170.99	42.111
1171.44	45.9568
1171.89	41.2555
1172.34	46.6838
1172.79	47.7548
1173.24	46.9827
1173.69	42.0547
1174.14	43.6375
1174.59	47.4912
1175.03	43.1457
1175.48	45.8408
1175.93	45.9364
1176.38	47.1212
1176.83	42.8966
1177.28	43.2792
1177.73	47.853
1178.18	48.5432
1178.63	40.2322
1179.08	50.3276

1179.53	44.8769
1179.98	45.3702
1180.43	41.3923
1180.88	44.6226
1181.32	49.8353
1181.77	44.7737
1182.22	41.8963
1182.67	46.8521
1183.12	47.2688
1183.57	41.132
1184.02	43.0215
1184.47	43.0024
1184.92	43.8891
1185.37	43.959
1185.81	46.8523
1186.26	49.6807
1186.71	45.5598
1187.16	43.8883
1187.61	41.8902
1188.06	44.1576
1188.5	48.6222
1188.95	51.199
1189.4	45.686
1189.85	44.7089
1190.3	46.1611
1190.75	44.1451
1191.2	40.2829

1191.64	47.8831
1192.09	47.3106
1192.54	45.3874
1192.99	46.4225
1193.44	43.8329
1193.88	42.7277
1194.33	45.0219
1194.78	46.0923
1195.23	46.6173
1195.68	46.6442
1196.12	45.5339
1196.57	42.1116
1197.02	42.9031
1197.47	44.6375
1197.92	44.079
1198.36	44.3833
1198.81	44.5991
1199.26	43.4732
1199.71	46.5989
1200.15	46.77
1200.6	51.8444
1201.05	47.1504
1201.5	45.9318
1201.94	43.0405
1202.39	45.0182
1202.84	43.0156
1203.29	43.5222

1203.73	46.5729
1204.18	43.7783
1204.63	41.6568
1205.07	45.0491
1205.52	48.3678
1205.97	45.2659
1206.42	41.0371
1206.86	50.5988
1207.31	42.0959
1207.76	42.2149
1208.2	46.5084
1208.65	46.7815
1209.1	44.8488
1209.55	49.8213
1209.99	49.6484
1210.44	47.092
1210.89	44.9083
1211.33	43.0794
1211.78	44.2763
1212.23	43.8878
1212.67	44.1642
1213.12	46.961
1213.57	42.104
1214.01	47.0415
1214.46	48.0444
1214.9	48.9434
1215.35	42.9828

1215.8	44.9257
1216.24	42.8177
1216.69	46.0998
1217.14	38.0071
1217.58	45.9529
1218.03	43.1123
1218.48	47.813
1218.92	41.0454
1219.37	44.025
1219.82	46.1146
1220.26	42.2859
1220.71	43.9381
1221.15	44.0359
1221.6	41.9991
1222.04	44.6715
1222.49	46.1752
1222.94	47.3105
1223.38	42.4285
1223.83	46.8801
1224.27	44.0842
1224.72	45.7912
1225.17	47.1612
1225.61	47.5966
1226.06	43.0517
1226.5	45.7413
1226.95	47.399
1227.39	43.4753

1227.84	46.1228
1228.28	41.9889
1228.73	42.8427
1229.18	46.4141
1229.62	44.2867
1230.07	46.6488
1230.51	45.5849
1230.96	42.4627
1231.4	49.8894
1231.85	40.3602
1232.29	44.06
1232.74	41.7028
1233.18	46.1322
1233.63	39.7402
1234.07	44.3142
1234.52	43.0533
1234.96	42.4561
1235.41	44.6449
1235.85	48.3387
1236.3	46.7446
1236.74	40.3066
1237.19	45.1477
1237.63	47.1996
1238.08	46.3314
1238.52	40.2465
1238.97	40.7928
1239.41	44.7051

1239.85	41.5506
1240.3	45.4467
1240.75	40.8908
1241.19	40.1274
1241.63	41.4051
1242.08	44.2498
1242.52	41.0073
1242.97	43.749
1243.41	40.4201
1243.86	45.7139
1244.3	46.1916
1244.74	43.2721
1245.19	39.2281
1245.63	40.6277
1246.08	40.6506
1246.52	41.1551
1246.96	40.7776
1247.41	41.7801
1247.85	42.0202
1248.3	44.5783
1248.74	41.8979
1249.18	41.2538
1249.63	39.2541
1250.07	42.6184
1250.52	43.2347
1250.96	41.2257
1251.4	45.8432

1251.85	42.6178
1252.29	40.7273
1252.73	39.8261
1253.18	43.0959
1253.62	40.6101
1254.06	43.2097
1254.51	42.831
1254.95	47.2901
1255.4	46.5103
1255.84	45.3046
1256.28	45.0693
1256.73	44.6544
1257.17	42.3315
1257.61	41.8786
1258.05	43.0812
1258.5	40.7397
1258.94	41.4695
1259.38	42.7462
1259.83	37.9081
1260.27	40.6561
1260.71	46.3649
1261.16	40.7184
1261.6	42.2307
1262.04	40.215
1262.49	44.4283
1262.93	41.277
1263.37	40.6316

1263.81	36.0716
1264.26	42.2232
1264.7	39.4242
1265.14	43.3815
1265.58	43.4553
1266.03	43.7224
1266.47	45.9939
1266.91	44.4412
1267.35	38.7276
1267.8	43.9873
1268.24	47.7515
1268.68	39.8871
1269.13	41.8194
1269.57	41.1639
1270.01	46.3936
1270.45	42.6198
1270.89	41.0618
1271.34	40.8161
1271.78	41.7728
1272.22	40.3199
1272.66	42.7277
1273.11	44.3103
1273.55	42.9148
1273.99	45.5172
1274.43	39.8559
1274.87	38.0738
1275.32	41.7466

1275.76	46.8337
1276.2	41.5716
1276.64	42.9512
1277.08	42.9965
1277.52	47.9263
1277.96	41.262
1278.41	40.9433
1278.85	42.5644
1279.29	43.714
1279.73	43.3801
1280.17	40.8184
1280.61	44.0825
1281.06	40.8292
1281.5	45.8232
1281.94	46.7613
1282.38	36.2452
1282.82	38.6212
1283.26	41.8157
1283.7	48.3896
1284.15	45.4645
1284.59	44.9523
1285.03	43.6818
1285.47	46.9663
1285.91	48.176
1286.35	48.3305
1286.79	45.9675
1287.23	45.4665

1287.67	42.6972
1288.11	47.819
1288.56	45.7341
1289	43.4795
1289.44	49.7175
1289.88	41.7805
1290.32	43.9689
1290.76	38.7383
1291.2	37.3769
1291.64	43.3199
1292.08	47.3815
1292.52	46.0303
1292.96	42.006
1293.4	43.3178
1293.85	40.324
1294.29	43.8444
1294.72	45.5173
1295.17	43.8244
1295.61	44.0801
1296.05	45.7549
1296.49	46.1329
1296.93	49.5345
1297.37	46.2754
1297.81	41.0508
1298.25	40.0549
1298.69	49.7681
1299.13	44.7695

1299.57	40.9464
1300.01	42.0742
1300.45	48.6336
1300.89	48.2663
1301.33	47.8009
1301.77	47.5795
1302.21	45.8907
1302.65	45.5011
1303.09	42.4278
1303.53	43.3269
1303.97	46.49
1304.41	46.5653
1304.85	48.6411
1305.29	49.7991
1305.72	47.3963
1306.16	45.6161
1306.6	45.2621
1307.04	46.4986
1307.48	42.6122
1307.92	47.9083
1308.36	44.7536
1308.8	46.1053
1309.24	48.5188
1309.68	47.4942
1310.12	40.6962
1310.56	42.1215
1311	47.9636

1311.44	47.507
1311.88	46.7244
1312.32	43.5977
1312.76	43.6809
1313.19	49.1321
1313.63	48.6194
1314.07	47.036
1314.51	49.7923
1314.95	47.181
1315.39	42.3079
1315.83	47.9687
1316.27	46.629
1316.7	43.8248
1317.14	47.7122
1317.58	48.1297
1318.02	44.8389
1318.46	43.8274
1318.9	48.3784
1319.34	46.4296
1319.77	39.9267
1320.21	42.5929
1320.65	44.8437
1321.09	43.9043
1321.53	44.765
1321.97	45.8009
1322.41	45.7608
1322.84	44.2781

1323.28	44.2837
1323.72	44.8018
1324.16	42.8965
1324.6	47.7725
1325.04	47.1092
1325.47	44.4915
1325.91	43.9641
1326.35	46.4066
1326.79	47.2571
1327.08	53.572
1327.52	39.909
1327.96	47.799
1328.4	45.9165
1328.84	47.7544
1329.29	43.7871
1329.73	45.8025
1330.17	37.943
1330.61	43.7694
1331.05	47.601
1331.49	45.6588
1331.93	40.084
1332.37	47.7934
1332.81	47.756
1333.25	40.0169
1333.69	45.2989
1334.13	43.6121
1334.57	47.6172

1335.01	47.7068
1335.45	47.7822
1335.89	43.7659
1336.33	41.9286
1336.77	41.7529
1337.21	43.6818
1337.65	43.909
1338.09	46.1475
1338.54	47.6713
1338.98	47.2809
1339.42	45.4699
1339.86	47.3958
1340.29	49.5357
1340.73	45.6123
1341.17	45.0366
1341.61	45.3198
1342.05	49.2695
1342.49	40.0031
1342.93	49.4906
1343.37	39.9802
1343.81	41.373
1344.25	41.6385
1344.69	49.0708
1345.13	42.0224
1345.57	49.3599
1346.01	43.7649
1346.45	40.1496

1346.89	42.0666
1347.33	47.2422
1347.77	40.115
1348.21	45.5915
1348.65	44.9011
1349.09	48.3373
1349.53	41.9423
1349.97	45.6136
1350.41	45.8729
1350.85	42.5894
1351.29	47.2633
1351.72	45.0842
1352.16	43.1592
1352.6	40.1299
1353.04	41.4071
1353.48	41.5017
1353.92	45.0088
1354.36	45.664
1354.8	45.8601
1355.24	37.1345
1355.67	47.1508
1356.11	45.7801
1356.55	47.1833
1356.99	43.3229
1357.43	41.2573
1357.87	45.3965
1358.31	48.5203

1358.75	43.884
1359.19	44.7998
1359.62	47.214
1360.06	43.8671
1360.5	48.7227
1360.94	40.2147
1361.38	46.2826
1361.82	36.8397
1362.25	43.3654
1362.69	42.5777
1363.13	50.6114
1363.57	43.2113
1364.01	43.8074
1364.44	40.2832
1364.88	45.7065
1365.32	40.0369
1365.76	52.2529
1366.2	43.443
1366.64	35.042
1367.07	41.533
1367.51	49.9008
1367.95	41.7965
1368.39	42.8743
1368.83	44.6591
1369.26	48.988
1369.7	43.3694
1370.14	40.723

1370.58	41.8003
1371.02	40.741
1371.45	41.1319
1371.89	49.6946
1372.33	43.0128
1372.77	44.5806
1373.2	40.3239
1373.64	44.5767
1374.08	48.9781
1374.52	51.6064
1374.95	39.2772
1375.39	46.5003
1375.83	45.2886
1376.26	46.5109
1376.7	51.5049
1377.14	34.7843
1377.58	41.4492
1378.01	43.8612
1378.45	47.3883
1378.89	41.8618
1379.32	42
1379.76	46.865
1380.2	47.9152
1380.64	47.3156
1381.07	38.5559
1381.51	47.7191
1381.95	48.4439

1382.38	42.9701
1382.82	39.3868
1383.26	42.9721
1383.69	43.7812
1384.13	45.3757
1384.57	41.9931
1385	40.9774
1385.44	43.7056
1385.88	47.518
1386.31	40.9849
1386.75	34.5589
1387.19	41.9093
1387.62	47.4757
1388.06	45.134
1388.5	42.5119
1388.93	44.8802
1389.37	44.7658
1389.8	39.6911
1390.24	45.159
1390.68	46.9355
1391.11	42.2683
1391.55	45.8234
1391.99	47.9566
1392.42	41.1747
1392.86	39.8035
1393.29	44.4036
1393.73	41.8295

1394.17	44.5802
1394.6	47.5911
1395.04	40.5097
1395.47	46.2838
1395.91	46.2128
1396.35	42.4063
1396.78	44.8549
1397.22	43.7518
1397.65	39.2985
1398.09	45.5163
1398.52	41.0855
1398.96	49.8928
1399.4	40.2786
1399.83	45.4759
1400.27	45.9834
1400.7	45.6299
1401.14	42.7681
1401.57	44.1546
1402.01	46.0213
1402.44	50.4207
1402.88	47.8721
1403.32	38.2747
1403.75	46.4871
1404.19	42.0724
1404.62	37.5316
1405.06	45.7578
1405.49	42.5616

1405.93	41.256
1406.36	43.4327
1406.8	41.5185
1407.23	44.5025
1407.67	42.3897
1408.1	42.7551
1408.54	42.8449
1408.97	44.1911
1409.41	44.1736
1409.84	41.1222
1410.27	46.8871
1410.71	44.826
1411.14	47.2994
1411.58	48.4493
1412.01	47.6479
1412.45	40.6716
1412.88	40.6495
1413.32	39.6707
1413.75	43.8493
1414.19	44.8725
1414.62	41.6865
1415.06	46.9599
1415.49	44.3772
1415.92	42.2392
1416.36	45.8366
1416.79	46.7434
1417.23	42.3438

1417.66	44.474
1418.1	43.8225
1418.53	40.3532
1418.96	48.6468
1419.4	47.1997
1419.83	51.4638
1420.27	47.4651
1420.7	42.9228
1421.13	39.8324
1421.57	47.0409
1422	44.1401
1422.43	40.593
1422.87	46.5549
1423.3	43.3378
1423.74	47.6016
1424.17	42.93
1424.6	44.0894
1425.04	44.272
1425.47	43.193
1425.91	43.0024
1426.34	38.6015
1426.77	45.1068
1427.21	43.968
1427.64	44.8364
1428.07	47.0251
1428.51	46.6441
1428.94	44.478

1429.37	46.3603
1429.81	49.6455
1430.24	47.2284
1430.67	44.9126
1431.1	44.8357
1431.54	44.2654
1431.97	45.5093
1432.4	40.1736
1432.84	45.7115
1433.27	41.9457
1433.7	43.3972
1434.14	44.5081
1434.57	44.6727
1435	43.45
1435.44	46.7223
1435.87	41.0395
1436.3	44.4705
1436.73	48.7892
1437.17	43.1825
1437.6	45.3672
1438.03	50.0824
1438.46	47.1184
1438.9	48.2504
1439.33	46.0638
1439.76	44.0891
1440.2	44.0896
1440.63	45.0062

1441.06	47.081
1441.49	44.0718
1441.92	46.0667
1442.36	46.292
1442.79	42.0834
1443.22	42.2856
1443.65	48.9433
1444.09	45.377
1444.52	42.191
1444.95	43.3
1445.38	44.4832
1445.82	45.3439
1446.25	44.2653
1446.68	44.076
1447.11	47.8293
1447.54	44.4165
1447.97	46.2068
1448.41	45.8363
1448.84	42.9813
1449.27	42.215
1449.7	43.9668
1450.13	50.7794
1450.57	42.961
1451	44.9026
1451.43	43.5287
1451.86	42.2802
1452.29	49.8353

1452.72	45.4455
1453.16	44.8256
1453.59	42.5635
1454.02	47.8234
1454.45	47.3025
1454.88	42.5903
1455.31	45.4269
1455.75	42.5012
1456.18	45.5267
1456.61	44.9194
1457.04	41.8892
1457.47	48.0522
1457.9	52.8917
1458.33	41.538
1458.76	46.9484
1459.19	48.858
1459.62	49.5988
1460.06	47.187
1460.49	42.6354
1460.92	43.8412
1461.35	46.3398
1461.78	45.4365
1462.21	46.7772
1462.64	45.0009
1463.07	43.9999
1463.5	48.7381
1463.93	47.1602

1464.36	47.1429
1464.8	46.2726
1465.23	46.9327
1465.66	41.7075
1466.09	44.9848
1466.52	43.8369
1466.95	46.5703
1467.38	40.402
1467.81	41.7673
1468.24	44.7165
1468.67	47.2952
1469.1	45.3043
1469.53	41.7493
1469.96	45.4157
1470.39	46.934
1470.82	47.1443
1471.25	44.7914
1471.68	44.3717
1472.11	40.8864
1472.54	43.8492
1472.97	47.7756
1473.4	47.0018
1473.83	43.7033
1474.26	43.7217
1474.69	45.0143
1475.12	40.3729
1475.55	44.1786

1475.98	45.693
1476.41	41.3069
1476.84	41.6824
1477.27	40.6546
1477.7	41.6604
1478.13	41.2154
1478.56	43.5692
1478.99	46.8009
1479.42	45.6487
1479.85	40.9337
1480.28	43.2248
1480.71	45.21
1481.14	45.2955
1481.57	46.7655
1482	46.1176
1482.42	45.6963
1482.85	42.5704
1483.28	39.6703
1483.71	39.7596
1484.14	42.1008
1484.57	43.0365
1485	45.1927
1485.43	44.9578
1485.86	44.835
1486.29	45.9429
1486.72	43.5129
1487.15	40.3364

1487.57	34.7412
1488	44.7127
1488.43	46.5249
1488.86	45.0643
1489.29	42.9404
1489.72	39.7315
1490.15	42.5223
1490.58	41.5106
1491.01	42.7486
1491.43	46.0335
1491.86	45.3656
1492.29	41.5486
1492.72	38.9708
1493.15	43.0623
1493.58	40.3443
1494	40.3596
1494.43	41.9789
1494.86	43.8025
1495.29	48.5751
1495.72	42.3173
1496.15	42.9112
1496.57	48.1206
1497	43.2368
1497.43	40.6002
1497.86	38.1546
1498.29	37.9886
1498.72	44.081

1499.14	42.6195
1499.57	39.143
1500	44.9011
1500.43	42.0088
1500.86	39.3764
1501.28	41.9773
1501.71	43.6325
1502.14	39.4381
1502.57	43.6609
1502.99	42.489
1503.42	40.4815
1503.85	45.4279
1504.28	44.5274
1504.71	39.1037
1505.13	41.8511
1505.56	40.1601
1505.99	41.4044
1506.42	49.177
1506.84	46.3695
1507.27	44.7141
1507.7	40.4139
1508.13	42.2822
1508.55	41.7982
1508.98	48.0169
1509.41	45.2211
1509.84	49.0719
1510.26	38.1612

1510.69	38.344
1511.12	48.4321
1511.54	49.447
1511.97	47.3543
1512.4	43.0732
1512.83	43.3478
1513.25	43.6474
1513.68	40.3573
1514.11	41.7043
1514.53	42.5194
1514.96	44.5086
1515.39	45.0434
1515.81	39.8748
1516.24	41.0356
1516.67	43.6279
1517.09	47.8924
1517.52	41.7217
1517.95	39.8283
1518.37	46.1718
1518.8	43.7699
1519.23	44.4206
1519.65	46.662
1520.08	44.3366
1520.51	39.8713
1520.93	45.5774
1521.36	49.4766
1521.79	44.6299

1522.21	45.674
1522.64	40.9555
1523.06	40.5616
1523.49	41.9224
1523.92	44.1249
1524.34	44.6501
1524.77	45.4538
1525.2	43.4202
1525.62	41.4413
1526.05	43.5162
1526.47	47.4914
1526.9	45.9194
1527.33	43.511
1527.75	45.0881
1528.18	42.6824
1528.6	46.3867
1529.03	45.2818
1529.46	39.932
1529.88	41.9106
1530.31	45.2146
1530.73	47.2836
1531.16	42.7079
1531.58	45.3376
1532.01	46.7846
1532.44	43.8708
1532.86	44.504
1533.29	41.944

1533.71	43.0881
1534.14	46.7321
1534.56	45.7276
1534.99	45.8664
1535.41	46.4627
1535.84	41.8672
1536.26	42.112
1536.69	45.8527
1537.11	42.3343
1537.54	43.6691
1537.96	47.5433
1538.39	44.8576
1538.81	46.8823
1539.24	47.8634
1539.66	45.4035
1540.09	47.3849
1540.52	51.2074
1540.94	46.989
1541.36	43.3403
1541.79	42.9006
1542.21	46.7849
1542.64	48.9842
1543.06	46.6384
1543.49	45.4016
1543.91	48.1907
1544.34	44.8388
1544.76	40.3864

1545.19	41.2994
1545.61	45.7983
1546.04	45.6993
1546.46	45.8336
1546.74	51.1109
1547.17	49.1501
1547.6	37.1977
1548.03	39.1396
1548.45	47.0134
1548.88	46.8757
1549.31	50.9373
1549.73	48.9474
1550.16	47.0885
1550.59	43.0621
1551.02	47.0024
1551.44	43.1621
1551.87	47.0018
1552.3	49.1094
1552.72	47.1751
1553.15	47.1169
1553.58	49.0977
1554	52.7381
1554.43	44.5985
1554.86	39.0028
1555.29	50.2035
1555.71	45.2115
1556.14	45.2027

1556.57	46.8082
1556.99	43.4362
1557.42	43.1079
1557.85	44.766
1558.27	50.8708
1558.7	47.239
1559.13	48.8543
1559.55	46.7138
1559.98	45.5213
1560.41	41.2676
1560.83	45.6815
1561.26	42.9934
1561.68	41.4127
1562.11	44.8166
1562.54	45.5577
1562.96	49.2832
1563.39	49.2874
1563.82	48.488
1564.24	46.4248
1564.67	48.8771
1565.09	45.1365
1565.52	41.3267
1565.95	32.7925
1566.37	46.2671
1566.8	43.2292
1567.23	40.0939
1567.65	40.8629

1568.08	45.0068
1568.5	45.8652
1568.93	42.9095
1569.35	42.2952
1569.78	45.3222
1570.2	48.9303
1570.63	37.716
1571.06	44.8405
1571.48	46.241
1571.91	44.3825
1572.33	42.2218
1572.76	46.8132
1573.18	50.4028
1573.61	48.8045
1574.04	46.0123
1574.46	52.5022
1574.89	46.7088
1575.31	43.7561
1575.74	44.6043
1576.16	43.5697
1576.59	46.7607
1577.01	47.0473
1577.44	45.6393
1577.86	43.8154
1578.29	47.5603
1578.71	42.239
1579.14	46.2501

1579.56	48.2006
1579.99	45.1281
1580.41	38.4279
1580.84	41.7861
1581.26	47.0178
1581.69	43.5805
1582.11	43.2483
1582.54	41.0272
1582.96	42.6564
1583.39	45.094
1583.81	45.0412
1584.24	43.798
1584.66	41.4649
1585.09	42.1032
1585.51	48.389
1585.94	46.0043
1586.36	51.5222
1586.79	46.6848
1587.21	46.1338
1587.63	48.4582
1588.06	43.8731
1588.48	45.3531
1588.91	48.2874
1589.33	44.7254
1589.76	47.1948
1590.18	40.9578
1590.6	41.7502

1591.03	38.3992
1591.45	43.7854
1591.88	37.2791
1592.3	41.7783
1592.72	40.2414
1593.15	42.6917
1593.57	46.854
1594	44.2941
1594.42	47.5932
1594.85	44.1827
1595.27	46.0036
1595.69	44.8247
1596.12	45.8996
1596.54	43.9964
1596.96	40.8378
1597.39	45.851
1597.81	44.3915
1598.23	47.8208
1598.66	44.3194
1599.08	47.6465
1599.51	48.2133
1599.93	41.2718
1600.35	43.4536
1600.78	38.8581
1601.2	44.0222
1601.62	47.4707
1602.05	47.7029

1602.47	45.0399
1602.89	48.4312
1603.32	42.1765
1603.74	46.2497
1604.16	42.932
1604.58	40.2756
1605.01	43.979
1605.43	43.3662
1605.85	45.3007
1606.28	42.955
1606.7	47.9579
1607.12	43.9719
1607.55	42.0117
1607.97	42.5723
1608.39	47.5341
1608.82	49.5204
1609.24	44.0406
1609.66	44.8134
1610.08	45.4705
1610.51	45.9341
1610.93	41.7416
1611.35	44.5276
1611.77	45.9109
1612.2	44.8711
1612.62	47.6441
1613.04	42.4568
1613.46	44.5138

1613.89	43.8921
1614.31	41.6253
1614.73	45.1366
1615.16	45.0438
1615.58	45.7534
1616	43.8331
1616.42	38.6896
1616.85	45.6403
1617.27	44.4029
1617.69	42.605
1618.11	45.2674
1618.53	43.8369
1618.95	46.3851
1619.38	46.5057
1619.8	44.6085
1620.22	49.7041
1620.64	47.5942
1621.07	42.3781
1621.49	42.8811
1621.91	37.1144
1622.33	43.1823
1622.75	40.4827
1623.17	48.4426
1623.6	49.803
1624.02	41.6847
1624.44	47.8501
1624.86	47.518

1625.28	42.2989
1625.7	45.0607
1626.13	44.9445
1626.55	41.6662
1626.97	45.0399
1627.39	35.8528
1627.81	42.4054
1628.23	41.9483
1628.65	44.8852
1629.08	48.8995
1629.5	41.201
1629.92	47.8061
1630.34	44.5502
1630.76	44.8078
1631.18	36.6282
1631.6	45.5527
1632.03	43.7357
1632.45	47.3455
1632.87	38.5829
1633.29	42.6399
1633.71	48.198
1634.13	51.0775
1634.55	47.1773
1634.97	44.2409
1635.39	42.3123
1635.81	40.9187
1636.24	46.9767

1636.66	47.9314
1637.08	47.2684
1637.5	46.4731
1637.92	46.6552
1638.34	39.7108
1638.76	43.3919
1639.18	45.6064
1639.6	45.8674
1640.02	44.5115
1640.44	46.4516
1640.86	46.4858
1641.28	44.4865
1641.7	47.1047
1642.12	43.3849
1642.54	43.7659
1642.96	47.1691
1643.38	42.8549
1643.8	39.8564
1644.22	43.8988
1644.64	50.3703
1645.06	41.4116
1645.49	46.3576
1645.91	42.6673
1646.32	38.1169
1646.75	42.847
1647.17	45.2089
1647.58	50.1997

1648	47.5216
1648.43	41.6447
1648.84	41.218
1649.26	43.4018
1649.69	44.6266
1650.1	44.2271
1650.52	42.1568
1650.94	43.7356
1651.36	44.2385
1651.78	44.624
1652.2	46.5539
1652.62	47.5221
1653.04	42.6714
1653.46	42.657
1653.88	45.6523
1654.3	42.6268
1654.72	46.1157
1655.14	44.6542
1655.56	44.1774
1655.98	46.1206
1656.4	41.6724
1656.82	46.095
1657.24	44.6473
1657.66	48.57
1658.07	46.2525
1658.49	46.5369
1658.91	49.2202

1659.33	45.0072
1659.75	45.6074
1660.17	43.09
1660.59	47.1218
1661.01	51.7811
1661.43	44.8319
1661.85	43.7684
1662.26	38.4412
1662.68	43.6277
1663.1	43.4981
1663.52	42.1701
1663.94	44.3213
1664.36	44.8854
1664.78	44.9618
1665.2	45.7127
1665.61	48.6027
1666.03	45.735
1666.45	45.5244
1666.87	40.7896
1667.29	46.3248
1667.71	44.5146
1668.13	44.0502
1668.54	43.1162
1668.96	43.1159
1669.38	42.8
1669.8	45.6745
1670.22	41.6624

1670.64	41.388
1671.05	40.9068
1671.47	48.3188
1671.89	42.5565
1672.31	44.5548
1672.73	45.9879
1673.14	44.6135
1673.56	44.2199
1673.98	47.2515
1674.4	40.0413
1674.82	45.5041
1675.23	43.0578
1675.65	41.9318
1676.07	46.5452
1676.49	39.8552
1676.91	41.8513
1677.32	46.945
1677.74	46.7139
1678.16	43.7254
1678.58	44.4893
1678.99	45.2593
1679.41	41.8678
1679.83	42.7473
1680.25	44.4901
1680.66	40.5901
1681.08	40.4211
1681.5	41.9424

1681.92	43.1047
1682.33	44.1598
1682.75	47.8342
1683.17	42.0884
1683.58	46.0201
1684	41.8206
1684.42	43.3587
1684.84	45.6267
1685.26	45.2201
1685.67	43.8034
1686.09	44.4975
1686.51	45.7
1686.92	46.4091
1687.34	44.5287
1687.76	43.0166
1688.17	44.4195
1688.59	36.1789
1689.01	44.6622
1689.42	43.7625
1689.84	45.0967
1690.26	40.6891
1690.67	47.858
1691.09	44.1189
1691.51	43.928
1691.92	45.9899
1692.34	47.8138
1692.76	44.2418

1693.17	44.2005
1693.59	41.8357
1694.01	45.4802
1694.42	41.7048
1694.84	43.9361
1695.26	43.7035
1695.67	44.2622
1696.09	42.1578
1696.51	41.0476
1696.92	45.4364
1697.34	42.7147
1697.76	45.1649
1698.17	45.1071
1698.59	42.8391
1699	40.5445
1699.42	41.3815
1699.84	44.6551
1700.25	44.322
1700.67	44.7416
1701.08	43.896
1701.5	47.4523
1701.91	40.5781
1702.33	44.3623
1702.75	47.9808
1703.16	48.3865
1703.58	50.5456
1703.99	46.3927

1704.41	45.6067
1704.83	41.5305
1705.24	40.6529
1705.66	41.5935
1706.07	46.049
1706.49	45.6161
1706.9	44.0449
1707.32	45.0482
1707.73	46.0829
1708.15	45.6328
1708.57	45.074
1708.98	46.9437
1709.4	43.937
1709.81	42.7871
1710.23	43.2099
1710.64	40.791
1711.06	44.475
1711.47	48.1795
1711.89	47.5267
1712.3	46.5778
1712.72	44.2971
1713.13	42.5187
1713.55	44.1839
1713.96	42.5536
1714.38	42.3715
1714.79	44.5238
1715.21	43.8283

1715.62	44.24
1716.04	47.3971
1716.45	47.4717
1716.87	44.6518
1717.28	41.2109
1717.7	41.2871
1718.11	41.9798
1718.53	42.1856
1718.94	42.6682
1719.36	41.51
1719.77	43.2675
1720.18	45.5125
1720.6	43.1903
1721.01	42.6473
1721.43	42.159
1721.84	42.7276
1722.26	39.0542
1722.67	43.0241
1723.09	47.9543
1723.5	48.9628
1723.91	46.9891
1724.33	42.4128
1724.74	42.2253
1725.16	44.0516
1725.57	43.4984
1725.98	43.6981
1726.4	43.2696

1726.81	43.5585
1727.23	41.6851
1727.64	42.0169
1728.05	43.6303
1728.47	39.6915
1728.88	44.5695
1729.3	40.9765
1729.71	41.6732
1730.13	43.5652
1730.54	42.0511
1730.95	43.48
1731.37	44.3745
1731.78	41.8541
1732.19	38.6567
1732.61	42.2131
1733.02	45.5832
1733.43	43.0954
1733.85	44.2206
1734.26	48.6417
1734.67	44.8002
1735.09	43.8724
1735.5	49.1939
1735.91	40.8079
1736.33	37.8252
1736.74	39.1022
1737.16	44.1827
1737.57	39.8103

1737.98	39.1348
1738.39	34.2856
1738.81	48.1723
1739.22	44.7718
1739.63	46.6333
1740.05	45.041
1740.46	43.0205
1740.87	43.0311
1741.28	44.0798
1741.7	49.7576
1742.11	38.3759
1742.52	40.1749
1742.94	40.675
1743.35	39.704
1743.76	43.1522
1744.18	44.1933
1744.59	45.0944
1745	40.8447
1745.41	41.1471
1745.83	39.4498
1746.24	36.0371
1746.65	45.1093
1747.06	45.4422
1747.48	48.8108
1747.89	47.0614
1748.3	45.2103
1748.71	48.6726

1749.13	38.2291
1749.54	41.1829
1749.95	44.8563
1750.36	45.0827
1750.77	45.2839
1751.19	45.4695
1751.6	42.049
1752.01	43.2461
1752.42	43.3258
1752.84	45.01
1753.25	40.2075
1753.66	44.6455
1754.07	40.444
1754.48	39.6604
1754.9	34.6251
1755.31	44.9184
1755.72	44.3042
1756.13	46.6426
1756.54	42.6943
1756.95	39.9769
1757.37	45.7716
1757.78	45.8906
1758.19	50.0211
1758.6	48.398
1759.01	47.4389
1759.42	45.9972
1759.84	48.496

1760.25	45.0893
1760.36	43.5337
1760.77	41.5412
1761.19	43.5486
1761.6	47.5517
1762.01	43.562
1762.43	43.583
1762.84	43.5992
1763.26	41.5952
1763.67	41.6506
1764.09	47.525
1764.5	41.5505
1764.91	43.5289
1765.33	45.5881
1765.74	43.5907
1766.16	39.7287
1766.57	47.5668
1766.98	41.7378
1767.4	43.8127
1767.81	43.7954
1768.23	37.8943
1768.64	41.7667
1769.05	45.6997
1769.47	41.7889
1769.88	41.8151
1770.29	39.6842
1770.71	41.8445

1771.12	39.9016
1771.54	43.775
1771.95	45.5629
1772.36	45.4249
1772.78	39.8943
1773.19	42.012
1773.6	43.7298
1774.02	51.2356
1774.43	41.7639
1774.84	45.6876
1775.26	45.9454
1775.67	45.6672
1776.08	51.3981
1776.5	43.5948
1776.91	45.9579
1777.33	39.8211
1777.74	49.3841
1778.15	44.1833
1778.56	49.1861
1778.98	43.9659
1779.39	47.6533
1779.8	45.9664
1780.22	38.576
1780.63	42.7287
1781.04	44.5379
1781.45	44.2232
1781.87	42.6222

1782.28	42.8152
1782.7	40.8633
1783.11	41.0914
1783.52	44.9003
1783.93	44.8841
1784.35	50.9785
1784.76	43.2358
1785.17	42.5561
1785.58	44.7789
1786	41.8841
1786.41	47.6504
1786.82	49.6502
1787.23	43.4264
1787.65	47.1522
1788.06	44.9144
1788.47	41.4001
1788.88	46.3695
1789.3	45.6973
1789.71	40.9659
1790.12	44.4726
1790.53	41.9903
1790.95	45.6389
1791.36	39.0527
1791.77	47.9226
1792.18	43.087
1792.59	37.2256
1793.01	49.861

1793.42	45.2297
1793.83	41.9316
1794.24	41.096
1794.66	49.116
1795.07	46.7987
1795.48	44.1627
1795.89	43.1932
1796.3	45.8218
1796.71	48.4427
1797.13	46.4556
1797.54	43.0296
1797.95	44.6995
1798.36	49.0994
1798.77	46.2278
1799.18	46.3769
1799.6	43.3297
1800.01	45.4193
1800.42	41.828
1800.83	43.0021
1801.24	45.9577
1801.65	44.2295
1802.06	47.5625
1802.48	44.4373
1802.89	49.5544
1803.3	45.5866
1803.71	43.6587
1804.12	48.2862

1804.53	48.594
1804.94	44.7269
1805.36	46.3617
1805.77	39.6002
1806.18	39.3507
1806.59	40.3591
1807	43.7967
1807.41	45.366
1807.82	41.9714
1808.23	47.1385
1808.64	45.7262
1809.06	48.7176
1809.46	48.5595
1809.88	47.7109
1810.29	44.6777
1810.7	47.2648
1811.11	45.6225
1811.52	49.5779
1811.93	43.246
1812.34	48.6073
1812.75	44.073
1813.16	38.0523
1813.57	48.7559
1813.98	36.8565
1814.39	41.6815
1814.8	44.5828
1815.21	46.8163

1815.62	47.0602
1816.04	41.4793
1816.45	45.4631
1816.86	39.8318
1817.27	43.0666
1817.68	44.5619
1818.09	44.0494
1818.5	45.506
1818.91	46.4839
1819.32	49.0359
1819.73	47.9169
1820.14	47.1096
1820.55	42.9268
1820.96	46.0559
1821.37	45.5415
1821.78	46.4986
1822.19	47.7606
1822.6	44.8675
1823.01	46.5586
1823.42	47.4837
1823.83	47.7239
1824.24	50.0422
1824.65	45.4772
1825.06	46.1603
1825.47	48.4224
1825.88	46.036
1826.29	46.0495

1826.69	47.3309
1827.1	45.3175
1827.51	43.8766
1827.92	45.5826
1828.33	45.9143
1828.74	42.3721
1829.15	42.2885
1829.56	47.3866
1829.97	43.2458
1830.38	45.3426
1830.79	46.2221
1831.2	41.413
1831.61	48.0445
1832.02	47.2185
1832.43	45.4092
1832.83	40.9936
1833.25	43.9057
1833.65	41.3346
1834.06	52.0033
1834.47	40.2348
1834.88	45.8824
1835.29	43.0812
1835.7	48.223
1836.11	50.532
1836.52	45.666
1836.93	48.1132
1837.33	49.169

1837.74	47.0215
1838.15	39.9578
1838.56	50.8895
1838.97	47.615
1839.38	42.8049
1839.79	44.1137
1840.2	47.349
1840.6	45.5845
1841.01	46.4177
1841.42	46.3753
1841.83	44.9441
1842.24	46.9241
1842.65	46.4364
1843.05	41.1773
1843.46	45.1373
1843.87	42.7824
1844.28	42.9992
1844.69	42.1451
1845.1	38.7891
1845.51	45.5194
1845.91	45.8869
1846.32	46.6607
1846.73	44.6691
1847.14	44.2655
1847.54	45.0773
1847.95	48.705
1848.36	39.6065

1848.77	47.0544
1849.18	50.235
1849.58	41.5654
1849.99	44.1885
1850.4	47.0265
1850.81	50.2133
1851.22	42.4783
1851.62	45.6881
1852.03	41.6996
1852.44	45.0586
1852.85	43.4751
1853.25	46.2006
1853.66	42.7997
1854.07	43.0433
1854.48	42.3618
1854.88	46.8192
1855.29	46.377
1855.7	51.4469
1856.11	43.0901
1856.51	48.1231
1856.92	45.3092
1857.33	41.0258
1857.74	40.8613
1858.14	41.614
1858.55	46.1442
1858.96	43.9068
1859.37	46.0728

1859.77	43.0612
1860.18	46.9936
1860.59	48.5722
1860.99	43.1899
1861.4	48.7884
1861.81	46.8442
1862.22	46.4841
1862.62	46.9709
1863.03	47.5342
1863.44	44.9
1863.84	45.4884
1864.25	44.2034
1864.66	43.5092
1865.06	51.098
1865.47	45.7589
1865.88	43.2484
1866.28	40.9213
1866.69	46.6001
1867.1	47.2452
1867.5	45.237
1867.91	46.211
1868.32	40.3538
1868.72	43.2131
1869.13	47.7689
1869.54	47.32
1869.94	47.0079
1870.35	39.8095

1870.75	43.2691
1871.16	46.1156
1871.57	44.5381
1871.97	44.842
1872.38	39.0047
1872.79	43.2814
1873.19	49.504
1873.6	44.0209
1874	45.1692
1874.41	42.6999
1874.82	43.9576
1875.22	40.9779
1875.63	45.5198
1876.03	42.6122
1876.44	44.8345
1876.85	43.6872
1877.25	45.5175
1877.66	44.4153
1878.06	43.3478
1878.47	44.3318
1878.88	47.9043
1879.28	43.6482
1879.69	40.8986
1880.09	43.6949
1880.5	44.9604
1880.9	47.8222
1881.31	46.6335

1881.72	43.9881
1882.12	42.106
1882.53	46.0849
1882.93	42.7161
1883.34	45.6415
1883.74	48.1872
1884.15	45.211
1884.55	49.1709
1884.96	49.6513
1885.36	42.9368
1885.77	43.0426
1886.17	41.3045
1886.58	42.8931
1886.98	44.2706
1887.39	44.6727
1887.79	43.7835
1888.2	45.0096
1888.6	44.918
1889.01	50.2411
1889.41	46.1899
1889.82	49.9018
1890.22	44.038
1890.63	49.8967
1891.03	45.72
1891.44	46.2652
1891.84	45.9415
1892.25	44.5118

1892.65	43.188
1893.06	41.1401
1893.46	44.0057
1893.87	44.5084
1894.27	45.5523
1894.67	46.7249
1895.08	41.4915
1895.48	47.9471
1895.89	43.6183
1896.29	40.7294
1896.7	40.9197
1897.1	42.4618
1897.51	46.3769
1897.91	41.2252
1898.32	44.2611
1898.72	43.2257
1899.12	48.1371
1899.53	44.1993
1899.93	49.1972
1900.34	43.3498
1900.74	45.5156
1901.15	45.932
1901.55	47.9262
1901.95	49.3173
1902.36	42.2085
1902.76	45.8429
1903.16	47.4698

1903.57	45.9533
1903.97	46.5774
1904.38	47.8602
1904.78	46.1884
1905.18	43.0624
1905.59	46.2899
1905.99	43.8776
1906.4	45.1014
1906.8	48.2634
1907.2	47.8702
1907.61	47.1587
1908.01	49.4164
1908.41	40.2042
1908.82	46.3232
1909.22	43.4079
1909.62	45.026
1910.03	48.1391
1910.43	44.4908
1910.83	46.203
1911.24	45.3447
1911.64	43.3878
1912.04	47.3966
1912.45	46.445
1912.85	48.5329
1913.25	43.3663
1913.66	44.9663
1914.06	44.0465

1914.46	44.0551
1914.87	44.8848
1915.27	47.4917
1915.67	49.1303
1916.07	48.2224
1916.48	47.1195
1916.88	48.253
1917.28	45.5765
1917.69	50.7469
1918.09	50.6483
1918.49	46.4052
1918.89	51.7421
1919.3	47.4827
1919.7	44.6193
1920.1	44.6596
1920.5	47.5619
1920.91	46.9607
1921.31	54.7119
1921.71	49.275
1922.12	48.4626
1922.52	47.4419
1922.92	44.299
1923.32	49.427
1923.73	52.1946
1924.13	48.1946
1924.53	42.7305
1924.93	44.6507

1925.33	45.8055
1925.74	43.0066
1926.14	45.8786
1926.54	43.2728
1926.94	45.9193
1927.35	44.5793
1927.75	42.0712
1928.15	43.2012
1928.55	47.5507
1928.95	51.5214
1929.36	47.6697
1929.76	44.382
1930.16	48.0861
1930.56	44.476
1930.96	43.5924
1931.36	45.2994
1931.77	47.4722
1932.17	46.8911
1932.57	48.3212
1932.97	45.6008
1933.37	47.5856
1933.78	49.5811
1934.18	42.8661
1934.58	47.5363
1934.98	50.3839
1935.38	45.6636
1935.78	44.7939

1936.18	47.6946
1936.59	48.1792
1936.99	43.6542
1937.39	46.8724
1937.79	50.5082
1938.19	50.9059
1938.59	48.439
1938.99	48.7214
1939.4	49.2381
1939.8	46.8038
1940.2	43.6042
1940.6	43.6038
1941	42.169
1941.4	47.2558
1941.8	49.3048
1942.2	47.9896
1942.6	47.7712
1943.01	46.4458
1943.41	49.3229
1943.81	46.9832
1944.21	44.9386
1944.61	48.3576
1945.01	47.7488
1945.41	47.5693
1945.81	47.9427
1946.21	45.9908
1946.61	48.8106

1947.01	42.9124
1947.41	49.2217
1947.81	50.0451
1948.22	48.6383
1948.62	47.119
1949.02	50.7713
1949.42	48.6218
1949.82	48.9352
1950.22	46.4325
1950.62	45.8488
1951.02	44.1427
1951.42	48.1189
1951.82	49.1289
1952.22	43.6694
1952.62	47.082
1953.02	49.6619
1953.42	50.1037
1953.82	43.9797
1954.22	48.5262
1954.62	47.9221
1955.02	48.7898
1955.42	42.5509
1955.82	43.386
1956.22	47.8265
1956.62	51.1878
1957.02	46.1357
1957.42	48.617

1957.82	49.1815
1958.22	48.3971
1958.62	44.521
1959.02	44.6711
1959.42	45.2804
1959.82	40.4848
1960.22	47.7444
1960.62	45.1264
1961.02	49.2248
1961.42	41.2335
1961.82	48.0342
1962.22	47.3744
1962.62	38.7136
1963.02	45.1961
1963.42	47.2844
1963.81	44.2537
1964.21	44.7056
1964.61	48.3829
1965.01	48.8076
1965.41	41.9245
1965.81	47.0358
1966.21	51.5476
1966.61	47.9689
1967.01	49.0052
1967.41	42.1407
1967.81	46.0181
1968.21	57.3192

1968.61	45.1156
1969	48.0359
1969.4	47.1787
1969.46	46.8905
1969.86	46.9675
1970.26	56.4962
1970.66	43.4945
1971.07	45.4941
1971.47	38.4222
1971.87	45.403
1972.27	46.4289
1972.67	40.9801
1973.07	44.8532
1973.48	43.0299
1973.88	41.7601
1974.28	44.3496
1974.68	46.2631
1975.08	47.123
1975.48	47.8707
1975.89	40.1716
1976.29	42.4485
1976.69	50.9003
1977.09	40.4264
1977.49	42.5154
1977.89	40.1331
1978.29	46.8706
1978.7	44.2065

1979.1	51.1157
1979.5	40.7134
1979.9	44.5764
1980.3	42.9706
1980.7	40.4234
1981.1	44.2497
1981.5	44.0102
1981.9	46.717
1982.3	44.2472
1982.71	46.011
1983.11	42.1429
1983.51	45.4978
1983.91	45.8609
1984.31	47.7529
1984.71	49.8989
1985.11	48.3331
1985.51	45.3356
1985.91	47.3628
1986.31	44.5714
1986.71	39.5269
1987.11	40.9012
1987.51	40.9977
1987.92	37.8603
1988.32	44.7506
1988.72	44.1078
1989.12	46.8026
1989.52	39.4034

1989.92	44.4401
1990.32	45.0879
1990.72	47.3981
1991.12	44.7553
1991.52	43.6253
1991.92	41.6321
1992.32	40.4551
1992.72	40.7909
1993.12	41.1823
1993.52	41.5885
1993.92	44.4908
1994.32	41.083
1994.72	43.5043
1995.12	43.5154
1995.52	43.7615
1995.92	43.999
1996.32	41.993
1996.72	43.7605
1997.12	42.763
1997.52	44.6159
1997.92	45.7893
1998.32	51.3602
1998.72	46.1875
1999.12	42.5148
1999.52	46.2267
1999.92	41.9386

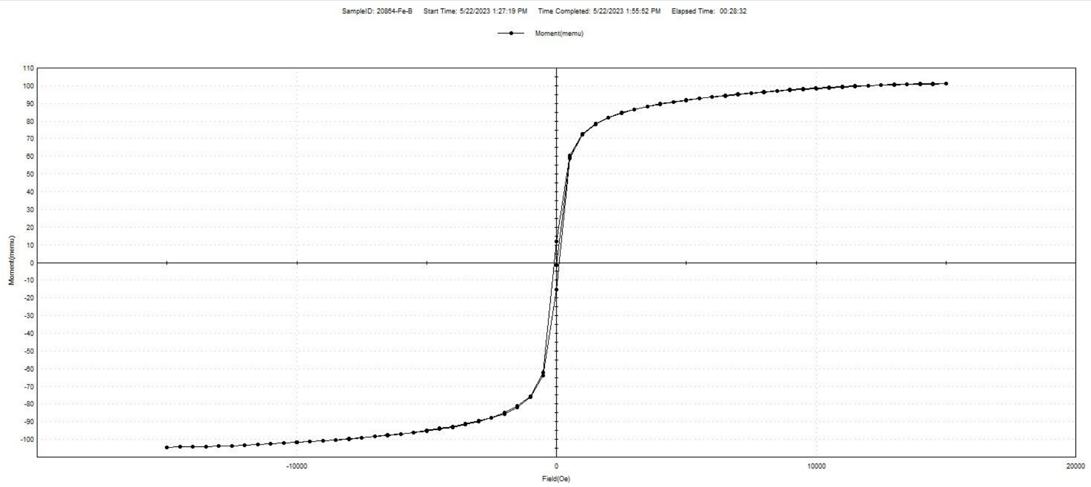

Area-Total: 67.868 erg
Average Time: 3 sec
Coercivity (Hci): 91.009 Oe
Field Increment: 500 Oe
Magnetization (Ms): 0.10291 emu
Mass: 9.0000E-3 g
Number of Points: 151
Retentivity (Mr): 13.491E-3 emu
Sensitivity: -110 emu
Time Constant: 1 sec

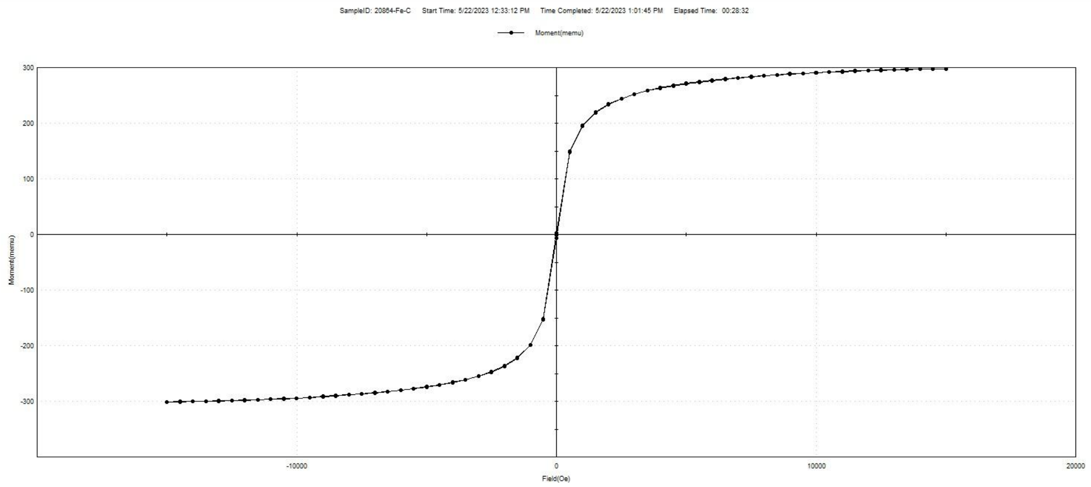

Area-Total: 161.18 erg
Average Time: 3 sec
Coercivity (Hci): 15.783 Oe
Field Increment: 500 Oe
Magnetization (Ms): 0.29921 emu
Mass: 14.300E-3 g
Number of Points: 151
Retentivity (Mr): 4.8962E-3 emu
Sensitivity: -110 emu
Time Constant: 1 sec

;RAW4.00

[RawHeader]

Date=05/11/2023

EstimatedTotalTime=828.644

FurtherDQLReading=0

HardwareIndicator=65519

MeasuredTotalTime=828.644

MeasurementFlag=1

NumberOfMeasuredRanges=1

NumberOfRanges=1

Time=10:35:10

[VarInfo]

Type=USER

Flags=0

Value=Lab Manager

[VarInfo]

Type=SAMPLEID

Flags=0

Value=

[VarInfo]

Type=COMMENT

Flags=0

Value=

[VarInfo]

Type=UTF

Flags=0

Value=

[VarInfo]

Type=CREATOR

Flags=0

Value=BrmlToV4Converter

[VarInfo]

Type=CREATOR_VERSION

Flags=0

Value=6.5.0.0

[HardwareConfiguration]

AbsorptionFactor=9999

ActivateAbsorber=0

Alpha1=1.5406

Alpha2=1.54439

AlphaAverage=1.5418

AlphaRatio=0.5

Analyzer=0

Anode=Cu

AntiScatteringSlit=9999

BeamOpticsFlags=0

Beta=1.39222

BetaRelativeIntensity=0

DeactivateAbsorber=0

DetectorSlit=9999

DivergenceSlit=10.5

GoniometerControl=0

GoniometerDiameter=560

GoniometerModel=512

GoniometerStage=7

Monochromator=0

NearSampleSlit=9999

PrimarySollerSlit=2.5

SampleChanger=0

SecondSollerSlit=9999

SynchronousAxis=0

ThinFilmAttachment=9999

WaveUnit=A

[RangeHeader]

ActuallyUsedLambda=1.5418

AdditionalDetectorMask=257

DataRecordLength=4

DelayTime=0

DisplayPlaneNumber=0

EstimatedScanTime=829

ExtraParametersMask=0

GeneratorCurrent=30

GeneratorVoltage=40

Increment=0.0194583

Increment3=0

NumberOfCompletedData=4363

NumberOfCounts=1

NumberOfDetectors=0

NumberOfDrives=3

NumberOfEncoderDrives=0

NumberOfMeasuredData=4363

NumberOfVaryingParameters=0

RangeStartTime=0

RotationSpeed=15

ScanMode=1

ScanType=Locked Coupled

SimulatedMeasConditions=0

SlitChangerIn=0

SmootingWidth=0

Start=5

Steps=4363

SynchronousRotation=0

Time=34.56

[VarInfo]

Type=PSD_DISCRIM

Flags=0

Value=0.11;0.25

[PSD]

ActualTwoTheta=9999

FirstUsedChannel=0

FixedPSD=0

PSDOpening=2.94947

PSDType=5

[Drive]

DriveFlags=1

DriveName=2Theta

DriveNumber=2

DriveOffset=0

OffsetIndex=0

OscillationAmplitude=0

OscillationSpeed=0

StartPosition=5

[Drive]

DriveFlags=1

DriveName=Theta

DriveNumber=1

DriveOffset=0

OffsetIndex=0

OscillationAmplitude=0

OscillationSpeed=0

StartPosition=2.5

[Drive]

DriveFlags=1

DriveName=Phi

DriveNumber=3

DriveOffset=0

OffsetIndex=0

OscillationAmplitude=0

OscillationSpeed=0

StartPosition=53.6016

[Data]

Angle,	PSD,
5,	271,
5.01946,	279,
5.03892,	243,
5.05837,	280,
5.07783,	272,
5.09729,	264,
5.11675,	243,
5.13621,	264,
5.15567,	242,
5.17512,	268,
5.19458,	245,
5.21404,	244,
5.2335,	218,
5.25296,	235,
5.27242,	234,
5.29187,	237,

5.31133,	245,
5.33079,	191,
5.35025,	199,
5.36971,	204,
5.38917,	188,
5.40862,	198,
5.42808,	176,
5.44754,	181,
5.467,	179,
5.48646,	156,
5.50592,	177,
5.52537,	142,
5.54483,	168,
5.56429,	137,
5.58375,	126,
5.60321,	140,
5.62266,	121,
5.64212,	136,
5.66158,	124,
5.68104,	127,
5.7005,	134,
5.71996,	107,
5.73941,	123,
5.75887,	123,
5.77833,	114,
5.79779,	131,
5.81725,	108,

5.83671,	104,
5.85616,	104,
5.87562,	106,
5.89508,	117,
5.91454,	99,
5.934,	94,
5.95346,	93,
5.97291,	80,
5.99237,	93,
6.01183,	108,
6.03129,	92,
6.05075,	102,
6.07021,	94,
6.08966,	97,
6.10912,	91,
6.12858,	103,
6.14804,	78,
6.1675,	93,
6.18695,	99,
6.20641,	88,
6.22587,	86,
6.24533,	98,
6.26479,	90,
6.28425,	87,
6.3037,	94,
6.32316,	89,
6.34262,	84,

6.36208, 87,

6.38154, 92,

6.401, 75,

6.42045, 86,

6.43991, 94,

6.45937, 80,

6.47883, 95,

6.49829, 88,

6.51775, 86,

6.5372, 82,

6.55666, 65,

6.57612, 84,

6.59558, 81,

6.61504, 85,

6.6345, 80,

6.65395, 68,

6.67341, 64,

6.69287, 76,

6.71233, 82,

6.73179, 80,

6.75124, 71,

6.7707, 98,

6.79016, 106,

6.80962, 88,

6.82908, 94,

6.84854, 84,

6.86799, 85,

6.88745, 67,

6.90691, 77,

6.92637, 76,

6.94583, 82,

6.96529, 65,

6.98474, 78,

7.0042, 82,

7.02366, 88,

7.04312, 78,

7.06258, 84,

7.08204, 75,

7.10149, 74,

7.12095, 62,

7.14041, 79,

7.15987, 88,

7.17933, 76,

7.19879, 80,

7.21824, 62,

7.2377, 73,

7.25716, 80,

7.27662, 87,

7.29608, 67,

7.31553, 89,

7.33499, 69,

7.35445, 82,

7.37391, 80,

7.39337, 94,

7.41283, 75,

7.43228, 77,

7.45174, 74,

7.4712, 70,

7.49066, 84,

7.51012, 74,

7.52958, 76,

7.54903, 75,

7.56849, 75,

7.58795, 56,

7.60741, 74,

7.62687, 61,

7.64633, 70,

7.66578, 73,

7.68524, 83,

7.7047, 84,

7.72416, 73,

7.74362, 81,

7.76308, 69,

7.78253, 76,

7.80199, 83,

7.82145, 71,

7.84091, 79,

7.86037, 69,

7.87983, 78,

7.89928, 73,

7.91874, 69,

7.9382,	76,
7.95766,	85,
7.97712,	77,
7.99657,	66,
8.01603,	71,
8.03549,	65,
8.05495,	78,
8.07441,	72,
8.09387,	71,
8.11332,	76,
8.13278,	70,
8.15224,	95,
8.1717,	62,
8.19116,	56,
8.21062,	68,
8.23007,	64,
8.24953,	75,
8.26899,	81,
8.28845,	67,
8.30791,	60,
8.32737,	81,
8.34682,	76,
8.36628,	85,
8.38574,	92,
8.4052,	83,
8.42466,	72,
8.44412,	74,

8.46357, 70,

8.48303, 68,

8.50249, 93,

8.52195, 66,

8.54141, 76,

8.56086, 79,

8.58032, 73,

8.59978, 85,

8.61924, 77,

8.6387, 78,

8.65816, 73,

8.67761, 80,

8.69707, 82,

8.71653, 73,

8.73599, 70,

8.75545, 77,

8.77491, 81,

8.79436, 83,

8.81382, 67,

8.83328, 74,

8.85274, 81,

8.8722, 73,

8.89166, 96,

8.91111, 78,

8.93057, 82,

8.95003, 64,

8.96949, 78,

8.98895, 73,

9.00841, 66,

9.02786, 83,

9.04732, 75,

9.06678, 71,

9.08624, 90,

9.1057, 98,

9.12515, 74,

9.14461, 82,

9.16407, 93,

9.18353, 97,

9.20299, 98,

9.22245, 77,

9.2419, 87,

9.26136, 77,

9.28082, 89,

9.30028, 87,

9.31974, 96,

9.3392, 93,

9.35865, 105,

9.37811, 96,

9.39757, 102,

9.41703, 83,

9.43649, 79,

9.45595, 109,

9.4754, 104,

9.49486, 83,

9.51432,	89,
9.53378,	129,
9.55324,	99,
9.5727,	104,
9.59215,	91,
9.61161,	83,
9.63107,	89,
9.65053,	94,
9.66999,	84,
9.68944,	95,
9.7089,	87,
9.72836,	109,
9.74782,	104,
9.76728,	109,
9.78674,	103,
9.80619,	94,
9.82565,	105,
9.84511,	94,
9.86457,	97,
9.88403,	116,
9.90349,	105,
9.92294,	92,
9.9424,	102,
9.96186,	125,
9.98132,	112,
10.0008,	108,
10.0202,	81,

10.0397,	101,
10.0592,	104,
10.0786,	107,
10.0981,	94,
10.1175,	94,
10.137,	102,
10.1564,	95,
10.1759,	89,
10.1954,	98,
10.2148,	122,
10.2343,	105,
10.2537,	110,
10.2732,	104,
10.2927,	100,
10.3121,	116,
10.3316,	119,
10.351,	108,
10.3705,	109,
10.3899,	116,
10.4094,	91,
10.4289,	123,
10.4483,	110,
10.4678,	123,
10.4872,	106,
10.5067,	105,
10.5262,	119,
10.5456,	111,

10.5651,	112,
10.5845,	133,
10.604,	111,
10.6234,	128,
10.6429,	135,
10.6624,	125,
10.6818,	121,
10.7013,	105,
10.7207,	114,
10.7402,	84,
10.7597,	122,
10.7791,	113,
10.7986,	101,
10.818,	114,
10.8375,	129,
10.8569,	114,
10.8764,	119,
10.8959,	110,
10.9153,	124,
10.9348,	123,
10.9542,	106,
10.9737,	124,
10.9931,	113,
11.0126,	118,
11.0321,	107,
11.0515,	118,
11.071,	141,

11.0904,	130,
11.1099,	115,
11.1294,	131,
11.1488,	132,
11.1683,	142,
11.1877,	124,
11.2072,	111,
11.2266,	110,
11.2461,	94,
11.2656,	111,
11.285,	122,
11.3045,	136,
11.3239,	122,
11.3434,	103,
11.3629,	113,
11.3823,	136,
11.4018,	129,
11.4212,	123,
11.4407,	106,
11.4601,	121,
11.4796,	126,
11.4991,	128,
11.5185,	124,
11.538,	125,
11.5574,	134,
11.5769,	109,
11.5964,	120,

11.6158,	102,
11.6353,	106,
11.6547,	114,
11.6742,	117,
11.6936,	127,
11.7131,	109,
11.7326,	118,
11.752,	129,
11.7715,	142,
11.7909,	128,
11.8104,	123,
11.8299,	137,
11.8493,	121,
11.8688,	117,
11.8882,	128,
11.9077,	122,
11.9271,	127,
11.9466,	127,
11.9661,	105,
11.9855,	120,
12.005,	114,
12.0244,	121,
12.0439,	129,
12.0634,	133,
12.0828,	149,
12.1023,	111,
12.1217,	126,

12.1412,	157,
12.1606,	128,
12.1801,	134,
12.1996,	118,
12.219,	119,
12.2385,	125,
12.2579,	120,
12.2774,	135,
12.2969,	126,
12.3163,	124,
12.3358,	119,
12.3552,	121,
12.3747,	119,
12.3941,	143,
12.4136,	109,
12.4331,	108,
12.4525,	120,
12.472,	148,
12.4914,	147,
12.5109,	127,
12.5304,	137,
12.5498,	143,
12.5693,	143,
12.5887,	146,
12.6082,	146,
12.6276,	124,
12.6471,	139,

12.6666,	122,
12.686,	133,
12.7055,	129,
12.7249,	135,
12.7444,	136,
12.7639,	128,
12.7833,	131,
12.8028,	112,
12.8222,	113,
12.8417,	143,
12.8611,	120,
12.8806,	150,
12.9001,	144,
12.9195,	133,
12.939,	137,
12.9584,	152,
12.9779,	139,
12.9974,	142,
13.0168,	136,
13.0363,	127,
13.0557,	133,
13.0752,	125,
13.0946,	145,
13.1141,	142,
13.1336,	116,
13.153,	129,
13.1725,	126,

13.1919,	123,
13.2114,	119,
13.2309,	127,
13.2503,	130,
13.2698,	132,
13.2892,	126,
13.3087,	146,
13.3281,	152,
13.3476,	152,
13.3671,	128,
13.3865,	126,
13.406,	136,
13.4254,	142,
13.4449,	140,
13.4644,	142,
13.4838,	142,
13.5033,	129,
13.5227,	135,
13.5422,	123,
13.5616,	135,
13.5811,	130,
13.6006,	148,
13.62,	147,
13.6395,	140,
13.6589,	126,
13.6784,	151,
13.6978,	155,

13.7173,	148,
13.7368,	144,
13.7562,	110,
13.7757,	135,
13.7951,	136,
13.8146,	131,
13.8341,	152,
13.8535,	142,
13.873,	132,
13.8924,	140,
13.9119,	131,
13.9313,	119,
13.9508,	137,
13.9703,	121,
13.9897,	120,
14.0092,	131,
14.0286,	145,
14.0481,	151,
14.0676,	150,
14.087,	136,
14.1065,	140,
14.1259,	133,
14.1454,	137,
14.1648,	142,
14.1843,	154,
14.2038,	129,
14.2232,	133,

14.2427, 146,

14.2621, 135,

14.2816, 156,

14.3011, 143,

14.3205, 131,

14.34, 136,

14.3594, 156,

14.3789, 144,

14.3983, 170,

14.4178, 122,

14.4373, 143,

14.4567, 134,

14.4762, 131,

14.4956, 153,

14.5151, 129,

14.5346, 127,

14.554, 126,

14.5735, 150,

14.5929, 142,

14.6124, 130,

14.6318, 130,

14.6513, 130,

14.6708, 128,

14.6902, 136,

14.7097, 138,

14.7291, 121,

14.7486, 145,

14.7681,	148,
14.7875,	151,
14.807,	127,
14.8264,	147,
14.8459,	144,
14.8653,	127,
14.8848,	129,
14.9043,	147,
14.9237,	151,
14.9432,	148,
14.9626,	138,
14.9821,	136,
15.0016,	148,
15.021,	143,
15.0405,	152,
15.0599,	158,
15.0794,	165,
15.0988,	154,
15.1183,	167,
15.1378,	148,
15.1572,	157,
15.1767,	123,
15.1961,	143,
15.2156,	135,
15.2351,	135,
15.2545,	147,
15.274,	131,

15.2934,	147,
15.3129,	117,
15.3323,	140,
15.3518,	160,
15.3713,	131,
15.3907,	151,
15.4102,	130,
15.4296,	132,
15.4491,	146,
15.4686,	133,
15.488,	149,
15.5075,	131,
15.5269,	125,
15.5464,	137,
15.5658,	148,
15.5853,	148,
15.6048,	143,
15.6242,	167,
15.6437,	142,
15.6631,	140,
15.6826,	147,
15.7021,	151,
15.7215,	157,
15.741,	142,
15.7604,	152,
15.7799,	160,
15.7993,	148,

15.8188,	132,
15.8383,	129,
15.8577,	137,
15.8772,	118,
15.8966,	121,
15.9161,	140,
15.9356,	146,
15.955,	146,
15.9745,	145,
15.9939,	156,
16.0134,	142,
16.0328,	143,
16.0523,	140,
16.0718,	131,
16.0912,	127,
16.1107,	153,
16.1301,	142,
16.1496,	130,
16.1691,	146,
16.1885,	144,
16.208,	132,
16.2274,	144,
16.2469,	153,
16.2663,	159,
16.2858,	128,
16.3053,	132,
16.3247,	149,

16.3442,	148,
16.3636,	146,
16.3831,	135,
16.4026,	139,
16.422,	140,
16.4415,	142,
16.4609,	124,
16.4804,	141,
16.4998,	160,
16.5193,	145,
16.5388,	154,
16.5582,	135,
16.5777,	137,
16.5971,	148,
16.6166,	124,
16.636,	130,
16.6555,	151,
16.675,	146,
16.6944,	146,
16.7139,	136,
16.7333,	149,
16.7528,	129,
16.7723,	156,
16.7917,	121,
16.8112,	153,
16.8306,	147,
16.8501,	143,

16.8695,	144,
16.889,	154,
16.9085,	135,
16.9279,	138,
16.9474,	151,
16.9668,	149,
16.9863,	148,
17.0058,	156,
17.0252,	167,
17.0447,	157,
17.0641,	149,
17.0836,	144,
17.103,	136,
17.1225,	143,
17.142,	153,
17.1614,	144,
17.1809,	179,
17.2003,	152,
17.2198,	143,
17.2393,	148,
17.2587,	148,
17.2782,	149,
17.2976,	152,
17.3171,	134,
17.3365,	136,
17.356,	130,
17.3755,	145,

17.3949,	152,
17.4144,	122,
17.4338,	139,
17.4533,	140,
17.4728,	159,
17.4922,	142,
17.5117,	136,
17.5311,	150,
17.5506,	155,
17.57,	130,
17.5895,	150,
17.609,	150,
17.6284,	147,
17.6479,	126,
17.6673,	124,
17.6868,	137,
17.7063,	134,
17.7257,	154,
17.7452,	150,
17.7646,	146,
17.7841,	142,
17.8035,	148,
17.823,	136,
17.8425,	159,
17.8619,	157,
17.8814,	145,
17.9008,	147,

17.9203,	160,
17.9398,	150,
17.9592,	168,
17.9787,	132,
17.9981,	151,
18.0176,	157,
18.037,	138,
18.0565,	166,
18.076,	145,
18.0954,	149,
18.1149,	145,
18.1343,	138,
18.1538,	173,
18.1733,	162,
18.1927,	142,
18.2122,	137,
18.2316,	135,
18.2511,	154,
18.2705,	160,
18.29,	147,
18.3095,	156,
18.3289,	154,
18.3484,	159,
18.3678,	154,
18.3873,	135,
18.4068,	159,
18.4262,	158,

18.4457,	142,
18.4651,	171,
18.4846,	128,
18.504,	150,
18.5235,	156,
18.543,	154,
18.5624,	164,
18.5819,	134,
18.6013,	152,
18.6208,	158,
18.6403,	131,
18.6597,	141,
18.6792,	168,
18.6986,	138,
18.7181,	155,
18.7375,	157,
18.757,	157,
18.7765,	165,
18.7959,	154,
18.8154,	152,
18.8348,	151,
18.8543,	157,
18.8738,	160,
18.8932,	191,
18.9127,	135,
18.9321,	172,
18.9516,	142,

18.971,	127,
18.9905,	167,
19.01,	154,
19.0294,	153,
19.0489,	168,
19.0683,	162,
19.0878,	136,
19.1073,	165,
19.1267,	172,
19.1462,	128,
19.1656,	153,
19.1851,	136,
19.2045,	154,
19.224,	165,
19.2435,	138,
19.2629,	149,
19.2824,	137,
19.3018,	165,
19.3213,	156,
19.3408,	148,
19.3602,	138,
19.3797,	163,
19.3991,	147,
19.4186,	148,
19.438,	150,
19.4575,	164,
19.477,	165,

19.4964,	143,
19.5159,	143,
19.5353,	158,
19.5548,	155,
19.5742,	166,
19.5937,	138,
19.6132,	178,
19.6326,	144,
19.6521,	149,
19.6715,	167,
19.691,	140,
19.7105,	163,
19.7299,	148,
19.7494,	138,
19.7688,	137,
19.7883,	132,
19.8077,	147,
19.8272,	158,
19.8467,	168,
19.8661,	151,
19.8856,	169,
19.905,	167,
19.9245,	154,
19.944,	157,
19.9634,	145,
19.9829,	168,
20.0023,	168,

20.0218, 143,

20.0412, 147,

20.0607, 162,

20.0802, 159,

20.0996, 146,

20.1191, 153,

20.1385, 149,

20.158, 150,

20.1775, 165,

20.1969, 180,

20.2164, 173,

20.2358, 153,

20.2553, 149,

20.2747, 153,

20.2942, 123,

20.3137, 162,

20.3331, 147,

20.3526, 146,

20.372, 154,

20.3915, 172,

20.411, 153,

20.4304, 159,

20.4499, 137,

20.4693, 163,

20.4888, 149,

20.5082, 143,

20.5277, 133,

20.5472, 143,

20.5666, 139,

20.5861, 160,

20.6055, 160,

20.625, 138,

20.6445, 161,

20.6639, 163,

20.6834, 171,

20.7028, 149,

20.7223, 151,

20.7417, 143,

20.7612, 143,

20.7807, 178,

20.8001, 136,

20.8196, 171,

20.839, 155,

20.8585, 152,

20.878, 152,

20.8974, 170,

20.9169, 152,

20.9363, 151,

20.9558, 161,

20.9752, 156,

20.9947, 160,

21.0142, 155,

21.0336, 149,

21.0531, 169,

21.0725,	142,
21.092,	166,
21.1115,	151,
21.1309,	146,
21.1504,	133,
21.1698,	161,
21.1893,	180,
21.2087,	172,
21.2282,	151,
21.2477,	164,
21.2671,	146,
21.2866,	146,
21.306,	157,
21.3255,	150,
21.345,	162,
21.3644,	150,
21.3839,	153,
21.4033,	157,
21.4228,	160,
21.4422,	164,
21.4617,	174,
21.4812,	154,
21.5006,	173,
21.5201,	151,
21.5395,	163,
21.559,	162,
21.5785,	157,

21.5979,	153,
21.6174,	165,
21.6368,	156,
21.6563,	156,
21.6757,	148,
21.6952,	164,
21.7147,	152,
21.7341,	146,
21.7536,	183,
21.773,	144,
21.7925,	166,
21.812,	155,
21.8314,	147,
21.8509,	155,
21.8703,	169,
21.8898,	175,
21.9092,	166,
21.9287,	156,
21.9482,	163,
21.9676,	164,
21.9871,	170,
22.0065,	130,
22.026,	142,
22.0455,	145,
22.0649,	151,
22.0844,	145,
22.1038,	151,

22.1233, 149,

22.1427, 165,

22.1622, 152,

22.1817, 171,

22.2011, 147,

22.2206, 173,

22.24, 142,

22.2595, 167,

22.279, 162,

22.2984, 151,

22.3179, 163,

22.3373, 154,

22.3568, 156,

22.3762, 158,

22.3957, 152,

22.4152, 135,

22.4346, 167,

22.4541, 146,

22.4735, 147,

22.493, 153,

22.5124, 167,

22.5319, 175,

22.5514, 169,

22.5708, 177,

22.5903, 142,

22.6097, 166,

22.6292, 139,

22.6487,	165,
22.6681,	128,
22.6876,	160,
22.707,	166,
22.7265,	172,
22.7459,	155,
22.7654,	183,
22.7849,	155,
22.8043,	164,
22.8238,	162,
22.8432,	175,
22.8627,	171,
22.8822,	168,
22.9016,	166,
22.9211,	142,
22.9405,	187,
22.96,	155,
22.9794,	186,
22.9989,	162,
23.0184,	189,
23.0378,	170,
23.0573,	178,
23.0767,	152,
23.0962,	140,
23.1157,	161,
23.1351,	141,
23.1546,	165,

23.174,	145,
23.1935,	178,
23.2129,	158,
23.2324,	183,
23.2519,	149,
23.2713,	136,
23.2908,	168,
23.3102,	154,
23.3297,	149,
23.3492,	153,
23.3686,	164,
23.3881,	162,
23.4075,	160,
23.427,	154,
23.4464,	159,
23.4659,	173,
23.4854,	155,
23.5048,	172,
23.5243,	143,
23.5437,	159,
23.5632,	178,
23.5827,	161,
23.6021,	164,
23.6216,	161,
23.641,	156,
23.6605,	168,
23.6799,	138,

23.6994,	147,
23.7189,	184,
23.7383,	145,
23.7578,	161,
23.7772,	173,
23.7967,	156,
23.8162,	166,
23.8356,	162,
23.8551,	175,
23.8745,	185,
23.894,	166,
23.9134,	147,
23.9329,	171,
23.9524,	157,
23.9718,	177,
23.9913,	142,
24.0107,	170,
24.0302,	159,
24.0497,	163,
24.0691,	157,
24.0886,	144,
24.108,	172,
24.1275,	164,
24.1469,	144,
24.1664,	168,
24.1859,	160,
24.2053,	182,

24.2248,	157,
24.2442,	167,
24.2637,	163,
24.2832,	153,
24.3026,	168,
24.3221,	164,
24.3415,	156,
24.361,	159,
24.3804,	134,
24.3999,	164,
24.4194,	180,
24.4388,	194,
24.4583,	171,
24.4777,	176,
24.4972,	189,
24.5167,	153,
24.5361,	157,
24.5556,	162,
24.575,	165,
24.5945,	176,
24.6139,	167,
24.6334,	149,
24.6529,	165,
24.6723,	156,
24.6918,	172,
24.7112,	167,
24.7307,	158,

24.7502, 165,

24.7696, 156,

24.7891, 165,

24.8085, 173,

24.828, 169,

24.8474, 158,

24.8669, 172,

24.8864, 154,

24.9058, 167,

24.9253, 177,

24.9447, 158,

24.9642, 162,

24.9837, 156,

25.0031, 165,

25.0226, 146,

25.042, 173,

25.0615, 158,

25.0809, 179,

25.1004, 160,

25.1199, 148,

25.1393, 184,

25.1588, 186,

25.1782, 164,

25.1977, 167,

25.2171, 170,

25.2366, 156,

25.2561, 180,

25.2755, 173,

25.295, 175,

25.3144, 162,

25.3339, 171,

25.3534, 172,

25.3728, 176,

25.3923, 180,

25.4117, 187,

25.4312, 189,

25.4506, 154,

25.4701, 166,

25.4896, 152,

25.509, 181,

25.5285, 144,

25.5479, 184,

25.5674, 158,

25.5869, 146,

25.6063, 166,

25.6258, 157,

25.6452, 167,

25.6647, 154,

25.6841, 177,

25.7036, 169,

25.7231, 182,

25.7425, 158,

25.762, 188,

25.7814, 155,

25.8009,	152,
25.8204,	158,
25.8398,	188,
25.8593,	152,
25.8787,	189,
25.8982,	166,
25.9176,	175,
25.9371,	155,
25.9566,	179,
25.976,	169,
25.9955,	170,
26.0149,	155,
26.0344,	171,
26.0539,	167,
26.0733,	166,
26.0928,	147,
26.1122,	182,
26.1317,	168,
26.1511,	171,
26.1706,	167,
26.1901,	168,
26.2095,	181,
26.229,	159,
26.2484,	151,
26.2679,	170,
26.2874,	170,
26.3068,	165,

26.3263,	169,
26.3457,	164,
26.3652,	182,
26.3846,	180,
26.4041,	153,
26.4236,	161,
26.443,	187,
26.4625,	169,
26.4819,	192,
26.5014,	154,
26.5209,	161,
26.5403,	193,
26.5598,	179,
26.5792,	157,
26.5987,	161,
26.6181,	182,
26.6376,	166,
26.6571,	162,
26.6765,	157,
26.696,	194,
26.7154,	140,
26.7349,	159,
26.7544,	155,
26.7738,	161,
26.7933,	183,
26.8127,	147,
26.8322,	153,

26.8516, 177,

26.8711, 181,

26.8906, 172,

26.91, 147,

26.9295, 165,

26.9489, 143,

26.9684, 150,

26.9879, 209,

27.0073, 180,

27.0268, 153,

27.0462, 190,

27.0657, 170,

27.0851, 148,

27.1046, 173,

27.1241, 179,

27.1435, 161,

27.163, 169,

27.1824, 190,

27.2019, 179,

27.2214, 174,

27.2408, 159,

27.2603, 194,

27.2797, 171,

27.2992, 171,

27.3186, 175,

27.3381, 172,

27.3576, 175,

27.377,	172,
27.3965,	146,
27.4159,	164,
27.4354,	184,
27.4549,	151,
27.4743,	183,
27.4938,	182,
27.5132,	149,
27.5327,	179,
27.5521,	153,
27.5716,	146,
27.5911,	182,
27.6105,	154,
27.63,	169,
27.6494,	181,
27.6689,	169,
27.6884,	174,
27.7078,	182,
27.7273,	155,
27.7467,	177,
27.7662,	165,
27.7856,	174,
27.8051,	149,
27.8246,	191,
27.844,	168,
27.8635,	179,
27.8829,	166,

27.9024,	153,
27.9219,	165,
27.9413,	160,
27.9608,	182,
27.9802,	159,
27.9997,	152,
28.0191,	168,
28.0386,	173,
28.0581,	159,
28.0775,	167,
28.097,	153,
28.1164,	183,
28.1359,	184,
28.1553,	154,
28.1748,	175,
28.1943,	165,
28.2137,	169,
28.2332,	157,
28.2526,	179,
28.2721,	167,
28.2916,	168,
28.311,	151,
28.3305,	182,
28.3499,	175,
28.3694,	167,
28.3888,	170,
28.4083,	174,

28.4278,	165,
28.4472,	180,
28.4667,	182,
28.4861,	179,
28.5056,	157,
28.5251,	187,
28.5445,	178,
28.564,	161,
28.5834,	189,
28.6029,	164,
28.6223,	161,
28.6418,	187,
28.6613,	160,
28.6807,	153,
28.7002,	178,
28.7196,	158,
28.7391,	161,
28.7586,	174,
28.778,	181,
28.7975,	178,
28.8169,	189,
28.8364,	173,
28.8558,	171,
28.8753,	163,
28.8948,	168,
28.9142,	170,
28.9337,	153,

28.9531,	175,
28.9726,	166,
28.9921,	172,
29.0115,	171,
29.031,	165,
29.0504,	199,
29.0699,	163,
29.0893,	197,
29.1088,	151,
29.1283,	166,
29.1477,	182,
29.1672,	146,
29.1866,	170,
29.2061,	189,
29.2256,	161,
29.245,	168,
29.2645,	170,
29.2839,	175,
29.3034,	192,
29.3228,	181,
29.3423,	166,
29.3618,	195,
29.3812,	185,
29.4007,	177,
29.4201,	158,
29.4396,	179,
29.4591,	172,

29.4785,	167,
29.498,	174,
29.5174,	181,
29.5369,	195,
29.5563,	173,
29.5758,	182,
29.5953,	172,
29.6147,	174,
29.6342,	190,
29.6536,	163,
29.6731,	185,
29.6926,	169,
29.712,	165,
29.7315,	187,
29.7509,	172,
29.7704,	183,
29.7898,	171,
29.8093,	180,
29.8288,	184,
29.8482,	192,
29.8677,	183,
29.8871,	184,
29.9066,	174,
29.9261,	180,
29.9455,	169,
29.965,	173,
29.9844,	170,

30.0039,	193,
30.0233,	161,
30.0428,	162,
30.0623,	185,
30.0817,	181,
30.1012,	163,
30.1206,	185,
30.1401,	178,
30.1596,	151,
30.179,	202,
30.1985,	161,
30.2179,	146,
30.2374,	153,
30.2568,	177,
30.2763,	163,
30.2958,	155,
30.3152,	177,
30.3347,	175,
30.3541,	189,
30.3736,	162,
30.3931,	180,
30.4125,	174,
30.432,	182,
30.4514,	174,
30.4709,	167,
30.4903,	159,
30.5098,	160,

30.5293,	208,
30.5487,	187,
30.5682,	187,
30.5876,	178,
30.6071,	170,
30.6266,	203,
30.646,	166,
30.6655,	171,
30.6849,	179,
30.7044,	183,
30.7238,	167,
30.7433,	169,
30.7628,	165,
30.7822,	165,
30.8017,	166,
30.8211,	182,
30.8406,	198,
30.8601,	172,
30.8795,	169,
30.899,	177,
30.9184,	161,
30.9379,	162,
30.9573,	182,
30.9768,	183,
30.9963,	179,
31.0157,	176,
31.0352,	156,

31.0546,	184,
31.0741,	170,
31.0935,	181,
31.113,	165,
31.1325,	165,
31.1519,	168,
31.1714,	177,
31.1908,	196,
31.2103,	171,
31.2298,	164,
31.2492,	174,
31.2687,	204,
31.2881,	162,
31.3076,	179,
31.327,	176,
31.3465,	201,
31.366,	181,
31.3854,	196,
31.4049,	194,
31.4243,	184,
31.4438,	158,
31.4633,	191,
31.4827,	177,
31.5022,	190,
31.5216,	185,
31.5411,	190,
31.5605,	200,

31.58,	197,
31.5995,	204,
31.6189,	154,
31.6384,	187,
31.6578,	170,
31.6773,	201,
31.6968,	197,
31.7162,	177,
31.7357,	159,
31.7551,	175,
31.7746,	152,
31.794,	178,
31.8135,	154,
31.833,	179,
31.8524,	175,
31.8719,	190,
31.8913,	164,
31.9108,	180,
31.9303,	182,
31.9497,	176,
31.9692,	180,
31.9886,	185,
32.0081,	185,
32.0275,	198,
32.047,	171,
32.0665,	206,
32.0859,	180,

32.1054, 164,

32.1248, 169,

32.1443, 180,

32.1638, 189,

32.1832, 181,

32.2027, 151,

32.2221, 196,

32.2416, 200,

32.261, 173,

32.2805, 164,

32.3, 181,

32.3194, 190,

32.3389, 177,

32.3583, 178,

32.3778, 176,

32.3973, 186,

32.4167, 150,

32.4362, 154,

32.4556, 167,

32.4751, 181,

32.4945, 180,

32.514, 180,

32.5335, 202,

32.5529, 180,

32.5724, 178,

32.5918, 169,

32.6113, 148,

32.6308,	197,
32.6502,	183,
32.6697,	177,
32.6891,	176,
32.7086,	162,
32.728,	160,
32.7475,	204,
32.767,	181,
32.7864,	182,
32.8059,	174,
32.8253,	176,
32.8448,	194,
32.8643,	167,
32.8837,	181,
32.9032,	176,
32.9226,	175,
32.9421,	208,
32.9615,	195,
32.981,	191,
33.0005,	167,
33.0199,	185,
33.0394,	193,
33.0588,	194,
33.0783,	164,
33.0978,	152,
33.1172,	146,
33.1367,	163,

33.1561,	160,
33.1756,	212,
33.195,	172,
33.2145,	160,
33.234,	169,
33.2534,	149,
33.2729,	191,
33.2923,	189,
33.3118,	195,
33.3313,	192,
33.3507,	171,
33.3702,	173,
33.3896,	184,
33.4091,	177,
33.4285,	165,
33.448,	153,
33.4675,	177,
33.4869,	161,
33.5064,	184,
33.5258,	183,
33.5453,	174,
33.5648,	180,
33.5842,	193,
33.6037,	211,
33.6231,	190,
33.6426,	188,
33.662,	204,

33.6815,	198,
33.701,	175,
33.7204,	178,
33.7399,	168,
33.7593,	185,
33.7788,	182,
33.7983,	159,
33.8177,	175,
33.8372,	208,
33.8566,	201,
33.8761,	174,
33.8955,	161,
33.915,	160,
33.9345,	183,
33.9539,	160,
33.9734,	196,
33.9928,	187,
34.0123,	191,
34.0317,	169,
34.0512,	198,
34.0707,	188,
34.0901,	198,
34.1096,	191,
34.129,	199,
34.1485,	170,
34.168,	201,
34.1874,	159,

34.2069,	172,
34.2263,	178,
34.2458,	191,
34.2652,	211,
34.2847,	199,
34.3042,	182,
34.3236,	164,
34.3431,	177,
34.3625,	183,
34.382,	170,
34.4015,	202,
34.4209,	203,
34.4404,	182,
34.4598,	182,
34.4793,	170,
34.4987,	172,
34.5182,	184,
34.5377,	154,
34.5571,	180,
34.5766,	189,
34.596,	169,
34.6155,	182,
34.635,	186,
34.6544,	180,
34.6739,	185,
34.6933,	184,
34.7128,	201,

34.7322,	187,
34.7517,	175,
34.7712,	187,
34.7906,	178,
34.8101,	178,
34.8295,	201,
34.849,	190,
34.8685,	197,
34.8879,	177,
34.9074,	184,
34.9268,	213,
34.9463,	178,
34.9657,	183,
34.9852,	211,
35.0047,	201,
35.0241,	195,
35.0436,	190,
35.063,	203,
35.0825,	179,
35.102,	188,
35.1214,	192,
35.1409,	190,
35.1603,	185,
35.1798,	204,
35.1992,	191,
35.2187,	198,
35.2382,	186,

35.2576,	208,
35.2771,	199,
35.2965,	211,
35.316,	184,
35.3355,	193,
35.3549,	208,
35.3744,	196,
35.3938,	209,
35.4133,	214,
35.4327,	222,
35.4522,	197,
35.4717,	193,
35.4911,	194,
35.5106,	225,
35.53,	189,
35.5495,	209,
35.569,	202,
35.5884,	181,
35.6079,	206,
35.6273,	220,
35.6468,	200,
35.6662,	204,
35.6857,	182,
35.7052,	192,
35.7246,	197,
35.7441,	200,
35.7635,	166,

35.783, 178,

35.8025, 189,

35.8219, 179,

35.8414, 199,

35.8608, 194,

35.8803, 195,

35.8997, 178,

35.9192, 214,

35.9387, 187,

35.9581, 219,

35.9776, 193,

35.997, 212,

36.0165, 174,

36.036, 192,

36.0554, 159,

36.0749, 187,

36.0943, 209,

36.1138, 215,

36.1332, 161,

36.1527, 175,

36.1722, 173,

36.1916, 174,

36.2111, 186,

36.2305, 185,

36.25, 191,

36.2695, 193,

36.2889, 191,

36.3084,	163,
36.3278,	188,
36.3473,	170,
36.3667,	182,
36.3862,	183,
36.4057,	182,
36.4251,	165,
36.4446,	174,
36.464,	175,
36.4835,	181,
36.503,	183,
36.5224,	194,
36.5419,	196,
36.5613,	204,
36.5808,	187,
36.6002,	173,
36.6197,	201,
36.6392,	161,
36.6586,	196,
36.6781,	181,
36.6975,	187,
36.717,	184,
36.7365,	190,
36.7559,	188,
36.7754,	174,
36.7948,	165,
36.8143,	180,

36.8337,	169,
36.8532,	202,
36.8727,	183,
36.8921,	193,
36.9116,	202,
36.931,	198,
36.9505,	191,
36.9699,	197,
36.9894,	179,
37.0089,	184,
37.0283,	184,
37.0478,	174,
37.0672,	179,
37.0867,	159,
37.1062,	186,
37.1256,	193,
37.1451,	177,
37.1645,	168,
37.184,	199,
37.2034,	186,
37.2229,	198,
37.2424,	202,
37.2618,	172,
37.2813,	184,
37.3007,	162,
37.3202,	190,
37.3397,	179,

37.3591, 170,

37.3786, 182,

37.398, 181,

37.4175, 178,

37.4369, 168,

37.4564, 159,

37.4759, 154,

37.4953, 196,

37.5148, 179,

37.5342, 207,

37.5537, 180,

37.5732, 172,

37.5926, 175,

37.6121, 181,

37.6315, 198,

37.651, 182,

37.6704, 178,

37.6899, 190,

37.7094, 176,

37.7288, 210,

37.7483, 183,

37.7677, 174,

37.7872, 175,

37.8067, 143,

37.8261, 155,

37.8456, 207,

37.865, 181,

37.8845,	172,
37.9039,	179,
37.9234,	205,
37.9429,	200,
37.9623,	174,
37.9818,	196,
38.0012,	204,
38.0207,	180,
38.0402,	169,
38.0596,	184,
38.0791,	187,
38.0985,	161,
38.118,	180,
38.1374,	172,
38.1569,	181,
38.1764,	209,
38.1958,	185,
38.2153,	185,
38.2347,	179,
38.2542,	188,
38.2737,	195,
38.2931,	167,
38.3126,	185,
38.332,	191,
38.3515,	176,
38.3709,	191,
38.3904,	183,

38.4099, 167,

38.4293, 169,

38.4488, 187,

38.4682, 201,

38.4877, 206,

38.5072, 146,

38.5266, 197,

38.5461, 173,

38.5655, 174,

38.585, 181,

38.6044, 175,

38.6239, 186,

38.6434, 188,

38.6628, 193,

38.6823, 165,

38.7017, 195,

38.7212, 194,

38.7407, 191,

38.7601, 198,

38.7796, 181,

38.799, 183,

38.8185, 198,

38.8379, 182,

38.8574, 194,

38.8769, 185,

38.8963, 195,

38.9158, 202,

38.9352, 160,

38.9547, 178,

38.9742, 174,

38.9936, 199,

39.0131, 180,

39.0325, 184,

39.052, 176,

39.0714, 180,

39.0909, 177,

39.1104, 182,

39.1298, 201,

39.1493, 183,

39.1687, 193,

39.1882, 164,

39.2077, 169,

39.2271, 168,

39.2466, 182,

39.266, 179,

39.2855, 203,

39.3049, 187,

39.3244, 164,

39.3439, 161,

39.3633, 205,

39.3828, 189,

39.4022, 185,

39.4217, 191,

39.4412, 180,

39.4606,	178,
39.4801,	178,
39.4995,	154,
39.519,	184,
39.5384,	182,
39.5579,	195,
39.5774,	182,
39.5968,	182,
39.6163,	183,
39.6357,	196,
39.6552,	180,
39.6746,	177,
39.6941,	181,
39.7136,	189,
39.733,	182,
39.7525,	195,
39.7719,	203,
39.7914,	189,
39.8109,	182,
39.8303,	178,
39.8498,	196,
39.8692,	181,
39.8887,	183,
39.9081,	187,
39.9276,	190,
39.9471,	174,
39.9665,	170,

39.986, 188,

40.0054, 195,

40.0249, 182,

40.0444, 178,

40.0638, 196,

40.0833, 190,

40.1027, 195,

40.1222, 204,

40.1416, 196,

40.1611, 180,

40.1806, 193,

40.2, 181,

40.2195, 198,

40.2389, 178,

40.2584, 172,

40.2779, 187,

40.2973, 188,

40.3168, 204,

40.3362, 206,

40.3557, 193,

40.3751, 187,

40.3946, 172,

40.4141, 186,

40.4335, 201,

40.453, 197,

40.4724, 184,

40.4919, 183,

40.5114, 184,

40.5308, 201,

40.5503, 158,

40.5697, 180,

40.5892, 197,

40.6086, 214,

40.6281, 202,

40.6476, 199,

40.667, 213,

40.6865, 183,

40.7059, 207,

40.7254, 189,

40.7449, 210,

40.7643, 192,

40.7838, 193,

40.8032, 205,

40.8227, 157,

40.8421, 189,

40.8616, 187,

40.8811, 205,

40.9005, 202,

40.92, 194,

40.9394, 196,

40.9589, 193,

40.9784, 183,

40.9978, 193,

41.0173, 178,

41.0367,	206,
41.0562,	209,
41.0756,	196,
41.0951,	168,
41.1146,	192,
41.134,	187,
41.1535,	179,
41.1729,	186,
41.1924,	179,
41.2119,	177,
41.2313,	179,
41.2508,	180,
41.2702,	188,
41.2897,	196,
41.3091,	204,
41.3286,	183,
41.3481,	187,
41.3675,	189,
41.387,	203,
41.4064,	183,
41.4259,	192,
41.4454,	205,
41.4648,	199,
41.4843,	191,
41.5037,	196,
41.5232,	182,
41.5426,	168,

41.5621,	191,
41.5816,	188,
41.601,	166,
41.6205,	212,
41.6399,	184,
41.6594,	176,
41.6789,	210,
41.6983,	203,
41.7178,	209,
41.7372,	196,
41.7567,	189,
41.7761,	191,
41.7956,	188,
41.8151,	168,
41.8345,	192,
41.854,	189,
41.8734,	181,
41.8929,	177,
41.9124,	190,
41.9318,	188,
41.9513,	172,
41.9707,	179,
41.9902,	173,
42.0096,	192,
42.0291,	182,
42.0486,	205,
42.068,	193,

42.0875, 169,

42.1069, 206,

42.1264, 184,

42.1459, 205,

42.1653, 183,

42.1848, 166,

42.2042, 165,

42.2237, 163,

42.2431, 171,

42.2626, 203,

42.2821, 190,

42.3015, 175,

42.321, 188,

42.3404, 173,

42.3599, 190,

42.3794, 169,

42.3988, 189,

42.4183, 191,

42.4377, 202,

42.4572, 165,

42.4766, 192,

42.4961, 217,

42.5156, 178,

42.535, 190,

42.5545, 189,

42.5739, 199,

42.5934, 156,

42.6128, 190,

42.6323, 200,

42.6518, 192,

42.6712, 191,

42.6907, 188,

42.7101, 180,

42.7296, 183,

42.7491, 192,

42.7685, 189,

42.788, 191,

42.8074, 193,

42.8269, 177,

42.8463, 204,

42.8658, 174,

42.8853, 184,

42.9047, 188,

42.9242, 201,

42.9436, 183,

42.9631, 179,

42.9826, 186,

43.002, 189,

43.0215, 192,

43.0409, 173,

43.0604, 183,

43.0798, 187,

43.0993, 181,

43.1188, 170,

43.1382,	174,
43.1577,	201,
43.1771,	194,
43.1966,	216,
43.2161,	182,
43.2355,	192,
43.255,	187,
43.2744,	179,
43.2939,	188,
43.3133,	200,
43.3328,	193,
43.3523,	190,
43.3717,	172,
43.3912,	172,
43.4106,	206,
43.4301,	205,
43.4496,	205,
43.469,	189,
43.4885,	180,
43.5079,	179,
43.5274,	178,
43.5468,	166,
43.5663,	173,
43.5858,	175,
43.6052,	199,
43.6247,	190,
43.6441,	204,

43.6636,	196,
43.6831,	192,
43.7025,	196,
43.722,	171,
43.7414,	172,
43.7609,	207,
43.7803,	176,
43.7998,	182,
43.8193,	201,
43.8387,	165,
43.8582,	179,
43.8776,	200,
43.8971,	197,
43.9166,	172,
43.936,	168,
43.9555,	199,
43.9749,	171,
43.9944,	171,
44.0138,	206,
44.0333,	195,
44.0528,	178,
44.0722,	178,
44.0917,	196,
44.1111,	189,
44.1306,	209,
44.1501,	174,
44.1695,	190,

44.189,	178,
44.2084,	180,
44.2279,	181,
44.2473,	192,
44.2668,	178,
44.2863,	173,
44.3057,	200,
44.3252,	182,
44.3446,	205,
44.3641,	200,
44.3836,	170,
44.403,	212,
44.4225,	186,
44.4419,	203,
44.4614,	175,
44.4808,	201,
44.5003,	186,
44.5198,	181,
44.5392,	205,
44.5587,	195,
44.5781,	184,
44.5976,	204,
44.6171,	181,
44.6365,	190,
44.656,	198,
44.6754,	195,
44.6949,	195,

44.7143,	185,
44.7338,	173,
44.7533,	190,
44.7727,	204,
44.7922,	198,
44.8116,	189,
44.8311,	166,
44.8506,	171,
44.87,	214,
44.8895,	186,
44.9089,	204,
44.9284,	172,
44.9478,	188,
44.9673,	176,
44.9868,	183,
45.0062,	187,
45.0257,	157,
45.0451,	193,
45.0646,	201,
45.0841,	200,
45.1035,	206,
45.123,	177,
45.1424,	191,
45.1619,	198,
45.1813,	160,
45.2008,	176,
45.2203,	181,

45.2397,	161,
45.2592,	239,
45.2786,	157,
45.2981,	200,
45.3176,	176,
45.337,	193,
45.3565,	182,
45.3759,	184,
45.3954,	204,
45.4148,	181,
45.4343,	196,
45.4538,	197,
45.4732,	195,
45.4927,	180,
45.5121,	200,
45.5316,	186,
45.551,	181,
45.5705,	185,
45.59,	186,
45.6094,	173,
45.6289,	188,
45.6483,	188,
45.6678,	217,
45.6873,	191,
45.7067,	196,
45.7262,	200,
45.7456,	184,

45.7651,	189,
45.7845,	206,
45.804,	171,
45.8235,	193,
45.8429,	182,
45.8624,	226,
45.8818,	196,
45.9013,	191,
45.9208,	183,
45.9402,	210,
45.9597,	171,
45.9791,	200,
45.9986,	185,
46.018,	200,
46.0375,	187,
46.057,	193,
46.0764,	159,
46.0959,	186,
46.1153,	189,
46.1348,	193,
46.1543,	184,
46.1737,	174,
46.1932,	180,
46.2126,	159,
46.2321,	195,
46.2515,	191,
46.271,	186,

46.2905, 217,

46.3099, 189,

46.3294, 185,

46.3488, 196,

46.3683, 175,

46.3878, 220,

46.4072, 196,

46.4267, 190,

46.4461, 220,

46.4656, 185,

46.485, 196,

46.5045, 162,

46.524, 200,

46.5434, 184,

46.5629, 177,

46.5823, 212,

46.6018, 188,

46.6213, 199,

46.6407, 166,

46.6602, 161,

46.6796, 183,

46.6991, 203,

46.7185, 184,

46.738, 216,

46.7575, 186,

46.7769, 192,

46.7964, 188,

46.8158,	186,
46.8353,	186,
46.8548,	161,
46.8742,	168,
46.8937,	215,
46.9131,	186,
46.9326,	191,
46.952,	206,
46.9715,	196,
46.991,	186,
47.0104,	181,
47.0299,	173,
47.0493,	181,
47.0688,	187,
47.0883,	194,
47.1077,	192,
47.1272,	176,
47.1466,	192,
47.1661,	180,
47.1855,	196,
47.205,	177,
47.2245,	203,
47.2439,	204,
47.2634,	183,
47.2828,	177,
47.3023,	179,
47.3218,	178,

47.3412,	197,
47.3607,	193,
47.3801,	225,
47.3996,	175,
47.419,	185,
47.4385,	192,
47.458,	191,
47.4774,	195,
47.4969,	210,
47.5163,	184,
47.5358,	182,
47.5553,	201,
47.5747,	177,
47.5942,	214,
47.6136,	191,
47.6331,	186,
47.6525,	187,
47.672,	196,
47.6915,	191,
47.7109,	186,
47.7304,	188,
47.7498,	182,
47.7693,	187,
47.7888,	179,
47.8082,	188,
47.8277,	207,
47.8471,	181,

47.8666,	190,
47.886,	192,
47.9055,	178,
47.925,	201,
47.9444,	213,
47.9639,	186,
47.9833,	183,
48.0028,	188,
48.0223,	190,
48.0417,	207,
48.0612,	193,
48.0806,	191,
48.1001,	165,
48.1195,	180,
48.139,	199,
48.1585,	204,
48.1779,	207,
48.1974,	182,
48.2168,	187,
48.2363,	187,
48.2558,	183,
48.2752,	188,
48.2947,	176,
48.3141,	177,
48.3336,	219,
48.353,	164,
48.3725,	194,

48.392,	223,
48.4114,	162,
48.4309,	180,
48.4503,	179,
48.4698,	180,
48.4892,	178,
48.5087,	179,
48.5282,	223,
48.5476,	221,
48.5671,	169,
48.5865,	182,
48.606,	172,
48.6255,	203,
48.6449,	188,
48.6644,	198,
48.6838,	178,
48.7033,	183,
48.7227,	180,
48.7422,	202,
48.7617,	177,
48.7811,	179,
48.8006,	188,
48.82,	195,
48.8395,	219,
48.859,	213,
48.8784,	205,
48.8979,	200,

48.9173, 197,

48.9368, 189,

48.9562, 191,

48.9757, 184,

48.9952, 201,

49.0146, 201,

49.0341, 200,

49.0535, 199,

49.073, 205,

49.0925, 211,

49.1119, 197,

49.1314, 157,

49.1508, 201,

49.1703, 191,

49.1897, 212,

49.2092, 183,

49.2287, 163,

49.2481, 177,

49.2676, 175,

49.287, 204,

49.3065, 186,

49.326, 171,

49.3454, 178,

49.3649, 207,

49.3843, 180,

49.4038, 205,

49.4232, 177,

49.4427, 176,

49.4622, 196,

49.4816, 194,

49.5011, 169,

49.5205, 199,

49.54, 209,

49.5595, 193,

49.5789, 204,

49.5984, 204,

49.6178, 186,

49.6373, 208,

49.6567, 183,

49.6762, 196,

49.6957, 202,

49.7151, 208,

49.7346, 195,

49.754, 200,

49.7735, 177,

49.793, 215,

49.8124, 175,

49.8319, 162,

49.8513, 214,

49.8708, 207,

49.8902, 200,

49.9097, 169,

49.9292, 191,

49.9486, 206,

49.9681,	167,
49.9875,	186,
50.007,	195,
50.0265,	198,
50.0459,	207,
50.0654,	215,
50.0848,	198,
50.1043,	184,
50.1237,	189,
50.1432,	197,
50.1627,	205,
50.1821,	195,
50.2016,	166,
50.221,	214,
50.2405,	175,
50.26,	188,
50.2794,	197,
50.2989,	181,
50.3183,	197,
50.3378,	204,
50.3572,	189,
50.3767,	198,
50.3962,	204,
50.4156,	182,
50.4351,	169,
50.4545,	184,
50.474,	178,

50.4935,	194,
50.5129,	195,
50.5324,	192,
50.5518,	186,
50.5713,	189,
50.5907,	192,
50.6102,	172,
50.6297,	185,
50.6491,	180,
50.6686,	190,
50.688,	178,
50.7075,	166,
50.727,	193,
50.7464,	179,
50.7659,	179,
50.7853,	179,
50.8048,	184,
50.8242,	166,
50.8437,	215,
50.8632,	173,
50.8826,	200,
50.9021,	173,
50.9215,	179,
50.941,	175,
50.9605,	198,
50.9799,	190,
50.9994,	197,

51.0188, 212,

51.0383, 195,

51.0577, 219,

51.0772, 185,

51.0967, 174,

51.1161, 212,

51.1356, 215,

51.155, 203,

51.1745, 188,

51.1939, 193,

51.2134, 176,

51.2329, 181,

51.2523, 172,

51.2718, 188,

51.2912, 192,

51.3107, 202,

51.3302, 215,

51.3496, 194,

51.3691, 196,

51.3885, 184,

51.408, 179,

51.4274, 218,

51.4469, 191,

51.4664, 202,

51.4858, 169,

51.5053, 200,

51.5247, 207,

51.5442,	173,
51.5637,	209,
51.5831,	185,
51.6026,	188,
51.622,	206,
51.6415,	173,
51.6609,	191,
51.6804,	187,
51.6999,	201,
51.7193,	197,
51.7388,	220,
51.7582,	184,
51.7777,	203,
51.7972,	206,
51.8166,	180,
51.8361,	182,
51.8555,	193,
51.875,	211,
51.8944,	213,
51.9139,	187,
51.9334,	194,
51.9528,	164,
51.9723,	183,
51.9917,	192,
52.0112,	198,
52.0307,	191,
52.0501,	183,

52.0696,	179,
52.089,	201,
52.1085,	191,
52.1279,	180,
52.1474,	197,
52.1669,	188,
52.1863,	190,
52.2058,	210,
52.2252,	186,
52.2447,	198,
52.2642,	191,
52.2836,	201,
52.3031,	178,
52.3225,	204,
52.342,	198,
52.3614,	186,
52.3809,	189,
52.4004,	203,
52.4198,	223,
52.4393,	190,
52.4587,	202,
52.4782,	179,
52.4977,	187,
52.5171,	200,
52.5366,	203,
52.556,	209,
52.5755,	188,

52.5949, 224,

52.6144, 176,

52.6339, 193,

52.6533, 201,

52.6728, 211,

52.6922, 198,

52.7117, 208,

52.7312, 201,

52.7506, 194,

52.7701, 181,

52.7895, 182,

52.809, 187,

52.8284, 208,

52.8479, 175,

52.8674, 193,

52.8868, 191,

52.9063, 192,

52.9257, 198,

52.9452, 197,

52.9647, 189,

52.9841, 209,

53.0036, 201,

53.023, 186,

53.0425, 190,

53.0619, 192,

53.0814, 214,

53.1009, 197,

53.1203, 170,

53.1398, 213,

53.1592, 189,

53.1787, 200,

53.1982, 165,

53.2176, 195,

53.2371, 202,

53.2565, 201,

53.276, 209,

53.2954, 196,

53.3149, 206,

53.3344, 184,

53.3538, 204,

53.3733, 187,

53.3927, 194,

53.4122, 181,

53.4317, 184,

53.4511, 201,

53.4706, 222,

53.49, 228,

53.5095, 241,

53.5289, 168,

53.5484, 193,

53.5679, 181,

53.5873, 183,

53.6068, 208,

53.6262, 182,

53.6457,	213,
53.6652,	211,
53.6846,	189,
53.7041,	229,
53.7235,	201,
53.743,	210,
53.7624,	184,
53.7819,	199,
53.8014,	208,
53.8208,	186,
53.8403,	195,
53.8597,	189,
53.8792,	185,
53.8987,	171,
53.9181,	200,
53.9376,	182,
53.957,	213,
53.9765,	206,
53.9959,	204,
54.0154,	191,
54.0349,	205,
54.0543,	203,
54.0738,	205,
54.0932,	200,
54.1127,	206,
54.1321,	182,
54.1516,	193,

54.1711,	234,
54.1905,	183,
54.21,	178,
54.2294,	199,
54.2489,	177,
54.2684,	204,
54.2878,	191,
54.3073,	205,
54.3267,	210,
54.3462,	191,
54.3656,	198,
54.3851,	171,
54.4046,	210,
54.424,	206,
54.4435,	196,
54.4629,	192,
54.4824,	190,
54.5019,	214,
54.5213,	216,
54.5408,	221,
54.5602,	185,
54.5797,	205,
54.5991,	207,
54.6186,	193,
54.6381,	209,
54.6575,	212,
54.677,	203,

54.6964,	204,
54.7159,	188,
54.7354,	192,
54.7548,	196,
54.7743,	204,
54.7937,	187,
54.8132,	159,
54.8326,	182,
54.8521,	192,
54.8716,	178,
54.891,	214,
54.9105,	181,
54.9299,	227,
54.9494,	181,
54.9689,	190,
54.9883,	212,
55.0078,	194,
55.0272,	194,
55.0467,	201,
55.0661,	200,
55.0856,	219,
55.1051,	192,
55.1245,	198,
55.144,	221,
55.1634,	204,
55.1829,	172,
55.2024,	231,

55.2218, 192,

55.2413, 211,

55.2607, 198,

55.2802, 188,

55.2996, 183,

55.3191, 198,

55.3386, 182,

55.358, 227,

55.3775, 204,

55.3969, 187,

55.4164, 168,

55.4359, 226,

55.4553, 206,

55.4748, 197,

55.4942, 191,

55.5137, 161,

55.5331, 210,

55.5526, 180,

55.5721, 188,

55.5915, 196,

55.611, 193,

55.6304, 201,

55.6499, 199,

55.6694, 186,

55.6888, 196,

55.7083, 193,

55.7277, 207,

55.7472,	206,
55.7666,	181,
55.7861,	185,
55.8056,	198,
55.825,	195,
55.8445,	208,
55.8639,	199,
55.8834,	184,
55.9029,	180,
55.9223,	183,
55.9418,	214,
55.9612,	201,
55.9807,	200,
56.0001,	194,
56.0196,	163,
56.0391,	201,
56.0585,	189,
56.078,	179,
56.0974,	200,
56.1169,	207,
56.1364,	195,
56.1558,	186,
56.1753,	206,
56.1947,	204,
56.2142,	212,
56.2336,	189,
56.2531,	218,

56.2726,	199,
56.292,	172,
56.3115,	201,
56.3309,	190,
56.3504,	188,
56.3699,	184,
56.3893,	217,
56.4088,	199,
56.4282,	191,
56.4477,	212,
56.4671,	191,
56.4866,	170,
56.5061,	218,
56.5255,	202,
56.545,	177,
56.5644,	209,
56.5839,	200,
56.6034,	178,
56.6228,	214,
56.6423,	182,
56.6617,	213,
56.6812,	192,
56.7006,	199,
56.7201,	180,
56.7396,	190,
56.759,	170,
56.7785,	187,

56.7979, 190,

56.8174, 185,

56.8369, 179,

56.8563, 195,

56.8758, 199,

56.8952, 214,

56.9147, 194,

56.9341, 192,

56.9536, 189,

56.9731, 193,

56.9925, 192,

57.012, 191,

57.0314, 199,

57.0509, 211,

57.0703, 199,

57.0898, 201,

57.1093, 187,

57.1287, 207,

57.1482, 197,

57.1676, 205,

57.1871, 177,

57.2066, 199,

57.226, 209,

57.2455, 213,

57.2649, 191,

57.2844, 221,

57.3038, 180,

57.3233,	183,
57.3428,	192,
57.3622,	218,
57.3817,	196,
57.4011,	191,
57.4206,	173,
57.4401,	187,
57.4595,	182,
57.479,	219,
57.4984,	214,
57.5179,	237,
57.5373,	206,
57.5568,	181,
57.5763,	215,
57.5957,	219,
57.6152,	185,
57.6346,	191,
57.6541,	197,
57.6736,	230,
57.693,	188,
57.7125,	214,
57.7319,	214,
57.7514,	204,
57.7708,	173,
57.7903,	203,
57.8098,	200,
57.8292,	170,

57.8487, 210,

57.8681, 186,

57.8876, 185,

57.9071, 215,

57.9265, 209,

57.946, 202,

57.9654, 187,

57.9849, 192,

58.0043, 206,

58.0238, 185,

58.0433, 202,

58.0627, 206,

58.0822, 219,

58.1016, 184,

58.1211, 227,

58.1406, 189,

58.16, 176,

58.1795, 172,

58.1989, 200,

58.2184, 180,

58.2378, 209,

58.2573, 190,

58.2768, 187,

58.2962, 185,

58.3157, 189,

58.3351, 181,

58.3546, 208,

58.3741,	191,
58.3935,	215,
58.413,	200,
58.4324,	208,
58.4519,	167,
58.4713,	201,
58.4908,	220,
58.5103,	173,
58.5297,	202,
58.5492,	183,
58.5686,	185,
58.5881,	182,
58.6076,	222,
58.627,	196,
58.6465,	214,
58.6659,	186,
58.6854,	185,
58.7048,	180,
58.7243,	176,
58.7438,	205,
58.7632,	203,
58.7827,	235,
58.8021,	175,
58.8216,	187,
58.8411,	195,
58.8605,	185,
58.88,	184,

58.8994,	208,
58.9189,	197,
58.9383,	175,
58.9578,	201,
58.9773,	213,
58.9967,	192,
59.0162,	177,
59.0356,	170,
59.0551,	178,
59.0746,	176,
59.094,	175,
59.1135,	211,
59.1329,	194,
59.1524,	209,
59.1718,	198,
59.1913,	190,
59.2108,	206,
59.2302,	212,
59.2497,	191,
59.2691,	190,
59.2886,	197,
59.3081,	205,
59.3275,	205,
59.347,	199,
59.3664,	182,
59.3859,	195,
59.4053,	185,

59.4248,	193,
59.4443,	173,
59.4637,	191,
59.4832,	167,
59.5026,	197,
59.5221,	188,
59.5416,	180,
59.561,	196,
59.5805,	195,
59.5999,	201,
59.6194,	186,
59.6388,	182,
59.6583,	213,
59.6778,	190,
59.6972,	226,
59.7167,	209,
59.7361,	195,
59.7556,	196,
59.7751,	222,
59.7945,	193,
59.814,	210,
59.8334,	199,
59.8529,	217,
59.8723,	193,
59.8918,	197,
59.9113,	191,
59.9307,	189,

59.9502, 224,

59.9696, 231,

59.9891, 192,

60.0085, 189,

60.028, 175,

60.0475, 195,

60.0669, 190,

60.0864, 213,

60.1058, 215,

60.1253, 215,

60.1448, 212,

60.1642, 179,

60.1837, 213,

60.2031, 202,

60.2226, 181,

60.242, 189,

60.2615, 196,

60.281, 204,

60.3004, 180,

60.3199, 190,

60.3393, 210,

60.3588, 176,

60.3783, 199,

60.3977, 193,

60.4172, 189,

60.4366, 212,

60.4561, 214,

60.4755,	215,
60.495,	214,
60.5145,	222,
60.5339,	201,
60.5534,	186,
60.5728,	177,
60.5923,	193,
60.6118,	180,
60.6312,	185,
60.6507,	213,
60.6701,	199,
60.6896,	218,
60.709,	204,
60.7285,	181,
60.748,	198,
60.7674,	197,
60.7869,	229,
60.8063,	204,
60.8258,	184,
60.8453,	210,
60.8647,	198,
60.8842,	208,
60.9036,	212,
60.9231,	198,
60.9425,	190,
60.962,	183,
60.9815,	218,

61.0009, 194,

61.0204, 203,

61.0398, 201,

61.0593, 209,

61.0788, 195,

61.0982, 213,

61.1177, 205,

61.1371, 213,

61.1566, 184,

61.176, 198,

61.1955, 180,

61.215, 196,

61.2344, 181,

61.2539, 197,

61.2733, 193,

61.2928, 177,

61.3123, 204,

61.3317, 186,

61.3512, 180,

61.3706, 202,

61.3901, 193,

61.4095, 204,

61.429, 210,

61.4485, 184,

61.4679, 179,

61.4874, 188,

61.5068, 198,

61.5263, 214,

61.5458, 218,

61.5652, 191,

61.5847, 209,

61.6041, 213,

61.6236, 201,

61.643, 199,

61.6625, 190,

61.682, 197,

61.7014, 194,

61.7209, 188,

61.7403, 196,

61.7598, 224,

61.7793, 218,

61.7987, 194,

61.8182, 201,

61.8376, 207,

61.8571, 198,

61.8765, 195,

61.896, 207,

61.9155, 223,

61.9349, 173,

61.9544, 200,

61.9738, 216,

61.9933, 201,

62.0128, 213,

62.0322, 201,

62.0517,	201,
62.0711,	176,
62.0906,	194,
62.11,	203,
62.1295,	199,
62.149,	206,
62.1684,	222,
62.1879,	207,
62.2073,	188,
62.2268,	187,
62.2463,	191,
62.2657,	190,
62.2852,	210,
62.3046,	187,
62.3241,	185,
62.3435,	229,
62.363,	194,
62.3825,	193,
62.4019,	197,
62.4214,	206,
62.4408,	211,
62.4603,	203,
62.4798,	191,
62.4992,	216,
62.5187,	216,
62.5381,	220,
62.5576,	209,

62.577, 185,

62.5965, 204,

62.616, 188,

62.6354, 197,

62.6549, 208,

62.6743, 180,

62.6938, 190,

62.7132, 212,

62.7327, 212,

62.7522, 208,

62.7716, 190,

62.7911, 197,

62.8105, 157,

62.83, 188,

62.8495, 211,

62.8689, 197,

62.8884, 223,

62.9078, 212,

62.9273, 206,

62.9467, 219,

62.9662, 198,

62.9857, 184,

63.0051, 187,

63.0246, 196,

63.044, 186,

63.0635, 194,

63.083, 201,

63.1024, 211,

63.1219, 203,

63.1413, 193,

63.1608, 204,

63.1802, 211,

63.1997, 236,

63.2192, 198,

63.2386, 207,

63.2581, 191,

63.2775, 212,

63.297, 226,

63.3165, 193,

63.3359, 211,

63.3554, 186,

63.3748, 196,

63.3943, 216,

63.4137, 183,

63.4332, 189,

63.4527, 183,

63.4721, 218,

63.4916, 215,

63.511, 189,

63.5305, 191,

63.55, 176,

63.5694, 207,

63.5889, 221,

63.6083, 185,

63.6278, 201,

63.6472, 171,

63.6667, 198,

63.6862, 186,

63.7056, 201,

63.7251, 199,

63.7445, 197,

63.764, 198,

63.7835, 203,

63.8029, 183,

63.8224, 211,

63.8418, 213,

63.8613, 197,

63.8807, 199,

63.9002, 210,

63.9197, 174,

63.9391, 210,

63.9586, 192,

63.978, 203,

63.9975, 207,

64.017, 204,

64.0364, 220,

64.0559, 204,

64.0753, 215,

64.0948, 167,

64.1142, 181,

64.1337, 188,

64.1532,	206,
64.1726,	231,
64.1921,	187,
64.2115,	177,
64.231,	200,
64.2505,	193,
64.2699,	223,
64.2894,	196,
64.3088,	195,
64.3283,	208,
64.3477,	189,
64.3672,	199,
64.3867,	205,
64.4061,	222,
64.4256,	197,
64.445,	191,
64.4645,	214,
64.484,	185,
64.5034,	175,
64.5229,	197,
64.5423,	185,
64.5618,	182,
64.5812,	210,
64.6007,	188,
64.6202,	192,
64.6396,	217,
64.6591,	193,

64.6785,	228,
64.698,	209,
64.7175,	196,
64.7369,	184,
64.7564,	199,
64.7758,	203,
64.7953,	195,
64.8147,	182,
64.8342,	201,
64.8537,	188,
64.8731,	190,
64.8926,	186,
64.912,	188,
64.9315,	181,
64.951,	208,
64.9704,	199,
64.9899,	221,
65.0093,	185,
65.0288,	177,
65.0482,	214,
65.0677,	206,
65.0872,	176,
65.1066,	187,
65.1261,	194,
65.1455,	215,
65.165,	219,
65.1845,	196,

65.2039,	211,
65.2234,	173,
65.2428,	210,
65.2623,	191,
65.2817,	210,
65.3012,	192,
65.3207,	221,
65.3401,	227,
65.3596,	218,
65.379,	198,
65.3985,	191,
65.418,	178,
65.4374,	203,
65.4569,	226,
65.4763,	201,
65.4958,	185,
65.5152,	235,
65.5347,	212,
65.5542,	200,
65.5736,	198,
65.5931,	207,
65.6125,	206,
65.632,	185,
65.6514,	175,
65.6709,	216,
65.6904,	200,
65.7098,	221,

65.7293, 201,

65.7487, 193,

65.7682, 204,

65.7877, 205,

65.8071, 214,

65.8266, 194,

65.846, 203,

65.8655, 211,

65.8849, 199,

65.9044, 215,

65.9239, 194,

65.9433, 210,

65.9628, 176,

65.9822, 223,

66.0017, 177,

66.0212, 176,

66.0406, 207,

66.0601, 223,

66.0795, 166,

66.099, 202,

66.1184, 207,

66.1379, 190,

66.1574, 207,

66.1768, 177,

66.1963, 188,

66.2157, 181,

66.2352, 203,

66.2547, 210,

66.2741, 209,

66.2936, 209,

66.313, 208,

66.3325, 203,

66.3519, 205,

66.3714, 181,

66.3909, 182,

66.4103, 210,

66.4298, 204,

66.4492, 195,

66.4687, 185,

66.4882, 223,

66.5076, 170,

66.5271, 208,

66.5465, 203,

66.566, 214,

66.5854, 209,

66.6049, 196,

66.6244, 211,

66.6438, 192,

66.6633, 181,

66.6827, 197,

66.7022, 186,

66.7217, 212,

66.7411, 223,

66.7606, 177,

66.78, 190,
66.7995, 207,
66.8189, 202,
66.8384, 200,
66.8579, 196,
66.8773, 205,
66.8968, 186,
66.9162, 190,
66.9357, 187,
66.9552, 213,
66.9746, 211,
66.9941, 189,
67.0135, 216,
67.033, 208,
67.0524, 197,
67.0719, 186,
67.0914, 194,
67.1108, 207,
67.1303, 206,
67.1497, 200,
67.1692, 215,
67.1887, 208,
67.2081, 187,
67.2276, 225,
67.247, 192,
67.2665, 180,
67.2859, 219,

67.3054,	193,
67.3249,	214,
67.3443,	192,
67.3638,	198,
67.3832,	190,
67.4027,	204,
67.4222,	198,
67.4416,	174,
67.4611,	209,
67.4805,	215,
67.5,	176,
67.5194,	200,
67.5389,	192,
67.5584,	194,
67.5778,	195,
67.5973,	193,
67.6167,	173,
67.6362,	202,
67.6557,	219,
67.6751,	218,
67.6946,	186,
67.714,	214,
67.7335,	218,
67.7529,	206,
67.7724,	218,
67.7919,	190,
67.8113,	198,

67.8308,	225,
67.8502,	165,
67.8697,	194,
67.8892,	192,
67.9086,	217,
67.9281,	197,
67.9475,	210,
67.967,	204,
67.9864,	201,
68.0059,	194,
68.0254,	188,
68.0448,	195,
68.0643,	201,
68.0837,	218,
68.1032,	196,
68.1227,	224,
68.1421,	199,
68.1616,	193,
68.181,	226,
68.2005,	202,
68.2199,	205,
68.2394,	187,
68.2589,	218,
68.2783,	201,
68.2978,	217,
68.3172,	191,
68.3367,	207,

68.3562,	194,
68.3756,	166,
68.3951,	179,
68.4145,	208,
68.434,	185,
68.4534,	190,
68.4729,	199,
68.4924,	201,
68.5118,	195,
68.5313,	202,
68.5507,	195,
68.5702,	223,
68.5896,	171,
68.6091,	198,
68.6286,	168,
68.648,	206,
68.6675,	210,
68.6869,	198,
68.7064,	214,
68.7259,	208,
68.7453,	201,
68.7648,	201,
68.7842,	201,
68.8037,	209,
68.8231,	180,
68.8426,	189,
68.8621,	212,

68.8815,	196,
68.901,	201,
68.9204,	215,
68.9399,	227,
68.9594,	193,
68.9788,	214,
68.9983,	184,
69.0177,	194,
69.0372,	202,
69.0566,	211,
69.0761,	205,
69.0956,	188,
69.115,	192,
69.1345,	224,
69.1539,	213,
69.1734,	214,
69.1929,	182,
69.2123,	188,
69.2318,	214,
69.2512,	220,
69.2707,	195,
69.2901,	188,
69.3096,	221,
69.3291,	190,
69.3485,	180,
69.368,	180,
69.3874,	184,

69.4069,	195,
69.4264,	191,
69.4458,	183,
69.4653,	192,
69.4847,	196,
69.5042,	192,
69.5236,	189,
69.5431,	195,
69.5626,	209,
69.582,	188,
69.6015,	195,
69.6209,	195,
69.6404,	204,
69.6599,	183,
69.6793,	201,
69.6988,	207,
69.7182,	212,
69.7377,	193,
69.7571,	197,
69.7766,	195,
69.7961,	209,
69.8155,	192,
69.835,	187,
69.8544,	188,
69.8739,	203,
69.8934,	186,
69.9128,	185,

69.9323,	175,
69.9517,	191,
69.9712,	206,
69.9906,	195,
70.0101,	197,
70.0296,	215,
70.049,	212,
70.0685,	214,
70.0879,	196,
70.1074,	198,
70.1269,	192,
70.1463,	189,
70.1658,	216,
70.1852,	222,
70.2047,	184,
70.2241,	223,
70.2436,	177,
70.2631,	179,
70.2825,	202,
70.302,	189,
70.3214,	203,
70.3409,	214,
70.3604,	245,
70.3798,	202,
70.3993,	183,
70.4187,	177,
70.4382,	201,

70.4576,	208,
70.4771,	182,
70.4966,	222,
70.516,	209,
70.5355,	198,
70.5549,	217,
70.5744,	207,
70.5939,	191,
70.6133,	196,
70.6328,	188,
70.6522,	216,
70.6717,	186,
70.6911,	200,
70.7106,	186,
70.7301,	197,
70.7495,	202,
70.769,	179,
70.7884,	217,
70.8079,	196,
70.8274,	210,
70.8468,	218,
70.8663,	200,
70.8857,	190,
70.9052,	230,
70.9246,	210,
70.9441,	190,
70.9636,	204,

70.983,	199,
71.0025,	197,
71.0219,	210,
71.0414,	222,
71.0609,	202,
71.0803,	197,
71.0998,	204,
71.1192,	189,
71.1387,	186,
71.1581,	198,
71.1776,	221,
71.1971,	199,
71.2165,	202,
71.236,	192,
71.2554,	194,
71.2749,	179,
71.2944,	202,
71.3138,	191,
71.3333,	203,
71.3527,	188,
71.3722,	202,
71.3916,	198,
71.4111,	202,
71.4306,	200,
71.45,	194,
71.4695,	190,
71.4889,	205,

71.5084, 203,

71.5278, 204,

71.5473, 195,

71.5668, 198,

71.5862, 205,

71.6057, 196,

71.6251, 172,

71.6446, 204,

71.6641, 184,

71.6835, 221,

71.703, 211,

71.7224, 204,

71.7419, 208,

71.7613, 208,

71.7808, 202,

71.8003, 217,

71.8197, 200,

71.8392, 208,

71.8586, 199,

71.8781, 190,

71.8976, 201,

71.917, 209,

71.9365, 201,

71.9559, 180,

71.9754, 214,

71.9948, 181,

72.0143, 245,

72.0338,	220,
72.0532,	196,
72.0727,	187,
72.0921,	194,
72.1116,	187,
72.1311,	185,
72.1505,	200,
72.17,	217,
72.1894,	188,
72.2089,	188,
72.2283,	193,
72.2478,	189,
72.2673,	175,
72.2867,	185,
72.3062,	215,
72.3256,	213,
72.3451,	218,
72.3646,	198,
72.384,	187,
72.4035,	197,
72.4229,	195,
72.4424,	212,
72.4618,	194,
72.4813,	194,
72.5008,	203,
72.5202,	171,
72.5397,	202,

72.5591,	214,
72.5786,	202,
72.5981,	201,
72.6175,	197,
72.637,	196,
72.6564,	214,
72.6759,	216,
72.6953,	220,
72.7148,	173,
72.7343,	214,
72.7537,	193,
72.7732,	209,
72.7926,	188,
72.8121,	177,
72.8316,	226,
72.851,	246,
72.8705,	218,
72.8899,	191,
72.9094,	217,
72.9288,	188,
72.9483,	187,
72.9678,	213,
72.9872,	195,
73.0067,	189,
73.0261,	192,
73.0456,	207,
73.0651,	195,

73.0845, 191,

73.104, 195,

73.1234, 218,

73.1429, 196,

73.1623, 212,

73.1818, 186,

73.2013, 208,

73.2207, 211,

73.2402, 185,

73.2596, 200,

73.2791, 185,

73.2986, 225,

73.318, 218,

73.3375, 210,

73.3569, 188,

73.3764, 193,

73.3958, 197,

73.4153, 182,

73.4348, 200,

73.4542, 180,

73.4737, 200,

73.4931, 205,

73.5126, 212,

73.5321, 187,

73.5515, 200,

73.571, 209,

73.5904, 187,

73.6099,	219,
73.6293,	203,
73.6488,	195,
73.6683,	246,
73.6877,	191,
73.7072,	193,
73.7266,	187,
73.7461,	206,
73.7656,	205,
73.785,	202,
73.8045,	218,
73.8239,	241,
73.8434,	225,
73.8628,	204,
73.8823,	195,
73.9018,	232,
73.9212,	199,
73.9407,	181,
73.9601,	192,
73.9796,	212,
73.9991,	197,
74.0185,	203,
74.038,	202,
74.0574,	192,
74.0769,	190,
74.0963,	196,
74.1158,	192,

74.1353, 219,

74.1547, 210,

74.1742, 195,

74.1936, 180,

74.2131, 203,

74.2326, 221,

74.252, 192,

74.2715, 206,

74.2909, 184,

74.3104, 183,

74.3298, 209,

74.3493, 208,

74.3688, 213,

74.3882, 201,

74.4077, 185,

74.4271, 223,

74.4466, 199,

74.466, 224,

74.4855, 173,

74.505, 205,

74.5244, 177,

74.5439, 181,

74.5633, 181,

74.5828, 191,

74.6023, 206,

74.6217, 214,

74.6412, 199,

74.6606,	194,
74.6801,	224,
74.6995,	209,
74.719,	202,
74.7385,	197,
74.7579,	231,
74.7774,	172,
74.7968,	213,
74.8163,	235,
74.8358,	216,
74.8552,	186,
74.8747,	202,
74.8941,	204,
74.9136,	187,
74.933,	207,
74.9525,	202,
74.972,	201,
74.9914,	206,
75.0109,	220,
75.0303,	193,
75.0498,	190,
75.0693,	178,
75.0887,	190,
75.1082,	214,
75.1276,	199,
75.1471,	209,
75.1665,	226,

75.186, 200,

75.2055, 180,

75.2249, 190,

75.2444, 200,

75.2638, 197,

75.2833, 217,

75.3028, 207,

75.3222, 182,

75.3417, 191,

75.3611, 179,

75.3806, 193,

75.4, 200,

75.4195, 211,

75.439, 209,

75.4584, 207,

75.4779, 188,

75.4973, 201,

75.5168, 207,

75.5363, 194,

75.5557, 186,

75.5752, 202,

75.5946, 195,

75.6141, 191,

75.6335, 206,

75.653, 214,

75.6725, 184,

75.6919, 187,

75.7114, 199,

75.7308, 175,

75.7503, 216,

75.7698, 208,

75.7892, 206,

75.8087, 187,

75.8281, 220,

75.8476, 194,

75.867, 209,

75.8865, 200,

75.906, 203,

75.9254, 216,

75.9449, 209,

75.9643, 195,

75.9838, 205,

76.0033, 175,

76.0227, 223,

76.0422, 204,

76.0616, 217,

76.0811, 207,

76.1005, 176,

76.12, 195,

76.1395, 197,

76.1589, 213,

76.1784, 215,

76.1978, 198,

76.2173, 198,

76.2368,	208,
76.2562,	193,
76.2757,	206,
76.2951,	168,
76.3146,	228,
76.334,	202,
76.3535,	195,
76.373,	186,
76.3924,	214,
76.4119,	222,
76.4313,	198,
76.4508,	197,
76.4703,	217,
76.4897,	188,
76.5092,	201,
76.5286,	211,
76.5481,	201,
76.5675,	203,
76.587,	176,
76.6065,	185,
76.6259,	173,
76.6454,	192,
76.6648,	209,
76.6843,	214,
76.7038,	192,
76.7232,	215,
76.7427,	203,

76.7621,	209,
76.7816,	229,
76.801,	209,
76.8205,	182,
76.84,	181,
76.8594,	193,
76.8789,	185,
76.8983,	192,
76.9178,	192,
76.9373,	205,
76.9567,	196,
76.9762,	189,
76.9956,	209,
77.0151,	205,
77.0345,	192,
77.054,	205,
77.0735,	190,
77.0929,	184,
77.1124,	241,
77.1318,	190,
77.1513,	200,
77.1707,	196,
77.1902,	189,
77.2097,	198,
77.2291,	227,
77.2486,	214,
77.268,	210,

77.2875,	209,
77.307,	211,
77.3264,	214,
77.3459,	215,
77.3653,	236,
77.3848,	208,
77.4042,	187,
77.4237,	196,
77.4432,	216,
77.4626,	175,
77.4821,	194,
77.5015,	192,
77.521,	202,
77.5405,	190,
77.5599,	233,
77.5794,	200,
77.5988,	215,
77.6183,	236,
77.6377,	204,
77.6572,	188,
77.6767,	191,
77.6961,	201,
77.7156,	213,
77.735,	214,
77.7545,	222,
77.774,	219,
77.7934,	201,

77.8129,	191,
77.8323,	226,
77.8518,	210,
77.8712,	207,
77.8907,	199,
77.9102,	196,
77.9296,	195,
77.9491,	195,
77.9685,	229,
77.988,	193,
78.0075,	216,
78.0269,	213,
78.0464,	184,
78.0658,	213,
78.0853,	207,
78.1047,	185,
78.1242,	177,
78.1437,	163,
78.1631,	197,
78.1826,	195,
78.202,	205,
78.2215,	203,
78.241,	220,
78.2604,	189,
78.2799,	183,
78.2993,	175,
78.3188,	187,

78.3382,	194,
78.3577,	187,
78.3772,	198,
78.3966,	193,
78.4161,	206,
78.4355,	220,
78.455,	184,
78.4745,	205,
78.4939,	197,
78.5134,	211,
78.5328,	176,
78.5523,	205,
78.5717,	206,
78.5912,	208,
78.6107,	209,
78.6301,	224,
78.6496,	210,
78.669,	191,
78.6885,	215,
78.708,	208,
78.7274,	209,
78.7469,	218,
78.7663,	189,
78.7858,	218,
78.8052,	180,
78.8247,	175,
78.8442,	217,

78.8636,	186,
78.8831,	196,
78.9025,	195,
78.922,	225,
78.9415,	199,
78.9609,	234,
78.9804,	221,
78.9998,	227,
79.0193,	214,
79.0387,	204,
79.0582,	222,
79.0777,	182,
79.0971,	172,
79.1166,	180,
79.136,	241,
79.1555,	216,
79.175,	181,
79.1944,	201,
79.2139,	196,
79.2333,	213,
79.2528,	197,
79.2722,	189,
79.2917,	188,
79.3112,	220,
79.3306,	214,
79.3501,	183,
79.3695,	163,

79.389,	195,
79.4085,	193,
79.4279,	181,
79.4474,	207,
79.4668,	212,
79.4863,	200,
79.5057,	178,
79.5252,	223,
79.5447,	218,
79.5641,	204,
79.5836,	211,
79.603,	217,
79.6225,	200,
79.642,	203,
79.6614,	238,
79.6809,	204,
79.7003,	197,
79.7198,	196,
79.7392,	194,
79.7587,	193,
79.7782,	185,
79.7976,	212,
79.8171,	198,
79.8365,	193,
79.856,	203,
79.8755,	203,
79.8949,	210,

79.9144,	198,
79.9338,	202,
79.9533,	223,
79.9727,	195,
79.9922,	189,
80.0117,	206,
80.0311,	196,
80.0506,	201,
80.07,	200,
80.0895,	207,
80.1089,	200,
80.1284,	229,
80.1479,	213,
80.1673,	194,
80.1868,	180,
80.2062,	176,
80.2257,	243,
80.2452,	192,
80.2646,	206,
80.2841,	234,
80.3035,	213,
80.323,	221,
80.3424,	196,
80.3619,	213,
80.3814,	212,
80.4008,	199,
80.4203,	201,

80.4397,	186,
80.4592,	209,
80.4787,	174,
80.4981,	205,
80.5176,	186,
80.537,	206,
80.5565,	213,
80.5759,	207,
80.5954,	197,
80.6149,	218,
80.6343,	216,
80.6538,	197,
80.6732,	202,
80.6927,	186,
80.7122,	216,
80.7316,	222,
80.7511,	214,
80.7705,	201,
80.79,	191,
80.8094,	193,
80.8289,	191,
80.8484,	229,
80.8678,	197,
80.8873,	222,
80.9067,	199,
80.9262,	202,
80.9457,	191,

80.9651, 174,

80.9846, 204,

81.004, 190,

81.0235, 194,

81.0429, 207,

81.0624, 204,

81.0819, 201,

81.1013, 209,

81.1208, 196,

81.1402, 198,

81.1597, 185,

81.1792, 198,

81.1986, 210,

81.2181, 232,

81.2375, 189,

81.257, 202,

81.2764, 209,

81.2959, 189,

81.3154, 213,

81.3348, 204,

81.3543, 201,

81.3737, 200,

81.3932, 199,

81.4127, 213,

81.4321, 207,

81.4516, 221,

81.471, 222,

81.4905,	170,
81.5099,	216,
81.5294,	203,
81.5489,	194,
81.5683,	205,
81.5878,	179,
81.6072,	183,
81.6267,	177,
81.6462,	194,
81.6656,	188,
81.6851,	191,
81.7045,	202,
81.724,	200,
81.7434,	195,
81.7629,	234,
81.7824,	213,
81.8018,	166,
81.8213,	209,
81.8407,	210,
81.8602,	187,
81.8797,	166,
81.8991,	210,
81.9186,	202,
81.938,	212,
81.9575,	222,
81.9769,	207,
81.9964,	210,

82.0159,	221,
82.0353,	221,
82.0548,	201,
82.0742,	198,
82.0937,	228,
82.1132,	176,
82.1326,	192,
82.1521,	192,
82.1715,	213,
82.191,	205,
82.2104,	226,
82.2299,	201,
82.2494,	197,
82.2688,	189,
82.2883,	205,
82.3077,	213,
82.3272,	202,
82.3467,	214,
82.3661,	197,
82.3856,	231,
82.405,	199,
82.4245,	179,
82.4439,	197,
82.4634,	210,
82.4829,	211,
82.5023,	207,
82.5218,	233,

82.5412, 229,

82.5607, 192,

82.5802, 185,

82.5996, 193,

82.6191, 215,

82.6385, 205,

82.658, 209,

82.6774, 222,

82.6969, 213,

82.7164, 214,

82.7358, 195,

82.7553, 224,

82.7747, 215,

82.7942, 200,

82.8137, 216,

82.8331, 201,

82.8526, 199,

82.872, 198,

82.8915, 194,

82.9109, 207,

82.9304, 209,

82.9499, 213,

82.9693, 203,

82.9888, 208,

83.0082, 211,

83.0277, 190,

83.0471, 193,

83.0666,	231,
83.0861,	208,
83.1055,	188,
83.125,	249,
83.1444,	188,
83.1639,	224,
83.1834,	194,
83.2028,	186,
83.2223,	208,
83.2417,	208,
83.2612,	169,
83.2806,	206,
83.3001,	195,
83.3196,	196,
83.339,	204,
83.3585,	203,
83.3779,	204,
83.3974,	205,
83.4169,	212,
83.4363,	205,
83.4558,	191,
83.4752,	190,
83.4947,	182,
83.5141,	214,
83.5336,	214,
83.5531,	203,
83.5725,	212,

83.592,	205,
83.6114,	192,
83.6309,	226,
83.6504,	207,
83.6698,	187,
83.6893,	209,
83.7087,	187,
83.7282,	211,
83.7476,	211,
83.7671,	195,
83.7866,	205,
83.806,	210,
83.8255,	237,
83.8449,	205,
83.8644,	202,
83.8839,	203,
83.9033,	212,
83.9228,	193,
83.9422,	206,
83.9617,	217,
83.9811,	200,
84.0006,	194,
84.0201,	193,
84.0395,	208,
84.059,	199,
84.0784,	200,
84.0979,	191,

84.1174, 192,

84.1368, 219,

84.1563, 186,

84.1757, 204,

84.1952, 211,

84.2146, 219,

84.2341, 191,

84.2536, 197,

84.273, 206,

84.2925, 208,

84.3119, 216,

84.3314, 200,

84.3509, 183,

84.3703, 214,

84.3898, 202,

84.4092, 192,

84.4287, 203,

84.4481, 227,

84.4676, 216,

84.4871, 216,

84.5065, 202,

84.526, 208,

84.5454, 217,

84.5649, 192,

84.5844, 211,

84.6038, 214,

84.6233, 194,

84.6427, 215,

84.6622, 214,

84.6816, 194,

84.7011, 184,

84.7206, 213,

84.74, 203,

84.7595, 194,

84.7789, 225,

84.7984, 212,

84.8179, 223,

84.8373, 197,

84.8568, 187,

84.8762, 230,

84.8957, 197,

84.9151, 198,

84.9346, 206,

84.9541, 204,

84.9735, 183,

84.993, 183,

85.0124, 220,

85.0319, 216,

85.0514, 213,

85.0708, 205,

85.0903, 192,

85.1097, 214,

85.1292, 193,

85.1486, 176,

85.1681, 201,

85.1876, 216,

85.207, 200,

85.2265, 191,

85.2459, 201,

85.2654, 217,

85.2849, 208,

85.3043, 224,

85.3238, 210,

85.3432, 199,

85.3627, 198,

85.3821, 214,

85.4016, 200,

85.4211, 197,

85.4405, 199,

85.46, 201,

85.4794, 193,

85.4989, 207,

85.5184, 212,

85.5378, 216,

85.5573, 177,

85.5767, 192,

85.5962, 184,

85.6156, 203,

85.6351, 198,

85.6546, 228,

85.674, 202,

85.6935, 204,

85.7129, 206,

85.7324, 197,

85.7519, 190,

85.7713, 188,

85.7908, 199,

85.8102, 216,

85.8297, 204,

85.8491, 231,

85.8686, 213,

85.8881, 213,

85.9075, 197,

85.927, 200,

85.9464, 225,

85.9659, 202,

85.9853, 220,

86.0048, 228,

86.0243, 211,

86.0437, 175,

86.0632, 223,

86.0826, 208,

86.1021, 209,

86.1216, 206,

86.141, 221,

86.1605, 219,

86.1799, 201,

86.1994, 212,

86.2188,	234,
86.2383,	227,
86.2578,	204,
86.2772,	214,
86.2967,	219,
86.3161,	202,
86.3356,	205,
86.3551,	218,
86.3745,	224,
86.394,	219,
86.4134,	199,
86.4329,	187,
86.4523,	219,
86.4718,	203,
86.4913,	200,
86.5107,	234,
86.5302,	174,
86.5496,	189,
86.5691,	208,
86.5886,	201,
86.608,	181,
86.6275,	219,
86.6469,	212,
86.6664,	204,
86.6858,	199,
86.7053,	203,
86.7248,	208,

86.7442,	179,
86.7637,	218,
86.7831,	194,
86.8026,	225,
86.8221,	207,
86.8415,	183,
86.861,	229,
86.8804,	215,
86.8999,	215,
86.9193,	193,
86.9388,	213,
86.9583,	213,
86.9777,	220,
86.9972,	192,
87.0166,	210,
87.0361,	181,
87.0556,	209,
87.075,	230,
87.0945,	225,
87.1139,	218,
87.1334,	190,
87.1528,	201,
87.1723,	216,
87.1918,	216,
87.2112,	190,
87.2307,	233,
87.2501,	218,

87.2696,	203,
87.2891,	221,
87.3085,	208,
87.328,	201,
87.3474,	199,
87.3669,	236,
87.3863,	172,
87.4058,	212,
87.4253,	183,
87.4447,	219,
87.4642,	185,
87.4836,	206,
87.5031,	222,
87.5226,	224,
87.542,	186,
87.5615,	196,
87.5809,	200,
87.6004,	202,
87.6198,	179,
87.6393,	216,
87.6588,	192,
87.6782,	197,
87.6977,	201,
87.7171,	196,
87.7366,	206,
87.7561,	211,
87.7755,	199,

87.795, 239,

87.8144, 199,

87.8339, 190,

87.8533, 194,

87.8728, 205,

87.8923, 199,

87.9117, 226,

87.9312, 215,

87.9506, 204,

87.9701, 190,

87.9896, 212,

88.009, 231,

88.0285, 202,

88.0479, 193,

88.0674, 202,

88.0868, 190,

88.1063, 214,

88.1258, 187,

88.1452, 204,

88.1647, 200,

88.1841, 188,

88.2036, 191,

88.2231, 202,

88.2425, 250,

88.262, 230,

88.2814, 198,

88.3009, 195,

88.3203,	197,
88.3398,	198,
88.3593,	197,
88.3787,	186,
88.3982,	201,
88.4176,	183,
88.4371,	204,
88.4566,	209,
88.476,	216,
88.4955,	192,
88.5149,	212,
88.5344,	201,
88.5538,	213,
88.5733,	201,
88.5928,	199,
88.6122,	203,
88.6317,	219,
88.6511,	209,
88.6706,	207,
88.69,	224,
88.7095,	245,
88.729,	176,
88.7484,	204,
88.7679,	227,
88.7873,	212,
88.8068,	186,
88.8263,	199,

88.8457,	197,
88.8652,	197,
88.8846,	218,
88.9041,	201,
88.9235,	204,
88.943,	213,
88.9625,	213,
88.9819,	216,
89.0014,	182,
89.0208,	199,
89.0403,	215,
89.0598,	203,
89.0792,	211,
89.0987,	212,
89.1181,	201,
89.1376,	188,
89.157,	201,
89.1765,	183,
89.196,	218,
89.2154,	191,
89.2349,	217,
89.2543,	236,
89.2738,	201,
89.2933,	212,
89.3127,	205,
89.3322,	187,
89.3516,	184,

89.3711,	223,
89.3905,	187,
89.41,	212,
89.4295,	226,
89.4489,	192,
89.4684,	210,
89.4878,	203,
89.5073,	195,
89.5268,	207,
89.5462,	195,
89.5657,	204,
89.5851,	191,
89.6046,	209,
89.624,	221,
89.6435,	207,
89.663,	216,
89.6824,	200,
89.7019,	188,
89.7213,	192,
89.7408,	186,
89.7603,	202,
89.7797,	190,
89.7992,	173,
89.8186,	216,
89.8381,	208,
89.8575,	230,
89.877,	205,

;RAW4.00

[RawHeader]

Date=05/11/2023

EstimatedTotalTime=616.4

FurtherDQLReading=0

HardwareIndicator=65519

MeasuredTotalTime=616.4

MeasurementFlag=1

NumberOfMeasuredRanges=1

NumberOfRanges=1

Time=10:54:24

[VarInfo]

Type=USER

Flags=0

Value=Lab Manager

[VarInfo]

Type=SAMPLEID

Flags=0

Value=

[VarInfo]

Type=COMMENT

Flags=0

Value=

[VarInfo]

Type=UTF

Flags=0

Value=

[VarInfo]

Type=CREATOR

Flags=0

Value=BrmlToV4Converter

[VarInfo]

Type=CREATOR_VERSION

Flags=0

Value=6.5.0.0

[HardwareConfiguration]

AbsorptionFactor=9999

ActivateAbsorber=0

Alpha1=1.5406

Alpha2=1.54439

AlphaAverage=1.5418

AlphaRatio=0.5

Analyzer=0

Anode=Cu

AntiScatteringSlit=9999

BeamOpticsFlags=0

Beta=1.39222

BetaRelativeIntensity=0

DeactivateAbsorber=0

DetectorSlit=9999

DivergenceSlit=10.5

GoniometerControl=0

GoniometerDiameter=560

GoniometerModel=512

GoniometerStage=7

Monochromator=0

NearSampleSlit=9999

PrimarySollerSlit=2.5

SampleChanger=0

SecondSollerSlit=9999

SynchronousAxis=0

ThinFilmAttachment=9999

WaveUnit=A

[RangeHeader]

ActuallyUsedLambda=1.5418

AdditionalDetectorMask=257

DataRecordLength=4

DelayTime=0

DisplayPlaneNumber=0

EstimatedScanTime=616

ExtraParametersMask=0

GeneratorCurrent=40

GeneratorVoltage=40

Increment=0.0194583

Increment3=0

NumberOfCompletedData=3850

NumberOfCounts=1

NumberOfDetectors=0

NumberOfDrives=3

NumberOfEncoderDrives=0

NumberOfMeasuredData=3850

NumberOfVaryingParameters=0

RangeStartTime=0

RotationSpeed=15

ScanMode=1

ScanType=Locked Coupled

SimulatedMeasConditions=0

SlitChangerIn=0

SmootingWidth=0

Start=5

Steps=3850

SynchronousRotation=0

Time=28.8

[VarInfo]

Type=PSD_DISCRIM

Flags=0

Value=0.11;0.25

[PSD]

ActualTwoTheta=9999

FirstUsedChannel=0

FixedPSD=0

PSDOpening=2.94947

PSDType=5

[Drive]

DriveFlags=1

DriveName=2Theta

DriveNumber=2

DriveOffset=0

OffsetIndex=0

OscillationAmplitude=0

OscillationSpeed=0

StartPosition=5

[Drive]

DriveFlags=1

DriveName=Theta

DriveNumber=1

DriveOffset=0

OffsetIndex=0

OscillationAmplitude=0

OscillationSpeed=0

StartPosition=2.5

[Drive]

DriveFlags=1

DriveName=Phi

DriveNumber=3

DriveOffset=0

OffsetIndex=0

OscillationAmplitude=0

OscillationSpeed=0

StartPosition=83.125

[Data]

Angle, PSD,

5, 259,

5.01946, 290,

5.03892, 273,

5.05837, 251,

5.07783, 281,

5.09729, 286,

5.11675, 269,

5.13621, 268,

5.15567, 262,

5.17512, 243,

5.19458, 235,

5.21404, 262,

5.2335, 264,

5.25296, 225,

5.27242, 261,

5.29187, 279,

5.31133,	227,
5.33079,	235,
5.35025,	222,
5.36971,	221,
5.38917,	223,
5.40862,	212,
5.42808,	213,
5.44754,	182,
5.467,	189,
5.48646,	175,
5.50592,	204,
5.52537,	176,
5.54483,	171,
5.56429,	152,
5.58375,	132,
5.60321,	143,
5.62266,	141,
5.64212,	150,
5.66158,	150,
5.68104,	151,
5.7005,	145,
5.71996,	130,
5.73941,	132,
5.75887,	128,
5.77833,	107,
5.79779,	122,
5.81725,	109,

5.83671,	98,
5.85616,	129,
5.87562,	107,
5.89508,	108,
5.91454,	104,
5.934,	95,
5.95346,	107,
5.97291,	96,
5.99237,	119,
6.01183,	82,
6.03129,	102,
6.05075,	89,
6.07021,	82,
6.08966,	98,
6.10912,	97,
6.12858,	116,
6.14804,	90,
6.1675,	96,
6.18695,	94,
6.20641,	100,
6.22587,	95,
6.24533,	94,
6.26479,	103,
6.28425,	119,
6.3037,	107,
6.32316,	90,
6.34262,	82,

6.36208,	77,
6.38154,	79,
6.401,	96,
6.42045,	103,
6.43991,	93,
6.45937,	75,
6.47883,	92,
6.49829,	87,
6.51775,	88,
6.5372,	82,
6.55666,	80,
6.57612,	80,
6.59558,	90,
6.61504,	86,
6.6345,	69,
6.65395,	74,
6.67341,	83,
6.69287,	77,
6.71233,	81,
6.73179,	86,
6.75124,	68,
6.7707,	89,
6.79016,	87,
6.80962,	89,
6.82908,	78,
6.84854,	95,
6.86799,	90,

6.88745,	80,
6.90691,	82,
6.92637,	74,
6.94583,	78,
6.96529,	81,
6.98474,	79,
7.0042,	87,
7.02366,	94,
7.04312,	74,
7.06258,	74,
7.08204,	108,
7.10149,	89,
7.12095,	90,
7.14041,	76,
7.15987,	87,
7.17933,	83,
7.19879,	86,
7.21824,	84,
7.2377,	76,
7.25716,	80,
7.27662,	63,
7.29608,	77,
7.31553,	90,
7.33499,	88,
7.35445,	69,
7.37391,	68,
7.39337,	87,

7.41283, 98,

7.43228, 75,

7.45174, 76,

7.4712, 85,

7.49066, 74,

7.51012, 85,

7.52958, 71,

7.54903, 72,

7.56849, 86,

7.58795, 78,

7.60741, 78,

7.62687, 74,

7.64633, 81,

7.66578, 76,

7.68524, 70,

7.7047, 72,

7.72416, 74,

7.74362, 76,

7.76308, 74,

7.78253, 66,

7.80199, 70,

7.82145, 83,

7.84091, 71,

7.86037, 81,

7.87983, 71,

7.89928, 72,

7.91874, 70,

7.9382,	85,
7.95766,	67,
7.97712,	75,
7.99657,	74,
8.01603,	80,
8.03549,	72,
8.05495,	70,
8.07441,	66,
8.09387,	82,
8.11332,	78,
8.13278,	70,
8.15224,	88,
8.1717,	67,
8.19116,	70,
8.21062,	79,
8.23007,	77,
8.24953,	77,
8.26899,	82,
8.28845,	90,
8.30791,	84,
8.32737,	74,
8.34682,	68,
8.36628,	77,
8.38574,	74,
8.4052,	82,
8.42466,	80,
8.44412,	96,

8.46357,	67,
8.48303,	66,
8.50249,	78,
8.52195,	73,
8.54141,	80,
8.56086,	75,
8.58032,	104,
8.59978,	77,
8.61924,	80,
8.6387,	93,
8.65816,	86,
8.67761,	97,
8.69707,	78,
8.71653,	91,
8.73599,	80,
8.75545,	88,
8.77491,	85,
8.79436,	78,
8.81382,	72,
8.83328,	96,
8.85274,	66,
8.8722,	78,
8.89166,	87,
8.91111,	72,
8.93057,	70,
8.95003,	95,
8.96949,	105,

8.98895,	92,
9.00841,	74,
9.02786,	83,
9.04732,	80,
9.06678,	76,
9.08624,	72,
9.1057,	83,
9.12515,	89,
9.14461,	94,
9.16407,	64,
9.18353,	88,
9.20299,	93,
9.22245,	88,
9.2419,	69,
9.26136,	84,
9.28082,	87,
9.30028,	80,
9.31974,	76,
9.3392,	79,
9.35865,	91,
9.37811,	87,
9.39757,	83,
9.41703,	90,
9.43649,	107,
9.45595,	83,
9.4754,	80,
9.49486,	81,

9.51432, 92,

9.53378, 86,

9.55324, 80,

9.5727, 91,

9.59215, 83,

9.61161, 81,

9.63107, 87,

9.65053, 84,

9.66999, 96,

9.68944, 96,

9.7089, 107,

9.72836, 102,

9.74782, 93,

9.76728, 79,

9.78674, 110,

9.80619, 114,

9.82565, 84,

9.84511, 77,

9.86457, 87,

9.88403, 99,

9.90349, 97,

9.92294, 106,

9.9424, 96,

9.96186, 93,

9.98132, 102,

10.0008, 99,

10.0202, 96,

10.0397,	87,
10.0592,	100,
10.0786,	113,
10.0981,	98,
10.1175,	104,
10.137,	89,
10.1564,	102,
10.1759,	86,
10.1954,	87,
10.2148,	89,
10.2343,	107,
10.2537,	91,
10.2732,	114,
10.2927,	93,
10.3121,	86,
10.3316,	92,
10.351,	88,
10.3705,	84,
10.3899,	103,
10.4094,	101,
10.4289,	110,
10.4483,	110,
10.4678,	104,
10.4872,	86,
10.5067,	101,
10.5262,	131,
10.5456,	93,

10.5651,	95,
10.5845,	96,
10.604,	100,
10.6234,	99,
10.6429,	93,
10.6624,	107,
10.6818,	88,
10.7013,	100,
10.7207,	85,
10.7402,	113,
10.7597,	124,
10.7791,	108,
10.7986,	90,
10.818,	110,
10.8375,	95,
10.8569,	101,
10.8764,	97,
10.8959,	103,
10.9153,	103,
10.9348,	106,
10.9542,	102,
10.9737,	98,
10.9931,	116,
11.0126,	99,
11.0321,	125,
11.0515,	116,
11.071,	126,

11.0904,	114,
11.1099,	110,
11.1294,	102,
11.1488,	99,
11.1683,	94,
11.1877,	114,
11.2072,	113,
11.2266,	111,
11.2461,	119,
11.2656,	104,
11.285,	116,
11.3045,	104,
11.3239,	100,
11.3434,	97,
11.3629,	115,
11.3823,	106,
11.4018,	103,
11.4212,	97,
11.4407,	116,
11.4601,	99,
11.4796,	113,
11.4991,	102,
11.5185,	95,
11.538,	116,
11.5574,	103,
11.5769,	115,
11.5964,	94,

11.6158,	117,
11.6353,	123,
11.6547,	112,
11.6742,	118,
11.6936,	117,
11.7131,	92,
11.7326,	118,
11.752,	96,
11.7715,	96,
11.7909,	111,
11.8104,	111,
11.8299,	100,
11.8493,	106,
11.8688,	125,
11.8882,	103,
11.9077,	108,
11.9271,	117,
11.9466,	107,
11.9661,	95,
11.9855,	118,
12.005,	116,
12.0244,	118,
12.0439,	128,
12.0634,	113,
12.0828,	94,
12.1023,	109,
12.1217,	121,

12.1412,	111,
12.1606,	137,
12.1801,	112,
12.1996,	117,
12.219,	117,
12.2385,	114,
12.2579,	108,
12.2774,	116,
12.2969,	115,
12.3163,	112,
12.3358,	95,
12.3552,	147,
12.3747,	108,
12.3941,	128,
12.4136,	128,
12.4331,	119,
12.4525,	131,
12.472,	123,
12.4914,	128,
12.5109,	135,
12.5304,	111,
12.5498,	108,
12.5693,	129,
12.5887,	107,
12.6082,	93,
12.6276,	106,
12.6471,	121,

12.6666,	114,
12.686,	97,
12.7055,	111,
12.7249,	128,
12.7444,	121,
12.7639,	125,
12.7833,	129,
12.8028,	105,
12.8222,	107,
12.8417,	116,
12.8611,	124,
12.8806,	119,
12.9001,	133,
12.9195,	106,
12.939,	120,
12.9584,	110,
12.9779,	128,
12.9974,	103,
13.0168,	109,
13.0363,	107,
13.0557,	115,
13.0752,	115,
13.0946,	118,
13.1141,	128,
13.1336,	130,
13.153,	133,
13.1725,	119,

13.1919,	111,
13.2114,	138,
13.2309,	123,
13.2503,	127,
13.2698,	108,
13.2892,	119,
13.3087,	115,
13.3281,	128,
13.3476,	122,
13.3671,	111,
13.3865,	128,
13.406,	122,
13.4254,	115,
13.4449,	124,
13.4644,	115,
13.4838,	123,
13.5033,	120,
13.5227,	126,
13.5422,	107,
13.5616,	126,
13.5811,	142,
13.6006,	130,
13.62,	124,
13.6395,	123,
13.6589,	143,
13.6784,	118,
13.6978,	125,

13.7173,	101,
13.7368,	108,
13.7562,	114,
13.7757,	122,
13.7951,	106,
13.8146,	115,
13.8341,	104,
13.8535,	103,
13.873,	107,
13.8924,	121,
13.9119,	103,
13.9313,	93,
13.9508,	106,
13.9703,	115,
13.9897,	129,
14.0092,	125,
14.0286,	111,
14.0481,	131,
14.0676,	111,
14.087,	119,
14.1065,	100,
14.1259,	134,
14.1454,	140,
14.1648,	128,
14.1843,	119,
14.2038,	108,
14.2232,	127,

14.2427, 117,

14.2621, 135,

14.2816, 130,

14.3011, 137,

14.3205, 113,

14.34, 139,

14.3594, 102,

14.3789, 120,

14.3983, 115,

14.4178, 107,

14.4373, 107,

14.4567, 100,

14.4762, 103,

14.4956, 118,

14.5151, 111,

14.5346, 129,

14.554, 116,

14.5735, 110,

14.5929, 123,

14.6124, 109,

14.6318, 128,

14.6513, 113,

14.6708, 145,

14.6902, 111,

14.7097, 118,

14.7291, 122,

14.7486, 99,

14.7681,	130,
14.7875,	112,
14.807,	127,
14.8264,	132,
14.8459,	123,
14.8653,	118,
14.8848,	124,
14.9043,	124,
14.9237,	122,
14.9432,	114,
14.9626,	119,
14.9821,	119,
15.0016,	117,
15.021,	114,
15.0405,	120,
15.0599,	131,
15.0794,	117,
15.0988,	136,
15.1183,	113,
15.1378,	135,
15.1572,	115,
15.1767,	133,
15.1961,	132,
15.2156,	139,
15.2351,	115,
15.2545,	123,
15.274,	145,

15.2934, 125,

15.3129, 125,

15.3323, 129,

15.3518, 112,

15.3713, 118,

15.3907, 122,

15.4102, 118,

15.4296, 121,

15.4491, 91,

15.4686, 121,

15.488, 149,

15.5075, 147,

15.5269, 103,

15.5464, 113,

15.5658, 107,

15.5853, 128,

15.6048, 105,

15.6242, 146,

15.6437, 114,

15.6631, 114,

15.6826, 104,

15.7021, 118,

15.7215, 112,

15.741, 121,

15.7604, 103,

15.7799, 122,

15.7993, 139,

15.8188, 121,

15.8383, 117,

15.8577, 104,

15.8772, 113,

15.8966, 97,

15.9161, 105,

15.9356, 132,

15.955, 119,

15.9745, 104,

15.9939, 126,

16.0134, 125,

16.0328, 139,

16.0523, 122,

16.0718, 126,

16.0912, 120,

16.1107, 128,

16.1301, 113,

16.1496, 151,

16.1691, 122,

16.1885, 119,

16.208, 113,

16.2274, 129,

16.2469, 148,

16.2663, 107,

16.2858, 124,

16.3053, 113,

16.3247, 120,

16.3442,	124,
16.3636,	123,
16.3831,	111,
16.4026,	117,
16.422,	131,
16.4415,	112,
16.4609,	117,
16.4804,	128,
16.4998,	128,
16.5193,	132,
16.5388,	106,
16.5582,	109,
16.5777,	121,
16.5971,	122,
16.6166,	128,
16.636,	130,
16.6555,	102,
16.675,	120,
16.6944,	122,
16.7139,	110,
16.7333,	118,
16.7528,	134,
16.7723,	141,
16.7917,	94,
16.8112,	135,
16.8306,	111,
16.8501,	123,

16.8695,	130,
16.889,	131,
16.9085,	131,
16.9279,	120,
16.9474,	116,
16.9668,	122,
16.9863,	124,
17.0058,	119,
17.0252,	129,
17.0447,	133,
17.0641,	114,
17.0836,	124,
17.103,	123,
17.1225,	106,
17.142,	110,
17.1614,	121,
17.1809,	117,
17.2003,	138,
17.2198,	130,
17.2393,	128,
17.2587,	117,
17.2782,	114,
17.2976,	112,
17.3171,	135,
17.3365,	135,
17.356,	125,
17.3755,	114,

17.3949, 120,

17.4144, 141,

17.4338, 116,

17.4533, 108,

17.4728, 131,

17.4922, 124,

17.5117, 117,

17.5311, 135,

17.5506, 115,

17.57, 116,

17.5895, 155,

17.609, 140,

17.6284, 114,

17.6479, 124,

17.6673, 122,

17.6868, 130,

17.7063, 121,

17.7257, 137,

17.7452, 103,

17.7646, 112,

17.7841, 123,

17.8035, 131,

17.823, 143,

17.8425, 127,

17.8619, 116,

17.8814, 134,

17.9008, 102,

17.9203, 103,

17.9398, 111,

17.9592, 131,

17.9787, 112,

17.9981, 124,

18.0176, 116,

18.037, 111,

18.0565, 134,

18.076, 112,

18.0954, 125,

18.1149, 107,

18.1343, 131,

18.1538, 121,

18.1733, 103,

18.1927, 135,

18.2122, 117,

18.2316, 110,

18.2511, 134,

18.2705, 99,

18.29, 117,

18.3095, 120,

18.3289, 97,

18.3484, 126,

18.3678, 135,

18.3873, 122,

18.4068, 117,

18.4262, 127,

18.4457,	123,
18.4651,	121,
18.4846,	123,
18.504,	106,
18.5235,	141,
18.543,	132,
18.5624,	112,
18.5819,	124,
18.6013,	131,
18.6208,	132,
18.6403,	120,
18.6597,	134,
18.6792,	116,
18.6986,	118,
18.7181,	113,
18.7375,	108,
18.757,	136,
18.7765,	120,
18.7959,	125,
18.8154,	113,
18.8348,	133,
18.8543,	141,
18.8738,	109,
18.8932,	140,
18.9127,	118,
18.9321,	132,
18.9516,	130,

18.971,	120,
18.9905,	132,
19.01,	125,
19.0294,	124,
19.0489,	118,
19.0683,	124,
19.0878,	101,
19.1073,	129,
19.1267,	136,
19.1462,	130,
19.1656,	107,
19.1851,	118,
19.2045,	131,
19.224,	122,
19.2435,	128,
19.2629,	114,
19.2824,	118,
19.3018,	125,
19.3213,	105,
19.3408,	104,
19.3602,	127,
19.3797,	126,
19.3991,	100,
19.4186,	157,
19.438,	126,
19.4575,	142,
19.477,	123,

19.4964,	108,
19.5159,	129,
19.5353,	106,
19.5548,	115,
19.5742,	114,
19.5937,	120,
19.6132,	135,
19.6326,	119,
19.6521,	129,
19.6715,	123,
19.691,	129,
19.7105,	118,
19.7299,	134,
19.7494,	98,
19.7688,	120,
19.7883,	132,
19.8077,	140,
19.8272,	138,
19.8467,	128,
19.8661,	134,
19.8856,	131,
19.905,	123,
19.9245,	135,
19.944,	125,
19.9634,	136,
19.9829,	134,
20.0023,	148,

20.0218,	134,
20.0412,	111,
20.0607,	116,
20.0802,	111,
20.0996,	124,
20.1191,	129,
20.1385,	107,
20.158,	129,
20.1775,	121,
20.1969,	126,
20.2164,	153,
20.2358,	145,
20.2553,	129,
20.2747,	120,
20.2942,	135,
20.3137,	130,
20.3331,	138,
20.3526,	123,
20.372,	121,
20.3915,	107,
20.411,	126,
20.4304,	138,
20.4499,	123,
20.4693,	98,
20.4888,	136,
20.5082,	133,
20.5277,	147,

20.5472, 140,

20.5666, 121,

20.5861, 137,

20.6055, 122,

20.625, 152,

20.6445, 134,

20.6639, 110,

20.6834, 143,

20.7028, 117,

20.7223, 145,

20.7417, 113,

20.7612, 129,

20.7807, 124,

20.8001, 112,

20.8196, 144,

20.839, 139,

20.8585, 127,

20.878, 131,

20.8974, 137,

20.9169, 124,

20.9363, 123,

20.9558, 125,

20.9752, 132,

20.9947, 129,

21.0142, 143,

21.0336, 115,

21.0531, 127,

21.0725,	140,
21.092,	115,
21.1115,	130,
21.1309,	131,
21.1504,	124,
21.1698,	136,
21.1893,	138,
21.2087,	141,
21.2282,	132,
21.2477,	144,
21.2671,	131,
21.2866,	141,
21.306,	142,
21.3255,	135,
21.345,	144,
21.3644,	136,
21.3839,	139,
21.4033,	149,
21.4228,	114,
21.4422,	149,
21.4617,	157,
21.4812,	133,
21.5006,	111,
21.5201,	139,
21.5395,	118,
21.559,	135,
21.5785,	113,

21.5979,	142,
21.6174,	127,
21.6368,	135,
21.6563,	118,
21.6757,	148,
21.6952,	128,
21.7147,	110,
21.7341,	132,
21.7536,	116,
21.773,	144,
21.7925,	125,
21.812,	125,
21.8314,	140,
21.8509,	130,
21.8703,	126,
21.8898,	132,
21.9092,	124,
21.9287,	119,
21.9482,	115,
21.9676,	149,
21.9871,	130,
22.0065,	110,
22.026,	120,
22.0455,	116,
22.0649,	136,
22.0844,	140,
22.1038,	128,

22.1233,	105,
22.1427,	144,
22.1622,	141,
22.1817,	131,
22.2011,	123,
22.2206,	135,
22.24,	120,
22.2595,	125,
22.279,	123,
22.2984,	119,
22.3179,	126,
22.3373,	126,
22.3568,	135,
22.3762,	146,
22.3957,	113,
22.4152,	152,
22.4346,	101,
22.4541,	111,
22.4735,	127,
22.493,	148,
22.5124,	129,
22.5319,	136,
22.5514,	135,
22.5708,	131,
22.5903,	120,
22.6097,	120,
22.6292,	154,

22.6487,	137,
22.6681,	131,
22.6876,	130,
22.707,	124,
22.7265,	125,
22.7459,	122,
22.7654,	140,
22.7849,	142,
22.8043,	130,
22.8238,	125,
22.8432,	134,
22.8627,	128,
22.8822,	119,
22.9016,	120,
22.9211,	130,
22.9405,	130,
22.96,	109,
22.9794,	136,
22.9989,	127,
23.0184,	121,
23.0378,	121,
23.0573,	151,
23.0767,	137,
23.0962,	128,
23.1157,	117,
23.1351,	137,
23.1546,	142,

23.174,	134,
23.1935,	125,
23.2129,	133,
23.2324,	133,
23.2519,	114,
23.2713,	140,
23.2908,	136,
23.3102,	117,
23.3297,	135,
23.3492,	129,
23.3686,	125,
23.3881,	133,
23.4075,	124,
23.427,	131,
23.4464,	134,
23.4659,	134,
23.4854,	115,
23.5048,	118,
23.5243,	131,
23.5437,	132,
23.5632,	126,
23.5827,	145,
23.6021,	126,
23.6216,	125,
23.641,	137,
23.6605,	128,
23.6799,	127,

23.6994,	130,
23.7189,	124,
23.7383,	127,
23.7578,	139,
23.7772,	136,
23.7967,	137,
23.8162,	112,
23.8356,	131,
23.8551,	127,
23.8745,	108,
23.894,	142,
23.9134,	105,
23.9329,	137,
23.9524,	124,
23.9718,	118,
23.9913,	125,
24.0107,	139,
24.0302,	139,
24.0497,	129,
24.0691,	140,
24.0886,	143,
24.108,	111,
24.1275,	117,
24.1469,	101,
24.1664,	147,
24.1859,	143,
24.2053,	127,

24.2248, 144,

24.2442, 134,

24.2637, 124,

24.2832, 149,

24.3026, 154,

24.3221, 124,

24.3415, 138,

24.361, 128,

24.3804, 131,

24.3999, 141,

24.4194, 133,

24.4388, 125,

24.4583, 134,

24.4777, 126,

24.4972, 106,

24.5167, 144,

24.5361, 126,

24.5556, 131,

24.575, 119,

24.5945, 146,

24.6139, 142,

24.6334, 131,

24.6529, 105,

24.6723, 123,

24.6918, 127,

24.7112, 125,

24.7307, 114,

24.7502,	138,
24.7696,	113,
24.7891,	138,
24.8085,	120,
24.828,	141,
24.8474,	133,
24.8669,	109,
24.8864,	110,
24.9058,	117,
24.9253,	139,
24.9447,	118,
24.9642,	133,
24.9837,	149,
25.0031,	141,
25.0226,	107,
25.042,	129,
25.0615,	124,
25.0809,	134,
25.1004,	155,
25.1199,	113,
25.1393,	131,
25.1588,	144,
25.1782,	120,
25.1977,	155,
25.2171,	121,
25.2366,	140,
25.2561,	123,

25.2755,	139,
25.295,	118,
25.3144,	128,
25.3339,	124,
25.3534,	125,
25.3728,	127,
25.3923,	138,
25.4117,	145,
25.4312,	138,
25.4506,	124,
25.4701,	151,
25.4896,	121,
25.509,	128,
25.5285,	117,
25.5479,	123,
25.5674,	118,
25.5869,	141,
25.6063,	137,
25.6258,	133,
25.6452,	140,
25.6647,	134,
25.6841,	143,
25.7036,	122,
25.7231,	117,
25.7425,	111,
25.762,	139,
25.7814,	147,

25.8009,	149,
25.8204,	125,
25.8398,	140,
25.8593,	132,
25.8787,	145,
25.8982,	126,
25.9176,	149,
25.9371,	149,
25.9566,	129,
25.976,	143,
25.9955,	129,
26.0149,	153,
26.0344,	143,
26.0539,	154,
26.0733,	115,
26.0928,	154,
26.1122,	172,
26.1317,	148,
26.1511,	134,
26.1706,	133,
26.1901,	147,
26.2095,	129,
26.229,	151,
26.2484,	159,
26.2679,	138,
26.2874,	136,
26.3068,	120,

26.3263,	125,
26.3457,	143,
26.3652,	108,
26.3846,	138,
26.4041,	129,
26.4236,	139,
26.443,	131,
26.4625,	146,
26.4819,	155,
26.5014,	147,
26.5209,	158,
26.5403,	145,
26.5598,	121,
26.5792,	122,
26.5987,	129,
26.6181,	136,
26.6376,	158,
26.6571,	146,
26.6765,	140,
26.696,	129,
26.7154,	122,
26.7349,	118,
26.7544,	141,
26.7738,	145,
26.7933,	123,
26.8127,	121,
26.8322,	127,

26.8516, 151,

26.8711, 130,

26.8906, 148,

26.91, 120,

26.9295, 128,

26.9489, 137,

26.9684, 113,

26.9879, 142,

27.0073, 141,

27.0268, 142,

27.0462, 127,

27.0657, 121,

27.0851, 129,

27.1046, 142,

27.1241, 115,

27.1435, 145,

27.163, 134,

27.1824, 118,

27.2019, 150,

27.2214, 147,

27.2408, 129,

27.2603, 140,

27.2797, 139,

27.2992, 127,

27.3186, 136,

27.3381, 126,

27.3576, 121,

27.377,	123,
27.3965,	139,
27.4159,	140,
27.4354,	136,
27.4549,	134,
27.4743,	123,
27.4938,	133,
27.5132,	132,
27.5327,	146,
27.5521,	129,
27.5716,	145,
27.5911,	129,
27.6105,	155,
27.63,	138,
27.6494,	118,
27.6689,	130,
27.6884,	129,
27.7078,	114,
27.7273,	130,
27.7467,	143,
27.7662,	143,
27.7856,	159,
27.8051,	142,
27.8246,	139,
27.844,	126,
27.8635,	130,
27.8829,	134,

27.9024,	138,
27.9219,	144,
27.9413,	151,
27.9608,	109,
27.9802,	128,
27.9997,	120,
28.0191,	146,
28.0386,	127,
28.0581,	160,
28.0775,	145,
28.097,	140,
28.1164,	132,
28.1359,	135,
28.1553,	127,
28.1748,	150,
28.1943,	140,
28.2137,	153,
28.2332,	148,
28.2526,	128,
28.2721,	120,
28.2916,	144,
28.311,	128,
28.3305,	137,
28.3499,	142,
28.3694,	129,
28.3888,	135,
28.4083,	131,

28.4278, 160,

28.4472, 129,

28.4667, 152,

28.4861, 130,

28.5056, 141,

28.5251, 141,

28.5445, 132,

28.564, 144,

28.5834, 152,

28.6029, 138,

28.6223, 128,

28.6418, 132,

28.6613, 135,

28.6807, 130,

28.7002, 130,

28.7196, 134,

28.7391, 138,

28.7586, 129,

28.778, 141,

28.7975, 143,

28.8169, 135,

28.8364, 136,

28.8558, 121,

28.8753, 121,

28.8948, 145,

28.9142, 135,

28.9337, 138,

28.9531,	142,
28.9726,	129,
28.9921,	142,
29.0115,	156,
29.031,	144,
29.0504,	143,
29.0699,	120,
29.0893,	140,
29.1088,	145,
29.1283,	127,
29.1477,	143,
29.1672,	157,
29.1866,	156,
29.2061,	144,
29.2256,	140,
29.245,	129,
29.2645,	138,
29.2839,	125,
29.3034,	131,
29.3228,	130,
29.3423,	137,
29.3618,	133,
29.3812,	129,
29.4007,	161,
29.4201,	156,
29.4396,	147,
29.4591,	146,

29.4785,	151,
29.498,	148,
29.5174,	138,
29.5369,	140,
29.5563,	146,
29.5758,	130,
29.5953,	126,
29.6147,	118,
29.6342,	166,
29.6536,	159,
29.6731,	140,
29.6926,	142,
29.712,	122,
29.7315,	130,
29.7509,	153,
29.7704,	118,
29.7898,	140,
29.8093,	117,
29.8288,	132,
29.8482,	157,
29.8677,	132,
29.8871,	146,
29.9066,	133,
29.9261,	127,
29.9455,	142,
29.965,	150,
29.9844,	152,

30.0039, 126,

30.0233, 123,

30.0428, 142,

30.0623, 115,

30.0817, 126,

30.1012, 138,

30.1206, 131,

30.1401, 143,

30.1596, 130,

30.179, 133,

30.1985, 158,

30.2179, 129,

30.2374, 134,

30.2568, 168,

30.2763, 151,

30.2958, 136,

30.3152, 140,

30.3347, 155,

30.3541, 148,

30.3736, 146,

30.3931, 150,

30.4125, 133,

30.432, 133,

30.4514, 159,

30.4709, 145,

30.4903, 128,

30.5098, 155,

30.5293, 148,

30.5487, 143,

30.5682, 148,

30.5876, 140,

30.6071, 137,

30.6266, 165,

30.646, 153,

30.6655, 134,

30.6849, 153,

30.7044, 145,

30.7238, 160,

30.7433, 127,

30.7628, 139,

30.7822, 130,

30.8017, 147,

30.8211, 145,

30.8406, 141,

30.8601, 143,

30.8795, 131,

30.899, 155,

30.9184, 126,

30.9379, 132,

30.9573, 127,

30.9768, 119,

30.9963, 142,

31.0157, 145,

31.0352, 137,

31.0546,	142,
31.0741,	129,
31.0935,	144,
31.113,	147,
31.1325,	151,
31.1519,	136,
31.1714,	168,
31.1908,	145,
31.2103,	116,
31.2298,	143,
31.2492,	124,
31.2687,	178,
31.2881,	146,
31.3076,	148,
31.327,	132,
31.3465,	163,
31.366,	135,
31.3854,	161,
31.4049,	145,
31.4243,	121,
31.4438,	147,
31.4633,	130,
31.4827,	133,
31.5022,	130,
31.5216,	157,
31.5411,	144,
31.5605,	139,

31.58, 138,

31.5995, 145,

31.6189, 122,

31.6384, 135,

31.6578, 134,

31.6773, 149,

31.6968, 136,

31.7162, 163,

31.7357, 141,

31.7551, 159,

31.7746, 170,

31.794, 167,

31.8135, 154,

31.833, 152,

31.8524, 149,

31.8719, 160,

31.8913, 132,

31.9108, 132,

31.9303, 132,

31.9497, 146,

31.9692, 150,

31.9886, 133,

32.0081, 145,

32.0275, 130,

32.047, 156,

32.0665, 139,

32.0859, 150,

32.1054,	125,
32.1248,	131,
32.1443,	148,
32.1638,	161,
32.1832,	143,
32.2027,	144,
32.2221,	137,
32.2416,	153,
32.261,	149,
32.2805,	155,
32.3,	144,
32.3194,	160,
32.3389,	138,
32.3583,	113,
32.3778,	141,
32.3973,	136,
32.4167,	161,
32.4362,	146,
32.4556,	137,
32.4751,	126,
32.4945,	139,
32.514,	131,
32.5335,	141,
32.5529,	156,
32.5724,	170,
32.5918,	153,
32.6113,	131,

32.6308,	127,
32.6502,	130,
32.6697,	133,
32.6891,	160,
32.7086,	156,
32.728,	144,
32.7475,	121,
32.767,	149,
32.7864,	132,
32.8059,	141,
32.8253,	133,
32.8448,	157,
32.8643,	140,
32.8837,	143,
32.9032,	143,
32.9226,	163,
32.9421,	129,
32.9615,	133,
32.981,	150,
33.0005,	131,
33.0199,	136,
33.0394,	112,
33.0588,	137,
33.0783,	149,
33.0978,	138,
33.1172,	179,
33.1367,	147,

33.1561, 162,

33.1756, 162,

33.195, 133,

33.2145, 134,

33.234, 150,

33.2534, 143,

33.2729, 130,

33.2923, 144,

33.3118, 147,

33.3313, 132,

33.3507, 159,

33.3702, 147,

33.3896, 117,

33.4091, 157,

33.4285, 134,

33.448, 134,

33.4675, 146,

33.4869, 155,

33.5064, 140,

33.5258, 148,

33.5453, 146,

33.5648, 143,

33.5842, 144,

33.6037, 140,

33.6231, 116,

33.6426, 142,

33.662, 140,

33.6815,	153,
33.701,	165,
33.7204,	129,
33.7399,	150,
33.7593,	148,
33.7788,	139,
33.7983,	143,
33.8177,	165,
33.8372,	157,
33.8566,	155,
33.8761,	141,
33.8955,	135,
33.915,	163,
33.9345,	144,
33.9539,	149,
33.9734,	157,
33.9928,	126,
34.0123,	150,
34.0317,	139,
34.0512,	153,
34.0707,	132,
34.0901,	130,
34.1096,	162,
34.129,	141,
34.1485,	149,
34.168,	164,
34.1874,	132,

34.2069,	165,
34.2263,	160,
34.2458,	161,
34.2652,	153,
34.2847,	139,
34.3042,	160,
34.3236,	130,
34.3431,	130,
34.3625,	149,
34.382,	129,
34.4015,	134,
34.4209,	128,
34.4404,	136,
34.4598,	144,
34.4793,	152,
34.4987,	145,
34.5182,	161,
34.5377,	155,
34.5571,	138,
34.5766,	140,
34.596,	138,
34.6155,	148,
34.635,	148,
34.6544,	175,
34.6739,	148,
34.6933,	167,
34.7128,	127,

34.7322,	137,
34.7517,	150,
34.7712,	144,
34.7906,	139,
34.8101,	153,
34.8295,	134,
34.849,	154,
34.8685,	140,
34.8879,	138,
34.9074,	137,
34.9268,	152,
34.9463,	139,
34.9657,	166,
34.9852,	145,
35.0047,	147,
35.0241,	129,
35.0436,	154,
35.063,	148,
35.0825,	168,
35.102,	139,
35.1214,	142,
35.1409,	147,
35.1603,	166,
35.1798,	130,
35.1992,	144,
35.2187,	146,
35.2382,	145,

35.2576,	134,
35.2771,	138,
35.2965,	143,
35.316,	139,
35.3355,	152,
35.3549,	156,
35.3744,	152,
35.3938,	153,
35.4133,	147,
35.4327,	152,
35.4522,	162,
35.4717,	158,
35.4911,	168,
35.5106,	169,
35.53,	154,
35.5495,	151,
35.569,	150,
35.5884,	154,
35.6079,	138,
35.6273,	147,
35.6468,	144,
35.6662,	159,
35.6857,	157,
35.7052,	131,
35.7246,	161,
35.7441,	168,
35.7635,	150,

35.783, 164,

35.8025, 159,

35.8219, 144,

35.8414, 145,

35.8608, 164,

35.8803, 152,

35.8997, 150,

35.9192, 169,

35.9387, 166,

35.9581, 175,

35.9776, 150,

35.997, 142,

36.0165, 164,

36.036, 140,

36.0554, 155,

36.0749, 121,

36.0943, 142,

36.1138, 142,

36.1332, 152,

36.1527, 136,

36.1722, 169,

36.1916, 168,

36.2111, 152,

36.2305, 140,

36.25, 149,

36.2695, 143,

36.2889, 143,

36.3084, 140,

36.3278, 174,

36.3473, 173,

36.3667, 147,

36.3862, 153,

36.4057, 168,

36.4251, 154,

36.4446, 141,

36.464, 172,

36.4835, 153,

36.503, 138,

36.5224, 141,

36.5419, 138,

36.5613, 122,

36.5808, 161,

36.6002, 165,

36.6197, 147,

36.6392, 159,

36.6586, 145,

36.6781, 146,

36.6975, 135,

36.717, 173,

36.7365, 187,

36.7559, 154,

36.7754, 155,

36.7948, 143,

36.8143, 163,

36.8337,	162,
36.8532,	170,
36.8727,	165,
36.8921,	154,
36.9116,	156,
36.931,	182,
36.9505,	161,
36.9699,	173,
36.9894,	155,
37.0089,	147,
37.0283,	150,
37.0478,	148,
37.0672,	146,
37.0867,	136,
37.1062,	143,
37.1256,	171,
37.1451,	165,
37.1645,	148,
37.184,	153,
37.2034,	142,
37.2229,	144,
37.2424,	131,
37.2618,	155,
37.2813,	123,
37.3007,	157,
37.3202,	160,
37.3397,	118,

37.3591,	135,
37.3786,	125,
37.398,	141,
37.4175,	124,
37.4369,	162,
37.4564,	145,
37.4759,	154,
37.4953,	142,
37.5148,	152,
37.5342,	154,
37.5537,	141,
37.5732,	143,
37.5926,	126,
37.6121,	155,
37.6315,	136,
37.651,	156,
37.6704,	134,
37.6899,	155,
37.7094,	167,
37.7288,	166,
37.7483,	133,
37.7677,	145,
37.7872,	152,
37.8067,	151,
37.8261,	146,
37.8456,	153,
37.865,	142,

37.8845,	134,
37.9039,	172,
37.9234,	139,
37.9429,	122,
37.9623,	143,
37.9818,	141,
38.0012,	149,
38.0207,	153,
38.0402,	164,
38.0596,	152,
38.0791,	161,
38.0985,	134,
38.118,	159,
38.1374,	149,
38.1569,	127,
38.1764,	152,
38.1958,	151,
38.2153,	155,
38.2347,	145,
38.2542,	154,
38.2737,	153,
38.2931,	122,
38.3126,	137,
38.332,	130,
38.3515,	145,
38.3709,	140,
38.3904,	145,

38.4099,	136,
38.4293,	128,
38.4488,	160,
38.4682,	153,
38.4877,	162,
38.5072,	131,
38.5266,	135,
38.5461,	157,
38.5655,	143,
38.585,	147,
38.6044,	123,
38.6239,	148,
38.6434,	115,
38.6628,	158,
38.6823,	174,
38.7017,	145,
38.7212,	128,
38.7407,	149,
38.7601,	147,
38.7796,	141,
38.799,	137,
38.8185,	132,
38.8379,	139,
38.8574,	137,
38.8769,	144,
38.8963,	157,
38.9158,	149,

38.9352,	158,
38.9547,	163,
38.9742,	139,
38.9936,	150,
39.0131,	153,
39.0325,	137,
39.052,	150,
39.0714,	143,
39.0909,	120,
39.1104,	140,
39.1298,	154,
39.1493,	142,
39.1687,	158,
39.1882,	158,
39.2077,	143,
39.2271,	126,
39.2466,	146,
39.266,	141,
39.2855,	152,
39.3049,	138,
39.3244,	131,
39.3439,	140,
39.3633,	138,
39.3828,	154,
39.4022,	130,
39.4217,	141,
39.4412,	130,

39.4606, 150,

39.4801, 165,

39.4995, 156,

39.519, 140,

39.5384, 160,

39.5579, 153,

39.5774, 149,

39.5968, 146,

39.6163, 163,

39.6357, 139,

39.6552, 142,

39.6746, 161,

39.6941, 152,

39.7136, 143,

39.733, 134,

39.7525, 167,

39.7719, 128,

39.7914, 148,

39.8109, 159,

39.8303, 163,

39.8498, 146,

39.8692, 175,

39.8887, 153,

39.9081, 139,

39.9276, 177,

39.9471, 153,

39.9665, 160,

39.986,	138,
40.0054,	166,
40.0249,	141,
40.0444,	140,
40.0638,	167,
40.0833,	163,
40.1027,	153,
40.1222,	163,
40.1416,	174,
40.1611,	154,
40.1806,	138,
40.2,	150,
40.2195,	151,
40.2389,	142,
40.2584,	136,
40.2779,	170,
40.2973,	140,
40.3168,	143,
40.3362,	146,
40.3557,	165,
40.3751,	150,
40.3946,	153,
40.4141,	138,
40.4335,	148,
40.453,	164,
40.4724,	151,
40.4919,	149,

40.5114,	138,
40.5308,	153,
40.5503,	153,
40.5697,	182,
40.5892,	154,
40.6086,	140,
40.6281,	132,
40.6476,	131,
40.667,	152,
40.6865,	142,
40.7059,	146,
40.7254,	154,
40.7449,	136,
40.7643,	135,
40.7838,	149,
40.8032,	142,
40.8227,	170,
40.8421,	178,
40.8616,	166,
40.8811,	169,
40.9005,	140,
40.92,	151,
40.9394,	140,
40.9589,	141,
40.9784,	152,
40.9978,	156,
41.0173,	147,

41.0367, 167,

41.0562, 154,

41.0756, 134,

41.0951, 166,

41.1146, 163,

41.134, 145,

41.1535, 146,

41.1729, 141,

41.1924, 165,

41.2119, 134,

41.2313, 155,

41.2508, 138,

41.2702, 140,

41.2897, 164,

41.3091, 155,

41.3286, 159,

41.3481, 150,

41.3675, 139,

41.387, 167,

41.4064, 149,

41.4259, 158,

41.4454, 141,

41.4648, 143,

41.4843, 143,

41.5037, 136,

41.5232, 145,

41.5426, 157,

41.5621,	146,
41.5816,	155,
41.601,	132,
41.6205,	152,
41.6399,	126,
41.6594,	172,
41.6789,	163,
41.6983,	143,
41.7178,	139,
41.7372,	151,
41.7567,	172,
41.7761,	133,
41.7956,	144,
41.8151,	162,
41.8345,	151,
41.854,	156,
41.8734,	128,
41.8929,	157,
41.9124,	148,
41.9318,	182,
41.9513,	151,
41.9707,	151,
41.9902,	154,
42.0096,	147,
42.0291,	141,
42.0486,	153,
42.068,	147,

42.0875,	120,
42.1069,	146,
42.1264,	176,
42.1459,	168,
42.1653,	150,
42.1848,	141,
42.2042,	148,
42.2237,	152,
42.2431,	143,
42.2626,	171,
42.2821,	148,
42.3015,	148,
42.321,	139,
42.3404,	153,
42.3599,	133,
42.3794,	143,
42.3988,	159,
42.4183,	139,
42.4377,	147,
42.4572,	147,
42.4766,	150,
42.4961,	134,
42.5156,	148,
42.535,	154,
42.5545,	139,
42.5739,	155,
42.5934,	140,

42.6128,	161,
42.6323,	131,
42.6518,	140,
42.6712,	161,
42.6907,	156,
42.7101,	160,
42.7296,	133,
42.7491,	151,
42.7685,	150,
42.788,	141,
42.8074,	157,
42.8269,	154,
42.8463,	155,
42.8658,	159,
42.8853,	159,
42.9047,	135,
42.9242,	147,
42.9436,	160,
42.9631,	148,
42.9826,	139,
43.002,	158,
43.0215,	163,
43.0409,	156,
43.0604,	141,
43.0798,	131,
43.0993,	145,
43.1188,	177,

43.1382, 149,

43.1577, 147,

43.1771, 183,

43.1966, 150,

43.2161, 142,

43.2355, 139,

43.255, 158,

43.2744, 162,

43.2939, 145,

43.3133, 149,

43.3328, 161,

43.3523, 125,

43.3717, 153,

43.3912, 141,

43.4106, 166,

43.4301, 135,

43.4496, 161,

43.469, 138,

43.4885, 159,

43.5079, 147,

43.5274, 164,

43.5468, 158,

43.5663, 157,

43.5858, 151,

43.6052, 151,

43.6247, 184,

43.6441, 159,

43.6636,	139,
43.6831,	154,
43.7025,	162,
43.722,	138,
43.7414,	164,
43.7609,	154,
43.7803,	149,
43.7998,	147,
43.8193,	172,
43.8387,	148,
43.8582,	154,
43.8776,	154,
43.8971,	147,
43.9166,	147,
43.936,	155,
43.9555,	136,
43.9749,	169,
43.9944,	145,
44.0138,	148,
44.0333,	150,
44.0528,	166,
44.0722,	138,
44.0917,	160,
44.1111,	151,
44.1306,	145,
44.1501,	135,
44.1695,	154,

44.189,	160,
44.2084,	146,
44.2279,	144,
44.2473,	139,
44.2668,	149,
44.2863,	151,
44.3057,	141,
44.3252,	146,
44.3446,	171,
44.3641,	145,
44.3836,	131,
44.403,	164,
44.4225,	151,
44.4419,	133,
44.4614,	151,
44.4808,	140,
44.5003,	160,
44.5198,	160,
44.5392,	137,
44.5587,	157,
44.5781,	145,
44.5976,	163,
44.6171,	155,
44.6365,	145,
44.656,	140,
44.6754,	154,
44.6949,	167,

44.7143, 149,

44.7338, 122,

44.7533, 155,

44.7727, 129,

44.7922, 145,

44.8116, 144,

44.8311, 159,

44.8506, 146,

44.87, 148,

44.8895, 157,

44.9089, 141,

44.9284, 160,

44.9478, 113,

44.9673, 148,

44.9868, 166,

45.0062, 162,

45.0257, 146,

45.0451, 160,

45.0646, 159,

45.0841, 160,

45.1035, 148,

45.123, 146,

45.1424, 134,

45.1619, 148,

45.1813, 160,

45.2008, 155,

45.2203, 164,

45.2397,	158,
45.2592,	149,
45.2786,	157,
45.2981,	125,
45.3176,	163,
45.337,	167,
45.3565,	138,
45.3759,	162,
45.3954,	161,
45.4148,	175,
45.4343,	161,
45.4538,	170,
45.4732,	166,
45.4927,	168,
45.5121,	180,
45.5316,	172,
45.551,	162,
45.5705,	180,
45.59,	157,
45.6094,	160,
45.6289,	147,
45.6483,	143,
45.6678,	150,
45.6873,	166,
45.7067,	141,
45.7262,	156,
45.7456,	146,

45.7651,	157,
45.7845,	137,
45.804,	153,
45.8235,	141,
45.8429,	135,
45.8624,	132,
45.8818,	148,
45.9013,	168,
45.9208,	163,
45.9402,	168,
45.9597,	135,
45.9791,	128,
45.9986,	145,
46.018,	167,
46.0375,	156,
46.057,	161,
46.0764,	139,
46.0959,	156,
46.1153,	152,
46.1348,	127,
46.1543,	157,
46.1737,	143,
46.1932,	180,
46.2126,	139,
46.2321,	159,
46.2515,	142,
46.271,	159,

46.2905, 173,

46.3099, 168,

46.3294, 167,

46.3488, 151,

46.3683, 149,

46.3878, 139,

46.4072, 147,

46.4267, 143,

46.4461, 147,

46.4656, 147,

46.485, 179,

46.5045, 142,

46.524, 147,

46.5434, 156,

46.5629, 161,

46.5823, 151,

46.6018, 143,

46.6213, 165,

46.6407, 132,

46.6602, 142,

46.6796, 145,

46.6991, 128,

46.7185, 153,

46.738, 168,

46.7575, 152,

46.7769, 162,

46.7964, 157,

46.8158,	165,
46.8353,	156,
46.8548,	160,
46.8742,	145,
46.8937,	143,
46.9131,	154,
46.9326,	144,
46.952,	143,
46.9715,	161,
46.991,	149,
47.0104,	136,
47.0299,	157,
47.0493,	164,
47.0688,	143,
47.0883,	157,
47.1077,	153,
47.1272,	156,
47.1466,	145,
47.1661,	165,
47.1855,	163,
47.205,	143,
47.2245,	144,
47.2439,	152,
47.2634,	173,
47.2828,	161,
47.3023,	145,
47.3218,	161,

47.3412, 163,

47.3607, 163,

47.3801, 153,

47.3996, 137,

47.419, 133,

47.4385, 137,

47.458, 166,

47.4774, 135,

47.4969, 142,

47.5163, 144,

47.5358, 162,

47.5553, 150,

47.5747, 167,

47.5942, 144,

47.6136, 152,

47.6331, 130,

47.6525, 136,

47.672, 153,

47.6915, 144,

47.7109, 143,

47.7304, 159,

47.7498, 150,

47.7693, 171,

47.7888, 135,

47.8082, 155,

47.8277, 141,

47.8471, 173,

47.8666,	164,
47.886,	154,
47.9055,	152,
47.925,	143,
47.9444,	150,
47.9639,	147,
47.9833,	139,
48.0028,	147,
48.0223,	150,
48.0417,	162,
48.0612,	138,
48.0806,	159,
48.1001,	139,
48.1195,	160,
48.139,	142,
48.1585,	126,
48.1779,	138,
48.1974,	152,
48.2168,	163,
48.2363,	159,
48.2558,	143,
48.2752,	151,
48.2947,	157,
48.3141,	167,
48.3336,	150,
48.353,	134,
48.3725,	177,

48.392,	156,
48.4114,	151,
48.4309,	167,
48.4503,	162,
48.4698,	176,
48.4892,	157,
48.5087,	138,
48.5282,	148,
48.5476,	176,
48.5671,	164,
48.5865,	163,
48.606,	138,
48.6255,	161,
48.6449,	153,
48.6644,	155,
48.6838,	147,
48.7033,	175,
48.7227,	144,
48.7422,	156,
48.7617,	169,
48.7811,	136,
48.8006,	163,
48.82,	142,
48.8395,	141,
48.859,	146,
48.8784,	140,
48.8979,	136,

48.9173,	144,
48.9368,	128,
48.9562,	158,
48.9757,	178,
48.9952,	140,
49.0146,	171,
49.0341,	165,
49.0535,	160,
49.073,	133,
49.0925,	148,
49.1119,	153,
49.1314,	131,
49.1508,	141,
49.1703,	159,
49.1897,	143,
49.2092,	159,
49.2287,	156,
49.2481,	161,
49.2676,	117,
49.287,	153,
49.3065,	166,
49.326,	149,
49.3454,	128,
49.3649,	129,
49.3843,	163,
49.4038,	134,
49.4232,	141,

49.4427, 158,

49.4622, 148,

49.4816, 162,

49.5011, 150,

49.5205, 167,

49.54, 152,

49.5595, 163,

49.5789, 137,

49.5984, 150,

49.6178, 168,

49.6373, 150,

49.6567, 153,

49.6762, 147,

49.6957, 155,

49.7151, 181,

49.7346, 162,

49.754, 172,

49.7735, 128,

49.793, 158,

49.8124, 151,

49.8319, 163,

49.8513, 151,

49.8708, 172,

49.8902, 168,

49.9097, 158,

49.9292, 168,

49.9486, 147,

49.9681,	164,
49.9875,	155,
50.007,	142,
50.0265,	144,
50.0459,	145,
50.0654,	144,
50.0848,	160,
50.1043,	166,
50.1237,	136,
50.1432,	162,
50.1627,	153,
50.1821,	166,
50.2016,	178,
50.221,	150,
50.2405,	165,
50.26,	143,
50.2794,	141,
50.2989,	153,
50.3183,	170,
50.3378,	163,
50.3572,	161,
50.3767,	171,
50.3962,	143,
50.4156,	169,
50.4351,	150,
50.4545,	151,
50.474,	159,

50.4935,	135,
50.5129,	152,
50.5324,	172,
50.5518,	138,
50.5713,	173,
50.5907,	169,
50.6102,	159,
50.6297,	146,
50.6491,	175,
50.6686,	179,
50.688,	155,
50.7075,	136,
50.727,	162,
50.7464,	184,
50.7659,	175,
50.7853,	177,
50.8048,	158,
50.8242,	148,
50.8437,	146,
50.8632,	156,
50.8826,	155,
50.9021,	167,
50.9215,	152,
50.941,	147,
50.9605,	168,
50.9799,	152,
50.9994,	160,

51.0188,	164,
51.0383,	170,
51.0577,	148,
51.0772,	165,
51.0967,	121,
51.1161,	161,
51.1356,	147,
51.155,	165,
51.1745,	166,
51.1939,	171,
51.2134,	174,
51.2329,	162,
51.2523,	158,
51.2718,	176,
51.2912,	152,
51.3107,	155,
51.3302,	152,
51.3496,	148,
51.3691,	160,
51.3885,	154,
51.408,	162,
51.4274,	157,
51.4469,	144,
51.4664,	169,
51.4858,	153,
51.5053,	163,
51.5247,	140,

51.5442,	175,
51.5637,	168,
51.5831,	150,
51.6026,	136,
51.622,	173,
51.6415,	162,
51.6609,	147,
51.6804,	167,
51.6999,	146,
51.7193,	165,
51.7388,	151,
51.7582,	147,
51.7777,	154,
51.7972,	166,
51.8166,	154,
51.8361,	128,
51.8555,	162,
51.875,	161,
51.8944,	150,
51.9139,	141,
51.9334,	171,
51.9528,	156,
51.9723,	146,
51.9917,	162,
52.0112,	166,
52.0307,	150,
52.0501,	151,

52.0696,	151,
52.089,	155,
52.1085,	160,
52.1279,	158,
52.1474,	165,
52.1669,	173,
52.1863,	152,
52.2058,	152,
52.2252,	189,
52.2447,	149,
52.2642,	154,
52.2836,	166,
52.3031,	153,
52.3225,	156,
52.342,	156,
52.3614,	164,
52.3809,	177,
52.4004,	135,
52.4198,	150,
52.4393,	139,
52.4587,	157,
52.4782,	146,
52.4977,	141,
52.5171,	156,
52.5366,	167,
52.556,	128,
52.5755,	147,

52.5949,	164,
52.6144,	179,
52.6339,	152,
52.6533,	151,
52.6728,	128,
52.6922,	168,
52.7117,	156,
52.7312,	156,
52.7506,	168,
52.7701,	143,
52.7895,	150,
52.809,	161,
52.8284,	140,
52.8479,	157,
52.8674,	163,
52.8868,	160,
52.9063,	161,
52.9257,	159,
52.9452,	179,
52.9647,	153,
52.9841,	161,
53.0036,	172,
53.023,	178,
53.0425,	151,
53.0619,	164,
53.0814,	142,
53.1009,	149,

53.1203,	158,
53.1398,	171,
53.1592,	141,
53.1787,	171,
53.1982,	168,
53.2176,	148,
53.2371,	158,
53.2565,	129,
53.276,	151,
53.2954,	161,
53.3149,	167,
53.3344,	176,
53.3538,	143,
53.3733,	169,
53.3927,	162,
53.4122,	193,
53.4317,	161,
53.4511,	190,
53.4706,	171,
53.49,	156,
53.5095,	162,
53.5289,	150,
53.5484,	154,
53.5679,	169,
53.5873,	138,
53.6068,	153,
53.6262,	148,

53.6457,	163,
53.6652,	153,
53.6846,	155,
53.7041,	147,
53.7235,	154,
53.743,	151,
53.7624,	146,
53.7819,	178,
53.8014,	171,
53.8208,	153,
53.8403,	158,
53.8597,	150,
53.8792,	159,
53.8987,	157,
53.9181,	154,
53.9376,	150,
53.957,	166,
53.9765,	158,
53.9959,	155,
54.0154,	164,
54.0349,	158,
54.0543,	158,
54.0738,	171,
54.0932,	151,
54.1127,	168,
54.1321,	149,
54.1516,	162,

54.1711, 169,

54.1905, 174,

54.21, 139,

54.2294, 164,

54.2489, 169,

54.2684, 175,

54.2878, 155,

54.3073, 167,

54.3267, 172,

54.3462, 155,

54.3656, 154,

54.3851, 149,

54.4046, 167,

54.424, 150,

54.4435, 169,

54.4629, 151,

54.4824, 153,

54.5019, 178,

54.5213, 154,

54.5408, 154,

54.5602, 185,

54.5797, 155,

54.5991, 148,

54.6186, 149,

54.6381, 134,

54.6575, 165,

54.677, 155,

54.6964,	143,
54.7159,	164,
54.7354,	148,
54.7548,	168,
54.7743,	171,
54.7937,	146,
54.8132,	160,
54.8326,	142,
54.8521,	188,
54.8716,	164,
54.891,	157,
54.9105,	163,
54.9299,	172,
54.9494,	171,
54.9689,	168,
54.9883,	162,
55.0078,	164,
55.0272,	160,
55.0467,	157,
55.0661,	167,
55.0856,	156,
55.1051,	162,
55.1245,	144,
55.144,	148,
55.1634,	154,
55.1829,	147,
55.2024,	151,

55.2218,	150,
55.2413,	154,
55.2607,	168,
55.2802,	179,
55.2996,	155,
55.3191,	166,
55.3386,	170,
55.358,	156,
55.3775,	161,
55.3969,	163,
55.4164,	164,
55.4359,	159,
55.4553,	175,
55.4748,	135,
55.4942,	163,
55.5137,	160,
55.5331,	163,
55.5526,	167,
55.5721,	174,
55.5915,	154,
55.611,	151,
55.6304,	151,
55.6499,	174,
55.6694,	157,
55.6888,	162,
55.7083,	146,
55.7277,	172,

55.7472,	152,
55.7666,	166,
55.7861,	143,
55.8056,	166,
55.825,	174,
55.8445,	170,
55.8639,	169,
55.8834,	159,
55.9029,	152,
55.9223,	154,
55.9418,	175,
55.9612,	169,
55.9807,	173,
56.0001,	157,
56.0196,	148,
56.0391,	151,
56.0585,	154,
56.078,	167,
56.0974,	155,
56.1169,	170,
56.1364,	177,
56.1558,	166,
56.1753,	167,
56.1947,	162,
56.2142,	163,
56.2336,	164,
56.2531,	162,

56.2726,	173,
56.292,	158,
56.3115,	144,
56.3309,	165,
56.3504,	156,
56.3699,	150,
56.3893,	156,
56.4088,	179,
56.4282,	171,
56.4477,	174,
56.4671,	167,
56.4866,	172,
56.5061,	168,
56.5255,	152,
56.545,	150,
56.5644,	162,
56.5839,	168,
56.6034,	165,
56.6228,	167,
56.6423,	148,
56.6617,	159,
56.6812,	178,
56.7006,	165,
56.7201,	163,
56.7396,	157,
56.759,	166,
56.7785,	162,

56.7979, 172,

56.8174, 157,

56.8369, 155,

56.8563, 166,

56.8758, 174,

56.8952, 152,

56.9147, 166,

56.9341, 161,

56.9536, 193,

56.9731, 145,

56.9925, 171,

57.012, 143,

57.0314, 156,

57.0509, 177,

57.0703, 157,

57.0898, 172,

57.1093, 160,

57.1287, 157,

57.1482, 158,

57.1676, 152,

57.1871, 168,

57.2066, 200,

57.226, 168,

57.2455, 160,

57.2649, 173,

57.2844, 161,

57.3038, 157,

57.3233, 152,

57.3428, 140,

57.3622, 155,

57.3817, 147,

57.4011, 192,

57.4206, 168,

57.4401, 152,

57.4595, 174,

57.479, 150,

57.4984, 153,

57.5179, 156,

57.5373, 148,

57.5568, 172,

57.5763, 169,

57.5957, 180,

57.6152, 175,

57.6346, 151,

57.6541, 153,

57.6736, 158,

57.693, 162,

57.7125, 150,

57.7319, 154,

57.7514, 169,

57.7708, 148,

57.7903, 163,

57.8098, 178,

57.8292, 164,

57.8487,	158,
57.8681,	146,
57.8876,	147,
57.9071,	170,
57.9265,	163,
57.946,	148,
57.9654,	138,
57.9849,	187,
58.0043,	157,
58.0238,	188,
58.0433,	153,
58.0627,	169,
58.0822,	153,
58.1016,	147,
58.1211,	180,
58.1406,	145,
58.16,	177,
58.1795,	164,
58.1989,	156,
58.2184,	168,
58.2378,	164,
58.2573,	188,
58.2768,	153,
58.2962,	158,
58.3157,	168,
58.3351,	159,
58.3546,	195,

58.3741,	156,
58.3935,	145,
58.413,	174,
58.4324,	165,
58.4519,	162,
58.4713,	131,
58.4908,	168,
58.5103,	149,
58.5297,	161,
58.5492,	179,
58.5686,	136,
58.5881,	152,
58.6076,	170,
58.627,	158,
58.6465,	158,
58.6659,	165,
58.6854,	169,
58.7048,	155,
58.7243,	187,
58.7438,	178,
58.7632,	170,
58.7827,	159,
58.8021,	156,
58.8216,	198,
58.8411,	196,
58.8605,	191,
58.88,	169,

58.8994,	181,
58.9189,	151,
58.9383,	169,
58.9578,	176,
58.9773,	152,
58.9967,	202,
59.0162,	184,
59.0356,	163,
59.0551,	168,
59.0746,	162,
59.094,	148,
59.1135,	175,
59.1329,	159,
59.1524,	161,
59.1718,	161,
59.1913,	171,
59.2108,	181,
59.2302,	160,
59.2497,	171,
59.2691,	156,
59.2886,	139,
59.3081,	184,
59.3275,	169,
59.347,	194,
59.3664,	130,
59.3859,	158,
59.4053,	179,

59.4248,	160,
59.4443,	151,
59.4637,	157,
59.4832,	154,
59.5026,	188,
59.5221,	156,
59.5416,	181,
59.561,	147,
59.5805,	165,
59.5999,	151,
59.6194,	143,
59.6388,	166,
59.6583,	148,
59.6778,	160,
59.6972,	128,
59.7167,	184,
59.7361,	137,
59.7556,	139,
59.7751,	146,
59.7945,	153,
59.814,	182,
59.8334,	165,
59.8529,	183,
59.8723,	150,
59.8918,	142,
59.9113,	174,
59.9307,	142,

59.9502, 175,

59.9696, 145,

59.9891, 158,

60.0085, 169,

60.028, 165,

60.0475, 158,

60.0669, 185,

60.0864, 161,

60.1058, 170,

60.1253, 173,

60.1448, 170,

60.1642, 161,

60.1837, 168,

60.2031, 169,

60.2226, 151,

60.242, 185,

60.2615, 168,

60.281, 162,

60.3004, 161,

60.3199, 174,

60.3393, 149,

60.3588, 176,

60.3783, 134,

60.3977, 169,

60.4172, 180,

60.4366, 147,

60.4561, 168,

60.4755,	157,
60.495,	177,
60.5145,	167,
60.5339,	179,
60.5534,	154,
60.5728,	185,
60.5923,	166,
60.6118,	173,
60.6312,	160,
60.6507,	186,
60.6701,	178,
60.6896,	178,
60.709,	138,
60.7285,	174,
60.748,	146,
60.7674,	134,
60.7869,	176,
60.8063,	173,
60.8258,	155,
60.8453,	179,
60.8647,	167,
60.8842,	158,
60.9036,	164,
60.9231,	160,
60.9425,	195,
60.962,	171,
60.9815,	163,

61.0009,	164,
61.0204,	146,
61.0398,	188,
61.0593,	193,
61.0788,	186,
61.0982,	179,
61.1177,	200,
61.1371,	162,
61.1566,	172,
61.176,	177,
61.1955,	160,
61.215,	165,
61.2344,	165,
61.2539,	173,
61.2733,	159,
61.2928,	158,
61.3123,	172,
61.3317,	182,
61.3512,	172,
61.3706,	141,
61.3901,	183,
61.4095,	188,
61.429,	152,
61.4485,	161,
61.4679,	166,
61.4874,	167,
61.5068,	154,

61.5263,	172,
61.5458,	182,
61.5652,	143,
61.5847,	182,
61.6041,	183,
61.6236,	175,
61.643,	200,
61.6625,	161,
61.682,	152,
61.7014,	143,
61.7209,	140,
61.7403,	158,
61.7598,	162,
61.7793,	159,
61.7987,	160,
61.8182,	163,
61.8376,	170,
61.8571,	160,
61.8765,	158,
61.896,	173,
61.9155,	166,
61.9349,	156,
61.9544,	146,
61.9738,	167,
61.9933,	142,
62.0128,	166,
62.0322,	160,

62.0517,	168,
62.0711,	175,
62.0906,	160,
62.11,	152,
62.1295,	180,
62.149,	177,
62.1684,	182,
62.1879,	175,
62.2073,	160,
62.2268,	159,
62.2463,	165,
62.2657,	159,
62.2852,	173,
62.3046,	185,
62.3241,	168,
62.3435,	173,
62.363,	163,
62.3825,	185,
62.4019,	146,
62.4214,	179,
62.4408,	181,
62.4603,	183,
62.4798,	163,
62.4992,	159,
62.5187,	182,
62.5381,	162,
62.5576,	153,

62.577,	161,
62.5965,	177,
62.616,	197,
62.6354,	159,
62.6549,	174,
62.6743,	166,
62.6938,	180,
62.7132,	169,
62.7327,	181,
62.7522,	193,
62.7716,	190,
62.7911,	199,
62.8105,	178,
62.83,	164,
62.8495,	191,
62.8689,	189,
62.8884,	195,
62.9078,	155,
62.9273,	164,
62.9467,	192,
62.9662,	155,
62.9857,	165,
63.0051,	181,
63.0246,	193,
63.044,	167,
63.0635,	173,
63.083,	188,

63.1024,	173,
63.1219,	148,
63.1413,	159,
63.1608,	155,
63.1802,	176,
63.1997,	172,
63.2192,	170,
63.2386,	172,
63.2581,	170,
63.2775,	176,
63.297,	159,
63.3165,	169,
63.3359,	164,
63.3554,	174,
63.3748,	177,
63.3943,	170,
63.4137,	171,
63.4332,	174,
63.4527,	196,
63.4721,	163,
63.4916,	166,
63.511,	172,
63.5305,	159,
63.55,	157,
63.5694,	198,
63.5889,	160,
63.6083,	145,

63.6278,	153,
63.6472,	172,
63.6667,	152,
63.6862,	175,
63.7056,	177,
63.7251,	146,
63.7445,	200,
63.764,	161,
63.7835,	176,
63.8029,	152,
63.8224,	167,
63.8418,	172,
63.8613,	176,
63.8807,	187,
63.9002,	168,
63.9197,	170,
63.9391,	152,
63.9586,	180,
63.978,	168,
63.9975,	173,
64.017,	185,
64.0364,	170,
64.0559,	164,
64.0753,	171,
64.0948,	184,
64.1142,	151,
64.1337,	164,

64.1532,	154,
64.1726,	170,
64.1921,	159,
64.2115,	179,
64.231,	173,
64.2505,	182,
64.2699,	166,
64.2894,	155,
64.3088,	175,
64.3283,	178,
64.3477,	168,
64.3672,	191,
64.3867,	148,
64.4061,	157,
64.4256,	147,
64.445,	178,
64.4645,	166,
64.484,	160,
64.5034,	145,
64.5229,	151,
64.5423,	182,
64.5618,	171,
64.5812,	166,
64.6007,	209,
64.6202,	175,
64.6396,	186,
64.6591,	186,

64.6785,	139,
64.698,	177,
64.7175,	178,
64.7369,	195,
64.7564,	157,
64.7758,	163,
64.7953,	174,
64.8147,	153,
64.8342,	161,
64.8537,	185,
64.8731,	144,
64.8926,	174,
64.912,	162,
64.9315,	160,
64.951,	166,
64.9704,	168,
64.9899,	175,
65.0093,	166,
65.0288,	184,
65.0482,	164,
65.0677,	195,
65.0872,	188,
65.1066,	174,
65.1261,	164,
65.1455,	167,
65.165,	164,
65.1845,	176,

65.2039, 167,

65.2234, 169,

65.2428, 160,

65.2623, 162,

65.2817, 163,

65.3012, 176,

65.3207, 152,

65.3401, 188,

65.3596, 160,

65.379, 147,

65.3985, 174,

65.418, 160,

65.4374, 156,

65.4569, 170,

65.4763, 195,

65.4958, 146,

65.5152, 160,

65.5347, 172,

65.5542, 175,

65.5736, 157,

65.5931, 179,

65.6125, 145,

65.632, 198,

65.6514, 171,

65.6709, 177,

65.6904, 188,

65.7098, 184,

65.7293, 145,

65.7487, 181,

65.7682, 164,

65.7877, 160,

65.8071, 172,

65.8266, 141,

65.846, 165,

65.8655, 175,

65.8849, 171,

65.9044, 175,

65.9239, 194,

65.9433, 165,

65.9628, 175,

65.9822, 161,

66.0017, 171,

66.0212, 165,

66.0406, 155,

66.0601, 145,

66.0795, 151,

66.099, 162,

66.1184, 167,

66.1379, 162,

66.1574, 166,

66.1768, 175,

66.1963, 147,

66.2157, 169,

66.2352, 177,

66.2547,	168,
66.2741,	164,
66.2936,	161,
66.313,	175,
66.3325,	166,
66.3519,	174,
66.3714,	161,
66.3909,	163,
66.4103,	168,
66.4298,	174,
66.4492,	184,
66.4687,	172,
66.4882,	166,
66.5076,	159,
66.5271,	143,
66.5465,	210,
66.566,	167,
66.5854,	159,
66.6049,	169,
66.6244,	177,
66.6438,	173,
66.6633,	145,
66.6827,	151,
66.7022,	175,
66.7217,	161,
66.7411,	175,
66.7606,	182,

66.78,	153,
66.7995,	148,
66.8189,	149,
66.8384,	166,
66.8579,	164,
66.8773,	140,
66.8968,	166,
66.9162,	170,
66.9357,	164,
66.9552,	176,
66.9746,	172,
66.9941,	155,
67.0135,	158,
67.033,	173,
67.0524,	171,
67.0719,	155,
67.0914,	179,
67.1108,	184,
67.1303,	198,
67.1497,	181,
67.1692,	184,
67.1887,	150,
67.2081,	172,
67.2276,	157,
67.247,	182,
67.2665,	199,
67.2859,	167,

67.3054,	161,
67.3249,	149,
67.3443,	168,
67.3638,	167,
67.3832,	177,
67.4027,	166,
67.4222,	179,
67.4416,	186,
67.4611,	184,
67.4805,	162,
67.5,	181,
67.5194,	174,
67.5389,	178,
67.5584,	182,
67.5778,	166,
67.5973,	171,
67.6167,	170,
67.6362,	163,
67.6557,	166,
67.6751,	177,
67.6946,	185,
67.714,	148,
67.7335,	131,
67.7529,	195,
67.7724,	168,
67.7919,	182,
67.8113,	176,

67.8308,	178,
67.8502,	155,
67.8697,	148,
67.8892,	180,
67.9086,	176,
67.9281,	150,
67.9475,	174,
67.967,	150,
67.9864,	170,
68.0059,	167,
68.0254,	166,
68.0448,	162,
68.0643,	195,
68.0837,	165,
68.1032,	188,
68.1227,	163,
68.1421,	167,
68.1616,	164,
68.181,	186,
68.2005,	154,
68.2199,	178,
68.2394,	173,
68.2589,	176,
68.2783,	170,
68.2978,	195,
68.3172,	162,
68.3367,	183,

68.3562,	178,
68.3756,	200,
68.3951,	182,
68.4145,	171,
68.434,	172,
68.4534,	178,
68.4729,	154,
68.4924,	163,
68.5118,	191,
68.5313,	182,
68.5507,	169,
68.5702,	189,
68.5896,	154,
68.6091,	170,
68.6286,	189,
68.648,	183,
68.6675,	146,
68.6869,	151,
68.7064,	167,
68.7259,	169,
68.7453,	158,
68.7648,	161,
68.7842,	183,
68.8037,	197,
68.8231,	177,
68.8426,	186,
68.8621,	147,

68.8815,	182,
68.901,	165,
68.9204,	169,
68.9399,	187,
68.9594,	178,
68.9788,	162,
68.9983,	173,
69.0177,	159,
69.0372,	164,
69.0566,	153,
69.0761,	144,
69.0956,	168,
69.115,	179,
69.1345,	171,
69.1539,	183,
69.1734,	166,
69.1929,	173,
69.2123,	131,
69.2318,	176,
69.2512,	164,
69.2707,	153,
69.2901,	156,
69.3096,	197,
69.3291,	176,
69.3485,	195,
69.368,	163,
69.3874,	180,

69.4069,	179,
69.4264,	145,
69.4458,	151,
69.4653,	148,
69.4847,	179,
69.5042,	169,
69.5236,	169,
69.5431,	168,
69.5626,	141,
69.582,	164,
69.6015,	166,
69.6209,	173,
69.6404,	168,
69.6599,	164,
69.6793,	179,
69.6988,	172,
69.7182,	159,
69.7377,	171,
69.7571,	138,
69.7766,	151,
69.7961,	163,
69.8155,	130,
69.835,	176,
69.8544,	163,
69.8739,	173,
69.8934,	180,
69.9128,	170,

69.9323,	183,
69.9517,	174,
69.9712,	154,
69.9906,	173,
70.0101,	173,
70.0296,	143,
70.049,	158,
70.0685,	165,
70.0879,	163,
70.1074,	182,
70.1269,	180,
70.1463,	151,
70.1658,	142,
70.1852,	165,
70.2047,	176,
70.2241,	167,
70.2436,	193,
70.2631,	175,
70.2825,	169,
70.302,	170,
70.3214,	173,
70.3409,	176,
70.3604,	165,
70.3798,	177,
70.3993,	168,
70.4187,	159,
70.4382,	148,

70.4576,	163,
70.4771,	165,
70.4966,	183,
70.516,	162,
70.5355,	191,
70.5549,	150,
70.5744,	152,
70.5939,	150,
70.6133,	168,
70.6328,	141,
70.6522,	182,
70.6717,	161,
70.6911,	175,
70.7106,	168,
70.7301,	151,
70.7495,	164,
70.769,	171,
70.7884,	165,
70.8079,	159,
70.8274,	157,
70.8468,	163,
70.8663,	180,
70.8857,	174,
70.9052,	182,
70.9246,	177,
70.9441,	178,
70.9636,	154,

70.983,	174,
71.0025,	159,
71.0219,	179,
71.0414,	163,
71.0609,	141,
71.0803,	202,
71.0998,	163,
71.1192,	159,
71.1387,	195,
71.1581,	159,
71.1776,	167,
71.1971,	156,
71.2165,	174,
71.236,	165,
71.2554,	175,
71.2749,	192,
71.2944,	159,
71.3138,	186,
71.3333,	193,
71.3527,	186,
71.3722,	175,
71.3916,	164,
71.4111,	168,
71.4306,	163,
71.45,	175,
71.4695,	173,
71.4889,	172,

71.5084, 201,

71.5278, 174,

71.5473, 187,

71.5668, 152,

71.5862, 179,

71.6057, 198,

71.6251, 181,

71.6446, 167,

71.6641, 157,

71.6835, 190,

71.703, 150,

71.7224, 187,

71.7419, 183,

71.7613, 172,

71.7808, 156,

71.8003, 177,

71.8197, 143,

71.8392, 147,

71.8586, 190,

71.8781, 195,

71.8976, 142,

71.917, 164,

71.9365, 154,

71.9559, 182,

71.9754, 162,

71.9948, 169,

72.0143, 174,

72.0338,	158,
72.0532,	152,
72.0727,	175,
72.0921,	162,
72.1116,	175,
72.1311,	178,
72.1505,	181,
72.17,	158,
72.1894,	169,
72.2089,	180,
72.2283,	178,
72.2478,	166,
72.2673,	168,
72.2867,	158,
72.3062,	178,
72.3256,	169,
72.3451,	172,
72.3646,	157,
72.384,	171,
72.4035,	183,
72.4229,	176,
72.4424,	168,
72.4618,	157,
72.4813,	157,
72.5008,	141,
72.5202,	155,
72.5397,	156,

72.5591, 163,

72.5786, 164,

72.5981, 165,

72.6175, 161,

72.637, 183,

72.6564, 166,

72.6759, 178,

72.6953, 160,

72.7148, 175,

72.7343, 186,

72.7537, 162,

72.7732, 157,

72.7926, 161,

72.8121, 158,

72.8316, 186,

72.851, 180,

72.8705, 182,

72.8899, 189,

72.9094, 156,

72.9288, 159,

72.9483, 168,

72.9678, 204,

72.9872, 166,

73.0067, 164,

73.0261, 165,

73.0456, 167,

73.0651, 177,

73.0845, 158,

73.104, 170,

73.1234, 163,

73.1429, 171,

73.1623, 163,

73.1818, 172,

73.2013, 162,

73.2207, 147,

73.2402, 149,

73.2596, 159,

73.2791, 153,

73.2986, 172,

73.318, 163,

73.3375, 165,

73.3569, 165,

73.3764, 165,

73.3958, 173,

73.4153, 177,

73.4348, 157,

73.4542, 179,

73.4737, 161,

73.4931, 150,

73.5126, 164,

73.5321, 172,

73.5515, 163,

73.571, 173,

73.5904, 173,

73.6099,	183,
73.6293,	168,
73.6488,	143,
73.6683,	158,
73.6877,	174,
73.7072,	176,
73.7266,	170,
73.7461,	159,
73.7656,	171,
73.785,	154,
73.8045,	173,
73.8239,	183,
73.8434,	167,
73.8628,	186,
73.8823,	172,
73.9018,	164,
73.9212,	166,
73.9407,	152,
73.9601,	167,
73.9796,	179,
73.9991,	164,
74.0185,	162,
74.038,	184,
74.0574,	162,
74.0769,	194,
74.0963,	154,
74.1158,	187,

74.1353, 157,

74.1547, 153,

74.1742, 156,

74.1936, 175,

74.2131, 152,

74.2326, 152,

74.252, 159,

74.2715, 188,

74.2909, 176,

74.3104, 158,

74.3298, 176,

74.3493, 166,

74.3688, 174,

74.3882, 153,

74.4077, 198,

74.4271, 174,

74.4466, 154,

74.466, 173,

74.4855, 180,

74.505, 163,

74.5244, 174,

74.5439, 178,

74.5633, 156,

74.5828, 175,

74.6023, 181,

74.6217, 182,

74.6412, 183,

74.6606,	160,
74.6801,	161,
74.6995,	165,
74.719,	158,
74.7385,	181,
74.7579,	157,
74.7774,	155,
74.7968,	172,
74.8163,	165,
74.8358,	189,
74.8552,	174,
74.8747,	173,
74.8941,	171,
74.9136,	156,
74.933,	184,
74.9525,	172,
74.972,	175,
74.9914,	185,
75.0109,	176,
75.0303,	165,
75.0498,	186,
75.0693,	171,
75.0887,	151,
75.1082,	154,
75.1276,	187,
75.1471,	165,
75.1665,	163,

75.186, 173,

75.2055, 165,

75.2249, 197,

75.2444, 173,

75.2638, 178,

75.2833, 177,

75.3028, 149,

75.3222, 150,

75.3417, 162,

75.3611, 154,

75.3806, 179,

75.4, 181,

75.4195, 171,

75.439, 191,

75.4584, 189,

75.4779, 162,

75.4973, 176,

75.5168, 160,

75.5363, 162,

75.5557, 188,

75.5752, 185,

75.5946, 166,

75.6141, 155,

75.6335, 172,

75.653, 153,

75.6725, 156,

75.6919, 174,

75.7114, 166,

75.7308, 184,

75.7503, 175,

75.7698, 164,

75.7892, 169,

75.8087, 168,

75.8281, 176,

75.8476, 174,

75.867, 161,

75.8865, 171,

75.906, 176,

75.9254, 157,

75.9449, 180,

75.9643, 187,

75.9838, 167,

76.0033, 171,

76.0227, 175,

76.0422, 161,

76.0616, 177,

76.0811, 171,

76.1005, 192,

76.12, 160,

76.1395, 153,

76.1589, 159,

76.1784, 141,

76.1978, 163,

76.2173, 161,

76.2368, 173,

76.2562, 180,

76.2757, 168,

76.2951, 193,

76.3146, 166,

76.334, 157,

76.3535, 174,

76.373, 180,

76.3924, 168,

76.4119, 148,

76.4313, 171,

76.4508, 170,

76.4703, 167,

76.4897, 168,

76.5092, 187,

76.5286, 165,

76.5481, 159,

76.5675, 177,

76.587, 155,

76.6065, 174,

76.6259, 174,

76.6454, 154,

76.6648, 155,

76.6843, 191,

76.7038, 191,

76.7232, 195,

76.7427, 181,

76.7621,	160,
76.7816,	168,
76.801,	145,
76.8205,	148,
76.84,	178,
76.8594,	168,
76.8789,	184,
76.8983,	163,
76.9178,	173,
76.9373,	146,
76.9567,	167,
76.9762,	210,
76.9956,	193,
77.0151,	171,
77.0345,	186,
77.054,	171,
77.0735,	171,
77.0929,	182,
77.1124,	142,
77.1318,	171,
77.1513,	176,
77.1707,	168,
77.1902,	170,
77.2097,	169,
77.2291,	156,
77.2486,	160,
77.268,	180,

77.2875,	153,
77.307,	176,
77.3264,	157,
77.3459,	172,
77.3653,	161,
77.3848,	159,
77.4042,	173,
77.4237,	163,
77.4432,	173,
77.4626,	181,
77.4821,	157,
77.5015,	172,
77.521,	166,
77.5405,	192,
77.5599,	183,
77.5794,	165,
77.5988,	148,
77.6183,	186,
77.6377,	172,
77.6572,	165,
77.6767,	157,
77.6961,	173,
77.7156,	187,
77.735,	158,
77.7545,	159,
77.774,	165,
77.7934,	174,

77.8129,	198,
77.8323,	170,
77.8518,	188,
77.8712,	164,
77.8907,	168,
77.9102,	165,
77.9296,	179,
77.9491,	159,
77.9685,	177,
77.988,	164,
78.0075,	172,
78.0269,	173,
78.0464,	200,
78.0658,	172,
78.0853,	166,
78.1047,	178,
78.1242,	153,
78.1437,	151,
78.1631,	193,
78.1826,	166,
78.202,	155,
78.2215,	191,
78.241,	158,
78.2604,	160,
78.2799,	155,
78.2993,	195,
78.3188,	152,

78.3382, 176,

78.3577, 178,

78.3772, 183,

78.3966, 164,

78.4161, 159,

78.4355, 149,

78.455, 184,

78.4745, 162,

78.4939, 184,

78.5134, 178,

78.5328, 158,

78.5523, 167,

78.5717, 162,

78.5912, 206,

78.6107, 155,

78.6301, 200,

78.6496, 183,

78.669, 188,

78.6885, 163,

78.708, 174,

78.7274, 175,

78.7469, 156,

78.7663, 145,

78.7858, 167,

78.8052, 178,

78.8247, 171,

78.8442, 181,

78.8636,	179,
78.8831,	161,
78.9025,	192,
78.922,	182,
78.9415,	150,
78.9609,	156,
78.9804,	141,
78.9998,	172,
79.0193,	141,
79.0387,	174,
79.0582,	163,
79.0777,	156,
79.0971,	181,
79.1166,	199,
79.136,	197,
79.1555,	162,
79.175,	175,
79.1944,	158,
79.2139,	180,
79.2333,	174,
79.2528,	170,
79.2722,	187,
79.2917,	180,
79.3112,	143,
79.3306,	181,
79.3501,	173,
79.3695,	183,

79.389,	190,
79.4085,	172,
79.4279,	169,
79.4474,	161,
79.4668,	177,
79.4863,	183,
79.5057,	183,
79.5252,	159,
79.5447,	174,
79.5641,	173,
79.5836,	179,
79.603,	162,
79.6225,	177,
79.642,	159,
79.6614,	149,
79.6809,	151,
79.7003,	183,
79.7198,	188,
79.7392,	174,
79.7587,	173,
79.7782,	166,
79.7976,	200,
79.8171,	182,
79.8365,	190,
79.856,	159,
79.8755,	155,
79.8949,	186,
